# Establishing Long-Term Efficacy in Chronic Disease: Use of Recursive Partitioning and Propensity Score Adjustment to Estimate Outcome in MS

**DOI:** 10.1371/journal.pone.0022444

**Published:** 2011-11-30

**Authors:** Douglas S. Goodin, Jason Jones, David Li, Anthony Traboulsee, Anthony T. Reder, Karola Beckmann, Andreas Konieczny, Volker Knappertz, and the 16-Year Long-Term Follow-up Study Investigators

**Affiliations:** 1 Department of Neurology, University of California San Francisco, San Francisco, California, United States of America; 2 Intermountain Healthcare, Salt Lake City, Utah, United States of America; 3 University of British Columbia, Vancouver, British Columbia, Canada; 4 Department of Neurology, University of Chicago, Chicago, Illinois, United States of America; 5 Bayer Schering Pharma AG, Berlin, Germany; 6 Lampe Konieczny & Company, Berlin, Germany; 7 Bayer HealthCare, Montville, New Jersey, United States of America; Institute Biomedical Research August Pi Sunyer (IDIBAPS) - Hospital Clinic of Barcelona, Spain

## Abstract

**Context:**

Establishing the long-term benefit of therapy in chronic diseases has been challenging. Long-term studies require non-randomized designs and, thus, are often confounded by biases. For example, although disease-modifying therapy in MS has a convincing benefit on several short-term outcome-measures in randomized trials, its impact on long-term function remains uncertain.

**Objective:**

Data from the 16-year Long-Term Follow-up study of interferon-beta-1b is used to assess the relationship between drug-exposure and long-term disability in MS patients.

**Design/Setting:**

To mitigate the bias of outcome-dependent exposure variation in non-randomized long-term studies, drug-exposure was measured as the *medication-possession-ratio*, adjusted up or down according to multiple different weighting-schemes based on MS severity and MS duration at treatment initiation. A recursive-partitioning algorithm assessed whether exposure (using any weighing scheme) affected long-term outcome. The optimal cut-point that was used to define “high” or “low” exposure-groups was chosen by the algorithm. Subsequent to verification of an exposure-impact that included all predictor variables, the two groups were compared using a weighted propensity-stratified analysis in order to mitigate any treatment-selection bias that may have been present. Finally, multiple sensitivity-analyses were undertaken using different definitions of long-term outcome and different assumptions about the data.

**Main Outcome Measure:**

Long-Term Disability.

**Results:**

In these analyses, the same weighting-scheme was consistently selected by the recursive-partitioning algorithm. This scheme reduced (down-weighted) the effectiveness of drug exposure as either disease duration or disability at treatment-onset increased. Applying this scheme and using propensity-stratification to further mitigate bias, high-exposure had a consistently better clinical outcome compared to low-exposure (Cox proportional hazard ratio = 0.30–0.42; p<0.0001).

**Conclusions:**

Early initiation and sustained use of interferon-beta-1b has a beneficial impact on long-term outcome in MS. Our analysis strategy provides a methodological framework for bias-mitigation in the analysis of non-randomized clinical data.

**Trial Registration:**

Clinicaltrials.gov NCT00206635

## Introduction

In general randomized controlled trials (RCTs) in many chronic diseases such as multiple sclerosis (MS) only establish the short-term efficacy of our current interventions [1]. Despite this, the main medical, social, and economic impacts of these diseases are typically caused by unremitting disability, which evolves slowly over many years. However, establishing whether a therapy alters long-term outcome of chronic disease is difficult because, in general, RCT designs are ill-equipped for this purpose. It is not realistic to continue a placebo arm after a drug has been demonstrated to alter short-term outcomes that are believed to be clinically relevant, especially after the therapy has been approved. In this circumstance, many patients will not consent to a prolonged placebo-exposure. Moreover, many clinicians are already prepared to accept such short-term outcome information as the basis for treatment decisions and, thus, would not recommend their patient's participation in a long-term placebo-controlled trial. Inevitably, therefore, establishing long-term efficacy for therapies of chronic diseases requires non-randomized observational study-designs. The pivotal-trial of interferon-beta-1b (IFNβ-1b; Betaseron®), begun in 1988, was the first successful trial of disease modifying therapy (DMT) in MS [2], [3]. The patient-cohort from this trial, which has now been followed out to 16 years, therefore, offers a unique opportunity to evaluate the efficacy of long-term DMT use in MS.

Despite this potential value, however, any such assessment faces several challenges. For example, when patients entered the RCT, they did so at very different points along the continuum of the MS disease course, with respect to both the disease-duration (i.e., the time since their first clinical symptom) and its severity (i.e., their disability status). If patient-characteristics such as these influence either the likelihood of responding to treatment or the likelihood of reaching a particular long-term outcome, baseline differences in these factors could potentially confound any assessment of long-term efficacy. Indeed, the RCT population had considerable variability on these measures (Table 1) and there are several pieces of evidence that suggest patients respond better to therapy earlier in their disease course [4]–[12].

**Table 1:** Baseline and on-RCT clinical characteristics of the patients included (and those not included) in the detailed LTF evaluation after 16 years*.

	Patients in theLTF population	Patients not inLTF population	p-value†
**Basleine Variables**			
Number of Patients	260**	112**	–
% women	69%	71%	0.7125
Age at disease onset; (years)	27.3 (6.8)	27.7 (7.3)	0.5361
Age at start of RCT; (years)	35.4 (7.4)	35.8 (6.7)	0.5220
EDSS	2.9 (1.3)	2.9 (1.3)	0.8373
EDSS ≥3; (% of population)	138 (53%)	62 (55%)	0.7343
Disease Duration; (yrs)	8.0 (6.2)	8.1 (6.3)	0.9950
Baseline MSSS	4.3 (2.3)	4.4 (2.2)	0.7118
Baseline, MRI T2 BOD; (cm^2^)	1.96 (2.0)	2.31 (2.4)	0.0699
Baseline, 3^rd^ ventricular width (mm)	4.86 (2.27)	5.19 (2.42)	0.1893
Annualized Relapse-rate (2 yrs prior to RCT)	1.68 (0.77)	1.67 (0.85)	0.5964
**On-Study (RCT) Variables**			
Annualized Relapse-rate	1.2 (1.2)	1.6 (2)	0.0849
Change, EDSS (actual)	0.0 (1.3)	0.3 (1.6)	0.2415
Number of new T2 CAL	2.4 (3.3)	3.0 (4.0)	0.1613
Change, MRI T2 BOD (cm^2^)	0.13 (0.6)	0.22 (1.0)	0.0729
Change, 3^rd^ ventricular width (mm)	0.62 (0.99)	0.63 (1.14)	0.7321
On IFNβ-1b (250 µg) during RCT (%)	37%	25%	0.0178

*Means are listed without parenthesis. Standard deviations are shown in parentheses.

**7 deceased patients included in the LTF population; 28 deceased patients not included in LTF population.

† P-value derived from Fisher's exact test for rates, z-score for percentages, and Wilcoxon's rank-sum test for all others.

LTF = long-term follow-up; EDSS = Expanded Disability Status Scale score; MSSS = Multiple Sclerosis Severity Score; BOD = burden of disease; CAL = Combined Active Lesions (New + Enlarging T2 Lesions).

Following the trial, the decision to start, to continue, or to switch therapy may also have been influenced by patient characteristics, thereby, leading to an imbalance (bias) between treated and non-treated patient-groups. Because such decisions are often based, in part, on the perceived response to treatment, such outcome-dependent variations in exposure (i.e., informed censoring) will also confound assessment of long-term efficacy. Patients doing well will stay on therapy, whereas patients doing poorly will stop or switch. Similarly, physician or patient preference for certain DMTs in certain circumstances may also lead to in an imbalance in baseline or other characteristics between groups. Because of these multiple potential sources of bias (Supplemental Material; Appendix S1; Table S1), assessment of long-term efficacy, of necessity, requires statistical methods that can mitigate these effects.

## Methods

### Patients

Data were collected from patients who agreed to participate in the 16-year long-term follow-up (LTF) of the IFNβ-1b pivotal trial [2], [3]. The design of the LTF study is described elsewhere [13] Briefly, patients from the original RCT [2], [3] were asked to participate in the LTF (see Protocol S1). Of the 372 patients in the RCT, 328 (88.2%) were identified and provided some information. Of these, 260 (70.0%; including the records from 7 who had died) underwent a detailed assessment of their treatment history and interim disease course (obtained through clinical evaluation and medical record review), and a determination of their outcome on a variety of long-term measures [13]–[16]. The extended disability status scale (EDSS) scores at treatment-onset ranged from 0 to 5.5, and treatment-exposure ranged from none (placebo-treated patients who did not initiate therapy) to 16 years (patients in the high-dose arm who continued therapy until the LTF evaluation).

Comparison of baseline characteristics between those who did and did not participate in the LTF indicated that both groups were substantially similar by all measures (Table 1). During the RCT, however, patients not participating in the LTF tended to have a slightly more aggressive disease course compared to those who participated (Table 1). Despite this tendency, a significantly greater percentage of patients participating in the LTF were from the group on IFNβ-1b (250 µg) during the RCT compared to those not participating (Table 1). Approval for the LTF study was obtained from institutional review boards of all participating centers. All patients gave written informed consent.

### Therapy

During the RCT, patients received placebo (n = 123), IFNβ-1b 50 µg (n = 125), or IFNβ-1b 250 µg (n = 124) subcutaneously every-other-day for 104 weeks [2], [3]. For our analysis, only the high-dose (250 µg) group was considered to have received optimal therapy at the time of randomization. This decision was made both because low-dose IFNβ-1b was less effective than the higher dose [2], [3] and because, on the basis of the evidence, the FDA declined to approve its use in MS patients.

After completion of the RCT, all patients were offered the opportunity of being treated with IFNβ-1b (250 µg) until FDA approval in 1993. No specific therapy was administered as part of the LTF although, until the 1996 approval of IFNβ-1a (Avonex®) and glatiramer acetate (GA, Copaoxone®), no other DMT was available. Nevertheless, over the 16 years, many patients received alternative DMTs [13]. However, IFNβ-1b accounted for the vast majority (>90%) of the overall time of DMT-exposure in the LTF. Consequently, our principal analysis only considered IFNβ-1b-exposure.

### Negative-outcome

“Hard” negative-outcomes were defined as reaching EDSS≥6.0, wheelchair use, conversion to secondary progressive (SP) MS, death, or the combined “any negative” outcome consisting of one or more of these other outcomes. These hard outcome measures are both clinically important and more reliable than scores in the lower EDSS range. For our principal analysis, the time to reaching EDSS≥6 required confirmation ≥3 months later. In addition to meeting this definition of confirmed progression, however, the patient was also required still to be EDSS≥6 at all subsequent evaluations including at the LTF evaluation. The purpose of this definition was to define the time at which a person reached “unremitting” EDSS≥6. SPMS was defined as progressive disability evolving over ≥12 months and, in the opinion of the investigator, not caused by relapses. Moreover, EDSS must have increased by ≥1 point over the previous 2 years. In addition, to be identified as SPMS, patients could not have reverted to RRMS subsequent to meeting these criteria.

Besides weighted treatment-exposure, several other variables potentially predictive of outcome were incorporated into the analysis, including age, gender, treatment assignment during the RCT, relapse-rate, EDSS, MS severity score (MSSS) [17], baseline T2-weighted MRI lesion count and volume, 3^rd^ ventricular width (a measure of atrophy), and neutralizing antibody (NAb) status during the first 3 years of therapy (Table 2). NAb-titers were measured in neutralizing-units per milliliter (NU/ml). NAb-status was divided into 7 categories, each of which could be combined with any (or several) of the other categories. These categories were defined as: 1) always negative (<20 NU/ml); 2) low titer (20–99 NU/ml); and 3) high-titer (one or more assays with ≥100 NU/ml but not consecutive); and 4) persistent high-titer (at least two consecutive titers ≥100 NU/ml during the pivotal trial). Each of these subcategories was further subdivided into titers that either reverted to NAb-negative status (at some time during the RCT) or remained persistently positive throughout the RCT.

**Table 2:** Recursive Partitioning associating each predictor variable independently with “any negative-outcome” (p<0.20 to split).

Parameter explored	Optimal split value	p-value	Interpretation
Age at 1^st^ symptom	No split	-	No relationship found
Age at entry to RCT	>37 years	0.11	Worse outcome with older age
Gender	No split	-	No relationship found
Time from 1^st^ symptom to RCT start	2.35 years	<0.001	Worse outcome with longer duration
Treatment during RCT	No split	-	No relationship found
Pre-RCT relapse-rate	No split	-	No relationship found
Baseline EDSS	Splits at >1,>2,>4	0.01<0.001<0.001	Worse outcome with higher baseline EDSS
Baseline MSSS	>2.93	<0.001	Worse outcome with higher baseline MSSS
Baseline MRI burden of disease (BOD)	>2005.5	<0.001	Worse outcome with higher baseline BOD
Baseline 3^rd^ ventricular width (atrophy)	>3.947	0.002	Worse outcome with greater 3^rd^ ventricular width
NAbs; any titer and any persistence during RCT	No split	-	No relationship found

Because complete MRI data was available for only about 75% of the sample, the entire analysis was run twice. In the first, each of the MRI variables (Table 1) were included. However, because none of these variables were selected as predictors of outcome, for the final analysis, these variables were omitted from consideration so that the sample size could be as large as possible. Nevertheless, importantly, the results of the analysis incorporating these MRI variables were not substantively different from that excluding them.

Cross-sectional cognitive data was obtained at the LTF examination. However, because a time-to-event analysis with bias mitigation was not possible for cognitive outcome, and because baseline testing was available in only a few patients, the cognitive data is presented elsewhere [15] and the impact of therapy on cognitive outcome could not be determined [16].

### Medication Possession Ratio (MPR) and Exposure Weighting

**Figure 1:**
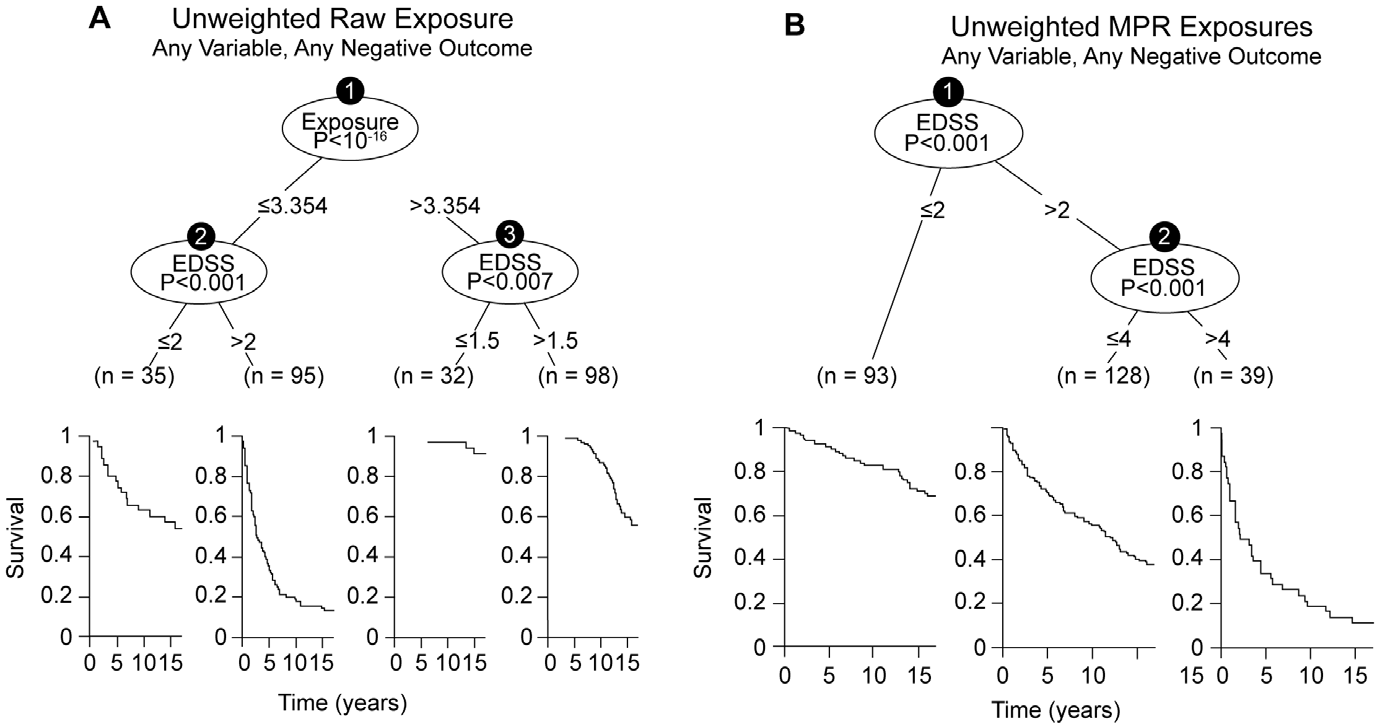
The effect of MPR transformation on the bias introduced by informative-censoring of exposure (see text). In panel A are the results of the RP analysis incorporating all of the baseline variables and the unweighted raw exposures (measured in years). In this analysis the exposure variable (in years) dominates all other variables with a p-value of 10^−16^. However, in panel B, where the same analysis is conducted using the unweighted-MPRs (in place of the unweighted “raw” exposures), the entire “spurious” treatment-effect disappears and the resulting tree is identical to that found when all predictor variables (but not treatment) are included in the RP-analysis. In both Panels, the Kaplan-Meier survival estimates are displayed below each of the identified subgroups (splits). X-axis is time in years. Y-axis is survival in % (1 = 100%).

**Figure 2:**
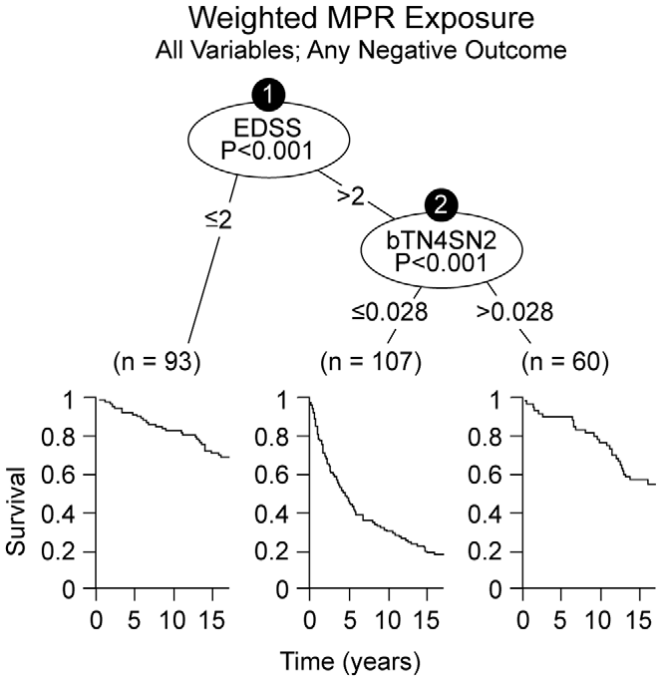
Optimal split determined by the recursive partitioning algorithm considering all predictor variables (**Table 2**) together with all possible weighted-MPR exposures to IFNβ-1b. Two highly significant split-levels were identified by the algorithm based on EDSS at the start of therapy and weighted IFNβ-1b exposure during the LTF. The first split (as in the Supplemental Material; Appendix S1; Figure S6) occurred at EDSS = 2, whereas a second level split occurred only for the EDSS>2 branch and was based on DMT exposure. Importantly, the algorithm for this analysis selected precisely the same weighting-scheme (bTN4SN2) that was selected in the Supplemental Material (Appendix S1; Figure S7). Survival curves are displayed below the identified subgroups and survival is best in the EDSS≤2 and the high-exposure groups. The split-point for DMT exposure is slightly different than that identified in the Supplemental Material (Appendix S1; Figure S7) because, in this instance, the split-point was determined only from the subgroup of patients with an EDSS>2. Note: the number (0.028) cannot be interpreted in time units because it represents a mathematical transformation from the raw exposure in years. In both Panels, the Kaplan-Meier survival estimates are displayed below each of the identified subgroups (splits). X-axis is time in years. Y-axis is survival in % (1 = 100%).

Data analysis was undertaken as a step-wise process. The first step (Supplemental Material; Appendix S1; Table S2; Step 1, A) was to measure treatment-exposure, not as years of IFNβ-1b 250 µg therapy, but to transform this variable into the so-called “medication possession ratio” or MPR [18], [19]. The purpose of this transformation was to compensate for the bias introduced by physicians or patients making treatment decisions based on perceived efficacy (i.e., outcome-dependent censoring of exposure) [20]. Thus, the MPR was defined individually as:
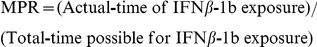



This is called the “raw” or “unweighted” MPR. The efficiency of the “raw” MPR the mitigating the bias of informative censoring (Supplemental Material; Appendix S1; Table S1) is amply demonstrated by comparison of an analysis using the actual (unweighted) years of IFNβ-1b-exposure to the same analysis following “unweighted” MPR transformation (Figure 1). Using “unweighted” treatment exposure, the significance of the treatment effect was (p<10^−16^). By contrast, using “unwieghted” MPR exposures, this apparent treatment effect is completely mitigated, indicating that the initial observation was due to bias. It was only after the possibility of exposure-weighting was introduced, that treatment re-emerged as significant factor associated with outcome (Figures 1 and 2).

Thus, these “raw” MPRs were weighted to account optimally for the possibility that a patient's response to therapy might be different based on how long they had had their illness and how disabled they were when therapy was started (see Supplemental Material; Appendix S1 for details).

### Creating Treatment Groups

The best MPR weighting-scheme was taken to be the one associated most significantly with a particular negative-outcome using a recursive-partitioning (RP) algorithm. The RP method is a two-step process [21], [22]. The first step (Supplemental Material; Appendix S1; Table S2; Step 1, B) identified which variable (in this case, which weighted-exposure) was most significantly associated with the particular negative-outcome [23]. The second step (Supplemental Material; Appendix S1; Table S2; Step 2, B) used recursive methods to identify the optimal split-point for the data (Supplemental Material; Appendix S1; Figure S1) [24]–[26]. Statistical significance was based on survival curves and associated log-rank tests for the subgroups being compared (Supplemental Material; Appendix S1; Figure S1) and the final significance adjusted (using a Bonferroni correction) for the number of variables and weighting schemes considered (Supplemental Material; Appendix S1).

The selected split-point for optimal-exposure (Supplemental Material; Appendix S1; Figure S1) defined the exposure-groups used in the final, integrated analysis (Supplemental Material; Appendix S1; Table S2; Step 2, D). In theory, the RP algorithm could have identified several weighted-MPR-exposure subgroups although, in the LTF dataset, evidence was found for only two. Although the split was based on weighted-MPR values, the difference between the two groups in unweighted-MPRs was notable. Thus, the median MPR in the high-exposure-group was 71% compared to a median of 0% in the low-exposure-group.

The RP algorithm was also applied to each of the predictor variables individually (Table 2) to explore preliminarily the relationships of these predictors to outcome. A “no-split” condition is defined for a given variable when that all of the possible splits were non-significant (p>0.2). As an interim verification model (Supplemental Material; Appendix S1; Table S2; Step 2, C), we re-ran the RP algorithm including all predictor variables together with all 161 weighted-MPR schemes (Supplemental Material; Appendix S1). This was done both to control the overall experiment-wise Type I error and to verify the homogeneity of the selected weighting-scheme and treatment effect after taking these other predictor variables into account. Theoretically, although it did not occur, the RP algorithm could have selected (at this step) a different weighting-scheme than that which was selected previously (Supplemental Material; Appendix S1; Table S2: Step 2, A). Such an occurrence might indicate heterogeneity in response-behavior between different subgroups of patients. If so, the entire analysis would need to be re-run considering each subgroup separately.

**Figure 3:**
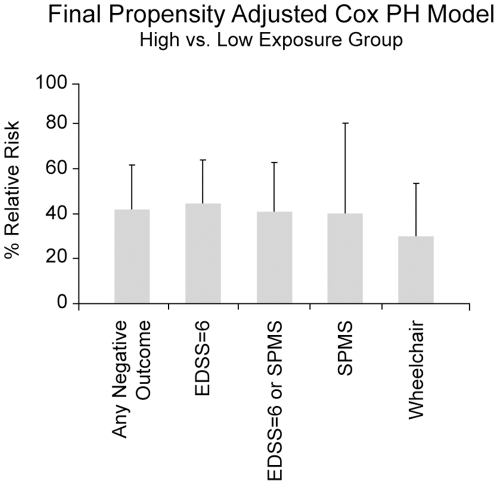
Propensity adjusted Cox proportional hazard estimates for the effect of treatment on each of the “hard” negative-outcomes examined in the study. The results for our principal analysis (i.e., for “any negative-outcome”) are shown on the far left. For each of these outcomes, there is approximately a 60% to 70% long-term benefit to therapy. Error bars indicate the 95% CI for each the different outcomes.

We also examined the stability and robustness of the model using multiple sensitivity analyses including different definitions of “hard” negative-outcome (i.e., EDSS≥6, SPMS, wheelchair use, death, either EDSS≥6 or SPMS, and “any negative-outcome”), different assumptions about the underlying data, different modeling approaches, and a variety of bootstrap methods (Supplemental material; Appendix S1). Each of these demonstrated that the model was quite stable and robust (Figures 3, Supplemental Material; Appendix S1; Figures S4 and S5; Table S3).

### Integrated Analysis

Final data analysis was done using a Cox proportional hazard model to estimate the risk of a negative-outcome based on exposure-group. To mitigate treatment-selection bias, a propensity scoring approach was used [27]–[32]. Logistic regression was used to estimate the likelihood that a patient would be in the “high” or “low” exposure-group using all available baseline characteristics (Supplemental Material; Appendix S1; Table S2; Step 2, D). The resulting likelihood estimates were then ranked to create bins in which patients were equally likely to be in either exposure-group. The Cox Regression model then used the propensity bin as a stratification variable and strata pooled to estimate the overall treatment-effect (Supplemental Material; Appendix S1; Table S2; Step 3, A). Multiple sensitivity analyses were also undertaken to assess the stability of the relationship between exposure-group and negative-outcome and to assess the stability of the derived relationships (Supplemental Material; Appendix S1) [33].

### Statistical tests of significance

The significance of differences between subgroups for all RP analyses was determined by the log-rank test and all reported p-values, including the verification model with all variables simultaneously considered (Supplemental Material; Appendix S1; Table S2; Step 2, C), were adjusted for the multiple comparisons (i.e., multiple potential predictor variables and multiple weighting schemes) using a Bonferroni correction. If the treatment effect was still significant after this analytic step (Supplemental Material; Appendix S1; Table S2; Step 2, C), then the final propensity-adjusted model (Supplemental Material; 1; Table S2; Step 3, A) was generated via Cox regression and the p-value for this final analysis was unadjusted for prior analysis steps.

## Results

Each predictor variable was evaluated by the RP algorithm independently and EDSS was the variable most strongly associated with negative-outcome (Table 2). When EDSS was evaluated alone, the RP algorithm identified four subgroups (Supplemental Material; Appendix S1; Figure S6). When all predictor variables were included (Table 2), the RP algorithm split only on EDSS and the tree was identical, except that the EDSS = 1 split (Figure S6; Supplemental Material; Appendix S1) was eliminated by Bonferroni adjustment (i.e., as in Figure 1-B). Baseline EDSS was also a highly significant predictor of outcome using standard logistic-regression analysis [14] In both analyses, the EDSS out-performed the MSSS.

Survival curves for “high” and “low” exposure to IFNβ-1b, demonstrate a significant benefit to “high” exposure (Supplemental Material; Appendix S1; Figure S7). The results of our initial validation (Supplemental Material; Appendix S1; Table S2; Step 2, C), when all predictor variables (Table 2) and all the weighted-MPR exposure variables are included together in the same analysis, indicate that only baseline EDSS and weighted treatment-exposure remain as significant factors in determining long-term outcome (Figure 2). Although Figure 2 might seem to indicate that only patients with an EDSS>2.0 derived benefit from therapy, there are two reasons to doubt this conclusion. First, because of the weighting scheme selected, those patients who entered the RCT with an EDSS>2.0, would have had a better response if they were treated earlier. Second, this analysis represents only an interim step to establish the independent contribution of therapy to outcome. The principal, and intended final, analysis was the propensity adjusted Cox model (see below).

Regardless of this nuance, however, it is of note that, despite treatment having a very significant impact on outcome in this analysis, the presence or absence of NAbs in the high-dose arm of the RCT (at any titer level or any degree of persistence or in any combination) did not mitigate this therapeutic effect. Also, when all of the variables are considered simultaneously, the same weighting-scheme is selected. As anticipated, there is a strong correlation between treatment assignment during the RCT and the weighted MPR. This is because the patients in the high-dosage arm had a 100% MPR for the first 2–5 years whereas the patients in the other arms had a 0% MPR for this same interval. Nevertheless, despite this correlation, there was no discernable association between treatment assignment and long-term outcome (Table 2).

The results of our final analysis (Supplemental Material; Appendix S1; Table S2; Step 3, A) showed very similar results. Thus, the propensity-adjusted analysis using the optimal weighting-scheme, found that the high-exposure-group much less likely to experience “any negative-outcome” compared with the low-exposure-group (HR = 0.423; 95% CI; 0.275, 0.651; p<0.0001). A similar result was found for every hard-outcome considered in this trial (Figure 3) and was confirmed by each of the sensitivity analyses performed (Supplemental Material; Appendix S1).

One of the underlying assumption of the Cox model is that of proportionate hazard. We, therefore, considered the potential impact of a violation of proportionate hazard and, indeed, this assumption only holds until approximately 10 years after RCT onset (Supplemental material; Appendix S1; Figure S8). After this point point, the patients with EDSS>2 and more exposure begin to fail at a greater rate than patients with EDSS>2 and less exposure (Supplemental material; Appendix S1; Figure S8;). The only way to satisfy the proportional hazards assumption is to truncate the data at 10 years. However, censoring the data at this point actually leads to a more extreme hazard ratio and a more significant difference between the two groups. Such an outcome is anticipated because any violation of the proportionate hazard assumption should be biased toward the null hypothesis.

## Discussion

The results of this study provide several pieces of important evidence about the relationship between the use of IFNβ-1b therapy and long-term outcome in MS. First, therapy seemed to have a consistent benefit on several measures of disability or progression including EDSS, use of a wheel chair, or progression to SPMS, and the combined measure of “any negative-outcome”. In this respect, our findings are similar to those reported previously by Trojano and colleagues [31] which, after propensity adjustment, demonstrated that 7 years of IFNβ therapy reduced disease progression as determined by the time to reach EDSS = 4, EDSS = 6, and SPMS. Second, not only did every such analysis (except death) show a statistically convincing and very similar effect (Figure 3; Supplemental Material; Appendix S1; Table S3), the magnitude of the benefit was also quite striking (Figures 2 and 3) with hazard ratios between 0.30 and 0.42. These two observations provide evidence that the use of IFNβ-1b results in a significant and clinically meaningful impact on long-term function in MS patients. Importantly, although there is a strong (and expected) correlation between treatment assignment during the RCT and the weighted MPR, there was no discernable association of this variable with outcome (Table 2). This indicates that the observed benefit of therapy is not merely a reflection of this early DMT experience. Importantly, also, our study provides no information about the value of other DMTs because over 90% of both the actual and the weighted-MPR exposure was from treatment with IFNβ-1b. This circumstance is due to two factors. First, IFNβ-1b was the only therapy available for the initial 6–8 years of the LTF. Second, the effect of any late DMT-exposure was considerably down-weighted as a result of the selected response curves (Supplemental Material; Appendix S1; Figure S3). Therefore, our sensitivity-analysis of “any DMT-exposure”, in reality, merely recapitulates our analysis using IFNβ-1b-exposure alone.

Third, the weighting-schemes chosen by every one of the different analyses were very consistent (Supplemental Material; Appendix S1; Table S3), suggesting that this scheme is probably accurate about when and how IFNβ-1b is most effective. The selected response curves (Supplemental Material; Appendix S1; Figure S3) resulted heavy down-weighting with increasing EDSS score and longer disease duration at therapy-onset. This suggests that therapy is more effective when given early in the disease course and is consistent with RCTs of both early MS (where the effect size seems greater) and SPMS (where the effect size seems less) compared to RCTs of relapsing-remitting MS [2], [3], [6]–[8], [11], [34]–[45]. It is also consistent with the observation that the on-therapy event-rates have fallen precipitously (even for the same medications) in the last 5 years compared to the first 10 years of the DMT era (Supplemental Material; Appendix S1; Figure S9). There is little question that less advanced patients are now being recruited into current randomized trials compared to earlier trials and this marked change in event-rate suggests that patients treated earlier in their disease course are responding much better to DMT medication, just as one would expect from the selected response curves shown in the Supplemental Material (Appendix S1; Figure S3). In sum then, this analysis consistently supports the value of early therapy. Moreover, this result seems unlikely to be due to ascertainment bias (Supplemental Material; Appendix S1; Table S1). Thus, despite a tendency to include, in the LTF, patients who had somewhat less aggressive disease during the RCT (Table 1), a greater percentage of the LTF patients were from the group on IFNβ-1b (250 µg) during the RCT compared to those not participating (Table 1). Thus, the anticipated bias produced by this imbalance should underestimate the benefit of treatment because untreated patients (doing poorly during the RCT) seem selectively less likely to have participated in the LTF.

This analysis also provides evidence for the utility of a methodological framework that mitigates bias in the estimation of long-term efficacy from non-randomized, observational, clinical trials. This framework is outlined in the Supplemental Material (Appendix S1; Table S2) and consists of several analytic steps, which are also referenced throughout the description of our analytic methods. The first step consists of data preparation and the transformation of absolute exposures into raw-MPR exposures. This step is critical because it converts the raw data, which are heavily biased by informative-censoring of exposure due to a patient's perception of their own response, into data that are corrected for this bias [20]. The second step consists of applying weighting-schemes to allow flexibility in defining the exposure-response relationship. This step is also critical because, by making no adjustment to the raw-MPR data, the relationship between exposure and outcome is forced to follow the bP0 (or eP0) weighting-scheme (Supplemental Material; Appendix S1; Figure S2). The third step is to use the RP algorithm to select the optimal weighting-scheme, to define the exposure-groups, and to run preliminary validation of the model. At the third step, the analysis can fail and the process terminated. Thus, if no significant split-point is found in the data during the selection of the optimal weighting-scheme, this suggests that there is no relationship between exposure and outcome. In this case, however, it is still worthwhile repeating the analysis in the validation step (Supplemental Material; Appendix S1; Table S2; Step 2, C) to be sure that a sub-group of patients is not responding. In this case, the analysis should be repeated including only the identified sub-group. If neither the initial RP nor the validation RP demonstrates a significant treatment-effect, the analysis is terminated. Similarly, if a significant split-point is initially found for treatment-exposure but this relationship disappears when other predictor variables are included in the RP algorithm, the analysis is terminated. If none of these termination-events occurs, however, then logistic regression is used to create propensity bins (based on the treatment groups identified in step 3) and a propensity-stratified survival analysis using a Cox proportional hazard model is undertaken to give an estimate for the size of the treatment effect. This step is also critical because propensity adjustment will mitigate any treatment-selection bias, provided that the baseline variables (on which treatment decisions were made) have been captured [27]–[32]. Indeed, using the method of propensity score adjustment, other authors have also reported a beneficial impact of therapy on outcome [31]. The last step is to perform multiple sensitivity-analyses utilizing different definitions of negative-outcome, different assumptions about the underlying data, and different modeling approaches to ensure that the findings are both consistent and robust. In summary, and, as exemplified by our analysis of the LTF data from the IFNβ-1b pivotal trial, utilization of this analytic strategy can provide a useful method for the minimization of bias in the analysis of non-randomized long-term data.

## Supporting Information

Figure S1 Derivation of split-points by the RP algorithm after selection of the optimum baseline variable (Panel A) and the optimum weighting-scheme (Panel B). In the case of the EDSS there are only 10 possible split-points from which to choose because the EDSS has only 10 possible split points from 0 to 5.5. Panel B has many more data points because weighted MPR could potentially be divided at many split-points. In both cases, however, the algorithm picks the maximum value of the test statistic for group-comparisons (in this case the log-rank test) to define the best split-point. In Panel A the split could have been at either at EDSS = 2 or EDSS = 3, as the test statistic was very similar at these two points. In Panel B the choice is more clear-cut. Also note that the actual of the “weighted” MPR is not meaningful because it represents a mathematical transformation of the raw exposure data in years into something that can't be interpreted in unit of time (see Figure S3).
(TIF)

Figure S2 The theoretical diffusion curves used for selecting weighting-schemes by the recursive partitioning algorithm. Both Bass diffusion curves (b) and exponential curves (e) were used (see text). Curves that represent increasing effectiveness of therapy with a increasing disease duration or EDSS score are called positive (P). By contrast, those that represent decreasing therapeutic effectiveness with increasing disease duration or EDSS are called negative (N). From these 17 curves, it can be appreciated that this collection (and selecting them in pairs) provides considerable flexibility to the RP algorithm such that essentially any exposure-weighting can be selected. Pairs of curves that could be selected include cases in which the MPR was decreased for one parameter (e.g., time since first symptom) and increased for the other (e.g., EDSS at therapy initiation) in addition to cases in which the MPR was increased (or decreased) for both parameters.
(TIF)

Figure S3 Calculation of the weighted-MPR for an individual beginning therapy after 2 years of disease and at an EDSS of 3.0. Panel A shows the selected (bTN4SN2) weighting scheme (i.e. the bN4 and bN2 curves – Figure S2). On the x-axis the term (EDSS·2) represents twice the EDSS score in order to simplify the graph. The designation (T) means that the time (disease duration) criteria is following bN4 curve whereas the designation (S) means that the severity (EDSS) criteria is following the bN2 curve (Figure 3). The table below the graph shows the calculation for the average weight. Panel B shows a two-dimensional graph of the same weighting-scheme as in Panel A for any combination of EDSS (Severity) and Duration (Time) at the onset of therapy, with 100% weighting (in dark blue) at the origin transitioning to 0% at the upper right corner (in red). The black square indicates this particular patient's location on this graph. Panel C shows how this weighting-scheme affects the MPR given this person's individual treatment history. The times that different outcomes were reached are indicated on the top line. A color-code for outcomes and treatments is at the bottom. Just prior to reaching EDSS = 6, this patient was switched to IFNβ-1a (Avonex®). Because the Avonex is started within 3 months of discontinuing to IFNβ-1b (Betaseron®), the weighting for the two drugs is the same. Because the other therapy was started more than 3 months after discontinuing to IFNβ-1a (Avonex®) and because it was started at such a long disease duration (∼10 yrs + the disease duration at the RCT start), it has been down-weighted to almost zero.
(TIF)

Figure S4 Percentage of the time that different weighting schemes were selected in the 2,000 samples selected using bootstrap methods (see text for details). The “optimal” weighting scheme (bTN4SN2) was chosen in 52% of the time. However, the next most commonly selected weighting schemes (bTN4SN3, eTN4SN3, eTN4SN4, and bTN1SN3) are similar to the bTN4SN2 scheme (Figures S2 and S3), with each markedly down-weighting the value of exposure both for an increased disease duration and for a greater EDSS at the start of therapy (see Figures S2 and S3). The solid black line shows the cumulative probability for the selected weighting schemes, with the first 5 schemes being selected more than 90% of the time. See Figure legends S2 and S3 for the definitions of b, e, P, N, T, and S.
(TIF)

Figure S5 Various tests of the stability of the Model. All panels A–D follow a similar structure. The leftmost, filled, black circle represents the original estimate observed using all 260 observations exactly once and the 5-bin propensity score adjustment method. For instance, the relative risk of any negative event was estimated to be 0.44 for the “more” treatment group compared to the low treatment group (p<0.001). After the “Original” estimate 3 sets of 3 other estimates are provided. The estimates are grouped first based on the weighting scheme that was selected. Immediately next to the “Original” estimate is the estimate that is observed only including the bootstrap samples where weighting scheme bTN4SN2 was selected, followed by bTN4SN3 and eTN4SN3 (the 2^nd^ and 3^rd^ ranked weighting schemes). Within each weighting scheme block, three modeling strategies are tested: (1) 5 bin propensity score adjustment, (2) stepwise selection with all terms included as covariates in the Cox Model, and (3) no adjustment (e.g., treatment effect is the only term entered into the model). A horizontal line extends from the “Original” point estimate for easy comparison. Vertical lines from each point estimate provide 95% confidence intervals based on the bootstrap sample and employing the empirical percentile confidence interval approach. In Panel A the effect size for treatment in the “high-exposure' group is shown. The horizontal dotted lines represent the 95% CI for the original analysis (including all of the 260 original observations). Panel B shows the levels of statistical significance for each analysis method and weighting scheme. The lower two panels (C and D) shows the predictive accuracy of the model (i,e, the area under the Receiver-Operator curve or the C-Index) for the bootstrap sample (”In Bag”) and for the observations not in the bootstrap sample (“Out of Bag” or OoB).
(TIF)

Figure S6 Optimal split determined by the RP algorithm considering only EDSS at the start of therapy. Two highly significant split-levels were identified by the algorithm based on EDSS at the trial entry. The first split occurred at EDSS = 2 and subsequent splits were found for both branches. Survival curves are displayed below each of the identified subgroups with survival markedly deteriorating with higher EDSS scores at trial entry. After including all predictor variables (except treatment) into the model, the secondary split-point at EDSS = 1 becomes non-significant after controlling for Type 1 error with a Bonferroni adjustment. Below the splits, the survival curves are plotted. X-axis is time in years. Y-axis is survival in % (1 = 100%).
(TIF)

Figure S7 Optimal split determined by the recursive partitioning algorithm considering only weighted-MPR exposure to IFNβ-1b. In this analysis, the RP algorithm was presented with all weighting-schemes (161) and selected the bTN4SN2 weighting-scheme as the one most closely associated with a negative-outcome. This is the one used in this analysis and the survival curves for the optimally split data are shown. The left-hand panel shows survival in the low-exposure group whereas the right-hand panel shows much better survival in the high-exposure group. Note: the number (0.034) cannot be interpreted in time units because it represents a mathematical transformation from the raw exposure in years. Below the splits, the survival curves are plotted. X-axis is time in years. Y-axis is survival in % (1 = 100%).
(TIF)

Figure S8 Superimposed survival curves for the same three groups presented previously in Figure 2 (Main paper). As can be appreciated from the Figure, the proportional hazard assumption only holds out to approximately 10 years. After that point, the patients with EDSS>2 and more exposure (blue line) begin to fail at a greater rate than patients with EDSS>2 and less exposure (red line). The only way to satisfy the proportional hazards assumption is to truncate the data at 10 years. Censoring the data at this point, however, actually leads to a more extreme hazard ratio and a more significant difference between the two groups. Such an outcome is anticipated because any violation of the proportionate hazard assumption should be biased toward the null hypothesis.
(TIF)

Figure S9 The on-therapy event-rate (annualized attack-rate) in clinical trials since the completion of the original IFNβ-1b (Betaseron®) trial^2, 3^ in 1992. Other trials include COP-1 (GA, Copaxone®)^33^; MSCRG (IFNβ-1a, Avonex®)^34^; PRISMS (IFNβ-1a, Rebif®)^35^; OWIMS (IFNβ-1a, Rebif®)^36^; EVIDENCE (Rebif® vs. Avonex®)^37^; INCOMIN (Betaseron® vs. Avonex®)^38^; AFFIRM (natalizumab, Tysabri®)^39^; SENTINEL (Avonex® vs. Avonex® plus Tysabri®)^34^; CamMS (alemtuzamab, Campath® vs. Rebif®)^41^; REGARD (Rebif® vs. Copaxone®)^9^; BEYOND (Betaseron® 500 µg vs. Betaseron® 250 µg vs. Copaxone®)^10^; CLARITY (Cladribine)^43^; TRANSFORMS (Fingolimod)^44^; and FREEDOMS (Fingolomid).^45^ These event-rates, even for the same study medications (Betaseron®, Rebif®, and Copaxone®), have fallen precipitously in the past 5 years, at least in part, because current trials tend to recruit patients with more mild disease (i.e., more patients with short disease courses and more patients with lower EDSS scores) compared to trials undertaken when no proven DMTs were available.
(TIF)

Table S1 Sources of Bias and Corrective Strategies.
(DOC)

Table S2 Strategy for bias-minimization in the analysis of non-randomized observational data.
(DOC)

Table S3 Sensitivity analyses using alternative definitions of outcome and exposure.
(DOC)

Protocol S1 (PDF)

Appendix S1 (DOC)
