# Supplementary figures and images for: Establishing Long-Term Efficacy in Chronic Disease: Use of Recursive Partitioning and Propensity Score Adjustment to Estimate Outcome in MS
Source: PLoS One. 2011 Nov 30;6(11):e22444. doi: 10.1371/journal.pone.0022444 (PMC3227563; doi:10.1371/journal.pone.0022444)

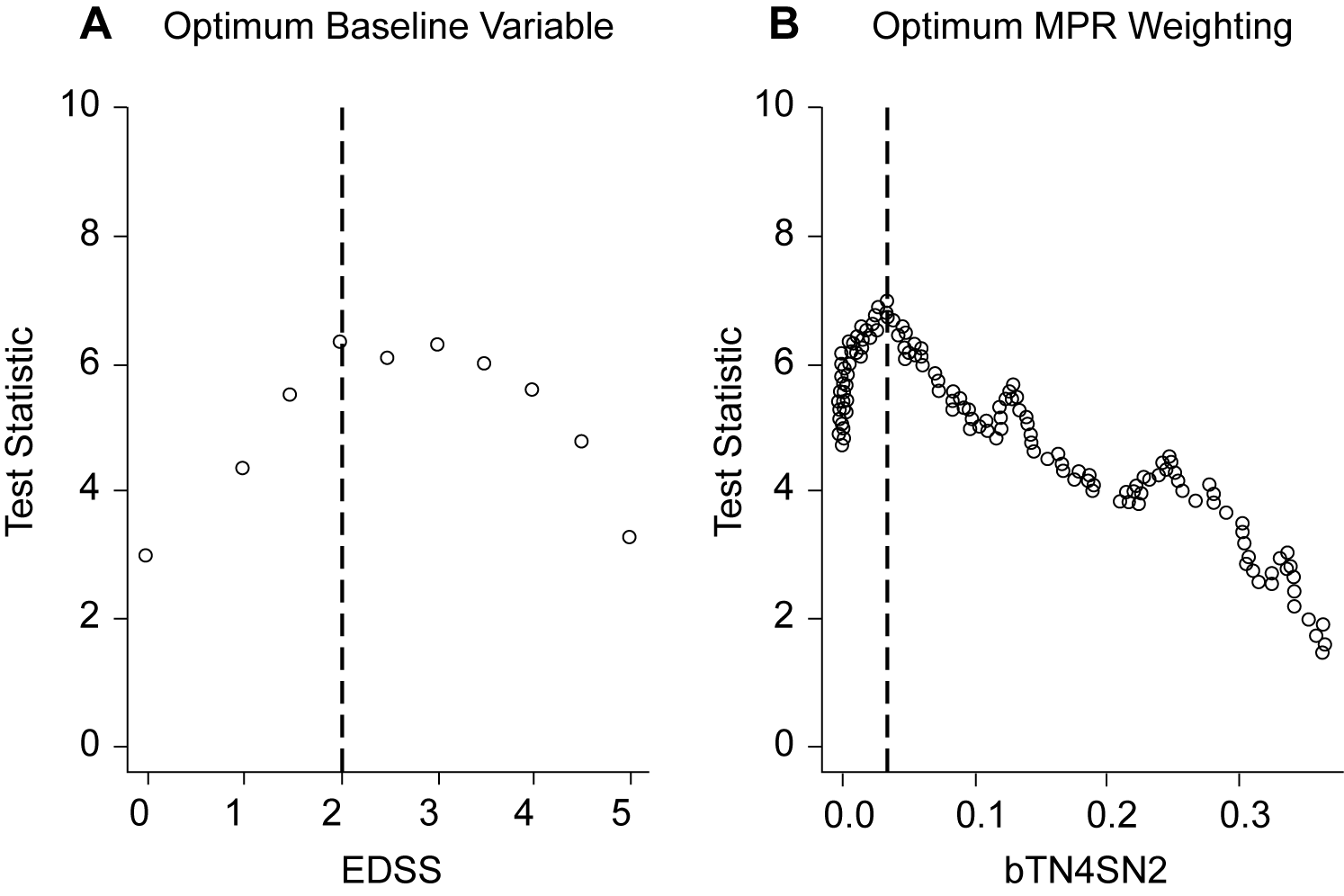

Supplement: Figure S1 — Derivation of split-points by the RP algorithm after selection of the optimum baseline variable (Panel A) and the optimum weighting-scheme (Panel B). In the case of the EDSS there are only 10 possible split-points from which to choose because the EDSS has only 10 possible split points from 0 to 5.5. Panel B has many more data points because weighted MPR could potentially be divided at many split-points. In both cases, however, the algorithm picks the maximum value of the test statistic for group-comparisons (in this case the log-rank test) to define the best split-point. In Panel A the split could have been at either at EDSS = 2 or EDSS = 3, as the test statistic was very similar at these two points. In Panel B the choice is more clear-cut. Also note that the actual of the “weighted” MPR is not meaningful because it represents a mathematical transformation of the raw exposure data in years into something that can't be interpreted in unit of time (see Figure S3). (TIF) [file pone.0022444.s001.tif]

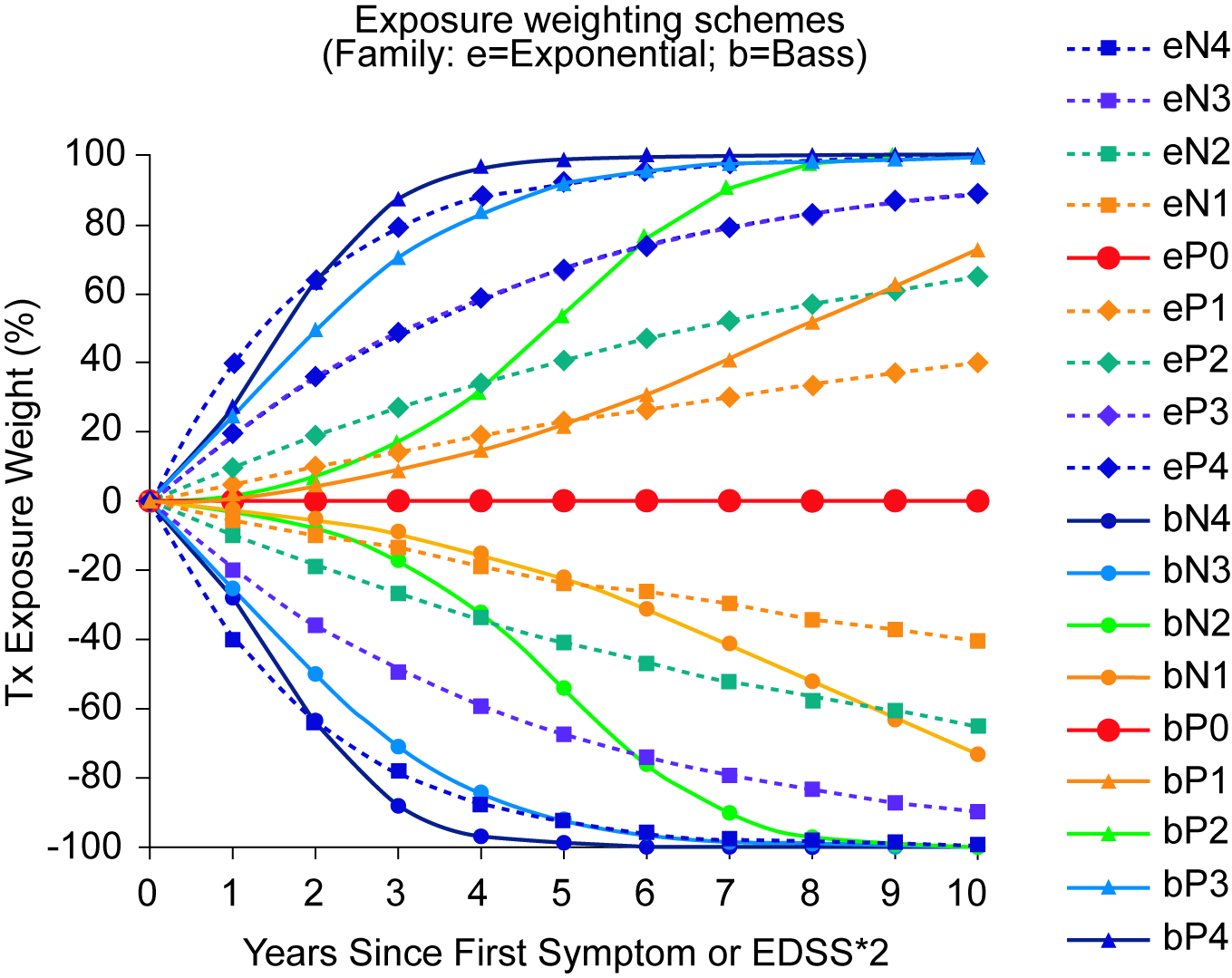

Supplement: Figure S2 — The theoretical diffusion curves used for selecting weighting-schemes by the recursive partitioning algorithm. Both Bass diffusion curves (b) and exponential curves (e) were used (see text). Curves that represent increasing effectiveness of therapy with a increasing disease duration or EDSS score are called positive (P). By contrast, those that represent decreasing therapeutic effectiveness with increasing disease duration or EDSS are called negative (N). From these 17 curves, it can be appreciated that this collection (and selecting them in pairs) provides considerable flexibility to the RP algorithm such that essentially any exposure-weighting can be selected. Pairs of curves that could be selected include cases in which the MPR was decreased for one parameter (e.g., time since first symptom) and increased for the other (e.g., EDSS at therapy initiation) in addition to cases in which the MPR was increased (or decreased) for both parameters. (TIF) [file pone.0022444.s002.tif]

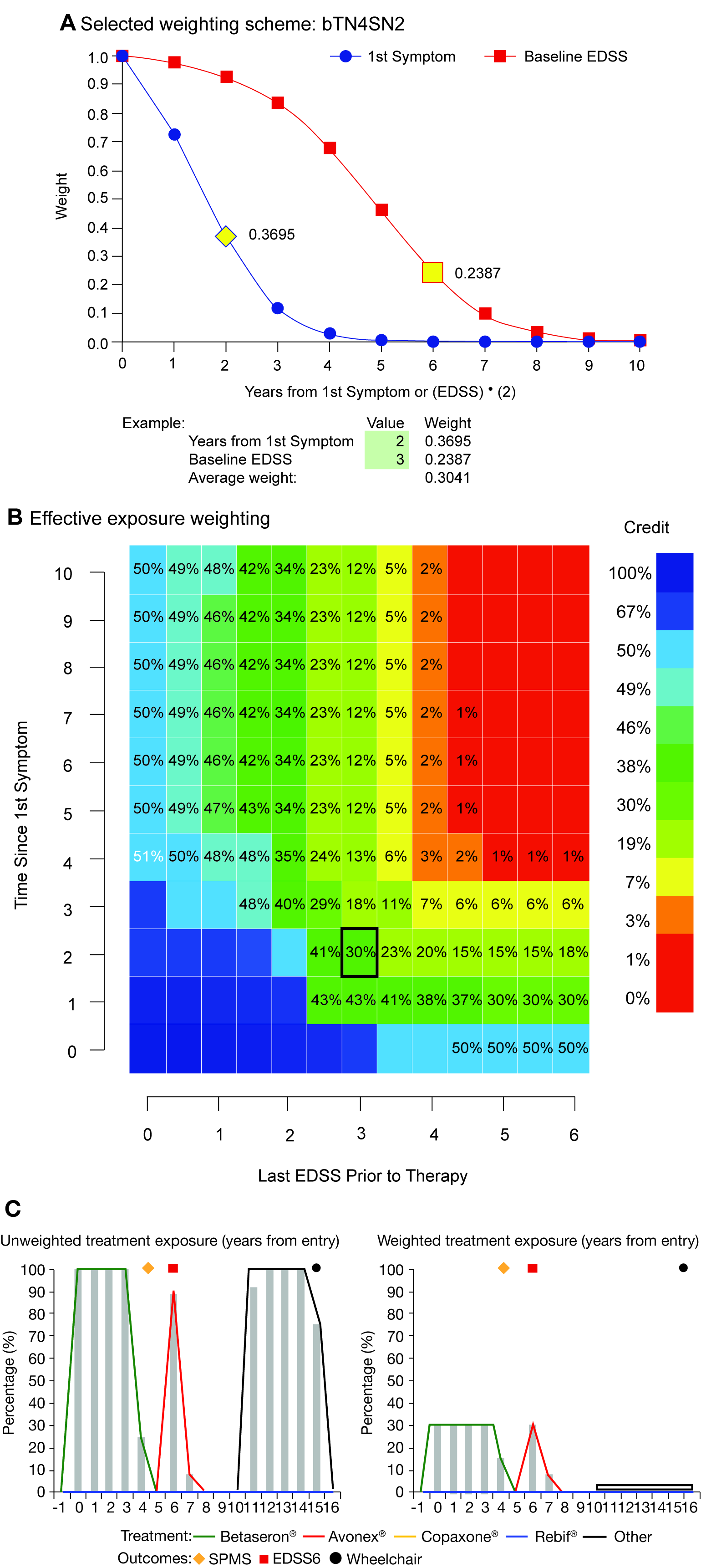

Supplement: Figure S3 — Calculation of the weighted-MPR for an individual beginning therapy after 2 years of disease and at an EDSS of 3.0. Panel A shows the selected (bTN4SN2) weighting scheme (i.e. the bN4 and bN2 curves – Figure S2). On the x-axis the term (EDSS·2) represents twice the EDSS score in order to simplify the graph. The designation (T) means that the time (disease duration) criteria is following bN4 curve whereas the designation (S) means that the severity (EDSS) criteria is following the bN2 curve (Figure 3). The table below the graph shows the calculation for the average weight. Panel B shows a two-dimensional graph of the same weighting-scheme as in Panel A for any combination of EDSS (Severity) and Duration (Time) at the onset of therapy, with 100% weighting (in dark blue) at the origin transitioning to 0% at the upper right corner (in red). The black square indicates this particular patient's location on this graph. Panel C shows how this weighting-scheme affects the MPR given this person's individual treatment history. The times that different outcomes were reached are indicated on the top line. A color-code for outcomes and treatments is at the bottom. Just prior to reaching EDSS = 6, this patient was switched to IFNβ-1a (Avonex®). Because the Avonex is started within 3 months of discontinuing to IFNβ-1b (Betaseron®), the weighting for the two drugs is the same. Because the other therapy was started more than 3 months after discontinuing to IFNβ-1a (Avonex®) and because it was started at such a long disease duration (∼10 yrs + the disease duration at the RCT start), it has been down-weighted to almost zero. (TIF) [file pone.0022444.s003.tif]

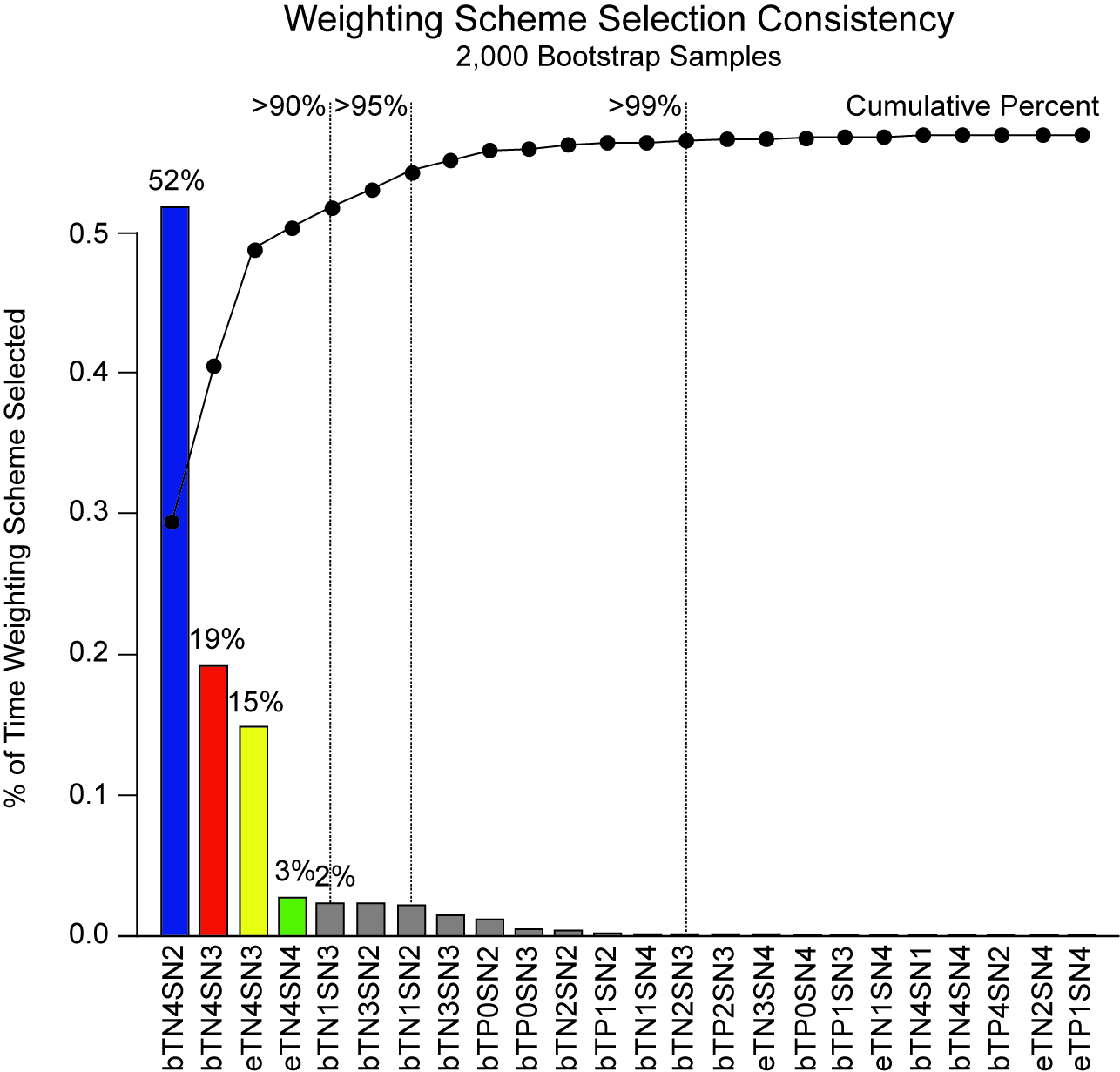

Supplement: Figure S4 — Percentage of the time that different weighting schemes were selected in the 2,000 samples selected using bootstrap methods (see text for details). The “optimal” weighting scheme (bTN4SN2) was chosen in 52% of the time. However, the next most commonly selected weighting schemes (bTN4SN3, eTN4SN3, eTN4SN4, and bTN1SN3) are similar to the bTN4SN2 scheme (Figures S2 and S3), with each markedly down-weighting the value of exposure both for an increased disease duration and for a greater EDSS at the start of therapy (see Figures S2 and S3). The solid black line shows the cumulative probability for the selected weighting schemes, with the first 5 schemes being selected more than 90% of the time. See Figure legends S2 and S3 for the definitions of b, e, P, N, T, and S. (TIF) [file pone.0022444.s004.tif]

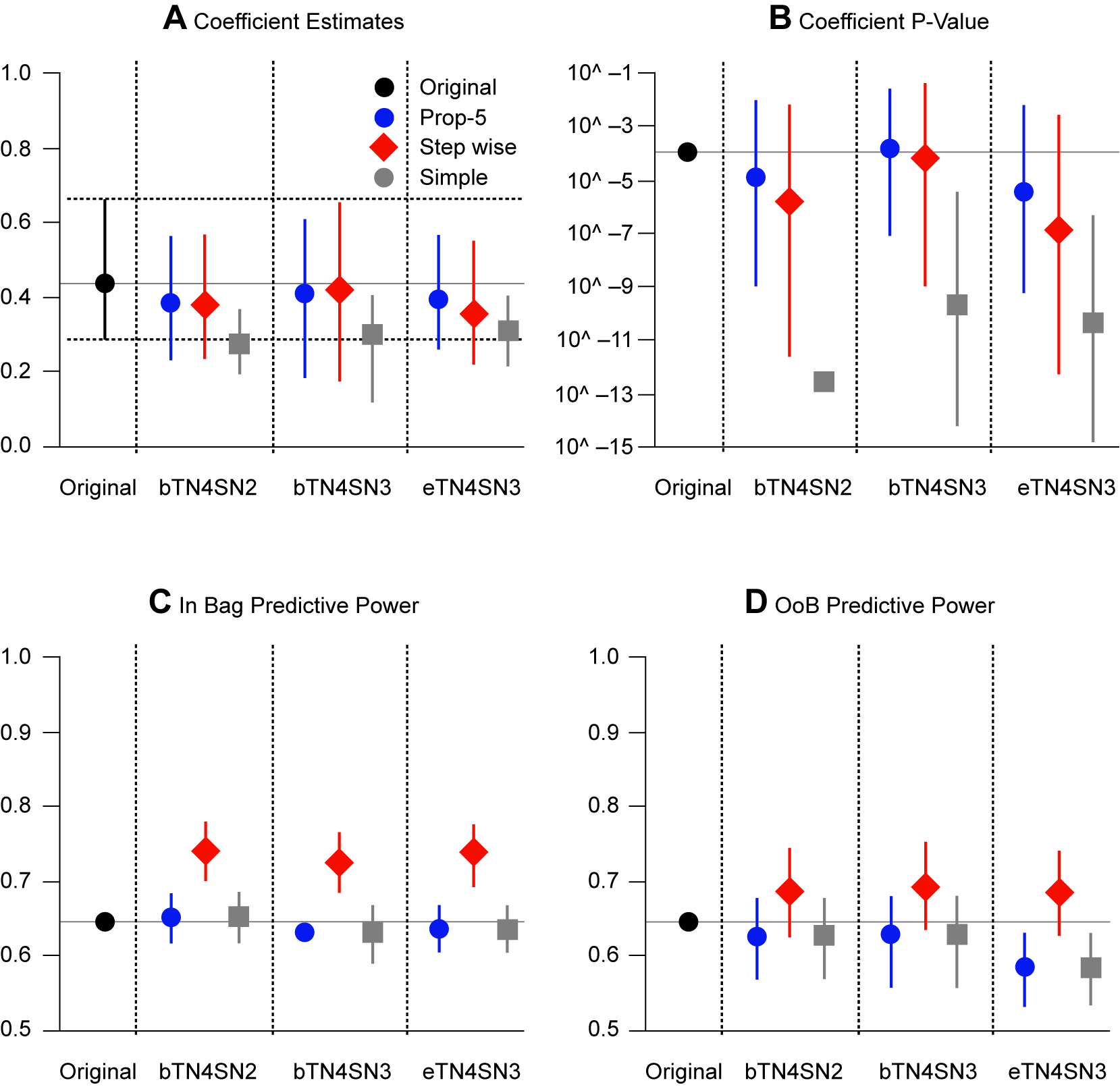

Supplement: Figure S5 — Various tests of the stability of the Model. All panels A–D follow a similar structure. The leftmost, filled, black circle represents the original estimate observed using all 260 observations exactly once and the 5-bin propensity score adjustment method. For instance, the relative risk of any negative event was estimated to be 0.44 for the “more” treatment group compared to the low treatment group (p<0.001). After the “Original” estimate 3 sets of 3 other estimates are provided. The estimates are grouped first based on the weighting scheme that was selected. Immediately next to the “Original” estimate is the estimate that is observed only including the bootstrap samples where weighting scheme bTN4SN2 was selected, followed by bTN4SN3 and eTN4SN3 (the 2nd and 3rd ranked weighting schemes). Within each weighting scheme block, three modeling strategies are tested: (1) 5 bin propensity score adjustment, (2) stepwise selection with all terms included as covariates in the Cox Model, and (3) no adjustment (e.g., treatment effect is the only term entered into the model). A horizontal line extends from the “Original” point estimate for easy comparison. Vertical lines from each point estimate provide 95% confidence intervals based on the bootstrap sample and employing the empirical percentile confidence interval approach. In Panel A the effect size for treatment in the “high-exposure' group is shown. The horizontal dotted lines represent the 95% CI for the original analysis (including all of the 260 original observations). Panel B shows the levels of statistical significance for each analysis method and weighting scheme. The lower two panels (C and D) shows the predictive accuracy of the model (i,e, the area under the Receiver-Operator curve or the C-Index) for the bootstrap sample (”In Bag”) and for the observations not in the bootstrap sample (“Out of Bag” or OoB). (TIF) [file pone.0022444.s005.tif]

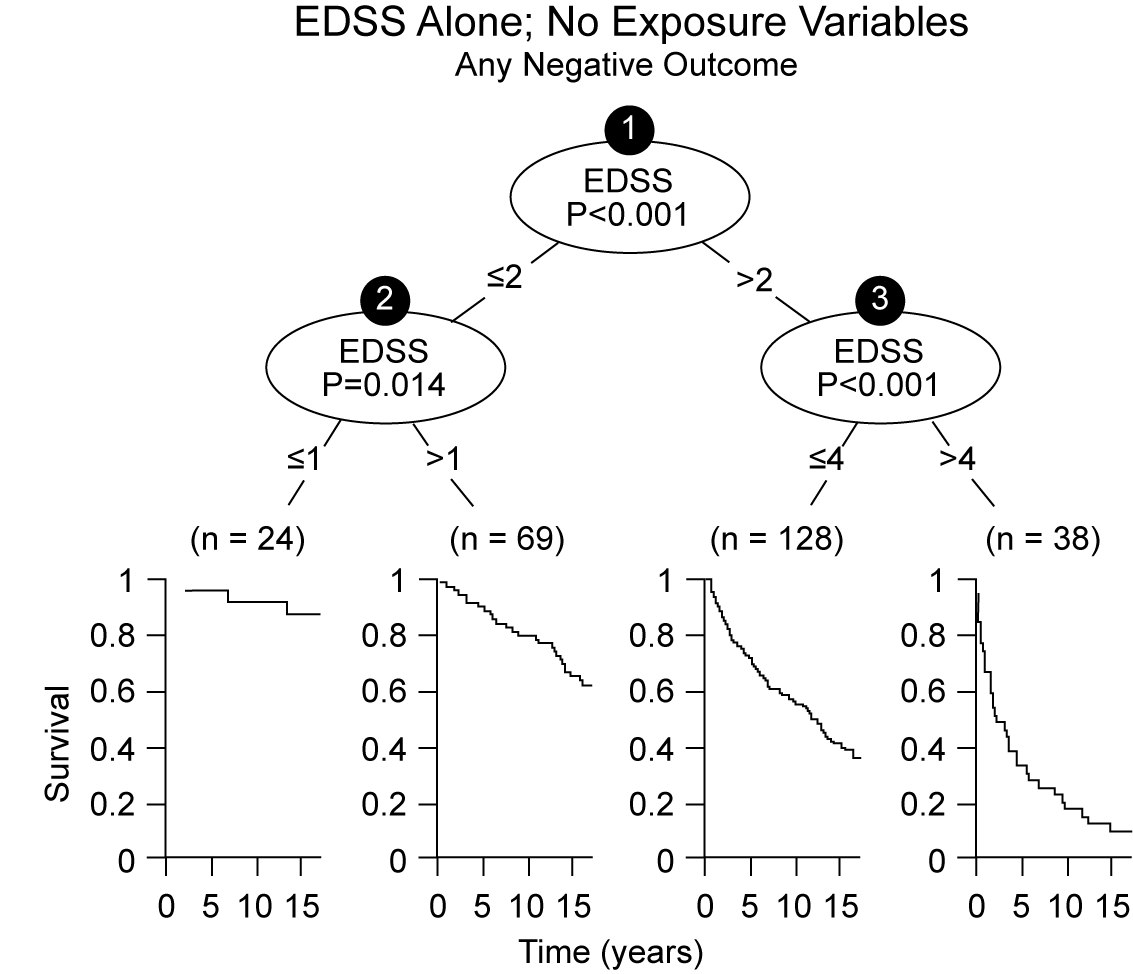

Supplement: Figure S6 — Optimal split determined by the RP algorithm considering only EDSS at the start of therapy. Two highly significant split-levels were identified by the algorithm based on EDSS at the trial entry. The first split occurred at EDSS = 2 and subsequent splits were found for both branches. Survival curves are displayed below each of the identified subgroups with survival markedly deteriorating with higher EDSS scores at trial entry. After including all predictor variables (except treatment) into the model, the secondary split-point at EDSS = 1 becomes non-significant after controlling for Type 1 error with a Bonferroni adjustment. Below the splits, the survival curves are plotted. X-axis is time in years. Y-axis is survival in % (1 = 100%). (TIF) [file pone.0022444.s006.tif]

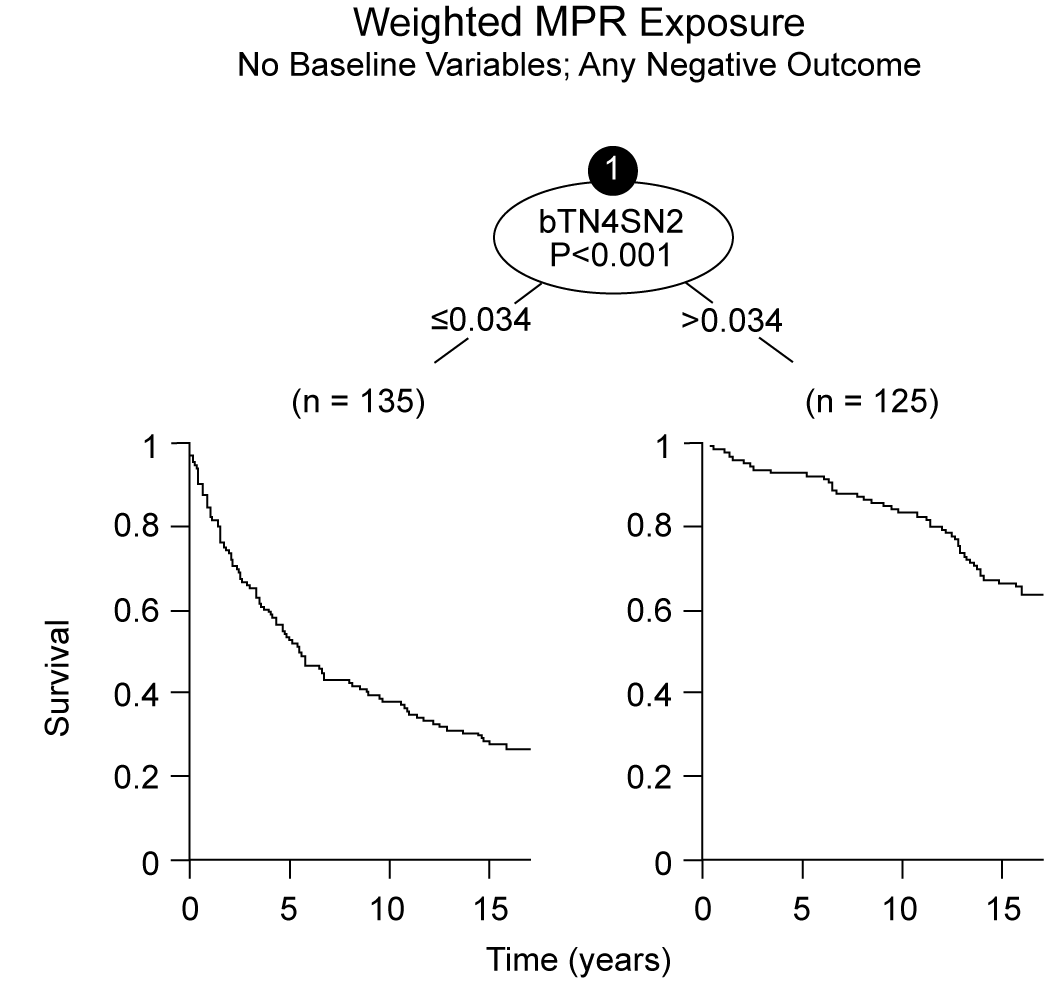

Supplement: Figure S7 — Optimal split determined by the recursive partitioning algorithm considering only weighted-MPR exposure to IFNβ-1b. In this analysis, the RP algorithm was presented with all weighting-schemes (161) and selected the bTN4SN2 weighting-scheme as the one most closely associated with a negative-outcome. This is the one used in this analysis and the survival curves for the optimally split data are shown. The left-hand panel shows survival in the low-exposure group whereas the right-hand panel shows much better survival in the high-exposure group. Note: the number (0.034) cannot be interpreted in time units because it represents a mathematical transformation from the raw exposure in years. Below the splits, the survival curves are plotted. X-axis is time in years. Y-axis is survival in % (1 = 100%). (TIF) [file pone.0022444.s007.tif]

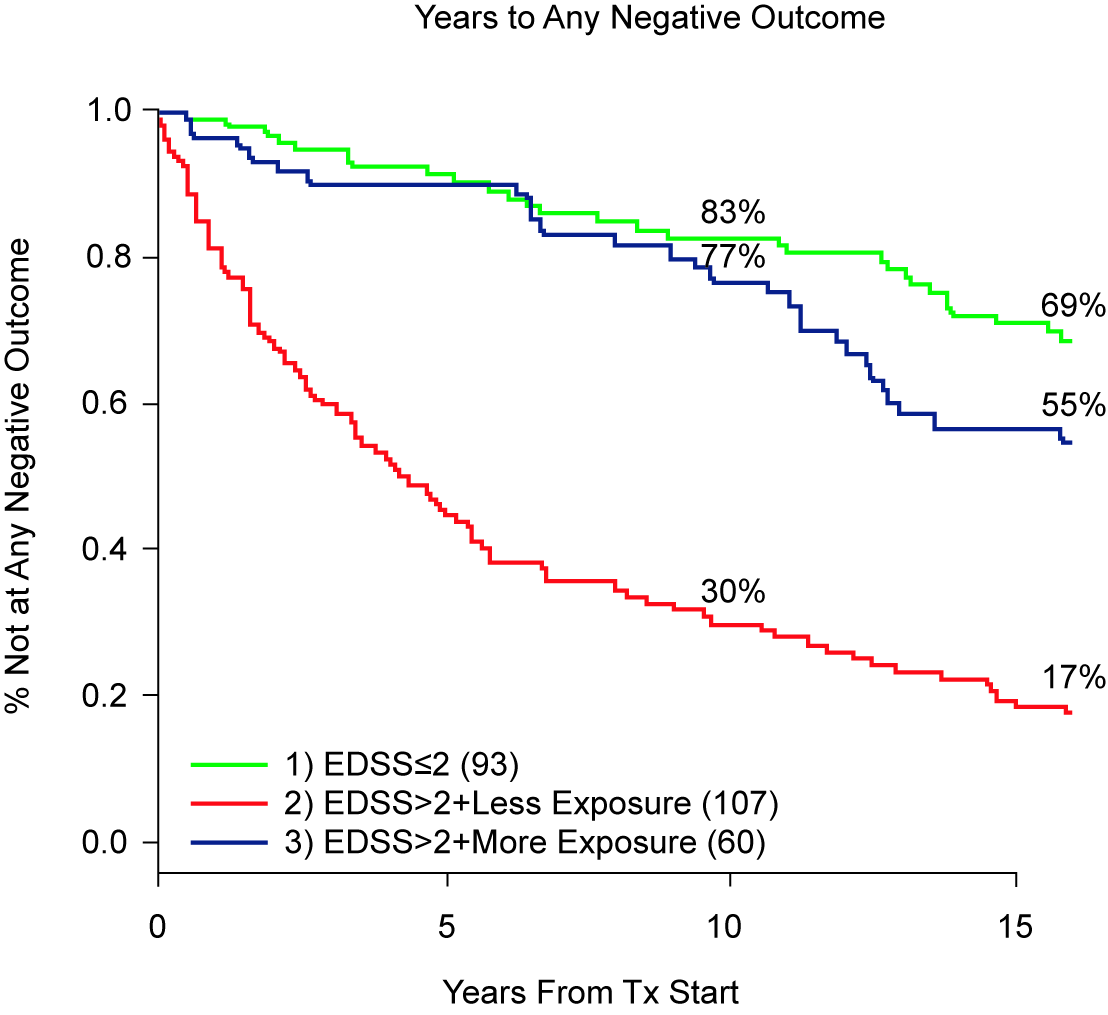

Supplement: Figure S8 — Superimposed survival curves for the same three groups presented previously in Figure 2 (Main paper). As can be appreciated from the Figure, the proportional hazard assumption only holds out to approximately 10 years. After that point, the patients with EDSS>2 and more exposure (blue line) begin to fail at a greater rate than patients with EDSS>2 and less exposure (red line). The only way to satisfy the proportional hazards assumption is to truncate the data at 10 years. Censoring the data at this point, however, actually leads to a more extreme hazard ratio and a more significant difference between the two groups. Such an outcome is anticipated because any violation of the proportionate hazard assumption should be biased toward the null hypothesis. (TIF) [file pone.0022444.s008.tif]

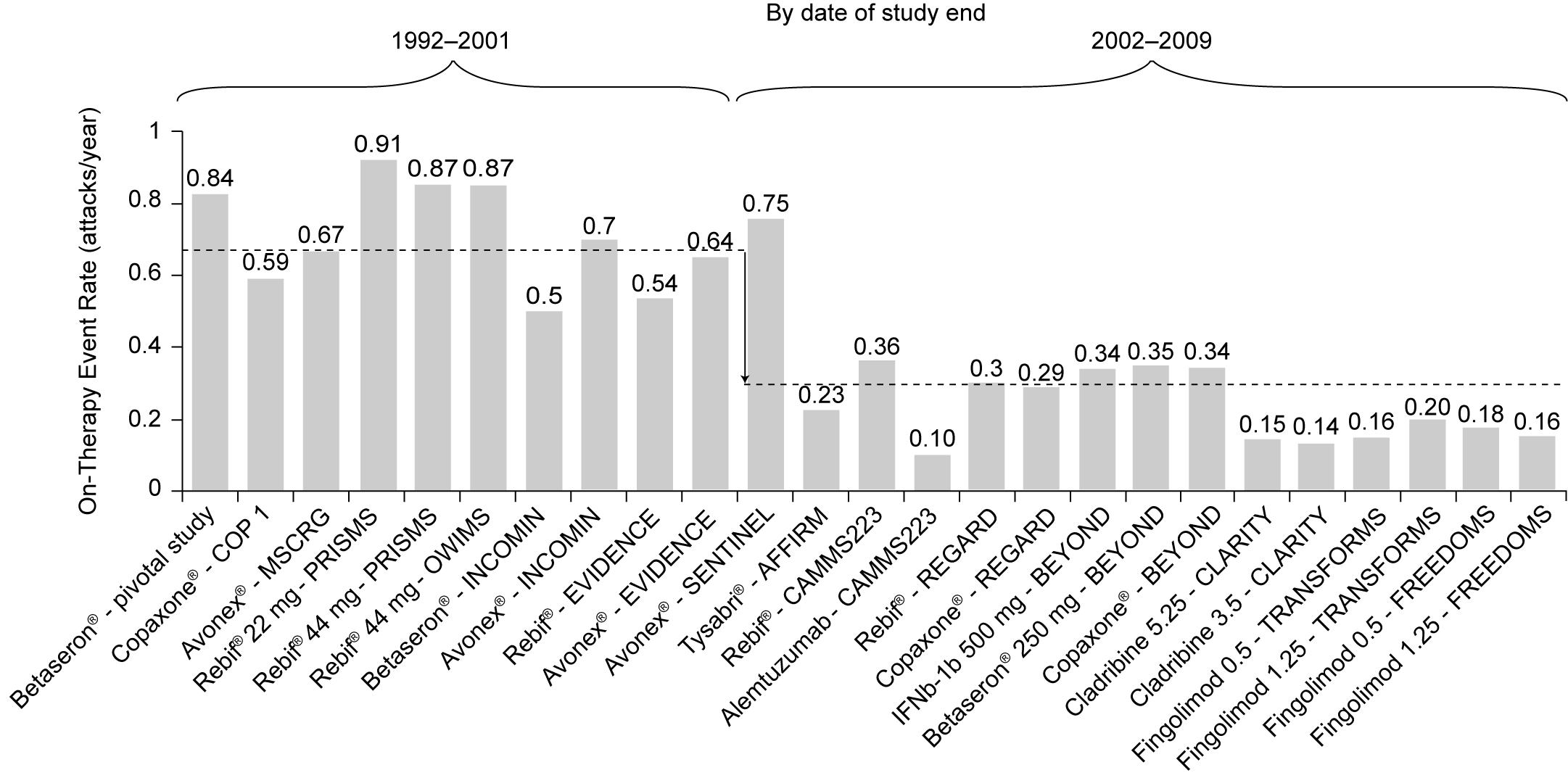

Supplement: Figure S9 — The on-therapy event-rate (annualized attack-rate) in clinical trials since the completion of the original IFNβ-1b (Betaseron®) trial2, 3 in 1992. Other trials include COP-1 (GA, Copaxone®)33; MSCRG (IFNβ-1a, Avonex®)34; PRISMS (IFNβ-1a, Rebif®)35; OWIMS (IFNβ-1a, Rebif®)36; EVIDENCE (Rebif® vs. Avonex®)37; INCOMIN (Betaseron® vs. Avonex®)38; AFFIRM (natalizumab, Tysabri®)39; SENTINEL (Avonex® vs. Avonex® plus Tysabri®)34; CamMS (alemtuzamab, Campath® vs. Rebif®)41; REGARD (Rebif® vs. Copaxone®)9; BEYOND (Betaseron® 500 µg vs. Betaseron® 250 µg vs. Copaxone®)10; CLARITY (Cladribine)43; TRANSFORMS (Fingolimod)44; and FREEDOMS (Fingolomid).45 These event-rates, even for the same study medications (Betaseron®, Rebif®, and Copaxone®), have fallen precipitously in the past 5 years, at least in part, because current trials tend to recruit patients with more mild disease (i.e., more patients with short disease courses and more patients with lower EDSS scores) compared to trials undertaken when no proven DMTs were available. (TIF) [file pone.0022444.s009.tif]
