# Supplementary material for: Establishing Long-Term Efficacy in Chronic Disease: Use of Recursive Partitioning and Propensity Score Adjustment to Estimate Outcome in MS
Source: PLoS One. 2011 Nov 30;6(11):e22444. doi: 10.1371/journal.pone.0022444 (PMC3227563; doi:10.1371/journal.pone.0022444)
Supplement: Table S1 — Sources of Bias and Corrective Strategies. (DOC) [file pone.0022444.s010.doc]

| **Table S1: Sources of Bias and Corrective Strategies** | | |
| --- | --- | --- |
| **Bias** | **Impact** | **Strategy** |
| Ascertainment | Modified therapeutic effect dependent on characteristics of participating patients. | Follow-up must be as complete as possible and independent of patient, disease, and treatment characteristics. Also need to directly compare both baseline and on-RCT characteristics of those patients participating to those not participating in long-term follow-up |
| Informed Censoring | Inflated estimate of therapeutic benefit because patients doing well continue therapy whereas failing patients switch or stop therapy. | MPR transformation: Use percent of total possible time on therapy instead of absolute time to measure exposure. |
| Treatment-selection | Modified therapeutic effect dependent on patient selection characteristics. | Propensity Scoring: Adjust for the propensity (i.e., likelihood) that a particular treatment will be selected based on available patient characteristics. |
| Multiple Testing | Increased risk of Type 1 error from the use of multiple predictor variables and multiple weighting-schemes | Create a single model incorporating all predictor variables and weighting-schemes and apply Bonferroni adjustment to p-values according to the number of predictors tested in the model. |
