# Supplementary material for: Establishing Long-Term Efficacy in Chronic Disease: Use of Recursive Partitioning and Propensity Score Adjustment to Estimate Outcome in MS
Source: PLoS One. 2011 Nov 30;6(11):e22444. doi: 10.1371/journal.pone.0022444 (PMC3227563; doi:10.1371/journal.pone.0022444)
Supplement: Table S2 — Strategy for bias-minimization in the analysis of non-randomized observational data. (DOC) [file pone.0022444.s011.doc]

**Table S2.** Strategy for bias-minimization in the analysis of non-randomized observational data

| **Analysis Steps** |  |
| --- | --- |
| **1. Data Preparation** | **A.** Calculate raw exposures (MPR)  **B.** Apply all weighting-schemes to raw exposures (Bass and exponential –Figure S2) |
| **2. Interim Modeling** | **A.** Select optimal weighting-scheme  (recursive partitioning [RP] using different weightings of raw-MPRs)  **B.** Build treatment groups  (RP using optimally weighted-MPR exposure)  **C.** Initial validation (RP using all predictor variables and different weightings of raw-MPRs)  **D.** Build propensity bins (logistic regression using optimally weighted-MPR) |
| **3. Final Modeling** | **A.** Propensity adjusted survival analysis (Cox proportional hazard model) |
| **4. Check Robustness** | **A.** Explore alternate outcomes, assumptions, and analysis methods |
