# Supplementary material for: Establishing Long-Term Efficacy in Chronic Disease: Use of Recursive Partitioning and Propensity Score Adjustment to Estimate Outcome in MS
Source: PLoS One. 2011 Nov 30;6(11):e22444. doi: 10.1371/journal.pone.0022444 (PMC3227563; doi:10.1371/journal.pone.0022444)
Supplement: Table S3 — Sensitivity analyses using alternative definitions of outcome and exposure. (DOC) [file pone.0022444.s012.doc]

**Table S3.** Sensitivity analyses using alternative definitions of outcome and exposure

| **Negative-outcome**  **(definition)** | **Assumed Exposure**  **(when uncertain)** | **Selected Weighting**  **Scheme*** | **Significance**  **(p-value)** |
| --- | --- | --- | --- |
| “Any” Negative-outcome | Maximum | **bTN4SN2** | 0.00002 |
| “Any” Negative-outcome | Minimum | **bTN4SN2** | 0.00004 |
| “Any” with EDSS Override | Maximum | **bTN4SN2** | 0.00002 |
| “Any” with EDSS Override | Minimum | **bTN4SN2** | 0.00004 |
| EDSS≥6 | Maximum | **bTN4SN2** | 0.0007 |
| EDSS≥6 | Minimum | **bTN4SN2** | 0.001 |
| EDSS≥6 with EDSS Override | Maximum | **bTN4SN2** | 0.00002 |
| EDSS≥6 with EDSS Override | Minimum | **bTN4SN2** | 0.00005 |
| ES (i.e., EDSS≥6 or SPMS) | Maximum | **bTN4SN2** | 0.00003 |
| ES (i.e., EDSS≥6 or SPMS) | Minimum | **bTN4SN2** | 0.00005 |
| ES with EDSS Override | Maximum | **bTN4SN2** | 0.00002 |
| ES with EDSS Override | Minimum | **bTN4SN2** | 0.00005 |
| SPMS | Maximum | **bTN4SN3** | 0.01 |
| SPMS | Minimum | **bTN4SN3** | 0.01 |
| Use of a Wheelchair | Maximum | **bTN4SN2** | 0.00006 |
| Use of a Wheelchair | Minimum | **bTN4SN2** | 0.00008 |

* Weighting-scheme is designated by the curve selected for the time since 1^st^ clinical event (T) and the disease severity (EDSS score) at the start of treatment (S). Thus, if the curve selected for time was bN4 and that selected for severity was bN2, the weighting-scheme would be designated bTN4SN2.
