# Supplementary material for: Establishing Long-Term Efficacy in Chronic Disease: Use of Recursive Partitioning and Propensity Score Adjustment to Estimate Outcome in MS
Source: PLoS One. 2011 Nov 30;6(11):e22444. doi: 10.1371/journal.pone.0022444 (PMC3227563; doi:10.1371/journal.pone.0022444)
Supplement: Protocol S1 — (PDF) [file pone.0022444.s013.pdf]

1. Title page

A long-term follow-up of patients enrolled in the pivotal study of Betaseron® (interferon beta 1b) in relapsing-remitting multiple sclerosis:

# – Protocol TB01-35686 (USA)
# – Protocol TB01-35886 (Canada)

Author: **Christian Wolf** **Date:** **20 August 2004**

Final Proposed Protocol

Version 3.0

for North America

An Observational Pilot Study

Confidential

Property of Berlex

This Study Protocol is produced on a word-processing system and bears no signatures.
The approval of the Study Protocol is documented in a separate Signature Document.

2. Synopsis

Study phase	Observational / no formal phase allocation
Investigational product, dosage, and route of administration	Not applicable
Reference product, dosage, and route of administration	Not applicable
Project code	MP-00102 / Protocol No. 308272
Indication	Multiple sclerosis (MS)
Study objectives	1. To explore and describe the course and the effects of the disease, the treatment history, safety and tolerability and possibly the effectiveness of Betaseron® (interferon-beta 1b; IFNB-1b) in patients having participated in the pivotal study of Betaseron® in relapsing-remitting multiple sclerosis (RRMS).<br>2. To compare today's characteristics of the patients of the pivotal study with those of a population-based natural-history cohort (NH cohort).<br>3. To assess the feasibility of recruiting a cohort of untreated patients with long-standing diagnosis of MS and to explore the suitability of such a cohort to serve as a control group for open long-term follow-up studies
Patient population	Patients who participated in the Betaseron® RRMS pivotal study in North America, i.e. US and Canada (NA cohort)
Study design	Observational. No protocol-induced treatment will be administered.
Concurrent control	None for the primary study objective.<br>Data from a population-based NH cohort will be used to evaluate the present characteristics of the patients from the pivotal study.<br>Approx. 60 untreated MS patients from Britain who fulfilled a basic set of selection criteria during the recruitment period of the pivotal Betaseron® study will be recruited to explore the suitability of such patients and their records to serve as a control group for open long-term follow-up studies (UK cohort).
Duration of treatment	Not applicable.
Duration of assessment	The period of assessment per patient should be limited to 21 days. During that period, it is suggested to perform the protocol-planned investigations during not more than 3 visits.
Methodology	Patients of the NA cohort will be contacted and asked to participate in this long-term follow-up study.<br>Assessments will include:<br>- Clinical evaluations:, neurological examination, disability status, a functional measure, ambulatory status, disease status, and check for injection sites<br>- Magnetic resonance imaging (MRI)<br>- Laboratory tests including neutralizing antibodies to IFNB-1b and immunological evaluations<br>- Neuropsychological testing (modified MACFIMS)

	- Health-related quality of life (HRQoL) assessment<br>- Assessment of resource use<br>- Retrospective data/chart review to assess survival, relapse rates, and side effects.<br>The patients of the UK cohort will be recruited and matched to patients in the NA cohort for age, gender, duration of disease, relapse rate, and disease status. The UK cohort will undergo all assessments listed previously with the exception of the clinical laboratory tests.<br>Further, pharmacogenetic evaluations are planned. The patients' participation in these pharmacogenetic evaluations will be subject to a separate informed consent. It is optional and independent of the participation in the study. A patient's agreement to or refusal of consent for this sampling will have no effect on the remainder of the study procedures.
Number of study centers	NA cohort: 11 centers that participated in the Betaseron® pivotal study (7 in the U.S. and 4 in Canada)<br>UK cohort: up to three centers in Britain
Total number of patients, minimum and maximum number of patients per center, statistical rationale provided	NA cohort: a total of 372 patients participated in Betaseron® pivotal study. Attempts will be made to obtain information about all patients and to examine as many patients as possible.<br>UK cohort: approximately 60 patients / limitations due to feasibility may exist.
Adverse events	Adverse events (AEs) occurring during the study period will be recorded and followed. AEs noted during chart review will be recorded separately.
Plan for data analysis	Descriptive exploration of survival, MS status, functional status, relapse rate, neuropsychological function, quality of life, resource use, MRI parameters, safety, and tolerability by length of exposure to Betaseron® and stratified by the original clinical trial group assignment (low dose, high dose, placebo).<br>Comparison of the characteristics of the NA cohort with those of the UK cohort.<br>Comparison of the characteristics of the NA cohort with those of a NH cohort.<br>Data may be also used to populate an already existing epidemiological MS model to compare treated patients with untreated patients and natural history data.
Planned start and end of recruitment	September 2004 – February 2005
Manufacturer(s) of the investigational / reference product(s)	Not applicable. No protocol-induced treatment will be administered.

3. Table of contents

1. Title page 1

2. Synopsis 2

3. Table of contents 4

4. Investigators and study administrative structure 7

5. Introduction 9

6. Study objectives 9

7. Investigational plan 10
   7.1 Overall study design and plan – description 10
   7.2 Discussion of study design, including the choice of control groups 11
   7.2.1 The London (Ontario) natural-history cohort 11
   7.2.2 The cohort of untreated patients in Britain 12
   7.3 Selection of study population 13
   7.3.1 North American cohort 13
   7.3.2 United Kingdom cohort 13
   7.3.3 Removal of patients from treatment or assessment 15
   7.4 Treatments 16
   7.4.1 Treatments administered 16
   7.4.2 Identity of investigational product(s) 16
   7.4.3 Method of assigning patients to treatment groups 16
   7.4.4 Selection of doses in the study 16
   7.4.5 Selection and timing of dose for each patient 16
   7.4.6 Blinding 16
   7.4.7 Prior and concomitant therapy 17
   7.4.8 Treatment compliance 17
   7.5 Efficacy and safety variables 17
   7.5.1 Procedures for efficacy and safety measurements and flow chart 17
   7.5.2 General guidance 17
   7.5.2.1 Schedule of activities and evaluations 18

7.5.2.2 Patient-reported measures: Mood, quality of life, utilities, and resource use 18
7.5.2.3 Neuropsychological evaluations 19
7.5.2.4 Multiple Sclerosis Functional Composite Measure 21
7.5.2.5 Functional Systems Scores and Expanded Disability Status Scale 21
7.5.2.6 Medical history 23
7.5.2.7 MS history 23
7.5.2.8 Medication history 24
7.5.2.9 Magnetic resonance imaging (MRI) 25
7.5.2.10 Laboratory Tests 25
7.5.2.11 Adverse reactions 26
7.5.3 Appropriateness of measurements 27
7.5.4 Efficacy variables 27
7.5.4.1 Primary efficacy variable(s) 27
7.5.4.2 Efficacy and/or effectiveness related variable(s) 27
7.5.5 Safety variables 28
7.5.5.1 Adverse events 28
7.5.5.1.1 Documentation 28
7.5.5.1.2 Expected adverse events 31
7.5.5.1.3 Unexpected adverse drug reaction 32
7.5.6 Pharmacokinetic and bioanalytical methods 33
7.6 Data quality assurance 33
7.7 Statistical methods and determination of sample size 34
7.7.1 Statistical and analytical plans 34
7.7.1.1 General 34
7.7.1.2 Analysis of efficacy- and effectiveness-related variables 34
7.7.1.3 Analysis of safety variables 34
7.7.2 Determination of sample size 35
7.7.3 Use of data in an epidemiological model 35
7.8 Ethics 36
7.8.1 Independent Ethics Committee and Institutional Review Board 36
7.8.2 Ethical conduct of the study 36
7.8.3 Patient information and consent 36
7.9 Data handling and record keeping 37

7.10 Drug accountability 38
7.11 Financing 38
7.12 Financial disclosure 38
7.13 Publication policy 39

8. Reference list 40

9. Abbreviations 42

10. Attachments 43

List of Text Tables

TT 1: Table of Activities 18

TT 2 Suggested order of administration for the MACFIMS tests 20

TT 3 Laboratory Parameters 26

TT 4 Blood loss 32

TT 5 Enrollment of the pivotal study by study site 35

4. Investigators and study administrative structure

The protocol for this study was written and approved by Berlex Pharmaceuticals, Inc. The administrative structure for the study is shown below. Administrative changes that do not affect the clinical management of patients will not require a protocol amendment.

Sponsor Staff

**Function**	**Name**	**Contact Information**
Sponsor	Berlex Pharmaceuticals, Inc.<br>attn. Dr. Edgar Salazar-Grueso	PO Box 1000<br>Montville, NJ 07045-1000 (USA)
Core Clinician Representative	Dr. Christian Wolf	SAG<br>Corporate Clinical Development CNS<br>13342 Berlin (Germany)
Study Manager	Dr. Nadia Tenenbaum	Berlex Laboratories<br>PO Box 1000<br>Montville, NJ 07045-1000 (USA)
Health Outcomes Research Advisor	Dr. Peter Kaskel	SAG<br>Corporate Outcomes Research<br>13342 Berlin (Germany)
MRI Advisor and Deputy of Core Clinician Representative	Klaus Wagner	SAG<br>Corporate Clinical Development CNS<br>13342 Berlin (Germany)
Biometry Liaison	Dr. Francis Boateng	Berlex Laboratories<br>PO Box 1000<br>Montville, NJ 07045-1000 (USA)
Data Management Liaison	Seth Farber	Berlex Laboratories<br>PO Box 1000<br>Montville, NJ 07045-1000 (USA)
Berlex Laboratories Liaison	Douglas Stefanelli	Berlex Laboratories<br>PO Box 1000<br>Montville, NJ 07045-1000 (USA)
Berlex Canada Liaison	Christine Boudreau	Berlex Canada Inc.<br>334, rue Avro<br>Pointe-Claire, Québec H9R 5W5 (Canada)
SHCL UK Liaison	Dr. Jacqueline Napier	SHCL<br>The Brow<br>Burgess Hill, West Sussex, RH15 9NE (UK)

Investigators

Function	Name	Contact Information
Principle and Signatory Investigator	Dr. George Ebers	University of Oxford<br>Department of Clinical Neurology<br>The Radcliffe Infirmary<br>Woodstock Road<br>Oxford OX2 6HE (UK)

Names and functions of all other principal investigators, sub-investigators, study nurses, study coordinators, psychologists, and other study-related personnel at the study centers are recorded in a current list filed in the Trial Master File (TMF) at Berlex or its designee.

Experts and Central Evaluation Units

Function	Name	Contact Information
Consultant and auditor for the neuropsychological assessment	Dr. Dawn W. Langdon	University of London - Royal Holloway<br>Dept of Psychology<br>Egham, Surrey TW20 0EX (UK)
Consultant for the neuropsychological assessment	Dr. Jennifer Gurd	University Department of Clinical Neurology<br>The Radcliffe Infirmary<br>Woodstock Road<br>Oxford OX2 6HE (UK)
Central MRI analysis facility	UBC MS/MRI Group<br>Dr. David Li<br>Dr. Anthony Traboulsee	University of British Columbia<br>2211 Wesbrook Mall<br>Vancouver V6T 2B5 (Canada)
Central laboratory facility	ACM Medical Laboratory	160 Elmgrove Park<br>Rochester NY 14624 (USA)

Operational Services

Function	Name	Contact Information
Operational Liaison<br>(PPD Project Management)	Marla Bowman	PPD Development, Inc.<br>3151 17th Street Extension<br>Wilmington, NC 28412 (USA)
Study Biometrician	Ann Buckley, MPH	PPD Development, Inc.<br>3151 17th Street Extension<br>Wilmington, NC 28412 (USA)
Monitor<br>(Clinical Research Associate)	Ann Hart	PPD Development, Inc.<br>3151 17th Street Extension<br>Wilmington, NC 28412 (USA)

5. Introduction

The Betaseron® pivotal protocols TB01-35686 in the United States (US) and TB01-35886 in Canada were conducted as one single Phase III, multicenter, double-blind, placebo-controlled, parallel-group study to evaluate Betaseron® treatment in relapse-remitting multiple sclerosis (RRMS). This protocol will refer to both parts as the "pivotal study".

Betaseron® (interferon beta-1b; IFNB-1b) was the first recombinant IFNB preparation approved for treatment of RRMS. Its first approval was based on the afore mentioned pivotal study. Efficacy was demonstrated in terms of reducing the frequency and severity of relapses and/or lesion number and load as per magnetic resonance imaging (MRI).

The side effect profile of parenterally administered IFNB is now well established, and IFNB preparations are being regarded as safe and well tolerated in the treatment of MS patients.

The mechanisms by which IFNB exerts its beneficial effect in MS are not well understood, but are most likely of immunomodulatory nature.

Enrollment for the pivotal study began in June 1988 and ended in July 1989. A total of 372 patients were enrolled in the U.S. and Canada. Patients were randomly assigned to receive 104 weeks of placebo, 1.6 MIU (50 µg) Betaseron®, or 8 MIU (250 µg) Betaseron® injected subcutaneously every other day. That study demonstrated that 8 MIU Betaseron® was both effective and well tolerated, reducing both the overall exacerbation rate and the rate of moderate and severe exacerbations compared to placebo. Patients completing the study were offered the option to continue double-blind treatment and evaluations for up to five years. After approval of Betaseron® for marketed use at 8 MIU, all remaining patients were switched to commercially available Betaseron® at 8 MIU.

The results of the pivotal studies were publicized by The IFNB Multiple Sclerosis Study Group in 1993. Another publication by Paty et al. 1993 describes the MRI methodology and the imaging-based results.

6. Study objectives

The primary objective of this study is to systematically describe the long-term course of the disease and possibly the long-term effectiveness of Betaseron® by evaluating the patients having participated in the pivotal study of Betaseron® in RRMS as a complete group and stratified by the original clinical trial group assignment (1.6 MIU, 8 MIU, and placebo).

Further objectives are a

- comparison of the course of the disease of the pivotal study patients with that of a natural-history cohort (NH cohort; first described by Weinshenker et al. 1989) and
- a second comparison with the course of the disease in a cohort of untreated patients chosen according to a set of major selection criteria of the original pivotal protocol.

To achieve the latter objective, it will be necessary to recruit a cohort of untreated patients with a long-standing diagnosis of MS. This may also allow the exploration of the suitability of such a cohort to serve as a control group for open long-term follow-up studies.

This study offers opportunities to generate hypotheses. Should – in the opinion of this study group – there be hypotheses generated which would be worthwhile to be followed, all patients who complete this study will be asked to continue a prospective follow-up after completion of this study. Details of subsequent evaluations will be determined based on findings from the pilot evaluations to be used in this study and will be the subject of a different research protocol or an amendment to this protocol.

7. Investigational plan

7.1 Overall study design and plan – description

- Multi-center, open, observational study in MS patients without study-induced treatment intervention.
- Patients who participated in the Betaseron® pivotal study will be contacted and asked to participate in this long-term follow-up study. They will form the North American cohort (NA cohort).
- Approx. 60 untreated RRMS patients will be recruited in Britain. They will form the UK cohort and will be matched to patients in the NA cohort for age, gender, duration of disease, relapse rate, level of disability, and disease status.
- The recruitment period is scheduled from September 2004 to February 2005.
- It is suggested to limit the individual study period to 21 days.
- It is suggested to limit the patient's visits to the study site to up to three regular visits. Please, note: investigations may be performed during 21 consecutive days beginning the date of the informed consent, i.e., also during the day of the informed consent. In that case, the study may consist of one visit for the patient.
- A table of activities is provided in TT 1 (page 18).
- The analysis of the results will comprise of:

  A descriptive exploration of survival, MS status, functional status, relapse rate, cognitive function, quality of life, resource use, imaging and laboratory parameters, safety and tolerability by length of exposure to Betaseron® and stratified by the original clinical trial group assignment (1.6 MIU, 8MIU, placebo).

  A comparison of the characteristics of the NA cohort with those of a NH cohort and the UK cohort.

  Data may be also used to populate an already existing epidemiological MS model to compare treated patients with untreated patients and natural history data.

This study will be conducted in compliance with the protocol, ICH-GCP and any applicable regulatory requirements.

7.2 Discussion of study design, including the choice of control groups

This is an observational study of patients who participated in the Betaseron® pivotal study (NA cohort). The characteristics of patients will be described systematically considering intention-to-treat and time-exposure analyses.

Furthermore, it is intended to perform comparison with partially validated natural history data (cf. Section 7.2.1 below) and an additional cohort of untreated patients. The cohort of untreated patients will be recruited in Britain (UK cohort) where disease-modifying therapies are not readily prescribed, thereby serving as a potential source for an untreated control population.

7.2.1 The London (Ontario) natural-history cohort

The London (Ontario) natural-history control group, i.e., the NH cohort, has been subject of many publications. It represents a population-based group of MS patients receiving virtually no treatment aimed at altering the natural course of the disease. The specific details of this cohort have been described in considerable detail (Weinshenker et al. 1989, 1989, 1991, 1991; Cottrell et al. 1999, 1999; Kremenchutzky et al. 1999). The database represents 25,000 patient years of observation and it is by far the largest such cohort in existence. Opportunities for developing additional natural-history data must be considered to have ceased. The widespread usage of a variety of treatments including the IFN under study in this protocol preclude the availability of more data of this type.

The London (Ontario) natural-history data has been used by several authorities in generating cost-effectiveness models. These data formed the basis for the study by Kobelt et al. from the Stockholm School of Economics (Kobelt et al. 2002). It also formed the basis for the economic model developed by the School of Health and Related Research at Sheffield (UK; ScHARR) (Tappenden et al. 2001) and at present represents the database to be used in the British Department of Health Risk Sharing Scheme (2002).

The reasons for the selection of this database for these analyses relate to its large size, nearly complete follow-up, nearly complete diagnostic certainty and the presence of detailed information on time to all the levels of the disability scale. A partial validation of this database was mentioned. It appears that at least for time-to-cane requirement essentially no difference can be found when comparing this dataset to results from Gøteborg (Sweden) and Tucson, Arizona (USA). More detailed comparisons are impossible since the systematic follow up employed in the London (Ontario) cohort has not been carried out in other sites over the long term. Nevertheless, despite uncertainties of ascertainment and the use of azathioprine in some 50% of the patients, the London (Ontario) results are still closely paralleled by those from Lyon (Confavreux et al. 2000).

Further validation of this cohort will increase the certainty of outcome predictions and comparisons and measurement of outcome parameters in other cohorts, both systematically collected and randomly generated will extend the validation process and such studies are in process in Oxford (UK).

The NH cohort – despite antedating some of the modern diagnostic tests such as MRI at least at the time of diagnosis – enjoys considerable diagnostic reliability. Since the mean follow up is 25 years, patients have had the opportunity to evolve in a way in which other diagnoses have occasionally come to light and when this has occurred, such patients are excluded from the database. This perhaps cannot be said to be true of all the modern cohorts. Indeed, among patients entered into clinical trials, the occasional emergence of other diagnoses happens at a rate not dissimilar to the diagnostic error confirmed at 25 years in the NH cohort.

7.2.2 The cohort of untreated patients in Britain

It is acknowledged that more than 15 years have passed since the original recruitment period (1988/1989). The extent, quality, and availability of the patients' documentation may hamper the recruitment of the targeted 60 untreated patients of the UK cohort. Therefore, it is also an aim of this study to explore the feasibility of recruiting a cohort of untreated patients with a long-standing diagnosis of MS and to explore the suitability of such a cohort to serve as a control group for open long-term follow-up studies.

Feasibility will be checked frequently between the Sponsor and the investigators.

Furthermore, it is acknowledged that the recruitment of the UK cohort could be affected by bias when compared to the selection of the patients of the original pivotal study due to but not limited to the following facts:

- different medico-social circumstances in North America and Britain, e.g. access to a neurologist, with impact on the following variables:
    - diagnosis of MS / perceived duration of disease
    - diagnosis of relapses (perception of symptomatology by patient and/or medical professional)
    - completeness of documentation of relapses and other disease characteristics
- impact of potential treatment 'triage' in favor of patients with a very aggressive course of disease (e.g., treatment with azathioprine, mitoxantrone, or other 'more accessible' agents may lead to exclusion of the patients with a more severe course of the disease)
- matching of patients with conversion to secondary progressive multiple sclerosis (SPMS)
- matching of patients with a fatal outcome

It should be noted that the bias discussed here may result in recruitment of a cohort with a more benign course of the disease. Furthermore, a suspected lower rate of documented relapses and other disease characteristics may favor the perception of the course of disease in the UK cohort.

However, it was felt the MRI investigations and the neuropsychological testing will provide data that will be affected to a lesser degree by bias.

7.3 Selection of study population

7.3.1 North American cohort

To be eligible for enrollment, patients of the NA cohort have to meet the following criteria:

- Previously enrolled in the Betaseron® pivotal study
- Signed and dated informed consent

There are no exclusion criteria for the study in general.

In case of partial inability of the patient to complete all study procedures, e.g. a MRI can not be done due to hypersensitivity to gadolinium-DTPA or only limited cognitive testing possible due to severe cognitive and/or visual impairment, please refer to the respective sections (Section 7.5.2.9, page 25 for MRI and Section 7.5.2.2, page 18, for cognition).

7.3.2 United Kingdom cohort

It is intended that the patients of the UK cohort will resemble the patients recruited for the pivotal study as closely as possible. The original protocol had the following set of selection criteria:

<u>Inclusion criteria of the pivotal study:</u>

1. Patients must have clinically definite MS or laboratory-supported definite MS as defined by Poser criteria for not less than 1 year.
2. Patients must have a history of clearly identified relapses and remissions.
   Relapse shall be defined as the appearance of a new neurologic abnormality or the reappearance of a neurologic abnormality at any time after the initial attack. This change in clinical state shall not be considered a relapse if it does not last at least 24 hours, and if it is not immediately preceded by a stable or improving neurologic state in the 30 days before deterioration.
   A remission is defined as the complete disappearance or significant decrease in severity followed by stability for at least 1 month of a neurologic abnormality that had lasted for at least 24 hours.
3. Patients will have had at least two relapses in the 2 years before entry into the study.
4. Patients will have stable disease for at least one month at time of the screen and baseline evaluations.
5. Patients will be between 18 and 50 years of age inclusive, and be considered a legal adult in the state or province in which the study is conducted.
6. Patients must show objective neurologic evidence of disease that reflects predominantly white matter (fiber-tract) damage. Symptoms alone cannot be accepted as diagnostic.
7. The patient's signs and symptoms cannot be better explained by another disease process.
8. Patients must have a friend or family member who can assist in carrying out the requirements of this protocol should progressive disability impair ability to comply.

9. Patient must be ambulatory, grade as 0 through 5.5 defined in the Kurtzke Disabi1ity Status Scale.
10. Patients must have signed an approved informed consent prior to initiating screening procedures.

<u>Exclusion criteria of the pivotal study:</u>

1. Pregnancy or lactation.
   Women no longer capable of childbearing (i.e., post-hysterectomy, tuba1 ligation or 1 year post-menopausal) are eligible for study enrollment. Women of childbearing potential must be using and agree to continue to use an IUD, birth control pills, or adequate barrier contraception.
2. Medical or psychiatric conditions that compromise the patient's ability to give informed consent or complete the study.
3. New York Heart Classification III or IV.
4. Uncontrolled angina pectoris.
5. Evidence of clinically significant, uncontrolled cardiac dysrhythmias.
6. Prior therapy with alpha, beta or gamma interferon.
7. Need for concomitant therapy with corticosteroids or ACTH or therapy with these agents within 30 days prior to entry into the study.
8. Previous immunosuppressive therapy with cytotoxic chemotherapy.
9. Observation by the principal investigators or their staff at participating institutions cannot be assured for the duration of the study.
10. Upper extremity disability that prevents self-administration of subcutaneous medication, and the absence of a friend or family member who can reliably administer the subcutaneous injections.
11. Patients who are in relapse or who have entered a progressive phase of their illness at time of entry into the study.
12. Allergy to acetaminophen.
13. Need for chronic concurrent therapy with aspirin or non-steroidal anti-inflamatory drugs.
14. Use of other investigational drugs in the 30 days preceding study entry.

For feasibility reasons, the following selection criteria were chosen for enrollment of the untreated patients of the UK

<u>Inclusion criteria:</u>

- In the calendar years 1988-1989:
  - clinically definite MS or laboratory-supported definite MS (Poser criteria) for not less than one year at date of qualification in 1988/89
  - a history of clearly defined relapses and remissions, with at least two relapses in the 2 years preceding the date of qualification in 1988/89
  - 18 to 50 years of age at the date of qualification in 1988/89
  - EDSS score below 6.0 / ambulatory at the date of qualification in 1988/89
    Please, note: if an EDSS score below 6.0 at the date of qualification in 1988/1989 is not documented in the patient chart, the investigators should consider that all EDSS scores below 6.0 require the patient being ambulatory (e.g., EDSS 5.5: ambulatory without aid or rest > 100 m). Use of a cane or crutches at the date of qualification i, 1988/1989 – except for comfort – indicate an EDSS score of 6.0 or higher. A fall

secondary to MS or an assistance device in use to leave the house may be as well indicative for an EDSS score of 6.0 or higher at the date of qualification in 1988/1989.

- Today:
    - MS that has been stable for at least 30 days
    - Signed and dated informed consent

<u>Exclusion criteria:</u>

- Primary progressive multiple sclerosis (PPMS) at any time
- Any previous treatment with immunomodulatory agents including any interferon or glatiramer acetate, bone marrow transplantation, or immunosuppressive agents (corticosteroids are not concerned)
- Any previous treatment with putative experimental MS treatments – only in case of a plausible mechanism of action for the putative intervention (questionable prior interventions should be discussed with the Sponsor's Core Clinician)
- known hypersensitivity to gadolinium-DTPA
- The symptoms / signs indicative for MS may be better explained or confounded by another disease process (e.g., neoplastic processes)

More detailed guidance for the selection of the UK cohort may be provided in a separate Working Procedure.

7.3.3 Removal of patients from treatment or assessment

Removal of patients from treatment is not applicable as no study-induced treatment will be administered (cf. Section 7.4, page 16).

The following medical conditions will lead to a removal from assessments for patients of the UK cohort:

- acute severe hypersensitivity reaction against gadolinium-DTPA
- patient in need for acute treatment with glucocorticoids
- conditions violating the selection criteria (cf. Section 7.3.2, page 13)

In such cases, the patient has to be withdrawn from assessment and should be replaced. The withdrawal of such patients will be documented and reasons for the withdrawal of the patient will be provided.

Patients in the NA cohort will not be removed from assessment. Should acute medical conditions prevent an immediate assessment of the NA patients, the assessment should be postponed until the protocol-stipulated investigations can be performed.

Every patient or his/her legal representative or proxy consenter has the right to refuse further participation in the study at any time and without providing reasons (see also section 7.8.3). A patient's participation is to terminate immediately upon his / her request. The investigator should seek to obtain the reason and record this on the case report form (CRF) and the source documentation (e.g., the patient's hospital file).

The patient may be withdrawn from the study at any time at the discretion of the investigator; the reason should be fully documented on the CRF and the source documentation (e.g., the patient's hospital file).

At the discretion of the Sponsor's Core Clinician, the entire study may be canceled for medical reasons. In addition, the Sponsor retains the right to end the study at any time if the study cannot be carried out as agreed upon in the protocol.

In case of premature termination or suspension of the study, the Sponsor will inform the investigator / institutions, regulatory authorities and IEC / IRBs of the termination or suspension and the reason for it.

7.4 Treatments

7.4.1 Treatments administered

Not applicable. Study drug is not being provided.

7.4.2 Identity of investigational product(s)

Not applicable. Study drug is not being provided.

7.4.3 Method of assigning patients to treatment groups

Not applicable. Study drug is not being provided.

7.4.4 Selection of doses in the study

Not applicable. Study drug is not being provided.

7.4.5 Selection and timing of dose for each patient

Not applicable. Study drug is not being provided.

7.4.6 Blinding

Not applicable. Study drug is not being provided.

7.4.7 Prior and concomitant therapy

NA cohort:
No restrictions. All MS-specific prior and concomitant medication since leaving the pivotal study has to be documented in the CRF (cf. Section 7.5.2.8, page 24).

UK cohort:
The restrictions as delineated in Section 7.3.2 (page 13) apply also during the study period. Furthermore, treatment with glucocorticoids is not allowed. Patients in need of glucocorticoid treatment before completion of all protocol-mandated procedures have to be excluded from investigation and will be replaced (cf. Section 7.3.3, page 15).

7.4.8 Treatment compliance

Not applicable. Study drug is not being provided. Attempts will be made to get an impression about the compliance of the patients of the NA cohort since the termination of the pivotal study.

7.5 Efficacy and safety variables

It is suggested to perform all tests and evaluations on any one patient within a 21-day period. The patient should be neurologically stable for at least 30 days prior to the start of this testing period. All tests can be performed on one day if desired by the study center and if the patient can tolerate it. Alternatively, tests can be spread during the entire study period after obtaining the informed consent. The actual study period is the period between the ascertainment of the informed consent and the last study procedure.

7.5.1 Procedures for efficacy and safety measurements and flow chart

7.5.2 General guidance

For retrospective measures, the actual information will be collected along with information on the source of documented data (i.e., clinic records, primary doctor records, outside records, or hospital records) and source of verbal reports (i.e., patient, spouse, other family, other acquaintance, or other health care professional).

7.5.2.1 Schedule of activities and evaluations

A schedule of activities to be completed by the NA cohort and the UK cohort is provided below. The tabulation reflects also the suggested order in which the procedures should be done. Individual or organizational circumstances may justify deviations from the proposed order.

TT 1: Table of Activities

Activity[b]	NA cohort	UK cohort
Informed consent	X	X
Patient-reported measures: EQ-5D[f], FAMS[f], HADS[f], MS-SHCS[f]	X	X
A 30 min break is suggested before proceeding with the neuropsychological testing.		
Neuropsychological testing[e]	X[e]	X[e]
MSFC	X	X
A short break is suggested before proceeding with the other investigations.		
Neurological examination and EDSS[a]	X[d]	X[d]
Medical history	X	X
MS history	X	X
Medication history	X	X
Blood sampling: clinical laboratory (cf. TT 3)	X	
Blood sampling: immunology	X	X
Blood sampling: pharmacogenetics	optional[g]	optional[g]
MRI	X	X
Retrospective data review	X	X

a If not neurologically stable, postpone visit for minimum of 30 days.
b Visit activities may be completed on separate days within a suggested 21-day period after signing of the informed consent.
c Informed consent must be signed before any study procedures are performed.
d EDSS score determined prior to evaluator's knowledge of previous EDSS score and history.
e Neuropsychological testing should take approximately 55 min.
f Self-assessment scale; will be included in the patients' booklet of the CRF.
g Subject to a separate informed consent and not mandatory for participation in the study.

7.5.2.2 Patient-reported measures: Mood, quality of life, utilities, and resource use

All patient-reported measures – mood, quality of life, resource use – will be self-administered using validated questionnaires. The questionnaires should be the first examination of the day. There should be a 30 minute break for rest after completion and before other examinations. The following questionnaires will be administered in the order listed below:

- the EuroQuol 5-dimensional questionnaire (EQ-5D; EuroQol Group 1990), and
- the Functional Assessment of Multiple Sclerosis (FAMS; Cella et al. 1996),

- Hospital Anxiety and Depression Scale (HADS; Zigmond and Snaith 1983)
- Multiple Sclerosis Standard Health Care Survey for resource use (MS-SHCS; Kobelt et al. 2001/2004; Hendriksson et al. 2001).

Trained study professionals should ensure that patients have sufficient time and privacy to complete the questionnaire. After questionnaires are explained to the patient, he/she will be asked to fill out the questionnaires as completely and accurately as possible. Assistance will be provided to patients who have difficulty with reading. Study professionals should check immediately afterwards that only one box has been ticked for each question and that every question has been answered.

7.5.2.3 Neuropsychological evaluations

It is suggested to perform the neuropsychological evaluations on all patients within 21 days after signing the informed consent.

If all study investigations will be performed during one day, the neuropsychological assessment should be done after a 30 minute break for rest after completion of patients' questionnaires and before all other examinations. Should the study investigations be performed during more than one day, the neuropsychological assessment should be performed during the second day as the first examination of the day.

The battery of tests to be used in this study is based on the "Minimal Assessment of Cognitive Function in MS (MACFIMS)" proposed by a panel of neuropsychologists and psychologists (Benedict et al 2002). The battery has been modified for the purpose of this study with regard to the following:

- The NART will be replaced by the WTAR. The WTAR provides the same stimuli for US and UK populations and has a set of norm data for each of theses countries.
- The PASAT (3-second version; PASAT-3") will be performed as a part of the MSFC (cf. Section 7.5.2.4, page 21).

The assessments of this modified battery will evaluate the cognitive domains "processing speed/working memory", "learning and memory", "executive function", "sustained attention", and "language" in the context of potential confounds to test interpretation (premorbid cognitive ability, mood, and visual acuity).

The neuropsychological tests include the following scales and tasks (all referenced in Benedict et al. 2002):

- Visual Acuity
  Rosenbaum Pocket Vision Screener
- Pre-morbid cognitive ability
  WTAR (Wechsler Test of Adult Reading Ability, Psychological Corporation, 2001)
- Verbal fluency
  COWAT (Controlled Oral Word Association Test, Benton and Hamsher, 1989)

- Memory
  CVLT-II (California Verbal Learning Test-II, Delis et al. 2000) / first five recall trials only
- Selective Attention
  SDMT (Symbol Digit Modality Task, Rao et al. 1991)
- Executive function
  D-KEFS (Delis-Kaplan Executive Function System, Delis et al. 2001)
  Free sorting condition only
- Sustained attention
  PASAT-3" (Paced Auditory Serial Addition Task, Gronwall, 1977)*

It is anticipated that the neuropsychological testing will not exceed 60 minutes per patient. The suggested order of administration of the tests is shown in the table below:

TT 2 Suggested order of administration for the MACFIMS tests

**Measure**	**Administration Time [min]**
Rosenbaum Pocket Vision Screener	5
COWAT	5
SDMT (oral administration)	5
CVLT-II (learning trials, recall and recognition)	10
D-KEFS Sorting Test (free sorting condition)	25
WTAR (reading subtest)	5
(PASAT-3")*	(5)
**Total**	**55**

A proportion of patients may not have English as a first language, among them patients in Canada with French as a first language. A comparable neuropsychological test battery for patients with French as a first language will be provided in a separate document. Patients with neither English or French as a first language will be invited to take the tests in the provided English versions. Participation will be strictly voluntary. The patient information will point out the voluntary participation. The test results of patients with a first language other than English will be evaluated separately.

Patients with severe cognitive impairment provide special problems. Although there would be reason to exclude them since they may not be able to carry out some tests requiring a higher degree of cognitive function, they represent an extreme of outcome that would contribute to the validation of efficacy. It is obvious that tests can not be scored which the patients are not able to do and it is recognized that the cognitive impairment shown by a few patients will nevertheless be counted in the Kurtzke functional scale and in the Expanded Disability Status Scale (EDSS). However there may still be useful data obtained from the subtests which can be scored. Inability to carry out tests would be thought by some to constitute a zero score, therefore, it is planned to

* The PASAT-3" will be performed within the context of the MSFC (cf. Section 7.5.2.4, page 21).

analyze the data in a number of ways to encompass the breadth of opinion as this is an exploratory study. This flexibility may aid in the generation of hypotheses.

Investigators should clearly indicate in the CRF such patients that

- in the judgement of the examiner have severe cognitive impairment that makes neuropsychological assessment inappropriate, and/or
- have a visual impairment that makes tests with visual stimuli inappropriate. The criterion will be the Rosenbaum visual screener, 20/50-70 threshold at 14" from corrected eye.

7.5.2.4 Multiple Sclerosis Functional Composite Measure

The Multiple Sclerosis Functional Composite Measure (MSFC) is a three-part, standardized, quantitative assessment instrument for use in clinical trials of MS (Cutter et al. 1999). The three components of the MSFC measure:

- leg function/ambulation (timed 25-foot walk; T25-FW),
- arm/hand function (9-hole peg test; 9-HPT), and
- neuropsychological function (PASAT-3").

Administration time will vary depending upon the ability of the patient. Total administration time for all three measures should be approximately 20-30 minutes. The MSFC measures are administered in person by a trained examiner. The examiner need not be a physician or nurse.

The MSFC can produce scores for each of the three individual measures as well as a composite score. In addition, there are a variety of ways to calculate scores depending on the nature of the study and sample. All three measures have been shown to have good inter-rater and test-retest reliability. In addition, there is considerable evidence for their validity and sensitivity to clinically relevant change in MS patients.

7.5.2.5 Functional Systems Scores and Expanded Disability Status Scale

The Functional Systems Scores (FSS) and Expanded Disability Status Scale (EDSS) are probably the most widely utilized assessment instruments in MS (Kurtzke 1983). Based on a standard neurological examination, optic, brain stem/cranial nerves, pyramidal, cerebellar, sensory, vegetative, and cerebral functions (the seven "functional systems") are rated. These ratings are then used in conjunction with observations and information concerning gait and use of assistive devices to rate the EDSS. Each of the FSS is an ordinal clinical rating scale ranging from 0 to 5 or 6. The EDSS is an ordinal clinical rating scale ranging from 0 (normal neurologic examination) to 10 (death due to MS) in half-point increments.

Administration time will vary depending upon the condition of the patient and the skill of the examiner. Although the FSS and EDSS themselves can be rated in a few minutes, the neurological examination that is needed to make the ratings can take 15-30 minutes.

The FSS and EDSS will be administered in person by a trained examiner, preferably a neurologist. However, nurse practitioners with the proper training can also complete the neurological examination and rate the FSS and EDSS.

The FSS and EDSS were developed in the 1950's and refined in the 1980's to provide a standardized measure of global neurological impairment in MS. Both test-retest reliability and inter-rater agreement have varied considerably from study to study with some studies finding high values and other studies rather low figures. Therefore, the EDSS represents a widely used albeit imperfect standard. The EDSS was also used during the Betaseron® pivotal study. It should be noted that an earlier predecessor version of the EDSS, the so-called DSS was used in the NH cohort.

EDSS scores 1.0 to 4.5 describe people with MS who are fully ambulatory; EDSS scores 5.0 to 9.5 are defined by the extent of impairment of ambulation. Times to reach certain EDSS scores are considered endpoints in this trial. Investigators must use their discretion to determine whether the following conditions apply:

<u>EDSS Score of 3.0</u>
An EDSS score of 3.0 describes patients who are ambulatory without the aid of a device, but their disability interferes with their daily lives. If an EDSS score of 3.0 is not documented in the patient chart, investigators must make a determination using the following guidelines:

- When did patients notice that a full day did not pass without experiencing difficulty?
- When did patients have to decrease their hours at work to less than full-time?
- When did others notice a decrease in the patient's function?

The final determination will be made at the discretion of the investigators.

<u>EDSS Score of 6.0</u>
An EDSS score of 6.0 describes patients who *require* some assistance with ambulation. If an EDSS score of 6.0 is not documented in the patient chart, investigators must make a determination using the following guidelines:

- When did patients begin using an assistance device (e.g., cane or walker)?
  (Note: The investigator should attempt to distinguish between the use of an assistance device for comfort and the use of an assistance device out of necessity.)
- When did patients first fall secondary to MS?
- When did patients first require an assistance device in order to leave their house?

The final determination will be made at the discretion of the investigators.

<u>Conventions for consistency of EDSS scoring:</u>
Since comparisons of outcomes in this study will be made to natural-history data it is important that EDSS, scored at long-term evaluations, be made to conform with a few of the conventions which were used in the natural history data. The first of these is that EDSS scores were felt to be confirmed if the patient had the same EDSS or worse one year later. If this confirmation was

found, the first appearance of the EDSS score was backdated to the original observation the previous year. Accordingly, this will mean that the collection of EDSS scores in the protocol will be made more reliable if it is possible to get scores from the previous two years. It is recognized that this will not be possible in all cases although a combination of historical record from the patient themselves, confirmed by their spouse or caretaker, may be necessary in some circumstances to add support to the neurologist's best opinion of where the patient was a year previously. To illustrate this, a patient seen at EDSS 3 who is 4 a year later but two years later had reverted to 3 would not be considered to have reached a confirmed 4. This convention proved to be operationally quite reliable since EDSS scores confirmed at a year virtually never reverted (<2% of cases).

7.5.2.6 Medical history

The treating physician will ask about relevant diseases of the following organ systems: skin, eyes, ears, nose, and throat, head and neck (including the thyroid), lungs, heart, breasts, abdomen, lymph nodes, musculoskeletal system (including extremities and spine), genito-urinary system, gynecological organs, and rectum. History of allergies, in particular with respect to proteins, interferon, or gadolinium-DTPA will be queried, as well as other abnormalities (if indicated). Furthermore, patients will also be asked if they have a history of alcohol or drug abuse, or a history of psychiatric disorder, with particular regard to known depression or suicidal tendencies, head injuries, or epilepsy. Any previous disease or surgeries will also be documented. The oral history obtained from the patient or a caretaker should be supplemented by a thorough chart review and/or by an interview of the medical doctor currently attending the patient for his MS. The source of information will be recorded in the CRF.

7.5.2.7 MS history

Patients will be asked to provide the date of diagnosis of MS and their history of relapses (i.e., total number of relapses and number of relapses requiring treatment). The oral MS history obtained from the patient or a caretaker will be supplemented by a thorough chart review and/or by an interview of the medical doctor currently attending the patient for his MS. The source of information will be recorded in the CRF.

In general, investigators must use their discretion to determine whether the following conditions apply:

<u>Relapse (retrospective definition)</u>
A relapse is the appearance of new neurological abnormality or the reappearance of a neurological abnormality, separated by at least 30 days from onset of a preceding clinical demyelinating event. The abnormality must be present for at least 24 hours and occur in the absence of fever or known infection (fever is defined as axillary temperature >37.5°C).

If a relapse is not documented in the patient chart, investigators must make a determination using the following questions as guidelines:

- Was the event described typical of a relapse?

- Was the duration of the event typical of a relapse?
- Did other family members substantiate the occurrence of the event?
- Was the patient hospitalized?
- Was the relapse treated? If yes, were corticosteroids used for treatment?

The final determination will be made at the discretion of the investigators. Relapses *confirmed* by an objective neurological examination performed by a qualified health professional and relapses reported subjectively by the patient should be reported separately.

Secondary progressive multiple sclerosis (SPMS)
If a diagnosis of SPMS is not documented in the patient chart, investigators must make a determination using the following guideline:

> *SPMS is defined as a progressive deterioration of disability for at least 12 months with an increase of at least one point between EDSS scores over the last 2 years (or a 0.5-increase between EDSS score of 6.0 and 6.5) with or without superimposed exacerbations following a relapsing-remitting course. The date of conversion to secondary progressive disease will be backdated to the onset of the deterioration in disability. In the opinion of the investigator, the deterioration of disability was not caused by relapses.*

The final determination will be made at the discretion of the investigators.

There has been no formal definition of SPMS in the original protocol, patients were – however – withdrawn from the study in case of "a phase of increasing disability that progresses unremittingly for six consecutive months" without specification of a EDSS quantification. Therefore, attempts should be made to reassess which of the patients of the NA cohort were true RRMS or rather SPMS patients at the date of study entry in 1988/89 applying the RRMS definition provided by Lublin and Reingold 1996 and the SPMS definition provided above:

> *Clearly defined disease relapses with full recovery or with sequelae and residual deficit upon recovery; periods between disease relapses characterized by a lack of disease progression.*

7.5.2.8 Medication history

Patients in the NA cohort will be questioned about their use of established or putative MS medications since they left the Betaseron® pivotal study. These medications might include Copaxone®, Avonex®, Rebif®, azathioprine, cyclophosphamide, methotrexate, mitoxanthrone, or any other medications used as an immunosuppressive or MS-disease-modifying agent. Also, medication to treat MS-related symptoms should be recorded (this also applies for the patients of the UK cohort).

Furthermore, patients should be explicitly queried for medication potentially interfering with cognitive functions, e.g., antiepileptic drugs, hyponotics, sedatives, or antipsychotic medication.

Patients will be asked to provide information on the length of time they took Betaseron® and reason(s) for discontinuation. The oral medication history obtained from the patient or a caretaker will be supplemented by a thorough chart review and/or by an interview of the medical doctor currently attending the patient for his MS. The source of information will be recorded in the CRF.

7.5.2.9 Magnetic resonance imaging (MRI)

All patients (i.e., the NA and the UK cohort) will undergo a single MRI scan according to a predetermined protocol. All MRI scans will be evaluated at a central analysis center (MRI-AC).

Note: MRIs should not be performed within 30 days of the last administration of corticosteroids.

Scans will be performed with 0.1 mmol/kg gadolinium-DTPA given intravenously. This is a standard diagnostic procedure in MS. The contrast medium may occasionally cause nausea and vomiting. It may cause warmth and pain at the injection site. Allergic reactions occur very rarely (anaphylactic shock in 13 of 5 million applications, based on post-marketing surveillance) and can be potentially serious in extremely rare instances.

MRI investigators and technicians from each center will be informed about technical implementation and image quality assurance prior to the start of the study. Thereafter, each center will perform a test run to assess image quality, repositioning and shipment procedures (for hardcopy and electronic image shipment to MRI-AC), and to evaluate the compatibility of archive media and format. Once the dry run is accepted, the parameter settings should remain unchanged for all scans obtained from study patients. A site can only start enrollment after acceptance of the MRI test run by the MRI-AC.

Quality of all scans will be assessed by the MRI-AC. The scans should be sent as hard copy to the MRI-AC within two working days, preferably along with a copy of the electronic image data, unless alternative routes have been established. After check of scan parameters, image quality and repositioning a fax will be sent to the contributing site within two working days of receipt of the scan confirming acceptance or rejection of the scan. Rejected scans should be repeated without delay and within the suggested 21-day study period.

A separate manual will describe the technical and logistical aspects of the MRI procedures in detail.

7.5.2.10 Laboratory Tests

The laboratory studies should be performed within 21 days after signing the informed consent. They will be performed in the patients of the NA cohort only. All laboratory tests will be performed in the central laboratory ACM. One copy of the results will be sent to the respective site and another one to the CRO for recording into the database. Original laboratory reports will be maintained at the respective study centers, where investigators will confirm receipt and review of data by signing the laboratory form. Serum samples for neutralizing antibodies to IFNβ will be analyzed in a different central laboratory, i.e. at MediTest (Laupheim, Germany) but handled through the central clinical laboratory ACM.

TT 3 Laboratory Parameters

Blood chemistry	creatinine, aspartate transaminase (AST/SGOT), alanine transaminase (ALT/SGPT), gamma glutamate transferase (γ-GT), alkaline phosphatase bilirubin, albumin
Hematology	erythrocytes, leukocytes, differential count, thrombocytes, hemoglobin, hematocrit
Thyroid parameters	thyroid-stimulating hormone (TSH)<br>TPO-Thyroid microsomal Abs
Neutralizing antibodies	antibodies found to bind to interferon β will be evaluated for the neutralization of activity as measured in the MxA protein assay
Other antibodies	Rheumatoid arthritis, Lupus erythematodes

In addition to the blood sampling for the above described laboratory investigations, one serum sample will be obtained and stored. It may be used for analyses of immunological parameters. Results may be compared with results generated in samples obtained during the pivotal study, where appropriate. The analyses may use proteonomic and metabolomic techniques.

Furthermore, it is intended to perform pharmacogenetic evaluations. The patient's participation in these pharmacogenetic evaluations will be subject to a separate informed consent and, therefore, will be optional and independent of the participation in the study. A patient's agreement to or refusal of consent for this sampling will have no effect on the remainder of the study procedures. The scientific, ethical, and procedural details of these evaluations are summarized in Appendix 12. Appendix 2 contains the separate patient information and informed consent form.

A separate manual will describe the technical and logistical aspects of the laboratory procedures in detail.

7.5.2.11 Adverse reactions

Patients on the NA cohort will be questioned about specific adverse reactions of Betaseron® that they might have experienced since leaving the Betaseron® pivotal study. These include flu-like symptoms, injection site reactions, fever, headache, malaise, myalgia, and increased liver transaminases. Those pre-specified adverse reactions will be only recorded for the duration of actual treatment with Betaseron®. They will not be recorded for such times when patients did not receive Betaseron®. In addition, discontinuation of Betaseron® due to any adverse reactions, including the timing of that discontinuation, should also be documented.

Adverse events and serious adverse events will not be collected and followed other than described above. All adverse events that occurred since the clinical trial ended or that will occur in the future after the study visit (serious or non-serious) are assumed to have been, or will be, reported through the usual channels by the physician providing care to that patient. The only exemption is the investigational period. During that period, the usual AE reporting and follow-up as described in Section 7.5.5.1 (page 28 ff.) will be applied.

7.5.3 Appropriateness of measurements

The assessments proposed in this study protocol are considered to be "standard assessments", i.e., widely used and generally recognized as reliable, accurate, and relevant.

7.5.4 Efficacy variables

7.5.4.1 Primary efficacy variable(s)

This is primarily an observational and descriptive study. It is not possible to define a primary efficacy variable a priori. The study may generate hypotheses about potential efficacy- or effectiveness-related endpoints. Hypotheses may be tested in later follow-up studies based on endpoints identified and effect sizes observed in this study.

7.5.4.2 Efficacy and/or effectiveness related variable(s)

The following variables related to MS status and clinical symptomatology will be targeted:

- Survival
- Level of disability/function as measured by EDSS*
- Level of disability/function as measured by MSFC
- Time to EDSS level of 3.0 from retrospective data review*
  (in the subset of patients with an EDSS score < 3.0 before start of pivotal study)
- Time to EDSS level of 6.0 from retrospective data review*
- Ambulatory status (time to first use of an ambulatory device, time to dependence on an ambulatory device, time to first use of a wheelchair)
- Conversion to secondary progressive multiple sclerosis (SPMS)

---

* The conventions used in the NH cohort should be followed (cf. Section 7.5.2.4).

The following MRI-derived variables will be targeted:

- volume of hyperintense lesions on T2-weighted images ("T2 burden of disease"; T2-BOD)
- normalized brain volume
- number of gadolinium-enhancing lesions on T1-weighted images
- number and volume of hypointense lesions on non-enhanced T1-weighted images ("black holes")
- cervical cord imaging

The MR spectroscopy as a method with putative information on demyelination and axonal loss may be applied as well in a subset of patients. Details on MR spectroscopy will be provided in a sub-study protocol for the study centers participating in that additional investigation.

In addition, scores from individual assessments of the neuropsychological and HRQoL scales and tests will be evaluated as variables with potential information about efficacy or effectiveness.

7.5.5 Safety variables

7.5.5.1 Adverse events

An **adverse event (AE)** is normally defined as any untoward medical occurrence in a patient administered a pharmaceutical product and which does not necessarily have to have a causal relationship with this treatment. As patients in this study will not receive any protocol-stipulated pharmaceutical product – with the exemption of gadolinium-DTPA – AEs in this study will be defined as any untoward medical occurrence in a patient administered **any** pharmaceutical product and which does not necessarily have to have a causal relationship with this pharmaceutical product.

An AE can therefore be any unfavorable and unintended sign (including an abnormal laboratory finding), symptom, or disease temporally associated with the use of a medicinal product, whether or not the AE is considered related to the medicinal product. AEs are to be coded using an internationally recognized dictionary.

It should be noted that only AEs occurring during the investigational period will be reported and followed as described in this Section.

7.5.5.1.1 Documentation

Attention is to be paid to the occurrence of AEs at all stages of the examination. Thus, the patient should be closely observed by the investigator both during and after the examination.

Any AEs (observed, volunteered, or elicited) are to be documented in detail indicated on the case report form (CRF). The following information is required.

- The date and time of onset of any AEs
- The duration (the entire duration of an event or symptom, calculated from date of onset and date of end, if not recorded directly)
- The maximum intensity (mild, moderate or severe; for definitions, see below).
- The relationship of the AE a pharmaceutical product used by the patient
- Any action taken by the investigator to resolve the AEs
- The outcome of the AE (recovered/resolved, recovering/resolving, not recovered/not resolved, recovered/resolved with residual effects, fatal).
- The main pattern of the AE (every drug administration, intermittent, continuous, other).
- An assessment of the seriousness of the AE will be made by the investigator, who is to complete a special form provided by the Sponsor in the case of a serious adverse event (SAE). However, SAEs will also be recorded briefly on the "Adverse Event Form" of the CRF. A definition of SAEs is provided below.

Intensity

The investigator is to classify the intensity of an adverse event according to the following definitions:

Mild	The intensity of the AE is assessed as mild,<br>taking into account the possible range of the intensity of the AE. An indicator could be the preservation of function in the concerned organ system despite the occurrence of the AE.
Moderate	The intensity of the AE is assessed as moderate,<br>taking into account the possible range of the intensity of the AE. An indicator could be any of function in the concerned organ system.
Severe	The intensity of the AE is assessed as severe,<br>taking into account the possible range of the intensity of the AE. An indicator could be the loss of function in the concerned organ system.

Relationship with a pharmaceutical product

The investigator is to classify the drug relationship of an AE according to the following definitions:

None	The time course between administration of the pharmaceutical product and occurrence or worsening of the AE rules out a causal relationship<br>*and/or*<br>another cause is confirmed and no indication of involvement of the pharmaceutical product in the occurrence/worsening of the AE exists.

Unlikely	The time course between administration of the pharmaceutical product and occurrence or worsening of the AE makes a causal relationship unlikely<br>*and/or*<br>the known effects of the pharmaceutical product or of the substance class provide no indication of involvement in occurrence/worsening of the AE and another cause adequately explaining the AE is known<br>*and/or*<br>regarding the occurrence/worsening of the AE a plausible causal chain may be deduced from the known effects of the pharmaceutical product or the substance class, but another cause is much more probable<br>*and/or*<br>another cause is confirmed and involvement of the pharmaceutical product in the occurrence/worsening of the AE is unlikely.
Possible	Regarding the occurrence/worsening of the AE, a plausible causal chain may be deduced from the pharmacological properties of the pharmaceutical product or the substance class, but another cause just as likely to be involved is also known<br>*or*<br>although the pharmacological properties of the pharmaceutical product or the substance class provide no indication of involvement in the occurrence/worsening of the AE, no other cause gives adequate explanation
Probable	The pharmacological properties of the pharmaceutical product or of the substance class<br>*and/or*<br>the course of the AE after dechallenge and, if applicable, after rechallenge<br>*and/or*<br>specific tests (e.g. positive allergy test, antibodies against pharmaceutical product/metabolites) suggest involvement of the pharmaceutical product in the occurrence/worsening of the AE, although another cause cannot be ruled out.
Definite	The pharmacological properties of the pharmaceutical product or of the substance class<br>*and*<br>the course of the AE after dechallenge and, if applicable, after rechallenge<br>*and*<br>specific tests (e.g. positive allergy test, antibodies against pharmaceutical product/metabolites) indicate involvement of the pharmaceutical product in the occurrence/worsening of the AE and no indication of other causes exists.
Unclassifiable	The available information is not sufficient for causality assessment.<br>(The option "unclassifiable" is not available on the AE form.)

Serious adverse events

A **serious adverse event (SAE)** is classified as any untoward medical occurrence that at any dose

- results in death, or
- is life-threatening, or
- requires inpatient hospitalization or prolongation of existing hospitalization, or

- results in persistent or significant disability/ incapacity, or
- is a congenital anomaly/ birth defect

Medical and scientific judgment should be exercised in deciding whether expedited reporting is appropriate in other situations, such as important medical events that may not be immediately life-threatening or result in death or hospitalization but may jeopardize the patient or may require intervention to prevent one of the other outcomes listed in the definition above. These should also usually be considered serious. Examples of such events are intensive treatment in an emergency room or at home for allergic bronchospasm; blood dyscrasias or convulsions that do not result in hospitalization; or development of drug dependency or drug abuse.

The investigator should take appropriate diagnostic and therapeutic measures to minimize the risk to the patient. Where appropriate he/she should take diagnostic measures to collect evidence for clarification of the relationship between the SAE and the pharmaceutical product.

The investigator must submit a complete SAE Report for all SAEs, regardless of a possible causal relationship, to Berlex's Global Medical Safety Surveillance function **immediately** (at the latest within 48 hours of having gained knowledge of the event). This report is to be sent on the SAE Report Form provided by the Sponsor. The investigator is required to document in full the course of the SAE and any therapy given, including any relevant findings / records in the report. The investigator will also inform the Sponsor of the relevant follow up information and the outcome of the SAE as soon as possible using the Sponsor's standard form.

The primary contacts will be:

United States	Berlex Global Medical Safety Surveillance<br>Telephone +1-888-237-5394, option #3, then option #8<br>Telefax +1-973-305-5447
Canada	Dr. Jean-Louis Stril<br>Telefax +1-514-782-2243

All SAEs after signature of the informed consent must be documented.

In addition, SAEs will also briefly be recorded on the "Adverse Event Form" of the CRF if they occur after administration of treatment.

7.5.5.1.2 Expected adverse events

Expected (or "listed") AEs for the patients of the NA cohort treated with Betaseron® are such that are listed in the prescribing information. The U.S. Prescribing Information and the Canadian Product Monograph for Betaseron® are appended to this protocol.

Typically disease-related AEs:
AEs which are typically caused by the underlying disease (MS), e.g. relapses and neurological deterioration, paresthesia or anesthesia, are expected AEs.

<u>Risks related to the injection of gadolinium-DTPA:</u>
MRI scans will be enhanced with gadolinium-DTPA given intravenously. Contrast-agent enhanced MRIs are a standard diagnostic procedure in MS. The contrast medium may occasionally cause nausea and vomiting. It may cause warmth and pain at the injection site. Allergic reactions occur very rarely (anaphylactic shock in 13 of 5 million applications, based on post-marketing surveillance) and can be potentially serious in extremely rare instances.

Other expected (or "listed") AEs are such that are described in the U.S. Prescribing Information and the Canadian Product Monograph for Magnevist®(gadolinium-DTPA).

The injection itself may be accompanied by mild bruising and also, in rare cases, by a transient inflammation of the vessel wall. After initial irritation, the site of venipuncture is usually painless and hardly noticeable.

<u>Risks related to blood sampling:</u>
On all visits including blood sampling, venipuncture with disposable needles will be performed, usually into cubital veins. Blood sampling may be accompanied by mild bruising and also, in rare cases, by a transient inflammation of the vessel wall. After initial irritation, the site of venipuncture is usually painless and hardly noticeable.

<u>Blood loss:</u>
The total amount of blood withdrawn during the study will be approx. 40 mL at maximum* (i.e., approx. slightly less than three tablespoons or a tenth of a normal blood donation: 450 or 500 mL). For details, cf. TT 4 below:

TT 4 Blood loss

Blood chemistry, thyroid parameters, rheumatoid arthritis antibodies, Lupus erythematodes antibodies	serum	10.0 mL
Hematology	EDTA	3.0 mL
Neutralizing antibodies	serum	7.5 mL
Immunological analyses	serum	7.5 mL
Pharmacogenetic analyses*	EDTA	12.0 mL*
**Total**		**40.0 mL**

*Pharmacogenetic analyses are optional and subject to separate consent (cf. Section 7.5.2.10, page 25). The blood loss in patients <u>not</u> consenting to the genetic analyses is 28 mL (approx. two tablespoons). Patients of the UK cohort will be sampled for immunological and genetic analyses only. Blood loss in the UK patients is therefore 19.5 mL or 7.5 mL, respectively.

7.5.5.1.3 Unexpected adverse drug reaction

Reports have to be considered as unexpected if they add significant information on the specificity or severity of an expected adverse reactions. The expectedness of an AE shall be determined by the Sponsor according to the Investigators Brochure and the relevant Prescribing Information.

Examples would be (a) acute renal failure listed in the Investigator's Brochure with a subsequent new report of interstitial nephritis and (b) hepatitis with a first report of fulminant hepatitis. "Unexpected" as used in this definition, refers to an adverse drug experience that has not been previously observed and included in the product information rather than from the perspective of such experience not being anticipated from the pharmacological properties of the investigational product.

7.5.6 Pharmacokinetic and bioanalytical methods

There will be no pharmacokinetic investigations in the study. Certain proteins – as described below – may be used to get an estimate of bioavailability for patients with an ongoing treatment of Betaseron®.

It is intended to compare samples preserved from the time of conduct of the pivotal study with a sample to be obtained in this study. Comparisons may include mass spectrometry-based analysis and broad profiling of proteins and low-molecular-weight organic molecules, cytometric analysis and profiling of different cell populations and cell-surface markers, or immunoassays for quantitative analysis of proteins and peptides.

7.6 Data quality assurance

Due to the nature of the study, only one on-site monitoring visit will be scheduled per center. The remaining support and management of study centers will occur by telephone contact and fax. On-site monitoring can and will occur in the event of a for cause need.

For source data verification, at least the following information must be included in the patient note / file :

- Patient's demographic data.
- Date of patient's written informed consent,
- the fact that the patient is in a study and the study number,
- patient's visit dates,
- serious adverse events,
- adverse events,
- confirmation of the diagnosis of the indication being treated,
- concomitant medication and diseases,

The CRA will collect the appropriate copy or copies of the completed forms. In addition, the CRA will determine whether all AEs and SAEs have been appropriately reported within the time periods required (SAEs only).

A member of the Quality Assurance Unit (Corporate Clinical Quality Assurance function) may arrange to visit the investigator in order to audit the performance of the study at the study site and the study documents originating there. The auditor(s) will usually be accompanied by the CRA or the study manager. The investigator will be informed about the outcome of the audit.

In addition, inspections by health authority representatives - including foreign authorities - and Independent Ethics Committees are possible at any time. The investigator is to notify the Sponsor of any such inspection immediately.

7.7 Statistical methods and determination of sample size

7.7.1 Statistical and analytical plans

7.7.1.1 General

The sponsor or its designee will perform all statistical analyses. All analyses will be detailed in a formal statistical analysis plan that will be prepared before database release for final analysis.

Demographic and background variables, such as age, race, sex, MS history, MRI and EDSS scores for the various cohorts will be analyzed by means of summary statistics, i.e., N and percentage for categorical variables and N, mean, standard deviation, minimum, lower quartile, median, upper quartile, and maximum for continuous variables.

7.7.1.2 Analysis of efficacy- and effectiveness-related variables

The study is exploratory in nature. No one particular variable can be designated as primary or secondary variable. The analyses of efficacy variables will be descriptive in nature for the NA cohort and where applicable for the NH cohort and the UK cohort of untreated patients. These variables include survival for the NA cohort, MS status as assessed by EDSS, MFSC and conversion to SPMS and any other relevant variable related to MS status. Also descriptive analyses will be provided for neuropsychological, HRQoL and resource use data, functional system scores, MRI parameters and health-related quality of life.

For the NA cohort, the descriptive analyses will also be stratified by the original clinical trial group assignment (low dose, high dose, and placebo). Time of exposure to Betaseron in the NA cohort will be determined appropriately and descriptive statistics will be provided based on time of exposure.

Comparison of the three cohorts based on selected characteristics will be performed descriptive after appropriate matching of the patients. Any comparison between the NA cohort and the UK cohort will be restricted to patients of the NA cohort that are alive.

7.7.1.3 Analysis of safety variables

The number and percentage of AEs will be presented by body system and intensity.

Summary statistics (N, mean, standard deviation, minimum, lower quartile, median, upper quartile, and maximum) for each clinical laboratory variable assessed in the NA cohort will be presented.

7.7.2 Determination of sample size

Since this study is exploratory in nature and no one particular variable can be selected as primary variable, the determination of sample size is not applicable.

A total of 372 patients participated in Betaseron® pivotal study (215 in the U.S. and 157 in Canada, cf. TT 5 below). Attempts will be made to obtain information about all patients and to examine as many patients as possible.

TT 5 Enrollment of the pivotal study by study site

Study Site	Placebo	Betaseron®		Total
		1.6 MIU	8 MIU	
Temple Univ Philadelphia	10	10	10	30
Thomas Jefferson Univ Philadelphia	10	10	10	30
Univ of California in San Francisco	10	10	10	30
Univ of Alabama Birmingham	4	4	4	12
Univ of Arizona Tuscon	12	12	11	35
Univ of Chicago	16	16	16	48
Univ of Maryland Baltimore	10	10	10	30
Hôpital de Notre Dame Montréal	12	12	12	36
Institut et Hôpital Neurologiques de Montréal	8	8	8	24
Univ of British Columbia Vancouver	17	18	17	52
London Health Sciences Centre London (Ontario)	14	15	16	45
**Total**	123	132	132	372

It is targeted to recruit approximately 60 untreated patients for the UK cohort. Limitations due to feasibility may exist.

7.7.3 Use of data in an epidemiological model

Data may be also used to populate an already existing epidemiological MS model.

7.8 Ethics

7.8.1 Independent Ethics Committee and Institutional Review Board

The study will commence only after the protocol has been approved by the appropriate Institutional Review Board (IRB) or Independent Ethics Committee (IEC) and written notification of the approval has been received by the Sponsor.

Neither the investigator nor the Sponsor will modify or alter this protocol without first obtaining the written agreement of the other. Alterations considered to be significant by the Sponsor's Core Clinician or by the investigator must be approved by the appropriate IEC/IRB prior to implementation, except where immediate implementation to eliminate an imminent hazard to the patient is necessary.

All protocol amendments that are agreed upon must be recorded on the standard Protocol Amendment Form, and must be signed and dated by both the Sponsor and the investigator.

7.8.2 Ethical conduct of the study

The planning and conduct of this clinical study are subject to national laws and the standard operating procedures for clinical investigation and documentation applicable in the Schering Group, including Berlex Laboratories. In the US, the study will be conducted in accordance with Good Clinical Practice (GCP) as required by 21 Code of Federal Regulations (CFR) Parts 50, 56 and 312. Compliance with these requirements also constitutes conformity with the ethical principles that have their origin in the Declaration of Helsinki and the International Conference on Harmonization (ICH)/GCP Guidelines of 17 Jan 1997.

At the discretion of the Sponsor's Core Clinician, the entire study may be cancelled for medical reasons. In addition, the Sponsor retains the right to end the study at any time if the study cannot be carried out as agreed upon in the study protocol. In case of premature termination the investigators, IRB/IECs and Regulatory Authorities will be informed by the Sponsor.

7.8.3 Patient information and consent

Attempts to contact patients who participated in the Betaseron® pivotal study will be made only after the individual IRB/IECs grant waivers, where needed, allowing each study center to contact these patients for long-term follow-up (e.g., "HIPAA waiver" for the U.S.). Patients who agree to participate will be asked to sign an informed consent form. Different informed consent form will be used for each of the cohorts, because study activities differ for the study cohort and the untreated MS cohort.

The investigator will explain the nature of the study, its purpose and associated procedures, the expected duration and the potential benefits and risks of participation to each patient prior to

his/her entry into the study (i.e. before examinations and procedures associated with selection for the study are performed). The information given will be based on a sample patient information and informed consent sheet provided by the Sponsor. The investigator will provide the patient with an IRB/IEC-approved patient information and informed consent form. Each patient will have ample opportunity to ask questions and will be informed about the right to withdraw from the study at any time without any disadvantage and without having to provide reasons for this decision.

Following this informative discussion a patient will be asked if he/she is willing to personally sign and date a statement of informed consent (general study). Only if the patient voluntarily agrees to sign the informed consent statement, and has done so, may he/she enter the study. The patient will receive a copy of his/her signed and dated form.

The informed consent statement is to remain in the investigator's files. The investigator will document on each CRF that he/she has informed the patient and that the patient has signed the informed consent statement.

If the patient is not capable of providing a signature, a verbal statement of consent can also be given in the presence of a witness. This is to be documented by a signature from the informing physician as well as by a signature from the witness. This procedure is subject to an explicit IRB/IEC approval.

The informed consent form and any other written information provided to patients will be revised whenever important new information becomes available that may be relevant to the patient's consent, or when there is an amendment to the protocol which necessitates a change to the content of the patient information and / or the written informed consent form. The investigator will inform the patient of changes in a timely manner and will ask the patient to confirm to continue his/her participation in the study by his/her signature on the revised informed consent form. Any revised written informed consent form and written information must receive the IRB/IEC's approval / favorable opinion in advance of use.

A sample informed consent form is part of the attachments to this protocol.

7.9 Data handling and record keeping

Data required according to this protocol are to be recorded on the CRFs provided by the Sponsor as soon as possible. Entries on the CRF must be made with a ballpoint pen and must be legible. Pencils and correction fluids may not be used.

If corrections are necessary they will be entered by an authorized member of the investigator's staff in the following manner: the wrong entry will be crossed out with a single line so that the text remains legible, and the correct entry will be placed next to it. Corrections will be initialed and dated.

Any documents related to the study must be archived at the study site or in a central archive. This includes the careful listing of the identities of the patients involved in the study. This list

and the signed informed consent statements are key documents in the files to be stored by the investigator.

All documents related to the study must be retained until at least 15 years after the end of the study. At the end of this period, the Sponsor will inform the investigators as to when these documents no longer need to be retained.

Patient (hospital) files will be archived according to local regulations.

7.10 Drug accountability

Not applicable.

7.11 Financing

Funding for the study will be agreed between the investigator and the Sponsor and must be confirmed in writing before the study commences.

7.12 Financial disclosure

Each investigator (including the principal investigator and any subinvestigators) who is directly involved in the treatment or evaluation of research subjects must disclose certain financial arrangements. The following arrangements with, and interests of, investigators (including the spouse and dependent children) are disclosable to the FDA:

- Compensation made to the investigator in which the value of compensation could be affected by study outcome (*e.g.*, higher compensation for a favorable outcome than for an unfavorable outcome, or a royalty interest related to product sales);
- A proprietary interest by the investigator in the tested product, including, but not limited to, a patent, trademark, copyright or licensing agreement;
- Any equity interest in the Sponsor of this study, *i.e.*, any ownership interest, stock options, or other financial interest whose value cannot be readily determined through reference to public prices, or any equity interest in a publicly held company that exceeds $ 50,000 in value held during the time the investigator is carrying out the study and for one year following completion of the study;
- Significant payments of other sorts, *i.e.*, payments that have a cumulative monetary value of $ 25,000 or more made by the Sponsor of a covered study to the investigator or the investigator's institution to support activities of the investigator exclusive of the costs of conducting the clinical study or other clinical studies, (*e.g.*, a grant to fund ongoing research, compensation in the form of equipment or retainers for ongoing consultation or honoraria)

during the time the investigator is carrying out the study and for 1 year following completion of the study.

In this context "investigator" is defined as all individuals listed on FDA form 1572 – or for non-IND studies performed outside the U.S. listed in the signature list – directly involved in the treatment or evaluation of research subjects. The term also includes the spouse and each dependent child of the investigator.

A financial disclosure statement must be provided to the Sponsor for each investigator (including each subinvestigator in IND studies identified on FDA Form 1572) at a study site before the study can commence. Financial disclosure statements must also be provided at the time the study is closed and at the 1-year anniversary of study closure.

7.13 Publication policy

It is intended to publish the results of the clinical trial in an accepted scientific journal. The Signatory Investigator (identified in Section 4.3), the Sponsor's Core Clinician, a representative of the MRI-AC, the Neuropsychological Auditor, and a representative of the Sponsor's Outcomes Research function will coordinate a joint publication. The Signatory Investigator will provide the main clinical publications and will review all other publications on any study results prior to their submission (e.g., separate publications on results of MRI or neuropsychological investigations or other fields of interest).

In order to give the Sponsor the opportunity to raise objections, all manuscripts including study results or parts of them must first be submitted to the Sponsor for review before any submission of any results or part of results to such scientific journal or any other journal or any publishing house will be made. The Sponsor will not unduly delay publication. All objections raised by the sponsor which are well-founded and not arbitrary will be considered.

A number of sub-studies have been proposed. These sub-studies will run under their own protocols with a separate informed consent. The provisions for publications outlined above do also apply to publications that the investigator intends to publish as a single center or in conjunction with a group of centers. However, this kind of publication may not be submitted before completion of the entire study and the submission of the principal publications.

8. Reference list

1. Benedict RH, Fischer JS, Archibald CJ et al. Minimal neuropsychological assessment of MS patients: a consensus approach. Clin Neuropsychol 2002;16:381-97.
2. Cella DF, Dineen K, Arnason B et al. Validation of the functional assessment of multiple sclerosis quality of life instrument. Neurology. 1996 Jul;47(1):129-39.
3. Confavreux C, Vukusic S, Moreau T, Adeleine P. Relapses and progression of disability in multiple sclerosis. N Engl J Med 2000;343:1430-8.
4. Cottrell DA, Kremenchutzky M, Rice GP et al. The natural history of multiple sclerosis: a geographically based study. 5. The clinical features and natural history of primary progressive multiple sclerosis. Brain 1999;122:625-39.
5. Cottrell DA, Kremenchutzky M, Rice GP et al. The natural history of multiple sclerosis: a geographically based study. 6. Applications to planning and interpretation of clinical therapeutic trials in primary progressive multiple sclerosis. Brain 1999;122:641-7.
6. Cutter GR, Baier ML, Rudick RA et al. Development of a multiple sclerosis functional composite as a clinical trial outcome measure. Brain 1999;122:871-82.
7. Henriksson SF, Fredriksson S, Masterman T, Jönsson B. Costs, quality of life and disease severity in multiple sclerosis: A cross-sectional study in Sweden. Eur J Neurol 2001;8:27-35.
8. Kobelt G, Lindgren P, Smala A et al. Costs and quality of life in multiple sclerosis. An observational study in Germany. Eur J Health Economics 2001;2:60-8.
9. Kobelt G, Miltenburger C, Kaskel P, Jönsson B. The MS Standard Health Care Survey Questionnaire (SHCS): a patient-reported measure for resource use assessment in multiple sclerosis. Accepted for presentation at ECTRIMS Vienna. Mult Scler 2004
10. Kurtzke JF. Rating neurologic impairment in multiple sclerosis: an expanded disability status scale (EDSS). Neurology 1983;33:1444-52.
11. Kremenchutzky M, Cottrell D, Rice G et al. The natural history of multiple sclerosis: a geographically based study. 7. Progressive-relapsing and relapsing-progressive multiple sclerosis: a re-evaluation. Brain 1999;122:1941-50.
12. Lublin FD, Reingold SC. Defining the clinical course of multiple sclerosis: results of an international survey. Neurology 1996;46: 907-11.
13. Paty DW, Li DKB, the UBC MS/MRI Study Group, and the IFNB Multiple Sclerosis Study Group. Interferon beta-1b is effective in relapsing-remitting multiple sclerosis, Neurology 1993;43:662-7.
14. Poser CM, Paty DW, Scheinberg L et al. New diagnostic criteria for multiple sclerosis: guidelines for research protocols. Ann Neurol 1983;13:227-31.
15. Tappenden P, Chilcott J, O'Hagan T et al. Cost effectiveness of beta interferons and glatiramer acetate in the management of multiple sclerosis – Final report to the National Institute for Clinical Excellence. July 2001

16. The EuroQol Group. EuroQol – a new facility for the measurement of health-related quality of life. Health Policy 1990;16:199-208.

17. The IFNB Multiple Sclerosis Study Group. Interferon beta-1b is effective in relapsing-remitting multiple sclerosis. Neurology 1993;43:655-61.

18. The IFNB Multiple Sclerosis Study Group and the University of British Columbia MS/MRI Analysis Group. Interferon beta-1b in the treatment of multiple sclerosis. Neurology 1995;45:1277-85.

19. Weinshenker BG, Bass B, Rice GP et al. The natural history of multiple sclerosis: a geographically based study. 1. Clinical course and disability. Brain 1989;112:133-46.

20. Weinshenker BG, Rice GP, Noseworthy JH et al. The natural history of multiple sclerosis: a geographically based study. 2. Predictive value of the early clinical course. Brain 1989;112:1419-28.

21. Weinshenker BG, Rice GP, Noseworthy JH et al. The natural history of multiple sclerosis: a geographically based study. 3. Multivariate analysis of predictive factors and models of outcome. Brain 1991;114:1045-56.

22. Weinshenker BG, Rice GP, Noseworthy JH et al. The natural history of multiple sclerosis: a geographically based study. 4. Applications to planning and interpretation of clinical therapeutic trials. Brain 1991;114:1057-67.

23. Zigmond P, Snaith S. The Hospital Anxiety and Depression Scale. Acta Psychiatr Scand 1983;13:141-7.

9. Abbreviations

AE	Adverse event
ALT	Alanine aminotransferase
AST	Aspartate aminotransferase
COWAT	Controlled Oral Word Association Test
CRA	Clinical Research Associate
CRO	Contract research organization
CRF	Case report form
CVLT-II	California Verbal Learning Test II
D-KEFS	Delis-Kaplan Executive Function System
DTPA	Diethylenetriaminepentaacetate
EDSS	Expanded Disability Status Scale
EQ-5D	EuroQol 5-Dimensional Questionnaire
FAMS	Functional Assessment in Multiple Sclerosis
FDA	Food and Drug Administration
FS	Functional system
FSS	Functional System Score
GCP	Good Clinical Practice
γ–GT	Gamma glutamate transferase
ICH	International Committee on Harmonization
IEC	Independent Ethics Committee
IFN	Interferon
IFNB	Interferon beta
IRB	Institutional Review Board
IU	International units
MACFIMS	Minimal assessment of cognitive function in MS
MIU	Million international units
MRI	Magnetic resonance imaging
MRI-AC	MRI analysis center
MS	Multiple sclerosis
MSFC	Multiple sclerosis functional composite
MS-SHCS	Multiple Sclerosis Standard Health Care Survey
NA	North America
NABs	Neutralizing antibodies
NART	National Adult Reading Test
NH	Natural history
PASAT	Paced Auditory Serial Addition Task
PPMS	Primary progressive multiple sclerosis
HRQoL	Health-related quality of life
RBC	Red blood cells
RNA	Ribonucleic acid
RRMS	Relapsing-remitting multiple sclerosis
SDMT	Symbol Digit Modality Task
SPMS	Secondary progressive multiple sclerosis
TMF	Trial master file
TSH	Thyroid stimulating hormone
UK	United Kingdom of Great Britain and Northern Ireland
US	United States of America
WTAR	Wechsler Test of Adult Reading Ability

10. Attachments

Appendix 1	Sample patient information and informed consent form (non-genetic part)
Appendix 2	Sample patient information and informed consent form (genetic part / optional)
Appendix 3	21 CFR 50.25 – Elements of informed consent
Appendix 4	Sample signature sheet for investigators
Appendix 5	Betaseron® – Prescribing Information Documents for the US and Canada
Appendix 6	Magnevist® – Prescribing Information Documents for the US and Canada
Appendix 7	Protocol TB01-35686 (US version)
Appendix 8	Investigator-administered neuropsychological tests
Appendix 9	Patient-reported scales and questionnaires
Appendix 10	Expanded Disability Status Scale (EDSS)
Appendix 11	Multiple Sclerosis Functional Composite Measure (MSFC)
Appendix 12	Pharmacogenetic investigations

Appendix 1

Patient information and informed consent form for patients in North America

Patient Information and Informed Consent Form

Study no:	308272
Date/Version of study protocol:	20 August 2004<br>Final Proposed Protocol / Version 3.0
Date/Version of the patient information and informed consent form:	20 August 2004<br>Version 2.0
Title of study protocol:	A long-term follow-up of patients enrolled in the pivotal study of Betaseron® (interferon beta 1b) in relapsing-remitting multiple sclerosis
Patient no.:	___ ___ ___ ___
Manufacturer of the investigational product:	No investigational product is given in this study.

Patient Information Leaflet for the study 308272

"A long-term follow-up of patients enrolled in the pivotal study of Betaseron® (interferon beta 1b) in relapsing-remitting multiple sclerosis"

Dear patient,

You are being asked to consider if you would like to participate in this long-term follow-up study because you participated in one of the first research studies with Betaseron® in relapsing-remitting multiple sclerosis (RRMS) approximately sixteen (16) years ago. This study led to the approval of the drug for use in RRMS by many governmental health protection authorities, among them the FDA and Health Canada. Therefore, the study is referred to as the "pivotal trial".

This new study is being sponsored by Berlex Pharmaceuticals, Inc. (Berlex) and is being managed by PPD Medical Communications (PPD). The following information describes the study and your role as a possible participant. Please read this information carefully and do not hesitate to ask any questions about the information provided.

To participate, you must have been previously enrolled in the Betaseron® pivotal study that began in 1988, be willing to complete all study procedures and answer questions relating to your experiences with Multiple Sclerosis (MS). This study will include approximately seven (7) physicians in the United States and four (4) in Canada, and will enroll approximately 300 patients. In addition, approximately 60 patents who have never been treated for their MS will be enrolled in the United Kingdom. Your participation in this study will begin when you sign this consent and will end when

you complete all study procedures. There is also a genetic sub-study planned. Your participation in this sub-study is optional and independent of participation in this study. Your doctor will discuss this sub-study with you separately.

Purpose

The purpose of this follow-up study is to systematically study the long-term course of the disease and possibly the long-term effectiveness of Betaseron® by evaluating the patients that were previously enrolled in the pivotal study. In addition, these patients will be compared with the group of patients from the U.K. that have never received treatment for MS. Furthermore, characteristics of the course of the disease from the patients in the pivotal study will also be compared with those of a natural-history database composed of MS patients from Ontario who have not been treated for their disease.

Procedures

Participation in the study involves collecting data regarding your MS status. This data can be collected at one study visit or during several visits during a 21-day period after signing consent. The following procedures will be done:

- Your doctor will ask you about your medical history that includes your general health, MS history, current status of your MS, information regarding the medications you have taken for your MS, and information about medications that may interfere with cognitive function.
- Your doctor will perform a neurological examination and use three standardized tests, the Functional Systems Scores, Expanded Disability Status scale, and Multiple Sclerosis Functional Composite to assess your level of disability.
- Your doctor will have you perform a series of cognitive tests to assess memory, learning, vision, attention, cognitive ability and language. These tests will take approximately 60-90 minutes to complete. The tests are available in English and French. If those languages are not your first language, you are invited to take the tests in one the provided versions, however your participation is strictly voluntary. You may decide not take the test in the provided versions. Not taking the tests will not affect the participation in the other parts of the study.
- A Magnetic Resonance Imaging (MRI) scan will be performed, the quality of which will be assessed by a Central Analysis Center. If the quality of the MRI scan is not acceptable, you may have to undergo an additional MRI scan.
- Laboratory Testing will include blood chemistry, hematology, thyroid panel, neutralizing antibodies against Betaseron®, and immunological tests. The blood sample for immunological tests may be used for comparison with blood samples taken during the pivotal study.
- You will be asked to complete four (4) questionnaires that ask your opinion of your health, mood, and the quality of your life. These questionnaires are available in English and French.

This will be the end of your obligations in this study. Some of these procedures may be done as part of your standard care even if you don't take part in this study. The study doctor or his staff can answer any questions you may have about the procedures that are not part of your standard care.

Risks and Discomforts

Blood samples will be taken and you might feel pain or light-headed from this. Further, the procedure has the usual risks coming with blood sampling, e.g., possible bleeding from the puncture site, bruising, pain, blood clot formation, or local infection and inflammation in the arm where the puncture was made (very rare cases). The amount of blood withdrawn during the study will be approximately 28 milliliters (approx. two tablespoons).

An MRI scan is a procedure that uses magnetic fields and radio waves (not x-rays) to take pictures of the brain. An MRI scan can be a noisy procedure, and some people may feel uncomfortable while lying in the scanner. People with pacemakers or certain other metallic implants may not undergo an MRI scan. In order for the scanner to detect areas of inflammation in your brain, a substance called gadolinium will be injected into your body through an intravenous (directly into your vein) line. Gadolinium can cause nausea, vomiting and slight warmth or pain at the injection site. Allergic reactions may also occur very rarely, and, in extremely rare instances, can be potentially serious. If you know that you have previously had a reaction to gadolinium, please be sure to inform your study doctor and you will not be required to undergo the MRI.

Participation in research may cause a loss of privacy. Berlex will do everything possible to ensure that information obtained from this study will never be inappropriately revealed to third parties. For further information on confidentiality, see Section "Confidentiality and authorization to use and disclose medical information" below.

Benefits

While there is no direct benefit associated with your participation in this study, it would provide your doctor with a profile of your experience with RRMS. With this and other information collected directly from this study, physicians may make more informed treatment decisions in the future for your care. By taking part in this registry study, you may contribute new information that may benefit other patients with MS in the future.

New Findings

You will be informed of any significant information regarding new findings that may develop during the course of the study that may relate to your willingness to continue.

Compensation for being part of this study

You will not be compensated for taking part in this study. You may discuss with your doctor or the study staff any questions about compensation.

Compensation or medical care for illness or injury

If you have an adverse reaction (get hurt or sick) as a direct result of properly performed procedures while taking part in this study, Berlex will pay your reasonable expenses for medical treatment of the reaction to the extent that these expenses are not covered by medical, third party, or government

insurance or programs. Appropriate medical treatment will be available, if required. For more information, please contact the study doctor or a member of the study staff.

Costs of participation

There is no cost to you or your insurance company to participate in this study. Your doctor will be paid a professional fee by Berlex for his/her help in conducting this study. You will still be responsible for the cost of your usual ongoing medical care, including procedures and/or non-study medications that your study doctor or regular doctor requires during this study as part of your usual medical care. If you have questions, please ask the study doctor or a member of the study staff.

Your participation is voluntary

Your participation is strictly voluntary. You may refuse to participate at any time during the course of this study without penalty or loss of benefits to which you are otherwise entitled. If you terminate your participation, you will continue to receive standard treatment and no prejudice will be shown towards you for medical care or participation in future studies. In addition, your participation may be ended by the study doctor or Berlex without your consent.

Berlex will pay you a stipend of US$ 250 (or an equivalent amount in Canadian dollar) to cover your travel expenses and to compensate you for the completion of the above mentioned four (4) questionnaires.

Alternatives

The study does not involve a treatment for any condition. Your alternative is to not participate.

Use of the data and data protection

Information obtained from the study will be electronically stored in an anonymous manner and processed for the purposes of scientific evaluation. Selected employees or representatives from the sponsor, representatives of governmental health protection authorities from your country or other countries (e.g. the FDA or Health Canada), and Ethics Committees or Institutional Review Boards (IRB) will be allowed to look at your study data and medical records in order to check that the study is being performed properly and that you have given your full informed consent (all of these individuals are bound to secrecy.) It will not be possible to identify you from any data from the study being sent to the study sponsor or licensing authorities, or from other data about illness collected by law. If the results of the study are published, your identity will remain confidential.

Confidentiality and authorization to use and disclose medical information

This section explains how your medical records and personal health information (together, your "records") will be used and disclosed for this study. "Personal health information" is information in your medical record that could be used to identify you, such as your name, address, telephone number, photographs, date of birth, social security number, prior medical records, or the types, dates, and results of various tests and procedures.

Under federal law your study records cannot be used or disclosed by the study doctor for research purposes unless you sign this authorization. You may not participate in the study unless you sign this authorization. If you sign it, you will be agreeing to the disclosures below:

- Your signature on this form allows the study doctor to disclose your records to the sponsor, Berlex Pharmaceuticals, Inc., or the sponsor's representatives. Berlex and its representatives will use the information to review the results of the study. Your study records will be assigned a code number by the study team and you will ordinarily not be identified by name in the study records that are sent to Berlex and its consultants. Any reports or publications resulting from this study will not disclose your identity. However, Berlex and its representatives will have the right to see your complete study records, including your name, and might choose to do so. Berlex and its representatives might review or copy all of your records to assure the quality of the study or for other uses allowed by law.
- Personnel from Berlex and its representatives will be visiting the study doctor's office to check the conduct of the study, and they will be reviewing your medical records and your study records for this purpose.
- All of your records and this signed consent form might be reviewed or copied by governmental health protection authorities (e.g., FDA or Health Canada, by <<your IRB>>, or by other regulatory agencies in this country and/or in other countries. These agencies might review your records to verify information collected in this study, to verify how the study is conducted, or for other uses allowed by law.

Federal and state laws require the study doctor to protect the confidentiality of your records. However, absolute confidentiality cannot be guaranteed because of the need to disclose information as described above. In addition, after the study doctor discloses your records, those laws may no longer protect the confidentiality of the information. If you would like to know how <<your IRB>> will protect the confidentiality of your records, you can contact <<your IRB>> at the telephone number listed below. If you would like to know how the Berlex and its representatives will protect the confidentiality of your records, ask your study doctor how to obtain this information.

You have the right to see and copy your records related to the study for 15 years following the completion of the study. However, by signing this form, you agree that you might not be able to review some of your records related to the study until after the study has been completed, at which time your right of access will be restored.

This authorization has no expiration date. You can cancel this authorization at any time by giving a written notice to the study doctor. If you cancel this authorization, then the study doctor will no longer use or disclose your records unless the study doctor needs to do so in order to preserve the scientific integrity of the study.

If you do not sign this form or if you cancel this authorization, then you no longer will be able to participate in the study.

If you decide to withdraw from the study early, you do not have to cancel your authorization to use and disclose your medical information. However, if you withdraw from the study and decide to

cancel your authorization to use and disclose you medical information, the information that has already been collected in your study record may continue to be used and disclosed as described above, but only as necessary to protect the integrity of this study.

Questions and Contact

Take the time to understand this written information and the verbal explanations given by the study doctor or her/his staff. The study doctor or study staff will answer any questions you have about this study or your participation in the study. Please request an additional consultation with your study doctor if you have further questions. You can ask questions at any time. You can also consult your own family doctor. Please call if you have any questions about the study or your experience in the study. Please call right away if you have an injury, illness, or side effect.

Study doctor: <<Investigator>>
telephone number: <<000-000-0000>>
after office hours: <<000-000-0000>>

This study was reviewed by <<your IRB>>. The purpose of the IRB is to protect the rights and safety of people who volunteer to take part in research studies. You may call if you have questions about your rights as a research participant, if you have a complaint, or if you have any concerns about participating in this study.

<<Name of your IRB>>
<< IRB contact information 1>>
<< IRB contact information 2>>
<< IRB contact information 3>>

Declaration of consent for Berlex long-term follow-up study 308272

Patient No.: __ __ __ __

I have been informed by the doctor whose signature is given below, about the nature of the study , as well as what effects and possible advantages and possible side effects or risks to expect. I have had sufficient opportunity to ask questions and do not have any further questions at the moment.

My participation in this study is voluntary. I may end my participation in the study at any time without suffering disadvantages. I am not required to give reasons for my decision. During my participation I will accept and follow the instructions of the study doctor and her/his staff. I understand that I have to inform the study doctor of any changes to my health immediately.

I have been informed and agree that the patient records my be handled, stored, and transmitted electronically and that the data transferred to the study sponsor Berlex Pharmaceuticals, Inc., is entered in an anonymous way to the company's database.

I also agree that my medical data can be reviewed by local and foreign health authorities, as well as by an Institutional Review Board and by representatives of the manufacturing company and of contracted research organizations (who are bound by a secrecy statement.) This is to compare original data with those passed on to the company by my doctor in order to ensure proper documentation of the study.

By signing this form I have not given up any of my legal rights as a research participant. I understand that I will receive a copy of the Patient Information Leaflet and a signed copy of this consent form for my records.

Signature of patient:

______________________	______________	______________________
Printed name of patient	*Date*	*Signature*

In case of consent provided by a legal representative – signature of legal representative:

______________________	______________	______________________
Printed name of legal representative	*Date*	*Signature*

Signature of person explaining the study and obtaining the consent:

Printed name of person obtaining consent	*Date*	*Signature*

Declaration and signature of principle investigator

I attest that I or my representative have discussed this study with the above named participant and/or legal representative. This person had enough time to consider this information, had an opportunity to ask questions, and voluntarily agreed to participate in this study.

Signature of principle investigator:

Printed name of principle investigator	*Date*	*Signature*

Appendix 2

Patient information and informed consent form for the pharmacogenetic investigations for patients in North America

Patient Information and Informed Consent Form

Study no:	308272
Date/version of study protocol:	20 August 2004<br>Final Proposed Protocol / Version 3.0
Date/version of the patient information and informed consent form:	20 August 2004<br>Version 3.0
Title of study protocol:	A long-term follow-up of patients enrolled in the pivotal study of Betaseron® (interferon beta 1b) in relapsing-remitting multiple sclerosis
Patient no.:	___ ___ ___ ___
Manufacturer of the investigational product:	No investigational product is given in this study.

Patient Information Leaflet for an additional pharmacogenetic study within the long-term follow-up study 308272

Dear Patient,

Over the past few years there has been an enormous increase in our knowledge about the important role that genes play in a number of diseases. Genes are also known as 'family factors' or 'hereditary factors'. We also know that the genetic 'makeup' of individuals may influence the way they react to drugs, both in terms of wanted and unwanted effects of drugs.

The investigation of such relationships is a new research area known as **pharmacogenetics**. The aim of pharmacogenetics is to collect information about the relationship between genetic 'information' and the ability of a drug to influence a disease. By collecting genetic and clinical data from patients, it may be possible to discover genes that help to predict which patients are more likely to benefit from a drug, to select the best dose, and/or to avoid side effects.

You are currently participating or considering to participate in the long-term follow-up study for patients who were participating in one of the earliest studies of Betaseron® in multiple sclerosis (MS). This study is sponsored by Berlex Pharmaceuticals, Inc ("Berlex"). Beside of the investigations described in the general patient information, we are planning to explore the relation between Betaseron®, the course of disease in your case, and your genetic 'makeup'. We are inviting

all patients participating in the study 308272 also to consider their participation in these additional pharmacogenetic analyses.

> Only for patients having stopped to take Betaseron®:
> Even if you may have stopped to take Betaseron® some time ago – be it for side effects or other reasons – there might be valuable information to be gained from your participation in this additional exploration, e.g. to find out why you and others may had to stop taking the drug.

1. Why do this pharmacogenetic study?

A number of genes have already been identified that may contribute to the inheritance of MS, but so far no factors have been identified that predict the treatment response to Betaseron®. It is still unclear why Betaseron® works better in some patients than in others and why some patients suffer from more side effects than others. Neither is it known to what extent the natural course of the disease itself plays a role in this regard. The planned pharmacogenetic analyses may help to find answers to these questions.

2. What is happening in the pharmacogenetic study?

A small amount of genetic material (the so-called DNA) is needed. The genetic material will be isolated from blood cells. You will be asked to give an extra blood sample of about 12 mL (approx. one tablespoon). The blood will be taken together with the scheduled sampling for the study. It will be sent to a laboratory in a coded form (using your patient study-ID – not your name). There, the genetic material (DNA) will be stored for subsequent analyses. Before the analyses a second code will be given to the sample to exclude that somebody not authorized to do so could link the sample to you. Double coding is used also for all later analyses.

The planned pharmacogenetic analyses will examine those genes which

- may be directly related to the development and/or severity of MS, or
- may contribute to the biological effects of Betaseron® and may influence the efficacy of the or may influence the occurrence of side effects.

As new knowledge about MS and Betaseron® will become available, new genes may also be of significance for pharmacogenetic analyses. Therefore, the number of genes analyzed may vary from a few to a large number of genes depending on how much new knowledge will become available regarding your disease and Betaseron®.

Your doctor has a list of the genes under study including their scientific names. If you are interested to see that list, please, request it from your study doctor. It is possible that Berlex may decide not to perform any pharmacogenetic analyses, for example if such an analyses are not justified by the clinical outcome of the trial.

The destruction of the linking data and the subsequent anonymization will be verified by an external auditor.

Scope of results of the pharmacogenetic study

The planned analyses are basic research, so we do not expected that the results will yield any direct clinical applications. They will probably need to be verified in further studies. It is also unlikely that the results of the pharmacogenetic analyses will have any immediate impact on your treatment. Nevertheless, it is possible that additional information may be gained about the genetic characteristics that may be of importance to you individually:

- information about MS that may be relevant for the prognosis or the treatment of your condition,
- information about so far unknown or not diagnosed diseases other than MS,
- information that may determine the safety of Betaseron® and other similar drugs,
- information that determine how Betaseron® or similar drugs work in the body.

This means that genes may be found that are predictive of the future course of your disease and/or of the occurrence of other diseases. If such genes are found you will be given the option to be told about the findings by your study doctor or by any other doctor you may nominate (see also the paragraph on "Risks and Benefits"). You will be given that option of information also should findings emerge that may affect either the prognosis of patients taking Betaseron® for a very long time, new treatment options, or the safety of Betaseron®. You will be asked to consent to this option separately.

When deciding about the option to recieve that information, please consider the possible consequences and distress that such information may hold for you personally and (if you yourself decide to pass on such data) for your family, your work situation, or your health insurance plan. Please discuss this issue in detail with your study doctor.

The following procedure would be followed: We will notify the responsible study doctors of the findings without disclosing the results of any individual patient. If it can not be excluded that this information is relevant to your case, your study doctor will ask you whether or not you wish to be informed of your individual result. He will explain what clinical significance it may hold for you. Based on your own specific circumstances, you may then decide whether or not to request the information. Only then will your study doctor request your individual result from the central laboratory which will inform your designated contact person (your study doctor or another doctor, e.g., a geneticist) to fulfill your request.

If you want to be informed, it is important that you enable your study doctor to contact you, i.e., providing him with your current address and keeping this updated.

3. Scientific and commercial use of the research findings

One day Berlex may be able to patent the results of this research project and publish the results in scientific journals and at conferences. These publications will not contain any personal information traceable to you. Any patents or other intellectual property that may result from this research project will become the sole property of Berlex (and its successors, licensees, and assigns) and may be used commercially, without any restrictions. This includes, for example, the development of a test to predict reactions to Betaseron®, or the development of a new drug. This commercial interest may also be granted to other companies collaborating with Berlex. Any possible future commercial use does not imply that you will be remunerated in any way.

4. Risks and Benefits

You may know that the risk of misuse of data from genetic analyses is currently under public scrutiny. Berlex and the institutions contracted to store and analyze the pharmacogenetic samples have taken appropriate precautionary measures to prevent unauthorized access to your pharmacogenetic data. Detailed information is given in Section "11. Confidentiality of collected pharmacogenetic data and information". However, participation in research may cause a loss of privacy. Berlex will do everything possible to ensure that information obtained from this study will never be inappropriately revealed to you or any third party. For further information on confidentiality, see Section 11.

New knowledge may emerge from the pharmacogenetic analyses with respect to your disease and/or possible treatments. As said above: should you wish to be informed about such findings, it is essential that you consider the possible consequences that knowledge of such information may hold for you personally and for your family, your work situation, or your health insurance plan should you decide to pass on such data. Please discuss this issue in detail with your study doctor.

As mentioned above, the planned analyses are basic research, and it is not expected that the results will yield any direct clinical applications. For this reason, it is improbable that you will benefit directly from your participation in this pharmacogenetic study.

5. Discomfort

For you to take part in this additional investigation, we need a blood sample. We will take the sample as part of the regular blood sampling for the main study and therefore, no additional discomfort will be associated with the participation in this protocol. Otherwise, the procedure has the usual risks associated with blood sampling, e.g., possible bleeding from the puncture site, bruising, pain, blood clot formation, or local infection and inflammation in the arm where the puncture was made (very rare cases).

6. Your participation is voluntary

Your participation in this additional pharmacogenetic analyses is voluntary. If you decide against participating in the additional pharmacogenetic study there will be no consequences in terms of your continued participation in the main clinical study nor for your subsequent medical treatment.

7. Alternatives

The study does not involve a treatment for any condition. Your alternative is to not participate.

8. Compensation for being part of this study

You will not be compensated for donating your blood sample. You may discuss with your doctor or the study staff any questions about compensation.

9. Costs of participation

You and/or your insurance company will not be expected to pay for any of the procedures or tests that are required as part of this study.

10. Compensation or medical care for illness or injury

If you have an adverse reaction (get hurt or sick) as a direct result of properly performed procedures while taking part in this study, Berlex will pay your reasonable expenses for medical treatment of the reaction to the extent that these expenses are not covered by medical, third party, or government insurance or programs. Appropriate medical treatment will be available, if required. For more information, please contact the study doctor or a member of the study staff.

11. Confidentiality of collected pharmacogenetic data and information

This section explains how the information of pharmacogenetic analyses will be generated, used, and disclosed in this study.. The general patient information (a separate document given to you by your study doctor) explains how your general (non-genetic) medical records and personal health information (together, your "records") will be used and disclosed for this study. "Personal health information" is information in your medical record that could be used to identify you, such as your name, address, telephone number, photographs, date of birth, social security number, prior medical records, or the types, dates, and results of various tests and procedures.

Confidentiality is of utmost importance to Berlex. Preventative security measures against potential disclosure of your personal information have been put in place. This means that your samples and data are unlikely to be traced back to you. For the samples that are not completely anonymized these include:

- Your pharmacogenetic consent form will be archived by the study doctor separately from your other study documentation and your patient file. With the exception of the situations explained above, the results of your pharmacogenetic analyses will not be communicated to your study doctor or anybody else in the institution, and as such, will not appear in your patient file nor in any of the study data forms and will – as such – not be part of your records or personal health information.
- The information obtained from this study will be electronically stored in an anonymous manner and processed for the purposes of scientific evaluation. The law requires that you need to consent explicitly to the blood sampling and the pharmacogenetic analyses described above to participate in the pharmacogenetic study. You will also need to consent that the results can be evaluated together with your data obtained in the study 308272.
- The blood samples taken for pharmacogenetic analyses will be sent to a central laboratory in a coded form (your patient study-ID – not your name). For additional security, the pharmacogenetic test samples will be provided with a new, second code before processing. This second code will be applied at the latest before any analyses of the sample is performed and will then be used also for all pharmacogenetic data obtained.
- Samples and all double-coded results of pharmacogenetic analyses will be stored in such a way as to prevent unauthorized access to the data. At the central laboratory storing your DNA only one person (and a deputy where applicable) will have access to a list linking the second code to your original patient ID (this is the first code - not your name). This person will not have access to any of your clinical data or your patient records.
- Only a strictly limited number of persons, i.e., the person finally analyzing your pharmacogenetic data and his/her deputy will have access to your single-coded clinical data from your study records and the double-coded results of the analyses of your pharmacogenetic data. These persons are employees of Berlex or consultants of Berlex and are not part of the clinical study. They are pledged to a high degree of confidentiality.

Under federal law your study records cannot be used or disclosed for research purposes unless you sign this authorization. You may not participate in the study unless you sign this authorization. If you sign it, you will be agreeing to the disclosures below:

- Your signature on this form allows the disclosure of your pharmacogenetic information to Berlex or it's representatives in the above described double-coded manner. Your samples and the information will be assigned a double-code number by the study team and the laboratory. You will not be identified by name in the records that are sent to Berlex and its consultants. Any reports or publications resulting from this study will not disclose your identity.
- However, Berlex and its representatives will have the right to see your <u>general</u> (non-genetic) records, including your name, and might choose to do so. Personnel from Berlex and its representatives will be visiting the study doctor's office to check the conduct of the study, and they will be reviewing your medical records and your study records to assure the quality of the study or for other uses allowed by law. Berlex and its representatives might review or copy all

of your records for this purpose. Double-coding will prevent the link between your records and the pharmacogenetic information.

- All of your records – including the pharmacogenetic information and this signed consent form – might be reviewed or copied by governmental health protection authorities (e.g, FDA or Health Canada, by <<your IRB>>, or by other regulatory agencies in this country and/or in other countries. These agencies might review your records to verify information collected in this study, to verify how the study is conducted, or for other uses allowed by law.

Federal and state laws require the study doctor to protect the confidentiality of your records. However, absolute confidentiality cannot be guaranteed because of the need to disclose information as described above. In addition, after disclosure of your records, those laws may no longer protect the confidentiality of the information. If you would like to know how <<your IRB>> will protect the confidentiality of your records, you can contact <<your IRB>> at the telephone number listed below. If you would like to know how the Berlex and its representatives will protect the confidentiality of your records, ask your study doctor how to obtain this information.

You have the right to see and copy your records related to the study for 20 years following the completion of the study. As said above (see Section 4), you may want to consider the possible consequences that knowledge of such information may carry. By signing this form, you also agree that you might not be able to review some of your records related to the study until after the study has been completed, at which time your right of access will be restored.

Your authorization of the pharmacogenetic analyses will be limited in terms of time and scope. We ask you to consent to the storage and usage of your DNA sample for a period of 20 years after the end of this study (collection and verification of all data). After termination of this program, we will destroy your sample together with any resulting biological material and delete all individual results of pharmacogenetic analyses.

In addition to this limited use there is the possibility for extended use after complete anonymization of your sample. In such a case your sample and the data from the pharmacogenetic analyses will be completely anonymized and no link will be maintained to your clinical data from study 308272. The samples will only be linked to a few key data that will not allow the sample to be traced to you in any way. As a result, neither you nor anyone else will ever receive <u>individual</u> results of any further studies. The anonymization process is verified by an external auditor. You will be asked to consent to this option separately.

You can cancel this authorization at any time by giving a written notice to the study doctor. If you decide to cancel this authorization, please inform your study doctor, who will then inform the central laboratory to destroy your sample and all its isolated constituents. Berlex will then also erase all of your existing pharmacogenetic data from the data base. The only exception to this will be if your data or part of your data have already been included in a final evaluation of all patients or patient subgroups in the study. The data will not then be deleted from the larger data set, but we will exclude your data from all future analyses.

If you do not sign this form or if you cancel this authorization, then you no longer will be able to participate in the study.

If you decide to withdraw from the study early, you do not have to cancel your authorization to use and disclose your medical information. However, if you withdraw from the study and decide to cancel your authorization to use and disclose you medical information, the information that has already been collected in your study record may continue to be used and disclosed as described above, but only as necessary to protect the integrity of this study.

Questions and contact

Take the time to understand this written information and the verbal explanations and information given by the study doctor. The study doctor or study staff will answer any questions you have about this study or your participation in the study. Please request an additional consultation with your study doctor if you have further questions. You can ask questions at any time. Please call if you have any questions about the study or your experience in the study. Please call right away if you have an injury, illness, or side effect.

Study doctor: <<Investigator>>
telephone number: <<000-000-0000>>
after office hours: <<000-000-0000>>

You can also consult your own doctor. You can also ask to discuss your questions with a second doctor not otherwise involved in the conduct of the main clinical study.

This study was reviewed by <<your IRB>>. The purpose of the IRB is to protect the rights and safety of people who volunteer to take part in research studies. You may call if you have questions about your rights as a research participant, if you have a complaint, or if you have any concerns about participating in this study.

<<Your IRB>>
<< IRB contact information 1>>
<< IRB contact information 2>>
<< IRB contact information 3>>

Declaration of consent to an additional pharmacogenetic study within the long-term follow-up study 308272

To be stored seperately from the study records and patient file!

Patient No. (in study 308272): __ __ __ __

This "Declaration of Consent" is separate from and independent of the one you signed to take part in the study 308272. Please take the time to read the Patient Information Leaflet that describes the purpose of the pharmacogenetic analyses.

I have obtained my personal copy of the Patient Information Leaflet for additional pharmacogenetic analyses within the long-term follow-up study 308272. I have read and understood the Patient Information Leaflet.

I have had my questions answered after discussion with the doctor (signature below) regarding the nature, goals and course of the pharmacogenetic analyses. I was given sufficient time to make my decision. I understand that my participation in the pharmacogenetic analyses is voluntary and that my participation in the long-term follow-up study 308272 will not be affected if I decline to take part in this additional pharmacogenetic investigation.

I consent to having a blood sample of 12 mL taken for the pharmacogenetic analyses. This pharmacogenetic analyses will examine those of my genes which:

- may be directly related to the development and/or severity of MS, or
- may contribute to the biological effects of Betaseron® and may influence the efficacy of the or may influence the occurrence of side effects.

I understand that the above listing limits the scope of the pharmacogenetic analyses. However, the number of genes to be analyzed may not be limited to the genes listed in the Patient Information Leaflet. I also understand with regard to the listing above that the number of genes to be studied in the future can vary from a few to a large number of genes depending on how much new knowledge will become available regarding my disease and Betaseron® or related drugs. I also understand that the analyses may be performed outside of the United States or Canada.

I also agree to the evaluation and further use of my coded clinical data from the long-term follow-up study 308272 in connection with my coded pharmacogenetic data.

I reserve the right to withdraw my sample at any time and without suffering any disadvantage. I will not be asked to state the reasons for my decision. If I request withdrawal, my samples will be destroyed and I will be given the option to have any existing pharmacogenetic analyses data deleted. I understand that my pharmacogenetic data will not be erased once these data have already been entered into the overall evaluation of the long-term follow-up study 308272.

I understand and agree that my consent to the pharmacogenetic analyses will be limited in terms of time and scope. It will remain valid for a period of 20 years after the end of this study, i.e., after collection and verification of all data. Thereafter my pharmacogenetic data will be deleted.

I agree to the complete anonymization of my sample or part of it. In such a case the link to my clinical data as documented for the study 308272 will be deleted. The samples will only be linked to a few key data that will not allow the sample to be traced back to me in any way. As a result, neither myself nor anyone else will ever receive personal results of any further studies. The anonymization process is verified by an external auditor. I understand that, in case of withdrawal of my consent, these pharmacogenetic sample cannot be destroyed as it is impossible to identify my individual sample after complete anonymization. I understand that the limitations in terms of scope of use and time of storage do not apply to samples kept in a completely anonymized form.

This statement requires a separate consent. Please, tick and initial one of the boxes below:

- **I agree to have parts or all of my pharmacogenetic sample kept in a completely anonymized form.** ☐
- **I do NOT want parts or all of my pharmacogenetic sample to be kept in a completely anonymized form.** ☐

I understand that results from the pharmacogenetic analyses will most likely need verification in additional trials. Therefore, I agree that under normal circumstances I will not be directly informed with regard to the general or individual results of the pharmacogenetic analyses. I understand that such findings may be of relevance to me once my individual data are retrieved. Such findings may concern links between my genetic make-up and Betaseron® and/or genes relevant to the prognosis or the treatment of MS. They may also reveal the risk of another –so far non-diagnosed hereditary disease. If I want to be informed, Berlex will contact me via my study doctor. I understand that after my last visit related to this clinical trial it is my obligation to enable Berlex to contact me (e.g., by providing my study doctor with my current address).

This statement requires a separate consent. Please, tick and initial one of the boxes below:

- **I want to be informed by the study doctor of any scientifically validated results of the study in general and how these might affect me specifically.** ☐
- **I do NOT want to be informed by the study doctor of any scientifically validated results of the study in general and how these might affect me specifically.** ☐

I understand and agree that Berlex or other persons involved in this study may use any results commercially for future tests or drug development (e.g. patents).

I understand that if I would have an adverse reaction as a direct result of properly performed procedures while taking part in this study, Berlex will pay my reasonable expenses for medical treatment of the reaction to the extent that these expenses are not covered by medical, third party, or government insurance or programs.

I agree to having the research results processed electronically, stored and exchanged in coded form between authorized persons as described in the Patient Information Leaflet.

I also agree that the health protection agencies of the governments in the US or Canada (e.g., the FDA or Health Canada) or of other countries and the competent IRB may be given access to both the pharmacogenetic information and clinical data, as well as to the code linking the second code with the original code and to this signed consent form.

I understand that information identifying me will be kept as confidential as possible and described in the Patient Information Leaflet and based on applicable laws and regulations. I understand also that absolute confidentiality cannot be guaranteed because of the need to provide information as described above.

By signing this form I have not given up any of my legal rights as a research participant. I understand that I will receive a copy of the Patient Information Leaflet and a signed copy of this consent form for my records.

Signature of patient:

Printed name of patient	*Date*	*Signature*

In case of consent provided by a legal representative – signature of legal representative:

Printed name of legal representative	*Date*	*Signature*

Signature of person explaining the study and obtaining the consent:

____________________	____________________	____________________
Printed name of person obtaining consent	*Date*	*Signature*

Declaration and signature of principle investigator

I attest that I or my representative have discussed this study with the above named participant and/or legal representative. This person had enough time to consider this information, had an opportunity to ask questions, and voluntarily agreed to participate in this study.

Signature of principle investigator:

____________________	____________________	____________________
Printed name of principle investigator	*Date*	*Signature*

Appendix 3

21 CFR 50.25 – Elements of informed consent

[Code of Federal Regulations]
[Title 21, Volume 1, Parts 1 to 99]
[Revised as of April 1, 1998]
From the U.S. Government Printing Office via GPO Access
[**CITE**: **21CFR50.25**]

[Page 249-250]

TITLE 21--FOOD AND DRUGS

CHAPTER I--FOOD AND DRUG ADMINISTRATION, DEPARTMENT OF HEALTH AND HUMAN SERVICES

PART 50--PROTECTION OF HUMAN SUBJECTS--Table of Contents

Subpart B--Informed Consent of Human Subjects

§ 50.**25** Elements of informed consent.

(a) *Basic elements of informed consent.* In seeking informed consent, the following information shall be provided to each subject:

(1) A statement that the study involves research, an explanation of the purposes of the research and the expected duration of the subject's participation, a description of the procedures to be followed, and identification of any procedures which are experimental.

[[Page 250]]

(2) A description of any reasonably foreseeable risks or discomforts to the subject.

(3) A description of any benefits to the subject or to others which may reasonably be expected from the research.

(4) A disclosure of appropriate alternative procedures or courses of treatment, if any, that might be advantageous to the subject.

(5) A statement describing the extent, if any, to which confidentiality of records identifying the subject will be maintained and that notes the possibility that the Food and Drug Administration may inspect the records.

(6) For research involving more than minimal risk, an explanation as to whether any compensation and an explanation as to whether any medical treatments are available if injury occurs and, if so, what they consist of, or where further information may be obtained.

(7) An explanation of whom to contact for answers to pertinent questions about the research and research subjects' rights, and whom to contact in the event of a research-related injury to the subject.

(8) A statement that participation is voluntary, that refusal to participate will involve no penalty or loss of benefits to which the subject is otherwise entitled, and that the subject may discontinue

participation at any time without penalty or loss of benefits to which the subject is otherwise entitled.

(b) *Additional elements of informed consent.* When appropriate, one or more of the following elements of information shall also be provided to each subject:

(1) A statement that the particular treatment or procedure may involve risks to the subject (or to the embryo or fetus, if the subject is or may become pregnant) which are currently unforeseeable.

(2) Anticipated circumstances under which the subject's participation may be terminated by the investigator without regard to the subject's consent.

(3) Any additional costs to the subject that may result from participation in the research.

(4) The consequences of a subject's decision to withdraw from the research and procedures for orderly termination of participation by the subject.

(5) A statement that significant new findings developed during the course of the research which may relate to the subject's willingness to continue participation will be provided to the subject.

(6) The approximate number of subjects involved in the study.

(c) The informed consent requirements in these regulations are not intended to preempt any applicable Federal, State, or local laws which require additional information to be disclosed for informed consent to be legally effective.

(d) Nothing in these regulations is intended to limit the authority of a physician to provide emergency medical care to the extent the physician is permitted to do so under applicable Federal, State, or local law.

Appendix 4

Sample Signature Page for Investigators

Sample Signature Document

Investigators

Study Title:

A long-term follow-up of patients enrolled in the pivotal study of Betaseron® (interferon beta 1b) in relapsing-remitting multiple sclerosis:

– Protocol TB01-35686 (USA)
– Protocol TB01-35886 (Canada)

Version: Final Proposed Protocol / Version 3.0
Date: 20 August 2004

The signatories declare

- that they agree to conduct their responsibilities within the study in accordance with local law, the principles of the Declaration of Helsinki, ICH-GCP and the study protocol as presented
- that they have acquainted themselves with the results of the pharmacological and toxicological trials of the investigational product and the results of other studies carried out to date
- that they have read the study protocol and agree to it in its entirety

Name	Affiliation/Address	Signature/Date
<local principle investigator>		
<other investigators and subinvestigators, coordinators, or testers>		
<other investigators and subinvestigators, coordinators, or testers>		
<other investigators and subinvestigators, coordinators, or testers>		
<other investigators and subinvestigators, coordinators, or testers>		

Appendix 5

Betaseron®

Part 1 - Prescribing Information (USA)
Part 2 - Monograph (Canada / English-language document)
Part 3 - Monographie (Canada / French-language document)

Appendix 5

Betaseron®

Part 1 - Prescribing Information (USA)

Revision Date: October 2003

Betaseron®
Interferon beta-1b

10004938

DESCRIPTION

Betaseron® (Interferon beta-Ib) is a purified, sterile, lyophilized protein product produced by recombinant DNA techniques. Interferon beta-1b is manufactured by bacterial fermentation of a strain of *Escherichia coli* that bears a genetically engineered plasmid containing the gene for human interferon $beta_{ser17}$. The native gene was obtained from human fibroblasts and altered in a way that substitutes serine for the cysteine residue found at position 17. Interferon beta-1b has 165 amino acids and an approximate molecular weight of 18,500 daltons. It does not include the carbohydrate side chains found in the natural material.

The specific activity of Betaseron is approximately 32 million international units (IU)/mg Interferon beta-Ib. Each vial contains 0.3 mg of Interferon beta-Ib. The unit measurement is derived by comparing the antiviral activity of the product to the World Health Organization (WHO) reference standard of recombinant human interferon beta. Mannitol, USP and Albumin (Human), USP (15 mg each/vial) are added as stabilizers.

Lyophilized Betaseron is a sterile, white to off-white powder, for subcutaneous injection after reconstitution with the diluent supplied (Sodium Chloride, 0.54% Solution).

CLINICAL PHARMACOLOGY

General

Interferons (IFNs) are a family of naturally occurring proteins, produced by eukaryotic cells in response to viral infection and other biologic agents. Three major groups of interferons have been distinguished: alpha, beta, and gamma. Interferons alpha and beta comprise the Type I interferons and interferon gamma is a Type II interferon. Type I interferons have considerably overlapping but also distinct biologic activities. The bioactivities of IFNs are mediated by their interactions with specific receptors found on the surfaces of human cells. Differences in bioactivites induced by IFNs likely reflect divergences in the signal transduction process induced by IFN-receptor binding.

Biologic Activities

The mechanism of action of Interferon beta-1b in patients with multiple sclerosis is unknown. Interferon beta-1b receptor binding induces the expression of proteins that are responsible for the pleiotropic bioactivities of Interferon beta-1b. A number of these proteins (including neopterin, β_2-microglobulin, MxA protein, and IL-10) have been measured in blood fractions from Betaseron-treated patients and Betaseron-treated healthy volunteers. Immunomodulatory effects of Interferon beta-1b include the enhancement of suppressor T cell activity, reduction of pro-inflammatory cytokine production, down-regulation of antigen presentation, and inhibition of lymphocyte trafficking into the central nervous system. It is not known if these effects play an important role in the observed clinical activity of Betaseron in multiple sclerosis (MS).

Pharmacokinetics

Because serum concentrations of Interferon beta-1b are low or not detectable following subcutaneous administration of 0.25 mg or less of Betaseron, pharmacokinetic information in patients with MS receiving the recommended dose of Betaseron is not available. Following single and multiple daily subcutaneous administrations of 0.5 mg Betaseron to healthy volunteers (N=12), serum Interferon beta-1b concentrations were generally below 100 IU/mL. Peak serum Interferon beta-1b concentrations occurred between one to eight hours, with a mean peak serum interferon concentration of 40 IU/mL. Bioavailability, based on a total dose of 0.5 mg Betaseron given as two subcutaneous injections at different sites, was approximately 50%.

After intravenous administration of Betaseron (0.006 mg to 2.0 mg), similar pharmacokinetic profiles were obtained from healthy volunteers (N=12) and from patients with diseases other than MS (N=142). In patients receiving single intravenous doses up to 2.0 mg, increases in serum concentrations were dose proportional. Mean serum clearance values ranged from 9.4 $mL/min \bullet kg^{-1}$ to 28.9 $mL/min \bullet kg^{-1}$ and were independent of dose. Mean terminal elimination half-life values ranged from 8.0 minutes to 4.3 hours and mean steady-state volume of distribution values ranged from 0.25 L/kg to 2.88 L/kg. Three-times-a-week intravenous dosing for two weeks resulted in no accumulation of Interferon beta-1b in sera of patients. Pharmacokinetic parameters after single and multiple intravenous doses of Betaseron were comparable.

Following every other day subcutaneous administration of 0.25 mg Betaseron in healthy volunteers, biologic response marker levels (neopterin, β_2- microglobulin, MxA protein, and the immunosuppressive cytokine, IL-10) increased significantly above baseline six-twelve hours after the first Betaseron dose. Biologic response marker levels peaked between 40 and 124 hours and remained elevated above baseline throughout the seven-day (168-hour) study. The relationship between serum Interferon beta-1b levels or induced biologic response marker levels and the clinical effects of Interferon beta-1b in multiple sclerosis is unknown.

CLINICAL STUDIES

The safety and efficacy of Betaseron have been assessed in three multicenter trials. Study 1 evaluated Betaseron in relapsing-remitting MS (RRMS) patients and Studies 2 and 3 assessed Betaseron in secondary progressive MS (SPMS) patients.

The effectiveness of Betaseron in relapsing-remitting MS (Study 1) was evaluated in a double blind, multiclinic, randomized, parallel, placebo controlled clinical investigation of two years duration. The study enrolled MS patients, aged 18 to 50, who were ambulatory (EDSS of ≤ 5.5), exhibited a relapsing-remitting clinical course, met Poser's criteria[1] for clinically definite and/or laboratory supported definite MS and had experienced at least two exacerbations over two years preceding the trial without exacerbation in the preceding month. Patients who had received prior immunosuppressant therapy were excluded.

An exacerbation was defined as the appearance of a new clinical sign/symptom or the clinical worsening of a previous sign/symptom (one that had been stable for at least 30 days) that persisted for a minimum of 24 hours.

Patients selected for study were randomized to treatment with either placebo (N=123), 0.05 mg of Betaseron (N=125), or 0.25 mg of Betaseron (N=124) self-administered subcutaneously every other day. Outcome based on the 372 randomized patients was evaluated after two years.

Patients who required more than three 28-day courses of corticosteroids were removed from the study. Minor analgesics (acetaminophen, codeine), antidepressants, and oral baclofen were allowed ad libitum, but chronic nonsteroidal anti-inflammatory drug (NSAID) use was not allowed.

The primary protocol-defined outcome measures were 1) frequency of exacerbations per patient and 2) proportion of exacerbation free patients. A number of secondary clinical and magnetic resonance imaging (MRI) measures were also employed. All patients underwent annual T2 MRI imaging and a subset of 52 patients at one site had MRIs performed every six weeks for assessment of new or expanding lesions.

The study results are shown in **Table 1**.

TABLE 1
Two Year RRMS Study Results
Primary and Secondary Clinical Outcomes

**Efficacy Parameters**		**Treatment Groups**			**Statistical Comparisons p-value**		
**Primary End Points**		Placebo (N=123)	0.05 mg (N=125)	0.25 mg (N=124)	Placebo vs 0.05 mg	0.05 mg vs 0.25 mg	Placebo vs 0.25 mg
Annual exacerbation rate		1.31	1.14	0.90	0.005	0.113	**0.0001**
Proportion of exacerbation-free patients†		16%	18%	25%	0.609	0.288	**0.094**
Exacerbation frequency per patient	0†	20	22	29	0.151	0.077	**0.001**
	1	32	31	39			
	2	20	28	17			
	3	15	15	14			
	4	15	7	9			
	≥5	21	16	8			
**Secondary Endpoints††**							
Median number of months to first on-study exacerbation		5	6	9	0.299	0.097	**0.010**
Rate of moderate or severe exacer-bations per year		0.47	0.29	0.23	0.020	0.257	**0.001**
Mean number of moderate or severe exacerbation days per patient		44.1	33.2	19.5	0.229	0.064	**0.001**
Mean change in EDSS score‡ at endpoint		0.21	0.21	-0.07	0.995	0.108	**0.144**
Mean change in Scripps score‡‡ at endpoint		-0.53	-0.50	0.66	0.641	0.051	**0.126**
Median duration in days per exacerbation		36	33	35.5	ND	ND	**ND**
% change in mean MRI lesion area at endpoint		21.4%	9.8%	-0.9%	0.015	0.019	**0.0001**

ND Not done

† 14 exacerbation free patients (0 from placebo, six from 0.05 mg, and eight from 0.25 mg) dropped out of the study before completing six months of therapy. These patients are excluded from this analysis.

†† Sequelae and Functional Neurologic Status, both required by protocol, were not analyzed individually but are included as a function of the EDSS.

‡ EDSS scores range from 1-10, with higher scores reflecting greater disability

‡‡ Scripps neurologic rating scores range from 0-100, with smaller scores reflecting greater disability.

Of the 372 RRMS patients randomized, 72 (19%) failed to complete two full years on their assigned treatments.

Over the two-year period, there were 25 MS-related hospitalizations in the 0.25 mg Betaseron-treated group compared to 48 hospitalizations in the placebo group. In comparison, non-MS hospitalizations were evenly distributed among the groups, with 16 in the 0.25 mg Betaseron group and 15 in the placebo group. The average number of days of MS-related steroid use was 41 days in the 0.25 mg Betaseron group and 55 days in the placebo group (p=0.004).

MRI data were also analyzed for patients in this study. A frequency distribution of the observed percent changes in MRI area at the end of two years was obtained by grouping the percentages in successive intervals of equal width. Figure 1 displays a histogram of the proportions of patients, which fell into each of these intervals. The median percent change in MRI area for the 0.25 mg group was -1.1%, which was significantly smaller than the 16.5% observed for the placebo group (p=0.0001).

Distribution of Change in MRI Area
Figure 1

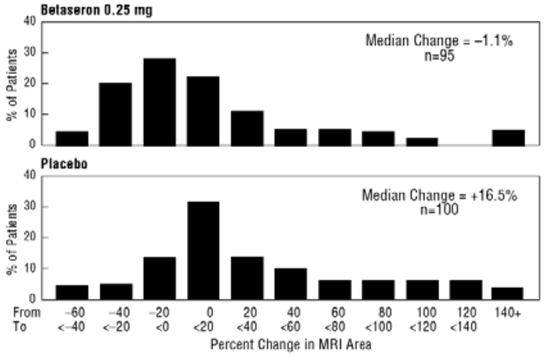

In an evaluation of frequent MRI scans (every six weeks) on 52 patients at one site, the percent of scans with new or expanding lesions was 29% in the placebo group and 6% in the 0.25 mg treatment group (p=0.006).

The exact relationship between MRI findings and clinical status of patients is unknown. Changes in lesion area often do not correlate with changes in disability progression. The prognostic significance of the MRI findings in this study has not been evaluated.

Studies 2 and 3 were multicenter, randomized, double-blind, placebo controlled trials conducted to assess the effect of Betaseron in patients with SPMS. Study 2 was conducted in Europe and Study 3 was conducted in North America. Both studies enrolled patients with clinically definite or laboratory-supported MS in the secondary progressive phase, and who had evidence of disability progression (both Study 2 and 3) or two relapses (Study 2 only) within the previous two years. Baseline Kurtzke expanded disability status scale (EDSS) scores ranged from 3.0 to 6.5.[2] Patients in Study 2 were randomized to receive Betaseron 0.25 mg (n=360) or placebo (n=358). Patients in Study 3 were randomized to Betaseron 0.25 mg (n=317), Betaseron 0.16 mg/m² of body surface area (n=314, mean assigned dose 0.30 mg), or placebo (n=308). Test agents were administered subcutaneously, every other day for three years.

The primary outcome measure was progression of disability, defined as a 1.0 point increase in the EDSS score, or a 0.5 point increase for patients with baseline EDSS ≥ 6.0. In Study 2, time to progression in EDSS was longer in the Betaseron treatment group (p=0.005), with estimated annualized rates of progression of 16% and 19% in the Betaseron and placebo groups, respectively. In Study 3, the rates of progression did not differ significantly between treatment groups, with estimated annualized rates of progression of 12%, 14%, and 12% in the Betaseron fixed dose, surface area-adjusted dose, and placebo groups, respectively.

Multiple analyses, including covariate and subset analyses based on sex, age, disease duration, clinical disease activity prior to study enrollment, MRI measures at baseline and early changes in MRI following treatment were evaluated in order to interpret the discordant study results. No demographic or disease-related factors enabled identification of a patient subset where Betaseron treatment was predictably associated with delayed progression of disability.

In Studies 2 and 3, like Study 1, a statistically significant decrease in the incidence of relapses associated with Betaseron treatment was demonstrated. In Study 2, the mean annual relapse rates were 0.42 and 0.63 in the Betaseron and placebo groups, respectively (p<0.001). In Study 3, the mean annual relapse rates were 0.16, 0.20, and 0.28, for the fixed dose, surface area-adjusted dose, and placebo groups, respectively (p<0.02).

MRI endpoints in both Study 2 and Study 3 showed lesser increases in T2 MRI lesion area and decreased number of active MRI lesions in patients in the Betaseron groups. The exact relationship between MRI findings and the clinical status of patients is unknown. Changes in MRI findings often do not correlate with changes in disability progression. The prognostic significance of the MRI findings in these studies is not known.

Safety and efficacy of treatment with Betaseron beyond three years are not known.

INDICATIONS AND USAGE

Betaseron (Interferon beta-1b) is indicated for the treatment of relapsing forms of multiple sclerosis to reduce the frequency of clinical exacerbations.

CONTRAINDICATIONS

Betaseron is contraindicated in patients with a history of hypersensitivity to natural or recombinant interferon beta, Albumin (Human), USP, or any other component of the formulation.

WARNINGS

Depression and Suicide

Betaseron (Interferon beta-1b) should be used with caution in patients with depression, a condition that is common in people with multiple sclerosis. Depression and suicide have been reported to occur with increased frequency in patients receiving interferon compounds, including Betaseron. Patients treated with Betaseron should be advised to report immediately any symptoms of depression and/or suicidal ideation to their prescribing physicians. If a patient develops depression, cessation of Betaseron therapy should be considered.

In the three randomized controlled studies there were three suicides and eight suicide attempts among the 1240 patients in the Betaseron treated groups compared to one suicide and four suicide attempts among the 789 patients in the placebo groups.

Injection Site Necrosis

Injection site necrosis (ISN) has been reported in 5% of patients in controlled clinical trials (see **ADVERSE REACTIONS**). Typically, injection site necrosis occurs within the first four months of therapy, although post-marketing reports have been received of ISN occurring over one year after initiation of therapy. Necrosis may occur at a single or multiple injection sites. The necrotic lesions are typically three cm or less in diameter, but larger areas have been reported. Generally the necrosis has extended only to subcutaneous fat. However, there are also reports of necrosis extending to and including fascia overlying muscle. In some lesions where biopsy results are available, vasculitis has been reported. For some lesions debridement and, infrequently, skin grafting have been required.

As with any open lesion, it is important to avoid infection and, if it occurs, to treat the infection. Time to healing was varied depending on the severity of the necrosis at the time treatment was begun. In most cases healing was associated with scarring.

Some patients have experienced healing of necrotic skin lesions while Betaseron therapy continued; others have not. Whether to discontinue therapy following a single site of necrosis is dependent on the extent of necrosis. For patients who continue therapy with Betaseron after injection site necrosis has occurred, Betaseron should not be administered into the affected area until it is fully healed. If multiple lesions occur, therapy should be discontinued until healing occurs.

Patient understanding and use of aseptic self-injection techniques and procedures should be periodically reevaluated, particularly if injection site necrosis has occurred.

Anaphylaxis

Anaphylaxis has been reported as a rare complication of Betaseron use. Other allergic reactions have included dyspnea, bronchospasm, tongue edema, skin rash and urticaria (see **ADVERSE REACTIONS**).

Albumin (Human), USP

This product contains albumin, a derivative of human blood. Based on effective donor screening and product manufacturing processes, it carries an extremely remote risk for transmission of viral diseases. A theoretical risk for transmission of Creutzfeldt-Jakob disease (CJD) also is considered extremely remote. No cases of transmission of viral diseases or CJD have ever been identified for albumin.

PRECAUTIONS

Information for Patients

All patients should be instructed to carefully read the supplied Betaseron Medication Guide. Patients should be cautioned not to change the dose or schedule of administration without medical consultation.

Patients should be made aware that serious adverse reactions during the use of Betaseron have been reported, including depression and suicidal ideation, injection site necrosis, and anaphylaxis (see **WARNINGS**). Patients should be advised of the symptoms of depression or suicidal ideation and be told to report them immediately to their physician. Patients should also be advised of the symptoms of allergic reactions and anaphylaxis.

Patients should be advised to promptly report any break in the skin, which may be associated with blue-black discoloration, swelling, or drainage of fluid from the injection site, prior to continuing their Betaseron therapy.

Patients should be informed that flu-like symptoms are common following initiation of therapy with Betaseron. In the controlled clinical trials, antipyretics and analgesics were permitted for relief of these symptoms. In addition, gradual dose titration during initiation of Betaseron treatment may reduce flu-like symptoms (see **DOSAGE AND ADMINISTRATION**).

Female patients should be cautioned about the abortifacient potential of Betaseron (see **PRECAUTIONS, Pregnancy - Teratogenic Effects**).

Instruction on Self-injection Technique and Procedures

Patients should be instructed in the use of aseptic technique when administering Betaseron. Appropriate instruction for reconstitution of Betaseron and self-injection should be provided, including careful review of the Betaseron Medication Guide. The first injection should be performed under the supervision of an appropriately qualified health care professional.

Patients should be cautioned against the re-use of needles or syringes and instructed in safe disposal procedures. A puncture resistant container for disposal of used needles and syringes should be supplied to the patient along with instructions for safe disposal of full containers.

Patients should be advised of the importance of rotating areas of injection with each dose, to minimize the likelihood of severe injection site reactions, including necrosis or localized infection, (see **Picking an Injection Site** section of the **Medication Guide**).

Laboratory Tests

In addition to those laboratory tests normally required for monitoring patients with multiple sclerosis, complete blood and differential white blood cell counts, platelet counts and blood chemistries, including liver function tests, are recommended at regular intervals (one, three, and six months) following introduction of Betaseron therapy, and then periodically thereafter in the absence of clinical symptoms. Thyroid function tests are recommended every six months in patients with a history of thyroid dysfunction or as clinically indicated. Patients with myelosuppression may require more intensive monitoring of complete blood cell counts, with differential and platelet counts.

Drug Interactions

No formal drug interaction studies have been conducted with Betaseron. In the placebo controlled studies in MS, corticosteroids or ACTH were administered for treatment of relapses for periods of up to 28 days in patients (N=664) receiving Betaseron.

Carcinogenesis, Mutagenesis, and Impairment of Fertility

Carcinogenesis: Interferon beta-1b has not been tested for its carcinogenic potential in animals.

Mutagenesis: Betaseron was not mutagenic when assayed for genotoxicity in the Ames bacterial test in the presence or absence of metabolic activation. Interferon beta-1b was not mutagenic to human peripheral blood lymphocytes *in vitro*, in the presence or absence of metabolic inactivation. Betaseron treatment of mouse BALBc-3T3 cells did not result in increased transformation frequency in an *in vitro* model of tumor transformation.

Impairment of fertility: Studies in normally cycling, female rhesus monkeys at doses up to 0.33 mg/kg/day (32 times the recommended human dose based on body surface area, body surface dose based on 70 kg female) had no apparent adverse effects on either menstrual cycle duration or associated hormonal profiles (progesterone and estradiol) when administered over three consecutive menstrual cycles. The validity of extrapolating doses used in animal studies to human doses is not known. Effects of Betaseron on normally cycling human females are not known.

Pregnancy - Teratogenic Effects

Pregnancy Category C: Betaseron was not teratogenic at doses up to 0.42 mg/kg/day when given to pregnant female rhesus monkeys on gestation days 20 to 70. However, a dose related abortifacient activity was observed in these monkeys when Interferon beta-1b was administered at doses ranging from 0.028 mg/kg/day to 0.42 mg/kg/day (2.8 to 40 times the recommended human dose based on body surface area comparison). The validity of extrapolating doses used in animal studies to human doses is not known. Lower doses were not studied in monkeys. Spontaneous abortions while on treatment were reported in patients (n=4) who participated in the Betaseron RRMS clinical trial. Betaseron given to rhesus monkeys on gestation days 20 to 70 did not cause teratogenic effects; however, it is not known if teratogenic effects exist in humans. There are no adequate and well-controlled studies in pregnant women. If the patient becomes pregnant or plans to become pregnant while taking Betaseron, the patient should be apprised of the potential hazard to the fetus and it should be recommended that the patient discontinue therapy.

Nursing Mothers

It is not known whether Betaseron is excreted in human milk. Because many drugs are excreted in human milk and because of the potential for serious adverse reactions in nursing infants from Betaseron, a decision should be made to either discontinue nursing or discontinue the drug, taking into account the importance of drug to the mother.

Pediatric Use

Safety and efficacy in pediatric patients have not been established.

Geriatric Use

Clinical studies of Betaseron did not include sufficient numbers of patients aged 65 and over to determine whether they respond differently than younger patients.

ADVERSE REACTIONS

In all studies, the most serious adverse reactions with Betaseron were depression, suicidal ideation and injection site necrosis (see **WARNINGS**). The incidence of depression of any severity was approximately 34% in both Betaseron-treated patients and placebo-treated patients. Anaphylaxis and other allergic reactions have been reported in patients using Betaseron (see **WARNINGS**). The most commonly reported adverse reactions were lymphopenia (lymphocytes<1500/mm^3), injection site reaction, asthenia, flu-like symptom complex, headache, and pain. The most frequently reported adverse reactions resulting in clinical intervention (e.g., discontinuation of Betaseron, adjustment in dosage, or the need for concomitant medication to treat an adverse reaction symptom) were depression, flu-like symptom complex, injection site reactions, leukopenia, increased liver enzymes, asthenia, hypertonia, and myasthenia.

Because clinical trials are conducted under widely varying conditions and over varying lengths of time, adverse reaction rates observed in the clinical trials of Betaseron cannot be directly compared to rates in clinical trials of other drugs, and may not reflect the rates observed in practice. The adverse reaction information from clinical trials does, however, provide a basis for identifying the adverse events that appear to be related to drug use and for approximating rates.

The data described below reflect exposure to Betaseron in the three placebo controlled trials of 1115 patients with MS treated with 0.25 mg or 0.16 mg/m^2, including 1041 exposed for greater than one year. The population encompassed an age range from 18 - 65 years. Sixty-five percent (65%) of the patients were female. The percentages of Caucasian, Black, Asian, and Hispanic patients were 94.0%, 4.3%, 0.2%, and 0.8%, respectively.

The safety profiles for Betaseron-treated patients with SPMS and RRMS were similar. Clinical experience with Betaseron in other populations (patients with cancer, HIV positive patients, etc.) provides additional data regarding adverse reactions; however, experience in non-MS populations may not be fully applicable to the MS population.

Injection Site Reactions

In three controlled clinical trials, injection site reactions occurred in 86% of patients receiving Betaseron with injection site necrosis in 5%. Inflammation (53%), pain (18%), hypersensitivity (3%), necrosis (5%), mass (2%), edema (3%) and non-specific reactions were significantly associated with Betaseron treatment (see **WARNINGS** and **PRECAUTIONS**). The incidence of injection site reactions tended to decrease over time, with approximately 76% of patients experiencing the event during the first three months of treatment, compared to approximately 45% at the end of the studies.

Flu-Like Symptom Complex

The rate of flu-like symptom complex was approximately 60% in the three controlled clinical trials. The incidence decreased over time, with only 10% of patients reporting flu-like symptom complex at the end of the studies. For patients who experienced a flu-like symptom complex in Study 1, the median duration was 7.5 days.

Laboratory Abnormalities

In the three clinical trials, leukopenia was reported in 18% and 5% of patients in Betaseron- and placebo-treated groups, respectively. No patients were withdrawn or dose reduced for neutropenia in Study 1. Three percent (3%) of patients in Studies 2 and 3 experienced leukopenia and were dose-reduced. Other laboratory abnormalities included SGPT greater than five times baseline value (10%), and SGOT greater than five times baseline value (3%). In Study 1, two patients were dose reduced for increased liver enzymes; one continued on treatment and one was ultimately withdrawn. In Studies 2 and 3, 1.5% of Betaseron patients were dose-reduced or interrupted treatment for increased liver enzymes. Three (0.3%) patients were withdrawn from treatment with Betaseron for any laboratory abnormality including two (0.2%) patients following dose reduction (see **PRECAUTIONS**, **Laboratory Tests**).

Menstrual Irregularities

In the three clinical trials, 82 (14%) of the 577 pre-menopausal females treated with Betaseron and 74 (18%) of the 405 pre-menopausal females treated with placebo reported menstrual disorders. One event was reported as severe, all other reports were mild to moderate severity. No patients withdrew from the studies due to menstrual irregularities.

Table 2 enumerates adverse events and laboratory abnormalities that occurred among all patients treated with 0.25 mg or 0.16 mg/m^2 Betaseron every other day for periods of up to three years in the controlled trials at an incidence that was at least 2% more than that observed in the placebo patients.

Table 2
Adverse Reactions and Laboratory Abnormalities

Adverse Reaction	Placebo (n=789)	Betaseron (n=1115)
**Body as a Whole**		
Injection site reaction	29%	85%
Asthenia	54%	61%
Flu-like symptom complex	41%	60%
Headache	48%	57%
Pain	42%	51%
Fever	22%	36%
Chills	11%	25%
Abdominal pain	13%	19%
Chest pain	7%	11%
Malaise	4%	8%
Injection site necrosis	0%	5%
**Cardiovascular System**		
Peripheral edema	12%	15%
Vasodilation	6%	8%
Hypertension	4%	7%
Peripheral vascular disorder	4%	6%
Palpitation	2%	4%
Tachycardia	2%	4%

(Continued)

Table 2
Adverse Reactions and Laboratory Abnormalities
(Continued)

Adverse Reaction	Placebo (n=789)	Betaseron (n=1115)
**Digestive System**		
Nausea	25%	27%
Constipation	18%	20%
Diarrhea	16%	19%
Dyspepsia	12%	14%
**Hemic and Lymphatic System**		
Lymphocytes < 1500/mm^3	70%	88%
ANC < 1500/mm^3	5%	14%
WBC < 3000/mm^3	4%	14%
Lymphadenopathy	4%	8%
**Metabolic and Nutritional Disorders**		
SGPT > 5 times baseline	4%	10%
SGOT > 5 times baseline	1%	3%
Weight gain	5%	7%
**Musculoskeletal System**		
Myasthenia	43%	46%
Arthralgia	29%	31%
Myalgia	16%	27%
Leg cramps	2%	4%
**Nervous System**		
Hypertonia	40%	50%
Dizziness	21%	24%
Insomnia	19%	24%
Incoordination	18%	21%
Anxiety	8%	10%
Nervousness	5%	7%
**Respiratory System**		
Dyspnea	4%	7%
**Skin and Appendages**		
Rash	18%	24%
Skin disorder	10%	12%
Sweating	6%	8%
Alopecia	2%	4%
**Urogential System**		
Urinary urgency	10%	13%
Metrorrhagia*	8%	11%
Menorrhagia*	6%	8%
Impotence**	7%	9%
Urinary frequency	5%	7%
Dysmenorrhea*	5%	7%
Prostatic disorder**	1%	3%

* pre-menopausal women
** male patients

The following adverse events have been observed during postmarketing experience with Betaseron and are classified within body system categories:

Body General: *fatal capillary leak syndrome; Cardiovascular: cardiomyopathy, deep vein thrombosis, pulmonary embolism; Digestive: hepatitis, pancreatitis, vomiting; Endocrine: hypothyroidism, hyperthyroidism, thyroid dysfunction; Hemic and Lymphatic System: anemia, thrombocytopenia; Metabolic and Nutritional: Gamma GT increase, hypocalcemia, hyperuricemia, triglyceride increase; Nervous: ataxia, confusion, convulsion, depersonalization, emotional lability, paresthesia; Respiratory: bronchospasm, pneumonia; Skin and Appendages: pruritus, skin discoloration, urticaria; Urogenital: urinary tract infection, urosepsis.

*The administration of cytokines to patients with a pre-existing monoclonal gammopathy has been associated with the development of this syndrome.

Immunogenicity

As with all therapeutic proteins, there is a potential for immunogenicity. Serum samples were monitored for the development of antibodies to Betaseron during the RRMS study. In patients receiving 0.25 mg every other day 56/124 (45%) were found to have serum neutralizing activity at one or more of the time points tested. The relationship between antibody formation and clinical safety or efficacy is not known.

These data reflect the percentage of patients whose test results were considered positive for antibodies to Betaseron using a biological neutralization assay that measures the ability of immune sera to inhibit the production of the interferon-inducible protein, MxA. Neutralization assays are highly dependent on the sensitivity and specificity of the assay. Additionally, the observed incidence of neutralizing activity in an assay may be influenced by several factors including sample handling, timing of sample collection, concomitant medications, and underlying disease. For these reasons, comparison of the incidence of antibodies to Betaseron with the incidence of antibodies to other products may be misleading.

Anaphylactic reactions have rarely been reported with the use of Betaseron.

DRUG ABUSE AND DEPENDENCE

No evidence or experience suggests that abuse or dependence occurs with Betaseron therapy; however, the risk of dependence has not been systematically evaluated.

OVERDOSAGE

Safety of doses higher than 0.25 mg every other day has not been adequately evaluated. The maximum amount of Betaseron that can be safely administered has not been determined.

DOSAGE AND ADMINISTRATION

The recommended dose of Betaseron is 0.25 mg injected subcutaneously every other day. Generally, patients should be started at 0.0625 mg (0.25 mL) subcutaneously every other day, and increased over a six-week period to 0.25 mg (1.0 mL) every other day (see **Table 3**).

Table 3. Schedule for Dose Titration

	Recommended Titration	Betaseron Dose	Volume
Weeks 1-2	25%	0.0625 mg	0.25 mL
Weeks 3-4	50%	0.125 mg	0.50 mL
Weeks 5-6	75%	0.1875 mg	0.75 mL
Week 7+	100%	0.25 mg	1.0 mL

To reconstitute lyophilized Betaseron for injection, attach the prefilled syringe containing the diluent (Sodium Chloride, 0.54% Solution) to the Betaseron vial using the vial adapter. Slowly inject 1.2 mL of diluent into the Betaseron vial. Gently swirl the vial to dissolve the drug completely; do not shake. Foaming may occur during reconstitution or if the vial is swirled or shaken too vigorously. If foaming occurs, allow the vial to sit undisturbed until the foam settles. Visually inspect the reconstituted product before use; discard the product if it contains particulate matter or is discolored. Keeping the syringe and vial adapter in place, turn the assembly over so that the vial is on top. Withdraw the appropriate dose of Betaseron solution. Remove the vial from the vial adapter before injecting Betaseron. One mL of reconstituted Betaseron solution contains 0.25 mg of Interferon beta-1b/mL.

Betaseron is intended for use under the guidance and supervision of a physician. It is recommended that physicians or qualified medical personnel train patients in the proper technique for self-administering subcutaneous injections. Patients should be advised to rotate sites for subcutaneous injections (see **PRECAUTIONS, Instruction on Self-injection Technique and Procedures**). Concurrent use of analgesics and/or antipyretics may help ameliorate flu-like symptoms on treatment days. Betaseron should be visually inspected for particulate matter and discoloration prior to administration.

Stability and Storage

The reconstituted product contains no preservative. Before reconstitution with diluent, store Betaseron at room temperature 25°C (77°F). Excursions of 15° to 30°C (59° to 86°F) are permitted. After reconstitution, if not used immediately, the product should be refrigerated and used within three hours. Avoid freezing.

HOW SUPPLIED

Betaseron is supplied as a lyophilized powder containing 0.3 mg of Interferon beta-1b, 15 mg Albumin (Human), USP, and 15 mg Mannitol, USP. Drug is packaged in a clear glass, single-use vial (3 mL capacity). A pre-filled single-use syringe containing 1.2 mL of diluent (Sodium Chloride, 0.54% solution), two alcohol prep pads, and one vial adapter with attached 27 gauge needle are included for each vial of drug. Betaseron and the diluent are for single-use only. Unused portions should be discarded. Store at room temperature.

NDC 50419-523-25 15 blister units, 0.3 mg/vial

Rx only

REFERENCES

1. Poser CM, et al. Ann Neurol 1983; 13(3): 227-231. 2. Kurtzke JF. Neurology 1983; 33(11): 1444-1452.

U.S. Patent No. 4,588,585; 4,959,314; 4,737,462; 4,530,787

Medication Guide
Betaseron® (bay-ta-seer-on)
Interferon beta-1b
(in-ter-feer-on beta-one-be)

Please read this leaflet carefully before you start to use Betaseron® and each time your prescription is refilled since there may be new information. The information in this medication guide does not take the place of talking with your doctor or healthcare professional.

What is the most important information I should know about Betaseron?

Betaseron will not cure multiple sclerosis (MS) but it has been shown to decrease the number of flare-ups of the disease. Betaseron can cause serious side effects, so before you start taking Betaseron, you should talk to your doctor about the possible benefits of Betaseron and its possible side effects to decide if Betaseron is right for you. Potential serious side effects include:

- **Depression.** Some patients treated with interferons, including Betaseron, have become seriously depressed (feeling sad). Some patients have thought about or have attempted to kill themselves. Depression (a sinking of spirits or sadness) is not uncommon in people with multiple sclerosis. However, if you are feeling noticeably sadder or helpless, or feel like hurting yourself or others, you should tell a family member or friend right away and call your doctor or health care provider as soon as possible. Your doctor may ask that you stop using Betaseron. Before starting Betaseron, you should also tell your doctor if you have ever had any mental illness, including depression, and if you take any medications for depression.
- **Risk to pregnancy.** If you become pregnant while taking Betaseron you should stop using Betaseron immediately and call your doctor. Betaseron may cause you to lose your baby (miscarry) or may cause harm to your unborn child. You and your doctor will need to decide whether the potential benefit of taking Betaseron is greater than the potential risks to your unborn child.
- **Allergic reactions.** Some patients taking Betaseron have had severe allergic reactions leading to difficulty breathing and swallowing; these reactions can happen quickly. Allergic reactions can happen after your first dose or may not happen until after you have taken Betaseron many times. Less severe allergic reactions such as rash, itching, skin bumps or swelling of the mouth and tongue can also happen. If you think you are having an allergic reaction, stop using Betaseron immediately and call your doctor.
- **Injection site problems.** Betaseron may cause redness, pain or swelling at the place where an injection was given. A few patients have developed skin infections or areas of severe skin damage (necrosis). If one of your injection sites becomes swollen and painful or the area looks infected and it doesn't heal within a few days, you should call your doctor.

What is Betaseron?

Betaseron is a type of protein called beta interferon that occurs naturally in the body. It is used to treat relapsing forms of multiple sclerosis. It will not cure your MS but may decrease the number of flare-ups of the disease. MS is a life-long disease that affects your nervous system by destroying the protective covering (myelin) that surrounds your nerve fibers. The way Betaseron works in MS is not known.

Who should not take Betaseron?

Do not take Betaseron if you:

- Have had allergic reactions such as difficulty breathing, flushing or hives to another interferon beta or to human albumin.

If you have any of the following conditions or serious medical problems, you should tell your doctor *before* taking Betaseron:

- Depression (a sinking feeling or sadness), anxiety (feeling uneasy, nervous, or fearful for no reason), or trouble sleeping
- Liver diseases
- Problems with your thyroid gland
- Blood problems such as bleeding or bruising easily and anemia (low red blood cells) or low white blood cells
- Epilepsy
- Are pregnant, breast feeding, or planning to become pregnant

You should tell your doctor if you are taking any other prescription or non-prescription medicines. This includes any vitamin or mineral supplements, or herbal products.

How should I take Betaseron?

Betaseron is given by injection under the skin (subcutaneous injection) every other day. Your injections should be approximately 48 hours (two days) apart, so it is best to take them at the same time each day, preferably in the evening just before bedtime.

You may be started on a lower dose when you first start taking Betaseron. Your doctor will tell you what dose of Betaseron to use, and that dose may change based on how your body responds. You should not change your dose without talking with your doctor.

If you miss a dose, you should take your next dose as soon as you remember or are able to take it. Your next injection should be taken about 48 hours (two days) after that dose. **Do not take Betaseron® on two consecutive days.** If you accidentally take more than your prescribed dose, or take it on two consecutive days, call your doctor right away.

You should always follow your doctor's instructions and advice about how to take this medication. If your doctor feels that you, or a family member or friend may give you the injections, then you and/or the other person should be trained by your doctor or healthcare provider in how to give an injection. Do not try to give yourself (or have another person give you) injections at home until you (or both of you) understand and are comfortable with how to prepare your dose and give the injection.

Always use a new, unopened, vial of Betaseron and syringe for each injection. Never reuse vials or syringes.

It is important that you change your injection site each time Betaseron is injected. This will lessen the chance of your having a serious skin reaction at the spot where you inject Betaseron. You should always avoid injecting Betaseron into an area of skin that is sore, reddened, infected or otherwise damaged.

At the end of this leaflet there are detailed instructions on how to prepare and give an injection of Betaseron. You should become familiar with these instructions and follow your doctor's orders before injecting Betaseron.

What should I avoid while taking Betaseron?

- **Pregnancy.** You should avoid becoming pregnant while taking Betaseron until you have talked with your doctor. Betaseron can cause you to lose your baby (miscarry).
- **Breast feeding.** You should talk to your doctor if you are breast feeding an infant. It is not known if the interferon in Betaseron can be passed to an infant in mother's milk, and it is not known whether the drug could harm the infant if it is passed to an infant.

What are the possible side effects of Betaseron?

- **Flu-like symptoms.** Most patients have flu-like symptoms (fever, chills, sweating, muscle aches and tiredness). For many patients, these symptoms will lessen or go away over time. You should talk to your doctor about whether you should take an over the counter medication for pain or fever reduction before or after taking your dose of Betaseron.
- **Skin reactions.** Soreness, redness, pain, bruising or swelling may occur at the place of injection. ***(see "What is the most important information I should know about Betaseron?")***.
- **Depression and anxiety.** Some patients taking interferons have become very depressed and/or anxious. There have been patients taking interferons who have had thoughts about killing themselves. If you feel sad or hopeless you should tell a friend or family member right away and call your doctor immediately. ***(see "What is the most important information I should know about Betaseron?")***.
- **Liver problems.** Your liver function may be affected. Symptoms of changes in your liver include yellowing of the skin and whites of the eyes and easy bruising.
- **Blood problems.** You may have a drop in the levels of infection-fighting white blood cells, red blood cells, or cells that help you form blood clots. If drops in levels are severe, they can lessen your ability to fight infections, make you feel tired or sluggish or cause you to bruise or bleed easily.
- **Thyroid problems.** Your thyroid function may change. Symptoms of changes in the function of your thyroid include feeling cold or hot much of the time or change in your weight (gain or loss) without a change in your diet or amount of exercise you are getting.
- **Allergic reaction.** Some patients have had hives, rash, skin bumps or itching while they were taking Betaseron. There is also a rare possibility that you can have a life-threatening allergic reaction. ***(see "What is the most important information I should know about Betaseron?").***

Whether you experience any of these side effects or not, you and your doctor should periodically talk about your general health. Your doctor may want to monitor you more closely and ask you to have blood tests done more frequently.

General Information About Prescription Medicines

Medicines are sometimes prescribed for purposes other than those listed in a Medication Guide. This medication has been prescribed for your particular medical condition. Do not use it for another condition or give this drug to anyone else. If you have any questions you should speak with your doctor or health care professional. You may also ask your doctor or pharmacist for a copy of the information provided to them with the product. Keep this and all drugs out of the reach of children.

Instructions for Preparing and Giving Yourself an Injection of Betaseron

1. Find a clean, flat working surface that is well-lit and collect all the supplies you will need to give yourself an injection. You will need:
   - One tray containing Betaseron. Make sure the tray contains: A pre-filled diluent syringe
   - A vial of Betaseron
   - Two (2) alcohol prep pads
   - A vial adapter with a 27 gauge needle attached (in the blister pack)
   - A puncture-resistant sealable container to dispose of used syringes/needles
2. Check the expiration date on the tray label to make sure that it has not expired. **Do not use it if the medication has expired.**
3. Wash your hands thoroughly with soap and water.
4. Open the tray by peeling off the label and take out all the contents. Make sure the blister pack containing the vial adapter is sealed. Check to make sure the rubber cap on the diluent syringe is firmly attached.
5. Turn the tray over, place the Betaseron vial in the well (vial holder) and place the prefilled diluent syringe in the U-shaped trough.

Reconstituting Betaseron

1. Remove the Betaseron vial from the well and take the cap off the vial.
2. Place the vial back into the vial holder. Use an alcohol prep pad to clean the top of the vial. Move the prep pad in one direction. Leave the alcohol prep pad on top of the vial until step 5.
3. Peel the label off the blister pack with the vial adapter in it, but do not remove vial adapter. The vial adapter is sterile; avoid touching the vial adapter.
4. Remove the alcohol prep pad from the top of the Betaseron vial. Keeping the vial adapter in the blister pack, place the adapter on top of the Betaseron vial and push down on the adapter until it pierces the rubber top of the Betaseron vial and snaps in place **(Figure 1)**. Remove the blister packaging from the vial adapter.

Figure 1

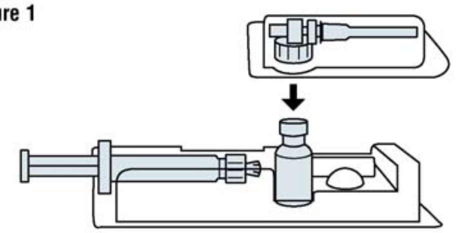

5. Remove the rubber cap from the diluent syringe using a twist and pull motion. Discard the rubber cap.
6. Keeping the syringe assembly attached to the vial, remove the vial from the tray. Be careful not to pull the vial adapter off the top of the vial.
7. Connect the syringe to the vial adapter by turning clockwise and tighten carefully. This will form the *syringe assembly* **(Figure 2)**.

Figure 2

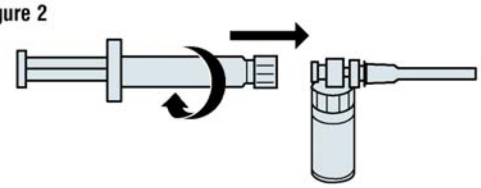

8. Slowly push the plunger of the diluent syringe all the way in. This will transfer all of the diluent in the syringe to the Betaseron vial **(Figure 3)**. The plunger may return to its original position after you release it.

Figure 3

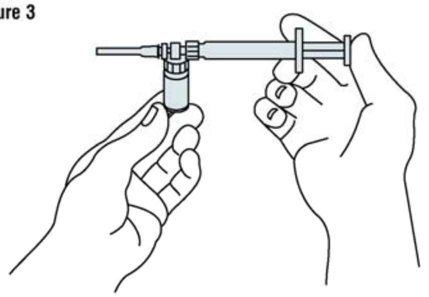

9. Gently swirl the vial to completely dissolve the white cake of Betaseron. Do not shake. Shaking can cause Betaseron to foam; even gently mixing the solution can cause foaming. If there is foam, allow the vial to sit undisturbed until the foam settles.
10. After the cake is dissolved, look closely at the solution to make sure the solution is clear and colorless and does not contain particles. If the mixture contains particles, or is discolored, do not use. Repeat the steps to prepare your dose using a new tray of Betaseron, prefilled syringe, vial adapter and alcohol prep pads. Contact Berlex at 1-800-788-1467 to obtain replacement product.

Preparing the Injection

You have completed the steps to reconstitute your Betaseron and are ready for the injection. The injection should be given immediately after mixing and allowing any foam in the solution to settle. If you must delay giving yourself the injection, you may refrigerate the solution and use within three hours of reconstitution. Do not freeze.

1. Push the plunger in and hold it there; then turn the syringe assembly so that the vial is on top. (The syringe is horizontal.) **(Figure 4)**.

Figure 4

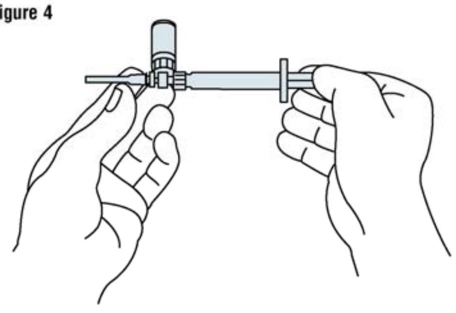

2. Slowly pull the plunger back to withdraw the entire contents of the Betaseron vial into the syringe.

   NOTE: The syringe barrel is marked with numbers from 0.25 to 1.0. If the solution in the vial cannot be drawn up to the 1.0 mark, discard the vial and syringe and start over with a new tray containing a Betaseron vial, prefilled diluent syringe, vial adapter and alcohol prep pads.
3. Turn the syringe assembly so that the needle end is pointing up. Remove any air bubbles by tapping the outer wall of the syringe with your fingers. Slowly push the plunger to the 1 mL mark on the syringe (or to the amount prescribed by your doctor).

   NOTE: If too much solution is pushed into the vial, repeat steps 1, 2, and 3.
4. Remove the vial adapter and the vial from the syringe by twisting the vial adapter as shown in Figure 5. This will remove the vial adapter and the vial from the syringe, but will leave the needle on the syringe **(Figure 5)**.

Figure 5

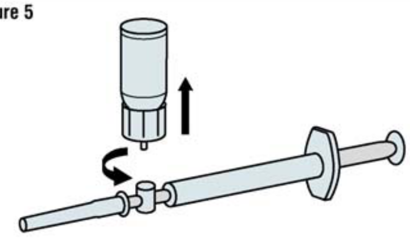

Picking an Injection Site

Betaseron (Interferon beta-1b) is injected under the skin and into the fat layer between the skin and the muscles (subcutaneous tissue). The best areas for injection are where the skin is loose and soft and away from the joints, nerves, and bones. Do not use the area near your navel or waistline. If you are very thin, use only the thigh or outer surface of the arm for injection.

You should pick a different site each time you give yourself an injection. The diagrams show different areas for giving injections. You should not choose the same area for two injections in a row. Keeping a record of your injections will help make sure you rotate your injection sites. You should decide where your injection will be given before you prepare your syringe for injection. If there are any sites that are difficult for you to reach, you can ask someone who has been trained to give injections to help you.

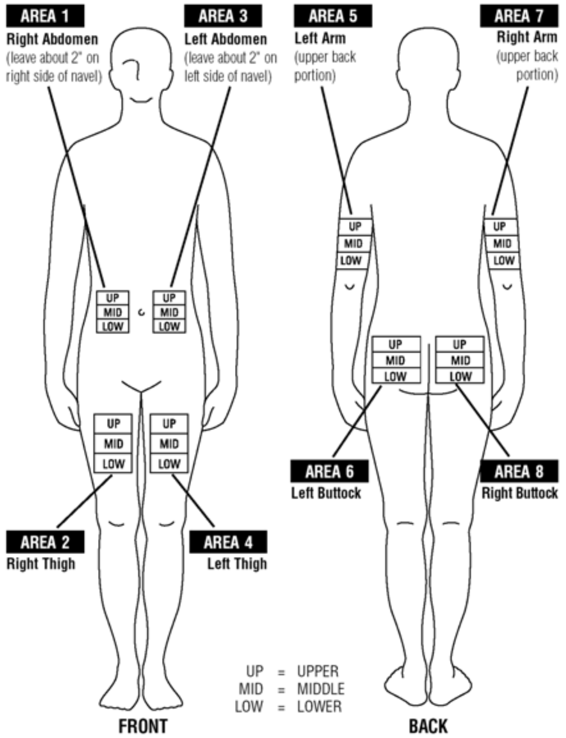

Do not inject in a site where the skin is red, bruised, infected, or scabbed, has broken open, or has lumps, bumps, or pain. Tell your doctor or healthcare provider if you find skin conditions like the ones mentioned here or any other unusual looking areas where you have been given injections.

Using a circular motion, and starting at the injection site and moving outward, clean the injection site with an alcohol wipe. Let the skin area dry before you inject the Betaseron.

Remove the cap from the needle.

Hold the syringe like a pencil or dart in one hand.

Gently pinch the skin around the site with the thumb and forefinger of the other hand.

While holding your skin, stick the needle straight into the skin at a 90° angle with a quick, firm motion. Once in your skin, slowly pull back on the plunger. If blood appears in the syringe it means that you have entered a blood vessel. Do not inject Betaseron. Withdraw the needle and repeat the steps to prepare your dose. Choose and clean a new injection site. You should not use the same syringe; discard it in your puncture-proof container.

If no blood appears, slowly push the plunger all the way in until the syringe is empty.

Remove the needle from the skin; then place a dry cotton ball or gauze pad over the injection site.

Gently massage the injection site for a few moments with the dry cotton ball or gauze pad.

Throw away the 1 mL syringe in the disposal container.

Disposing of syringes and needles

Used needles and syringes may be placed in a container made specially for disposing of used syringes and needles (called a "Sharps" container), or a hard plastic container with a screw-on cap or metal container with a plastic lid labeled *"Used Syringes"*. Do not use glass or clear plastic containers.You should always check with your healthcare provider for instructions on how to properly dispose of used vials, needles and syringes. You should follow any special state or local laws regarding the proper disposal of needles and syringes.

DO NOT throw the needle or syringe in the household trash or recycle.

Always keep the disposal container out of the reach of children.

How Should I Store Betaseron?

Betaseron should be stored at room temperature (77°F), but may be stored between 59° and 86°F. Avoid freezing.

This Medication Guide has been approved by the U.S. Food and Drug Administration

Manufactured by:
Chiron Corporation
Emeryville, CA 94608

U.S. License No. 1106

Distributed by:

Berlex Laboratories

Montville, NJ 07045

© 2003 Berlex Laboratories All rights reserved.

Printed in U.S.A. on recycled paper ♻

Part Number 10004938 Revision date 10/03 (6052800 BERLEX)

03-521-0106

Betaseron®

Part 2 - Monograph (Canada / English-language document)

Revision Date: 28 July 2003

PRODUCT MONOGRAPH

Pr BETASERON®

Interferon beta-1b

THERAPEUTIC CLASSIFICATION

Immunomodulator

Berlex Canada Inc.
334, Avro Avenue
Pointe-Claire, Quebec
H9R 5W5

Date of Revision: July 28, 2003

This Product Monograph is the exclusive property of Berlex Canada Inc. and cannot be referenced, published or copied without the written approval of Berlex Canada Inc.

PRODUCT MONOGRAPH

Pr BETASERON®

Interferon beta-1b

THERAPEUTIC CLASSIFICATION

Immunomodulator

ACTION AND CLINICAL PHARMACOLOGY

Description

BETASERON® (interferon beta-1b) is a purified, sterile, lyophilized protein product produced by recombinant DNA techniques and formulated for use by injection. Interferon beta-1b is manufactured by bacterial fermentation of a strain of *Escherichia coli* that bears a genetically engineered plasmid containing the gene for human interferon $beta_{ser17}$. The native gene was obtained from human fibroblasts and altered in a way that substitutes serine for the cysteine residue found at position 17. Interferon beta-1b is a highly purified protein that has 165 amino acids and an approximate molecular weight of 18,500 daltons. It does not include the carbohydrate side chains found in the natural material.

General

Interferons are a family of naturally occurring proteins, which have molecular weights ranging from 15,000 to 21,000 daltons. Three major classes of interferons have been identified: alpha, beta, and gamma. Interferon beta-1b, interferon alpha, and interferon gamma have overlapping yet distinct biologic activities. The activities of interferon beta are species-restricted and, therefore, the most pertinent pharmacological information on BETASERON (interferon beta-1b) is derived from studies of human cells in culture and *in vivo*.

Biologic Activities

Interferon beta-1b has been shown to possess both antiviral and immunomodulatory activities. The mechanisms by which BETASERON exerts its actions in multiple sclerosis (MS) are not clearly understood. However, it is known that the biologic response-modifying properties of interferon beta-1b are mediated through its interactions with specific cell receptors found on the surface of human cells. The binding of interferon beta-1b to these receptors induces the expression of a

number of interferon-induced gene products (e.g., 2',5'-oligoadenylate synthetase, protein kinase, and indoleamine 2,3-dioxygenase) that are believed to be the mediators of the biological actions of interferon beta-1b. A number of these interferon-induced products have been readily measured in the serum and cellular fractions of blood collected from patients treated with interferon beta-1b.

Clinical Trials

The efficacy of 8 MIU BETASERON, administered subcutaneously every other day, has been studied in one placebo-controlled clinical trial in relapsing-remitting MS patients (n=124) and a placebo-controlled trial in secondary-progressive MS patients (n=360).

1. Relapsing-Remitting MS

The effectiveness of BETASERON in relapsing-remitting MS was evaluated in a double-blind, multiclinic (11 sites: 4 in Canada and 7 in the U.S.), randomized, parallel, placebo-controlled clinical investigation of 2 years duration. The study included MS patients, aged 18 to 50, who were ambulatory (Kurtzke expanded disability status scale [EDSS] of $\leq$ 5.5), exhibited a relapsing-remitting clinical course, met Poser's criteria for clinically definite and/or laboratory supported definite MS and had experienced at least two exacerbations over 2 years preceding the trial without exacerbation in the preceding month. Patients who had received prior immunosuppressant therapy were excluded.

An exacerbation was defined, per protocol, as the appearance of a new clinical sign/symptom or the clinical worsening of a previous sign/symptom (one that had been stable for at least 30 days) that persisted for a minimum of 24 hours.

Patients selected for study were randomized to treatment with either placebo (n=123), 0.05 mg (1.6 MIU) BETASERON (n=125), or 0.25 mg (8 MIU) BETASERON (n=124) self-administered subcutaneously every other day. Outcome based on the first 372 randomized patients was evaluated after 2 years.

Patients who required more than three 28-day courses of corticosteroids were withdrawn from the study. Minor analgesics (e.g., acetaminophen), antidepressants, and oral baclofen were allowed ad libitum but chronic nonsteroidal anti-inflammatory drug (NSAID) use was not allowed.
The primary, protocol defined, outcome assessment measures were 1) frequency of exacerbations per patient and 2) proportion of exacerbation free patients. A number of secondary outcome measures were also employed as described in Table 1.

In addition to clinical measures, annual magnetic resonance imaging (MRI) was performed and quantitated for extent of disease as determined by changes in total area of lesions. In a substudy of patients (n=52) at one site, MRIs were performed every 6 weeks and quantitated for disease activity as determined by changes in size and number of lesions.

Results at the protocol designated endpoint of 2 years (see TABLE 1): In the 2-year analysis, there was a 31% reduction in annual exacerbation rate, from 1.31 in the placebo group to 0.9 in the 0.25 mg (8 MIU) group. The p-value for this difference was 0.0001. The proportion of patients free of exacerbations was 16% in the placebo group, compared with 25% in the BETASERON 0.25 mg (8 MIU) group.

Of the first 372 patients randomized, 72 (19%) failed to complete 2 full years on their assigned treatments. The reasons given for withdrawal varied with treatment assignment. Excessive use of steroids accounted for 11 of the 26 placebo withdrawals. In contrast, among the 25 withdrawals from the 0.25 mg (8 MIU) assigned group, excessive steroid use accounted for only one withdrawal. Withdrawals for adverse events attributed to study article, however, were more common among BETASERON-treated patients: 1 and 10 withdrew from the placebo and 0.25 mg (8 MIU) groups, respectively.

Over the 2-year period, there were 25 MS-related hospitalizations in the 0.25 mg (8 MIU) BETASERON-treated group compared to 48 hospitalizations in the placebo group. In comparison, non-MS hospitalizations were evenly distributed between the groups, with 16 in the 0.25 mg (8 MIU) BETASERON group and 15 in the placebo group. The average number of days of MS-related steroid use was 41 days in the 0.25 mg (8 MIU) BETASERON group and 55 days in the placebo group (p=0.004).

Table 1
2-Year Study Results
Primary and Secondary Endpoints

Efficacy Parameters		Treatment Groups			Statistical Comparisons p-value		
**Primary Clinical Endpoints**		**Placebo (n=123)**	**0.05 mg (1.6 MIU) (n =125)**	**0.25 mg (8 MIU) (n=124)**	**Placebo vs 0.05 mg (1.6 MIU)**	**0.05 mg (1.6 MIU) vs 0.25 mg (8 MIU)**	**Placebo vs 0.25 mg (8 MIU)**
**Annual exacerbation rate**		1.31	1.14	0.90	0.005	0.113	0.0001
**Proportion of exacerbation-free patients †**		16%	18%	25%	0.609	0.288	0.094
**Exacerbation frequency per patient**	**0 †**	20	22	29	0.151	0.077	0.001
	**1**	32	31	39			
	**2**	20	28	17			
	**3**	15	15	14			
	**4**	15	7	9			
	**≥5**	21	16	8			
**Secondary Endpoints ††**							
**Median number of months to first on-study exacerbation**		5	6	9	0.299	0.097	0.010
**Rate of moderate or severe exacerbations per year**		0.47	0.29	0.23	0.020	0.257	0.001
**Mean number of moderate or severe exacerbation days per patient**		44.1	33.2	19.5	0.229	0.064	0.001
**Mean change in EDSS score ‡ at endpoint**		0.21	0.21	-0.07	0.995	0.108	0.144
**Mean change in Scripps score ‡‡ at endpoint**		-0.53	-0.50	0.66	0.641	0.051	0.126
**Median duration per exacerbation (days)**		36	33	35.5	ND	ND	ND
**% change in mean MRI lesion area at endpoint**		21.4%	9.8%	-0.9%	0.015	0.019	0.0001

ND Not done.
† 14 exacerbation-free patients (0 from placebo, 6 from 0.05 mg, and 8 from 0.25 mg groups) dropped out of the study before completing 6 months of therapy. These patients are excluded from this analysis.
†† Sequelae and Functional Neurologic Status, both required by protocol, were not analyzed individually but are included as a function of the EDSS.
‡ EDSS scores range from 0-10, with higher scores reflecting greater disability.
‡‡ Scripps neurologic rating scores range from 0-100, with smaller scores reflecting greater disability.

MRI data were also analyzed for patients in this study. A frequency distribution of the observed percent changes in MRI area at the end of 2 years was obtained by grouping the percentages in successive intervals of equal width. Figure 1 displays a histogram of the proportions of patients who fell into each of these intervals. The median percent change in MRI area for the 0.25 mg (8 MIU) group was -1.1% which was significantly smaller than the 16.5% observed for the placebo group (p=0.0001).

Fifty-two patients at one site had frequent MRI scans (every 6 weeks). The percentage of scans with new or expanding lesions was 29% in the placebo group and 6% in the 0.25 mg (8 MIU) treatment group (p=0.006).

Figure 1: Distribution of Change in MRI Area

Betaseron 0.25 mg (8 million IU)

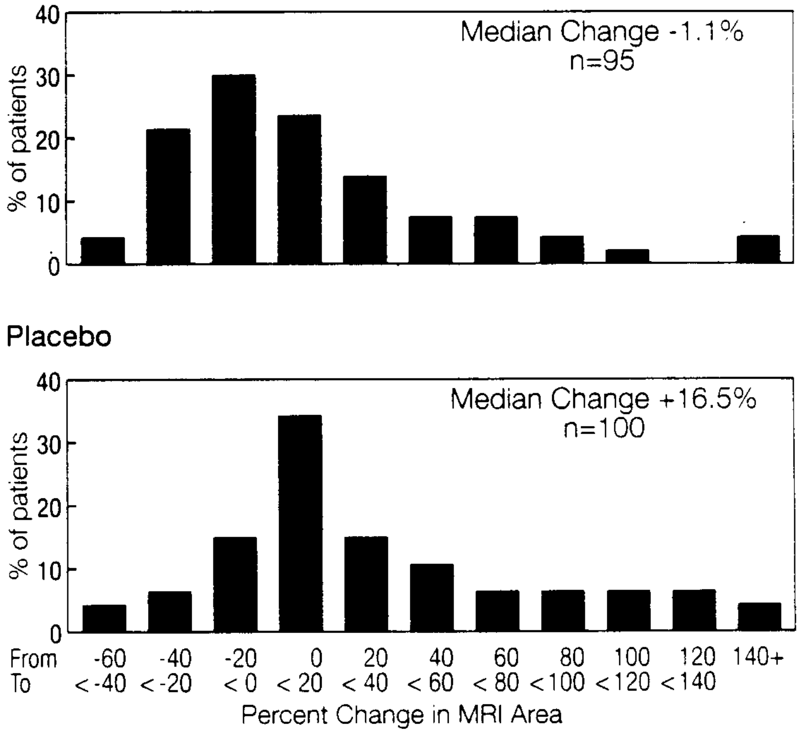

MRI scanning is viewed as a useful means to visualize changes in white matter that are believed to be a reflection of the pathologic changes that, appropriately located within the central nervous system (CNS), account for some of the signs and symptoms that typify relapsing-remitting MS. The exact relationship between MRI findings and the clinical status of patients is unknown. Changes in lesion area often do not correlate with clinical exacerbations probably because many of the lesions affect so-called "silent" regions of the CNS. Moreover, it is not clear what fraction of the

lesions seen on MRI become foci of irreversible demyelinization (i.e., classic white matter plaques). The prognostic significance of the MRI findings in this study has not been evaluated.

At the end of 2 years on assigned treatment, patients in the study had the option of continuing on treatment under blinded conditions. Approximately 80% of patients in each treatment group accepted. Although there was a trend toward patient benefit in the BETASERON groups during the third year, particularly in the 0.25 mg (8 MIU) group, there was no statistically significant difference between the BETASERON-treated vs. placebo-treated patients in exacerbation rate, or in any of the secondary endpoints described in Table 1. As noted above, in the 2-year analysis, there was a 31% reduction in exacerbation rate in the 0.25 mg (8 MIU) group, compared to placebo. The p-value for this difference was 0.0001. In the analysis of the third year alone, the difference between treatment groups was 28%. The p-value was 0.065. The lower number of patients may account for the loss of statistical significance, and lack of direct comparability among the patient groups in this extension study make the interpretation of these results difficult. The third year MRI data did not show a trend toward additional benefit in the BETASERON arm compared with the placebo arm.

Throughout the clinical trial, serum samples from patients were monitored for the development of antibodies to interferon beta-1b. In patients receiving 0.25 mg (8 MIU) BETASERON (n=124) every other day, 45% were found to have serum neutralizing activity on at least one occasion. One third had neutralizing activity confirmed by at least two consecutive positive titres. This development of neutralizing activity may be associated with a reduction in clinical efficacy, although the exact relationship between antibody formation and therapeutic efficacy is not yet known.

2. Secondary-Progressive MS

The effectiveness of BETASERON administered subcutaneously at a dose of 0.25 mg (8 MIU) every other day for 3 years was studied in a European multicenter (32 sites), randomized, double-blind, placebo-controlled trial in patients with secondary-progressive MS.

The study included patients between 18 and 55 years of age who had clinically definite or laboratory-supported definite MS for not less than one year. Disease had to be in the secondary-progressive phase and deterioration could not be exclusively related to incomplete recovery from relapses. EDSS score at study entry was between 3.0 and 6.5 and patients had to have a history of at least two clearly identified relapses, or deterioration of at least 1 EDSS point (or 0.5 points between EDSS scores of 6.0 to 7.0) within the preceding 24 months.

The primary efficacy endpoint was time to confirmed progression in disability, as determined by an increase by one point on the EDSS from baseline if the entry score was 3.0 to 5.5, or 0.5 points on the EDSS if the baseline score was 6.0 or 6.5. The increased score had to be maintained for three months before progression was confirmed. Secondary efficacy endpoints included time to becoming wheelchair-bound (EDSS 7.0) and annual relapse rate.

Although the study was designed with a treatment duration of three years, a prospectively planned interim analysis of efficacy was performed after all patients had completed 2 years in the study. This resulted in a decision by an independent Advisory Board to terminate the study early. Approximately 85% of all EDSS data for the three year study duration were available for the interim analysis of the primary endpoint. The primary analysis of efficacy was based on all patients randomized to treatment (Intent to Treat). The primary statistical method for the primary endpoint was a non-parametric analysis of covariance with stratification for centre and adjustment for baseline EDSS. Results presented below are for the dataset at study termination.

During the study, assessment of the EDSS was performed by a physician not otherwise involved in the treatment of the patient. All EDSS physicians were regularly trained to guarantee a maximally standardized assessment of the EDSS. All efforts were undertaken to maintain the blinding, e.g., standard clothing to cover injection sites was obligatory.

A total of 718 patients (358 on placebo and 360 on BETASERON) were enrolled. In both treatment groups, the proportion of female patients exceeded that of males (Placebo: 64.2% vs. 35.8%; BETASERON: 58.1% vs. 41.9%), but this difference was not statistically significant. The mean time on treatment was 886 days for placebo and 909 days for BETASERON. Eighty-eight (88) patients were lost to follow-up; the remainder were followed up until the end of study irrespective of continuation of study drug. Over the 3-year study period, treatment was discontinued prematurely by 117 (32.7%) placebo patients and 103 (29.6%) BETASERON patients. Lack of efficacy, adverse events and non-compliance were the most common reasons for ending treatment in 15.6%, 6.4% and 7.5% of the placebo group and in 7.5%, 14.2% and 3.3% of the BETASERON group, respectively. The treatment groups were well-balanced for all relevant baseline values, including EDSS at baseline, and time since evidence of secondary-progressive disease.

There was a statistically significant difference in time to confirmed progression in disability in favour of BETASERON (p=0.0046), as shown in Table 2. The delay in progression in disability became apparent after 9 months of treatment and was statistically significant from month 12 onwards. The proportion of patients with confirmed progression in disability was reduced from 60.9% in the placebo group to 51.9% in the BETASERON group (p=0.0245).

The treatment effect was consistent across all baseline EDSS levels studied; however, the relative difference in the proportion of patients having confirmed progression in disability between BETASERON and placebo-treated patients was lower for patients with study entry EDSS values of $\geq$6.0, compared to the other EDSS categories, when all patients lost to follow-up were assumed to have progressed (EDSS $\leq$3.5: 27.1%; EDSS 4.0-5.5: 17.8% and EDSS $\geq$6.0: 5.8%). When patients lost to follow-up were assumed not to have progressed, the respective values were 16.6%, 15.5% and 14.2% (shown in Table 3). Although the proportion of male patients in the BETASERON group with confirmed progression in disability was slightly higher than that of female patients, piecewise logistic regression analysis did not reveal any significant treatment by gender interaction (p=0.4335).

Kaplan-Meier plots (post-hoc analysis) of the data are shown in Figure 2. The Kaplan-Meier estimate of the percentage of patients progressing by the end of 3 years was 53.9% for placebo and 45.3% for BETASERON-treated patients.

The time to becoming wheelchair-bound (EDSS = 7.0) was also significantly prolonged (p=0.0047) and the proportion of patients becoming wheelchair-bound was reduced from 28.5% in the placebo group to 18.6% in the BETASERON group (p=0.0069).

BETASERON reduced the relapse rate by 26.3% over the entire study period (p=0.0034). The proportion of patients with moderate or severe relapses was reduced from 54.2% in the placebo group to 47.2% in the BETASERON group (p=0.0508). The mean annual rate of moderate or severe relapses was 0.44 and 0.31 in the placebo and the BETASERON group, respectively (p=0.0037).

The incidence of hospitalizations due to MS was reduced: 44.4% of placebo patients required hospitalization due to MS vs. 36.1% in the BETASERON group (p=0.0003). The number of patients with steroid courses was 73.2% and 62.5% of patients in the placebo and BETASERON group respectively (p=0.0010).

In addition to clinical measures, annual magnetic resonance imaging (MRI) was performed. All patients underwent a T2-weighted MRI scanning at baseline and yearly thereafter, while a subgroup of patients (Placebo, n = 61; BETASERON, n= 64) underwent monthly scans in months 1-6 and 19-24 in addition to the annual scans scheduled for the general study population. Results of secondary and tertiary MRI endpoints showed significant differences between treatment groups in favor of BETASERON (see Table 2). The exact relationship between MRI findings and the clinical status of patients is unknown.

Serum samples were collected throughout the study to test for the development of neutralizing antibodies (NAB) against interferon beta-1b. Analyses were performed to assess the association between NAB status (measured by an MxA neutralization assay) and treatment response as measured by clinical and MRI outcome measures. Confirmed NAB titers of 1:20, 1:100 and 1:400 were observed in 28%, 14% and 8% of patients, respectively. Despite continued therapy with BETASERON, 50% of the NAB-positive patients were found to have negative titers subsequent to the first development of confirmed quantifiable titers. The relationship between antibody formation and clinical efficacy is not known.

Table 2<br>Secondary-Progressive MS Study Results<br>Summary of Key Efficacy Endpoints			
	**Treatment Groups**		**p-value**
	**Placebo (n=358)**	**Betaseron 0.25 mg (8 MIU) (n=360)**	
**Primary Endpoints**			
Time to Confirmed Progression in Disability[1]			0.0046
**Year 1**	0.70	0.81	0.0032
**Year 2**	0.53	0.64	0.0013
**Month 33**	0.44	0.53	0.0066
**Secondary Clinical Endpoints**			
Time to becoming wheelchair-bound[2]			0.0047
**Year 1**	0.90	0.96	0.0139
**Year 2**	0.81	0.86	0.0096
**Month 36**	0.69	0.80	0.0047
Proportion of patients becoming wheelchair-bound	28.5 %	18.6%	0.0069
Mean annual relapse rate	0.57	0.42	0.0034
MRI: mean percent change in T2 lesion volume (baseline to last scan)	15.4	-2.1	<0.0001
MRI: mean number of newly active lesions (months 1-6)	10.24 (n=61)	3.57 (n=64)	<0.0001
**Tertiary endpoints**			
Proportion of patients with confirmed progression	60.9%	51.9%	0.0245
Mean endpoint EDSS	5.93	5.58	0.0065
Median time to first relapse (days)	385	644	0.0088
MRI: mean number of persistently enhancing lesions (months 1-6)	3.10 (n=61)	1.02 (n=64)	0.0009
MRI: mean number of persistently enhancing lesions (months 19-24)	3.04 (n=53)	0.36 (n=56)	0.0004

[1] Probability of remaining progression-free during the interval.
[2] Probability of not becoming wheelchair-bound during the interval.

Table 3
Proportion of Patients with Confirmed Progression Stratified by Baseline EDSS Category

Baseline EDSS	Treatment Group	ITT-A[1] Analysis			ITT-B[2] Analysis		
		% Patients with Confirmed Progression	% Difference vs Placebo		% Patients with Confirmed Progression	% Difference vs. Placebo	
			Relative	Absolute		Relative	Absolute
≤ 3.5	Placebo<br>Betaseron	55.3<br>40.3	-27.1	-15.0	44.7<br>37.3	-16.6	-7.4
4.0 – 5.5	Placebo<br>Betaseron	63.4<br>52.1	-17.8	-11.3	54.9<br>46.4	-15.5	-8.5
≥ 6.0	Placebo<br>Betaseron	60.4<br>56.9	-5.8	-3.5	55.6<br>47.7	-14.2	-7.9
Overall	Placebo<br>Betaseron	60.9<br>51.9	-14.8	-9.0	53.9<br>45.3	-16.0	-8.6

[1]ITT-A: Patients lost to follow-up were evaluated as having confirmed progression during the 3 month interval which follows the interval of follow-up loss.
[2]ITT-B: Patients lost to follow-up were evaluated as not having confirmed progression by the end of the study.

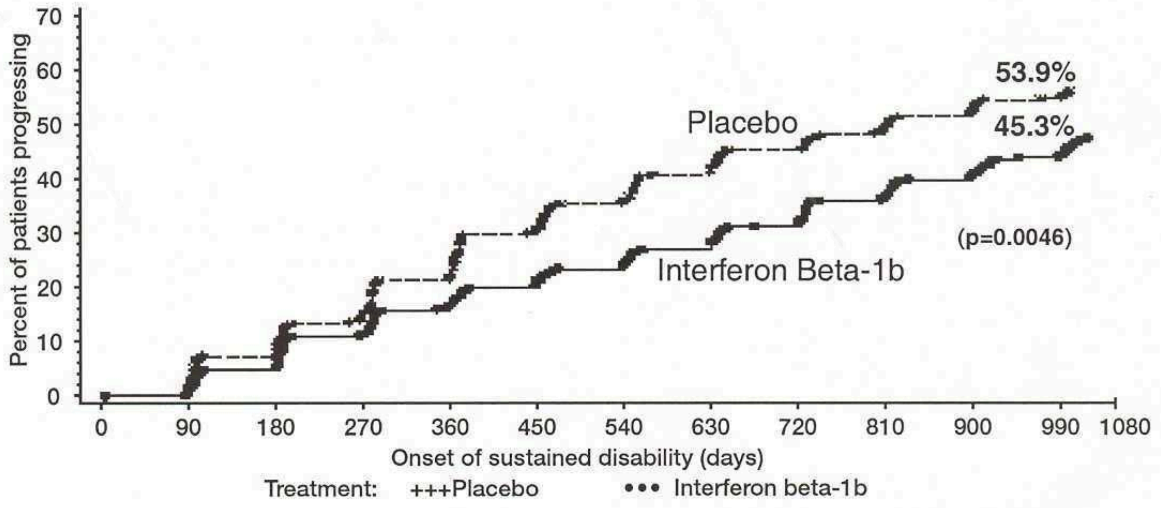

Figure 2
Onset of Progression in Disability by Time in Study
(Kaplan-Meier Methodology: Post-hoc Analysis)
Estimate of the Percentage of Patients Progressing by the End of 3 Years

Note: The p value of 0.0046 refers to the statistical difference between the overall distribution of the two curves, not to the difference in estimates at any given timepoint.

INDICATIONS AND CLINICAL USE

BETASERON (interferon beta-1b) is indicated for:

- the reduction of the frequency of clinical exacerbations in ambulatory patients with relapsing-remitting multiple sclerosis. Relapsing-remitting MS is characterized by recurrent attacks of neurologic dysfunction followed by complete or incomplete recovery.

- the slowing of progression in disability and the reduction of the frequency of clinical exacerbations in patients with secondary-progressive multiple sclerosis.

The safety and efficacy of BETASERON in primary progressive MS have not been evaluated.

CONTRAINDICATIONS

BETASERON (interferon beta-1b) is contraindicated in patients with a history of hypersensitivity to natural or recombinant interferon beta, Albumin Human USP, or any other component of the formulation.

WARNINGS

The administration of cytokines to patients with a pre-existing monoclonal gammopathy has been associated with the development of systemic capillary leak syndrome with shock-like symptoms and fatal outcome.

In the RR-MS clinical trial, one suicide and four attempted suicides were observed among 372 study patients during a 3-year period. All five patients received BETASERON (interferon beta-1b) (three in the 0.05 mg [1.6 MIU] group and two in the 0.25 mg [8.0 MIU] group). There were no attempted suicides in patients on study who did not receive BETASERON. In the SP-MS study there were 5 suicide attempts in the placebo group and 3 in the BETASERON group including one patient in each group who committed suicide. Depression and suicide have been reported to occur in patients receiving interferon alpha, a related compound. Patients treated with BETASERON should be informed that depression and suicidal ideation may be a side effect of the treatment and should report these symptoms immediately to the prescribing physician. Patients exhibiting depression should be monitored closely and cessation of therapy should be considered.

PRECAUTIONS

General

Rare cases of cardiomyopathy have been reported. If this occurs, and a relationship to BETASERON (interferon beta-1b) is suspected, treatment should be discontinued.

Rare cases of thyroid dysfunction (hyper- as well as hypothyroidism) associated with the use of BETASERON have been reported.

Symptoms of flu syndrome observed with BETASERON therapy may prove stressful to patients with severe cardiac conditions. Patients with cardiac disease such as angina, congestive heart failure or arrhythmia should be monitored closely for worsening of their clinical conditions.

Information to be Provided to the Patient

Patients should be instructed in injection techniques to assure the safe self-administration of BETASERON. (See below and the **BETASERON® INFORMATION FOR THE PATIENT** section.)

Instruction on Self-injection Technique and Procedures: It is recommended that the first injection be administered by, or under the direct supervision of, a physician. Appropriate instructions for reconstitution of BETASERON and self-injection, using aseptic techniques, should be given to the patient. A careful review of the **BETASERON® INFORMATION FOR THE PATIENT** section is also recommended.

Patients should be cautioned against the re-use of needles or syringes and instructed in safe disposal procedures. Information on how to acquire a puncture-resistant container for disposal of used needles and syringes should be given to the patient along with instructions for safe disposal of full containers.

Overall, 80% of patients in the two controlled clinical trials reported injection site reactions at one or more times during therapy. Post-marketing experience has been consistent with this finding, with infrequent reports of injection site necrosis.

The onset of injection site necrosis usually appears early in therapy with most cases reported to have occurred in the first two to three months of therapy. The number of sites where necrosis has been observed was variable.
Rarely, the area of necrosis has extended to subcutaneous fat or fascia. Response to treatment of injection site necrosis with antibiotics and/or steroids has been variable. In some of these

patients elective debridement and, less frequently, skin grafting took place to facilitate healing which could take from three to six months.

Some patients experienced healing of necrotic skin lesions while BETASERON therapy continued. In other cases new necrotic lesions developed even after therapy was discontinued.

The nature and severity of all reported reactions should be carefully assessed. Patient understanding and use of aseptic self-injection technique and procedures should be periodically reevaluated.

Flu-like symptoms are not uncommon following initiation of therapy with BETASERON. In the controlled MS clinical trials, acetaminophen was permitted for relief of fever or myalgia.

Patients should be cautioned not to change the dosage or the schedule of administration without medical consultation.

Awareness of Adverse Reactions: Patients should be advised about the common adverse events associated with the use of BETASERON, particularly, injection site reactions and the flu-like symptom complex (see **ADVERSE REACTIONS).**

Patients should be cautioned to report depression or suicidal ideation (see **WARNINGS**).

Patients should be advised about the abortifacient potential of BETASERON (see **PRECAUTIONS, Use in Pregnancy**).

Laboratory Tests

The following laboratory tests are recommended prior to initiating BETASERON therapy and at periodic intervals thereafter: thyroid function test, hemoglobin, complete and differential white blood cell counts, platelet counts and blood chemistries including liver function tests. A pregnancy test, chest roentgenogram and ECG should also be performed prior to initiating BETASERON therapy. In the controlled MS trials, patients were monitored every 3 months. The study protocol stipulated that BETASERON therapy be discontinued in the event the absolute neutrophil count fell below 750/mm^3. When the absolute neutrophil count had returned to a value greater than 750/mm^3, therapy could be restarted at a 50% reduced dose. No patients were withdrawn or dose-reduced for neutropenia or lymphopenia.

Similarly, if AST/ALT (SGOT/SGPT) levels exceeded 10 times the upper limit of normal, or if the serum bilirubin exceeded 5 times the upper limit of normal, therapy was discontinued. In each instance during the controlled MS trial, hepatic enzyme abnormalities returned to normal following discontinuation of therapy. When measurements had decreased to below these levels, therapy could be restarted at a 50% dose reduction, if clinically appropriate. Dose was reduced in two patients due to increased liver enzymes; one continued on treatment and one was ultimately withdrawn.

Drug Interactions

Interactions between BETASERON and other drugs have not been evaluated. Although studies designed to examine drug interactions have not been done, it was noted that BETASERON patients (n=180) have received corticosteroid or ACTH treatment of relapses for periods of up to 28 days.

BETASERON administered in three cancer patients over a dose range of 0.025 mg (0.8 MIU) to 2.2 mg (71 MIU) led to a dose-dependent inhibition of antipyrine elimination. The effect of alternate-day administration of 0.25 mg (8 MIU) BETASERON on drug metabolism in MS patients is unknown.

Interferons have been reported to reduce the activity of hepatic cytochrome P450-dependent enzymes in humans and animals. Caution should be exercised when BETASERON is administered in combination with agents that have a narrow therapeutic index and are largely dependent on the hepatic cytochrome P450 system for clearance.

Impairment of Fertility

Studies in female rhesus monkeys with normal menstrual cycles, at doses up to 0.33 mg (10.7 MIU)/kg/day (equivalent to 32 times the recommended human dose based on body surface area comparison) showed no apparent adverse effects on the menstrual cycle or on associated hormonal profiles (progesterone and estradiol) when administered over 3 consecutive menstrual cycles. The extrapolability of animal doses to human doses is not known. Effects of BETASERON on women with normal menstrual cycles are not known.

Use in Pregnancy

BETASERON was not teratogenic at doses up to 0.42 mg (13.3 MIU)/kg/day in rhesus monkeys, but demonstrated dose-related abortifacient activity when administered at doses ranging from 0.028 mg (0.89 MIU)/kg/day (2.8 times the recommended human dose based on body surface area comparison) to 0.42 mg (13.3 MIU)/kg/day (40 times the recommended human dose based on body surface area comparison). The extrapolability of animal doses to human doses is not known. Lower doses were not studied in monkeys. Spontaneous abortions while on treatment were reported in 4 patients who participated in the BETASERON RR-MS clinical trial, whereas there was one induced abortion in each of the placebo and BETASERON groups in the SP-MS trial. BETASERON given to rhesus monkeys on gestation days 20 to 70 did not cause teratogenic effects; however, it is not known if teratogenic effects exist in humans. There are no adequate and well-controlled studies in pregnant women. Women of childbearing potential should take reliable contraceptive measures. If the patient becomes pregnant or plans to become pregnant while taking BETASERON, the patient should discontinue therapy. It is not known if interferons alter the efficacy of oral contraceptives.

Nursing Mothers

It is not known whether BETASERON is excreted in human milk. Given that many drugs are excreted in human milk, there is a potential for serious adverse reactions in nursing infants, therefore a decision should be made whether to discontinue nursing or discontinue BETASERON treatment.

Pediatric Use

Safety and efficacy in children under 18 years of age have not been established.

Dependence Liability

No evidence or experience suggests that abuse or dependence occurs with BETASERON therapy; however, the risk of dependence has not been systematically evaluated.

ADVERSE REACTIONS

The following adverse events were observed in placebo-controlled clinical studies of BETASERON (interferon beta-1b), at the recommended dose of 0.25 mg (8 MIU), in patients with relapsing-remitting MS (n=124) and secondary-progressive MS (n=360):

1. Relapsing-remitting MS

Injection site reactions (85%) and injection site necrosis (5%) occurred after administration of BETASERON. Inflammation, pain, hypersensitivity, necrosis, and non-specific reactions were significantly associated ($p<0.05$) with the 0.25 mg (8 MIU) BETASERON-treated group, compared to placebo. Only inflammation, pain, and necrosis were reported as severe events. The incidence rate for injection site reactions was calculated over the course of 3 years. This incidence rate decreased over time, with 79% of patients experiencing the event during the first 3 months of treatment compared to 47% during the last 6 months. The median time to the first occurrence of an injection site reaction was 7 days. Patients with injection site reactions reported these events 183.7 days per year. Three patients withdrew from the 0.25 mg (8 MIU) BETASERON-treated group for injection site pain.

Flu-like symptom complex was reported in 76% of the patients treated with 0.25 mg (8 MIU) BETASERON. A patient was defined as having a flu-like symptom complex if flu-like syndrome or at least two of the following symptoms were concurrently reported: fever, chills, myalgia, malaise or sweating. Only myalgia, fever, and chills were reported as severe in more than 5% of the patients. The incidence rate for flu-like symptom complex was also calculated over the course of 3 years. The incidence rate of these events decreased over time, with 60% of patients experiencing the event during the first 3 months of treatment compared to 10% during the last 6 months. The median time to the first occurrence of flu-like symptom complex was 3.5 days and the median duration per patient was 7.5 days per year.

Laboratory abnormalities included:

- lymphocyte count < 1500/mm^3 (82%),
- ALT (SGPT) > 5 times baseline value (19%),
- absolute neutrophil count < 1500/mm^3 (18%)
  (no patients had absolute neutrophil counts < 500/mm^3),
- WBC < 3000/mm^3 (16%), and
- total bilirubin > 2.5 times baseline value (6%).

Three patients were withdrawn from treatment with 0.25 mg (8 MIU) BETASERON for abnormal

liver enzymes including one following dose reduction (see **PRECAUTIONS, Laboratory Tests).**

Twenty-one (28%) of the 76 females of childbearing age treated at 0.25 mg (8 MIU) BETASERON and 10 (13%) of the 76 females of childbearing age treated with placebo reported menstrual disorders. All reports were of mild to moderate severity and included: intermenstrual bleeding and spotting, early or delayed menses, decreased days of menstrual flow, and clotting and spotting during menstruation.

Mental disorders such as depression, anxiety, emotional lability, depersonalization, suicide attempts and confusion were observed in this study. Two patients withdrew for confusion. One suicide and four attempted suicides were also reported. It is not known whether these symptoms may be related to the underlying neurological basis of MS, to BETASERON treatment, or to a combination of both. Some similar symptoms have been noted in patients receiving interferon alpha and both interferons are thought to act through the same receptor. Patients who experience these symptoms should be monitored closely and cessation of therapy should be considered.

Additional common clinical and laboratory adverse events associated with the use of BETASERON are listed in the following paragraphs. These events occurred at an incidence of 5% or more in the 124 MS patients treated with 0.25 mg (8 MIU) BETASERON every other day for periods of up to 3 years in the controlled trial, and at an incidence that was at least twice that observed in the 123 placebo patients. Common adverse clinical and laboratory events associated with the use of BETASERON were:

- injection site reaction (85%),
- lymphocyte count < 1500/mm^3 (82%),
- ALT (SGPT) > 5 times baseline value (19%),
- absolute neutrophil count < 1500/mm^3 (18%),
- menstrual disorder (17%),
- WBC < 3000/mm^3 (16%),
- palpitation (8%),
- dyspnea (8%),
- cystitis (8%),
- hypertension (7%),
- breast pain (7%),
- tachycardia (6%),
- gastrointestinal disorders (6%),

- total bilirubin > 2.5 times baseline value (6%),
- somnolence (6%),
- laryngitis (6%),
- pelvic pain (6%),
- menorrhagia (6%),
- injection site necrosis (5%), and
- peripheral vascular disorders (5%).

A total of 277 MS patients have been treated with BETASERON in doses ranging from 0.025 mg (0.8 MIU) to 0.5 mg (16 MIU). During the first 3 years of treatment, withdrawals due to clinical adverse events or laboratory abnormalities not mentioned above included:

- fatigue (2%, 6 patients),
- cardiac arrhythmia (< 1%, 1 patient),
- allergic urticarial skin reaction to injections (< 1%, 1 patient),
- headache (< 1%, 1 patient),
- unspecified adverse events (< 1%, 1 patient), and
- "felt sick" (< 1%, 1 patient).

The table that follows enumerates adverse events and laboratory abnormalities that occurred at an incidence of 2% or more among the 124 MS patients treated with 0.25 mg (8 MIU) BETASERON every other day for periods of up to 3 years in the controlled trial and at an incidence that was at least 2% more than that observed in the 123 placebo patients. Reported adverse events have been re-classified using the standard COSTART glossary to reduce the total number of terms employed in Table 4. In the following table, terms so general as to be uninformative, and those events where a drug cause was remote have been excluded.

Table 4<br>Adverse Events and Laboratory Abnormalities		
**Adverse Event**	**Placebo<br>n=123**	**0.25 mg (8 MIU)<br>n=124**
Body as a Whole		
Injection site reaction*	37%	85%
Headache	77%	84%
Fever*	41%	59%
Flu-like symptom complex*	56%	76%
Pain	48%	52%
Asthenia*	35%	49%
Chills*	19%	46%
Abdominal pain	24%	32%
Malaise*	3%	15%
Generalized edema	6%	8%
Pelvic pain	3%	6%
Injection site necrosis*	0%	5%
Cyst	2%	4%
Necrosis	0%	2%
Suicide attempt	0%	2%
Cardiovascular System		
Migraine	7%	12%
Palpitation*	2%	8%
Hypertension	2%	7%
Tachycardia	3%	6%
Peripheral vascular disorder	2%	5%
Hemorrhage	1%	3%
Digestive System		
Diarrhea	29%	35%
Constipation	18%	24%
Vomiting	19%	21%
Gastrointestinal disorder	3%	6%
Endocrine System		
Goiter	0%	2%
Hemic and Lymphatic System		
Lymphocytes < 1500/mm^3	67%	82%
ANC < 1500/mm^3*	6%	18%
WBC < 3000/mm^3*	5%	16%
Lymphadenopathy	11%	14%
Metabolic and Nutritional Disorders		
ALT (SGPT) > 5 times baseline*	6%	19%
Glucose < 55 mg/dL	13%	15%
Total bilirubin > 2.5 times baseline	2%	6%
Urine protein > 1+	3%	5%
AST (SGOT) > 5 times baseline*	0%	4%
Weight gain	0%	4%
Weight loss	2%	4%

Table 4 (cont'd)<br>Adverse Events and Laboratory Abnormalities		
**Adverse Event**	**Placebo**<br>**n=123**	**0.25 mg (8 MIU)**<br>**n=124**
Musculoskeletal System		
Myalgia*	28%	44%
Myasthenia	10%	13%
Nervous System		
Dizziness	28%	35%
Hypertonia	24%	26%
Depression	24%	25%
Anxiety	13%	15%
Nervousness	5%	8%
Somnolence	3%	6%
Confusion	2%	4%
Speech disorder	1%	3%
Convulsion	0%	2%
Hyperkinesia	0%	2%
Amnesia	0%	2%
Respiratory System		
Sinusitis	26%	36%
Dyspnea*	2%	8%
Laryngitis	2%	6%
Skin and Appendages		
Sweating*	11%	23%
Alopecia	2%	4%
Special Senses		
Conjunctivitis	10%	12%
Abnormal vision	4%	7%
Urogenital System		
Dysmenorrhea	11%	18%
Menstrual disorder*	8%	17%
Metrorrhagia	8%	15%
Cystitis	4%	8%
Breast pain	3%	7%
Menorrhagia	3%	6%
Urinary urgency	2%	4%
Fibrocystic breast	1%	3%
Breast neoplasm	0%	2%

* significantly associated with BETASERON treatment ($p<0.05$)

It should be noted that the figures cited in Table 4 cannot be used to predict the incidence of side effects in the course of usual medical practice where patient characteristics and other factors differ from those that prevailed in the clinical trials. The cited figures do provide the prescribing physician with some basis for estimating the relative contribution of drug and nondrug factors to the side effect incidence rate in the population studied.

2. Secondary-progressive MS

The incidence of adverse events that occurred in at least 2% of patients treated with 8 MIU BETASERON or placebo for up to three years, <u>or</u> where an adverse event was reported at a frequency at least 2% higher with BETASERON than that observed for placebo-treated patients in the secondary-progressive study, is presented in Table 5. Adverse events significantly associated with BETASERON compared to placebo ($p<0.05$) are also indicated in Table 5.

Table 5<br>Incidence of Adverse Events ≥ 2% or >2% Difference (BETASERON vs. Placebo)<br>in the Secondary-Progressive MS Study		
**Adverse Event**	**Placebo**<br>**n=358**	**0.25 mg (8 MIU)**<br>**n=360**
Body as a Whole		
Asthenia	58%	63%
Flu syndrome*	40%	61%
Pain	25%	31%
Fever*	13%	40%
Back pain	24%	26%
Accidental injury	17%	14%
Chills*	7%	23%
Pain in extremity	12%	14%
Infection	11%	13%
Abdominal pain*	6%	11%
Malaise	5%	8%
Neck pain	6%	5%
Abscess*	2%	4%
Laboratory test abnormal	1%	3%
Allergic reaction	3%	2%
Chills and fever*	0%	3%
Thorax pain	2%	1%
Cardiovascular System		
Vasodilatation	4%	6%
Peripheral vascular disorder	5%	5%
Chest pain	4%	5%
Migraine	3%	4%
Hypotension	4%	2%
Hypertension*	2%	4%
Palpitation	3%	2%
Syncope	3%	2%
Hemorrhage	2%	2%
Tachycardia	1%	2%
Digestive System		
Nausea	13%	13%
Constipation	12%	12%
Diarrhea	10%	7%
Gastroenteritis	5%	6%
Vomiting	6%	4%
Dysphagia	5%	4%
Gastrointestinal disorder	5%	4%
Tooth disorder	4%	4%
Dyspepsia	4%	4%
Anorexia	2%	4%

**Table 5 (cont'd)<br>Incidence of Adverse Events ≥ 2% or >2% Difference (BETASERON vs. Placebo)<br>in the Secondary-Progressive MS Study**		
**Adverse Event**	**Placebo<br>n=358**	**0.25 mg (8 MIU)<br>n=360**
Digestive System (cont'd)		
Fecal incontinence	3%	2%
Liver function test abnormal	1%	3%
Gastritis	2%	2%
Flatulence	1%	3%
Sore throat	1%	2%
Colitis	2%	0%
Gastrointestinal pain	0%	2%
Gingivitis	0%	2%
Hemic and Lymphatic System		
Leukopenia*	5%	10%
Anemia	5%	2%
Ecchymosis	2%	1%
Lymphadenopathy	1%	3%
Injection Site		
Injection site reaction*	10%	46%
Injection site inflammation*	4%	48%
Injection site pain	5%	9%
Injection site necrosis*	0%	5%
Injection site hemorrhage	2%	2%
Metabolic and Nutritional Disorders		
Peripheral edema	7%	7%
Weight loss	3%	2%
SGPT increased	2%	2%
Hypercholesterolemia	2%	1%
Musculoskeletal System		
Myasthenia	40%	39%
Arthralgia	20%	20%
Myalgia*	9%	23%
Bone fracture (not spontaneous)	5%	3%
Muscle cramps	3%	3%
Spontaneous bone fracture	3%	3%
Arthritis	1%	2%
Joint disorder	1%	2%

Table 5 (cont'd)<br>Incidence of Adverse Events ≥ 2% or >2% Difference (BETASERON vs. Placebo)<br>in the Secondary-Progressive MS Study		
**Adverse Event**	**Placebo<br>n=358**	**0.25 mg (8 MIU)<br>n=360**
Nervous System		
Headache	41%	47%
Neuropathy	41%	38%
Paresthesia	39%	35%
Hypertonia*	31%	41%
Abnormal gait	34%	34%
Depression	31%	27%
Ataxia	23%	19%
Dizziness	14%	14%
Incoordination	13%	11%
Insomnia	8%	12%
Vertigo	12%	8%
Emotional lability	11%	8%
Paralysis	10%	8%
Somnolence	8%	8%
Tremor	9%	6%
Sweating increased	6%	6%
Neuralgia	7%	5%
Movement disorder	6%	5%
Sleep disorder	5%	6%
Anxiety	5%	6%
Hypesthesia	4%	6%
Nervousness	3%	4%
Speech disorder	5%	2%
Dysarthria	4%	2%
Spastic paralysis	1%	3%
Convulsion	2%	2%
Hyperesthesia	2%	2%
Amnesia	3%	1%
Dry mouth	2%	1%
Hemiplegia	2%	1%
Thinking abnormal	2%	1%
Myoclonus	2%	0%
Respiratory System		
Rhinitis	32%	28%
Pharyngitis	20%	16%
Bronchitis	12%	9%
Cough increased	10%	5%
Sinusitis	6%	6%
Pneumonia	5%	5%

Table 5 (cont'd)<br>Incidence of Adverse Events ≥ 2% or >2% Difference (BETASERON vs. Placebo)<br>in the Secondary-Progressive MS Study		
**Adverse Event**	**Placebo<br>n=358**	**0.25 mg (8 MIU)<br>n=360**
Respiratory System (cont'd)		
Dyspnea	2%	3%
Upper respiratory tract infection	2%	3%
Asthma	2%	1%
Voice alteration	2%	1%
Skin and Appendages		
Rash*	12%	20%
Pruritus	6%	6%
Skin disorder	4%	4%
Eczema	4%	2%
Herpes simplex	2%	3%
Alopecia	2%	2%
Acne	2%	2%
Dry skin	3%	1%
Subcutaneous hematoma	3%	1%
Breast pain	2%	1%
Herpes zoster	2%	1%
Seborrhea	2%	1%
Special Senses		
Abnormal vision	15%	11%
Amblyopia	10%	7%
Diplopia	9%	7%
Eye pain	5%	4%
Otitis media	3%	2%
Conjunctivitis	3%	2%
Eye disorder	2%	3%
Deafness	3%	1%
Optic neuritis	2%	2%
Ear disorder	2%	1%
Tinnitus	2%	1%
Urogenital System		
Urinary tract infection	25%	22%
Urinary incontinence	15%	8%
Urinary tract disorder	10%	7%
Cystitis	9%	7%
Urinary urgency	7%	8%
Menstrual disorder	13%	9%
Increased urinary frequency	5%	6%
Metrorrhagia	6%	12%
Urinary retention	6%	4%
Vaginitis	4%	3%

Table 5 (cont'd)<br>Incidence of Adverse Events ≥ 2% or >2% Difference (BETASERON vs. Placebo)<br>in the Secondary-Progressive MS Study		
**Adverse Event**	**Placebo<br>n=358**	**0.25 mg (8 MIU)<br>n=360**
Urogenital System (cont'd)		
Amenorrhea	4%	3%
Dysuria	2%	2%
Impotence	4%	7%
Menopause	4%	2%
Menorrhagia	4%	2%
Nocturia	1%	2%
Vaginal moniliasis	2%	2%
Kidney pain	2%	0%
Pyelonephritis	0%	2%
Prostatic disorder	1%	2%

*significantly associated with BETASERON treatment (p<0.05)

Seventy-four (74) patients discontinued treatment due to adverse events (23 on placebo and 51 on BETASERON). Injection site reactions were significantly associated with early termination of treatment in the BETASERON group compared to placebo (p<0.05). The highest frequency of adverse events leading to discontinuation involved the nervous system, of which depression (7 on placebo and 11 on BETASERON) was the most common.

Significantly more patients on active therapy (14.4% vs. 4.7% on placebo) had elevated ALT (SGPT) values (>5 times baseline value). Elevations were also observed in AST (SGOT) and gamma-GT values in the BETASERON group throughout the study. In the BETASERON group, most ALT (SGPT) abnormalities resolved spontaneously with continued treatment whereas some resolved upon dose reduction or temporary discontinuation of treatment.

Lymphopenia (<1500/mm^3) was observed in 90.9% of BETASERON patients compared to 74.3% of placebo patients and neutropenia (<1400/mm^3) was noted in 18.0% BETASERON and 5.1% placebo patients.

Other events observed during pre-marketing evaluation of various doses of BETASERON in 1440 patients are listed in the paragraphs that follow. Given that most of the events were observed in open and uncontrolled studies, the role of BETASERON in their causation cannot be reliably determined.

Body as a Whole: abscess, adenoma, anaphylactoid reaction, ascites, cellulitis, hernia, hydrocephalus, hypothermia, infection, peritonitis, photosensitivity, sarcoma, sepsis, and shock;

Cardiovascular System: angina pectoris, arrhythmia, atrial fibrillation, cardiomegaly, cardiac arrest, cerebral hemorrhage, cerebral ischemia, endocarditis, heart failure, hypotension, myocardial infarct, pericardial effusion, postural hypotension, pulmonary embolus, spider angioma, subarachnoid hemorrhage, syncope, thrombophlebitis, thrombosis, varicose vein, vasospasm, venous pressure increased, ventricular extrasystoles, and ventricular fibrillation;

Digestive System: aphthous stomatitis, cardiospasm, cheilitis, cholecystitis, cholelithiasis, duodenal ulcer, dry mouth, enteritis, esophagitis, fecal impaction, fecal incontinence, flatulence, gastritis, gastrointestinal hemorrhage, gingivitis, glossitis, hematemesis, hepatic neoplasia, hepatitis, hepatomegaly, ileus, increased salivation, intestinal obstruction, melena, nausea, oral leukoplakia, oral moniliasis, pancreatitis, periodontal abscess, proctitis, rectal hemorrhage, salivary gland enlargement, stomach ulcer, and tenesmus;

Endocrine System: Cushing's Syndrome, diabetes insipidus, diabetes mellitus, hypothyroidism, and inappropriate ADH;

Hemic and Lymphatic System: chronic lymphocytic leukemia, hemoglobin less than 9.4 g/100 mL, petechia, platelets less than 75,000/mm^3, and splenomegaly;

Metabolic and Nutritional Disorders: alcohol intolerance, alkaline phosphatase greater than 5 times baseline value, BUN greater than 40 mg/dL, calcium greater than 11.5 mg/dL, cyanosis, edema, glucose greater than 160 mg/dL, glycosuria, hypoglycemic reaction, hypoxia, ketosis, and thirst;

Musculoskeletal System: arthritis, arthrosis, bursitis, leg cramps, muscle atrophy, myopathy, myositis, ptosis, and tenosynovitis;

Nervous System: abnormal gait, acute brain syndrome, agitation, apathy, aphasia, ataxia, brain edema, chronic brain syndrome, coma, delirium, delusions, dementia, depersonalization, diplopia, dystonia, encephalopathy, euphoria, facial paralysis, foot drop, hallucinations, hemiplegia, hypalgesia, hyperesthesia, incoordination, intracranial hypertension, libido decreased, manic reaction, meningitis, neuralgia, neuropathy, neurosis, nystagmus, oculogyric crisis,

ophthalmoplegia, papilledema, paralysis, paranoid reaction, psychosis, reflexes decreased, stupor, subdural hematoma, torticollis, tremor and urinary retention;

Respiratory System: apnea, asthma, atelectasis, carcinoma of the lung, hemoptysis, hiccup, hyperventilation, hypoventilation, interstitial pneumonia, lung edema, pleural effusion, pneumonia, and pneumothorax;

Skin and Appendages: contact dermatitis, erythema nodosum, exfoliative dermatitis, furunculosis, hirsutism, leukoderma, lichenoid dermatitis, maculopapular rash, psoriasis, seborrhea, skin benign neoplasm, skin carcinoma, skin hypertrophy, skin necrosis, skin ulcer, urticaria, and vesiculobullous rash;

Special Senses: blepharitis, blindness, deafness, dry eyes, ear pain, iritis, keratoconjunctivitis, mydriasis, otitis externa, otitis media, parosmia, photophobia, retinitis, taste loss, taste perversion, and visual field defect;

Urogenital System: anuria, balanitis, breast engorgement, cervicitis, epididymitis, gynecomastia, hematuria, impotence, kidney calculus, kidney failure, kidney tubular disorder, leukorrhea, nephritis, nocturia, oliguria, polyuria, salpingitis, urethritis, urinary incontinence, uterine fibroids enlarged, uterine neoplasm, and vaginal hemorrhage.

DOSAGE AND ADMINISTRATION

(FOR SUBCUTANEOUS USE ONLY)

BETASERON (interferon beta-1b) should only be prescribed by (or following consultation with) clinicians who are experienced in the diagnosis and management of multiple sclerosis.

The recommended dose of BETASERON for both relapsing-remitting and secondary-progressive MS patients is 0.25 mg (8 MIU) injected subcutaneously every other day. Limited data regarding the activity of a lower dose in relapsing-remitting MS patients are presented above (see **ACTION AND CLINICAL PHARMACOLOGY, Clinical Trials**).

In the secondary-progressive MS study, patients initiated treatment with half the dose (4 MIU s.c. every other day) for a period of 2 weeks prior to escalating to the recommended dose of 8 MIU

(s.c. every other day).

Efficacy of treatment for longer than 2 years has not been substantially demonstrated in relapsing-remitting multiple sclerosis. For secondary-progressive multiple sclerosis, safety and efficacy data beyond 3 years are not available.

To reconstitute lyophilized BETASERON for injection, use the vial adapter to inject the entire contents of the pre-filled diluent syringe containing Sodium Chloride, 0.54% Solution into the BETASERON vial. Gently swirl the vial of BETASERON to dissolve the drug completely; do not shake. Inspect the reconstituted product visually and discard the product before use if it contains particulate matter or is discolored. After reconstitution with diluent, each mL of solution contains 0.25 mg (8 MIU) interferon beta-1b, 13 mg Albumin Human USP and 13 mg Mannitol USP.

Withdraw 1 mL of reconstituted solution from the vial back into the syringe, fitted with a 27-gauge ½-inch needle, and inject the solution subcutaneously. Sites for self-injection include abdomen, buttocks and thighs. All components are suitable for single use only; unused portions should be discarded. (See **BETASERON® [interferon beta-1b] INFORMATION FOR THE PATIENT** section for **SELF-INJECTION PROCEDURE**.)

PHARMACEUTICAL INFORMATION

Common Name: interferon beta-1b (USAN)

Molecular Weight: approximately 18,500 daltons

Physical Form: sterile, lyophilized powder

Composition (each vial contains): 0.3 mg (9.6 MIU) interferon beta-1b
15 mg Albumin Human, USP
15 mg Mannitol, USP

The specific activity of BETASERON is approximately 32 million international units per mg (MIU/mg) interferon beta-1b. The unit measurement is derived by comparing the antiviral activity of the product to the World Health Organization (WHO) reference standard of recombinant human interferon beta. Prior to 1993, a different analytical standard was used to determine potency. It assigned 54 million IU to 0.3 mg interferon beta-1b.

Stability (before reconstitution): Store at room temperature between 15°C and 30°C (59°F and 86°F). Avoid freezing. Do not use beyond the expiration date indicated on the labels of the Betaseron vial and the pre-filled diluent syringe.

Stability (after reconstitution): The reconstituted product contains no preservative. If not used immediately, store under refrigeration between 2°C and 8°C (36°F and 46°F) **and use within 3 hours of reconstitution.** Avoid freezing.

AVAILABILITY OF DOSAGE FORMS

BETASERON (interferon beta-1b) is presented in single-use vials of lyophilized powder containing 0.3 mg (9.6 MIU) interferon beta-1b, 15 mg Albumin Human USP, and 15 mg Mannitol USP. Betaseron is supplied in cartons containing 15 single-use packs. Each single-use pack contains the necessary components to prepare and inject a single dose of Betaseron: 1 vial of medication, 1 pre-filled diluent syringe (1.2 mL of Sodium Chloride 0.54% solution per syringe), 1 vial adapter with attached 27 gauge ½ " needle and 3 alcohol wipes.

INFORMATION FOR THE PATIENT

BETASERON (interferon beta-1b) is intended for use under the guidance and supervision of a physician. Your physician or his/her delegate should instruct you in the preparation and self-injection technique of BETASERON. Do not begin your BETASERON treatment without training.

BETASERON should be used as prescribed by your physician. However, if you miss a dose, take it as soon as you remember. While using BETASERON, please keep in mind the following facts:

- **Before reconstitution:** Store BETASERON at room temperature, between 15°C and 30°C (59°F and 86°F). Do not freeze.

  After reconstitution: If not used immediately, the product must be refrigerated and **used within three hours.** Do not freeze.

- Keep syringes and needles away from children. Do not reuse needles or syringes. Discard used syringes and needles in a syringe disposal unit.

- **Women:** BETASERON should not be used during pregnancy or if you are trying to become pregnant. While using BETASERON, women of childbearing age should use birth control measures. If you wish to become pregnant while using BETASERON, discuss the matter with your doctor. If you do become pregnant, you should discontinue treatment and contact your doctor immediately.

- Injection site reactions are common. They include redness, pain, swelling, and discoloration. Less frequently, injection site necrosis (skin breakdown and tissue destruction) has been observed. To minimize the chances of a reaction, change injection areas every time you inject yourself and wait at least one week before reusing an area. Do not make an injection into skin that is tender, red or hard. Do not use any areas where you

feel lumps, depressions, pain or discoloration; talk to your doctor or nurse about anything you find. If you experience a break in the skin or drainage of fluid from the injection site, consult your doctor.

■ Flu-like symptoms are also common. They include fever, chills, sweating, fatigue and muscle aches. Taking BETASERON at night may help lessen the impact of flu-like symptoms.

■ Depression, including suicide attempts, has been reported by patients. If you experience such symptoms, contact your doctor promptly.

■ As with any prescription medication, side effects related to therapy can occur. Consult your doctor if you have any problems, whether or not you think they may be related to BETASERON.

SELF-INJECTION PROCEDURE

SAFETY TIPS

- Use only the supplies that come with your Betaseron package.
- Use only the diluent from the pre-filled syringe.
- Wash your hands thoroughly with soap and water before starting.
- Keep the items sterile. Do not touch the needle, the piercing spike of the vial adapter or the top of the cleaned vial.
- Make sure none of the items in your package have been opened or are damaged.
- Do not reuse opened materials. Throw away any unused portions of Betaseron and diluent.
- Throw away used syringes and needles in the proper disposal container.

STEP 1: CHOOSING AN INJECTION SITE

BETASERON should be injected into subcutaneous tissue (under the skin, between the fat layer and the muscles beneath). The best areas for injection are loose and soft, away from joints.

■ **Choose an injection site from the following areas:**

- Abdomen, above the waistline (at least 5 cm on either side of the navel)
- Right thigh (at least 5 cm above the knee and 5 cm below the groin)

- Left thigh (at least 5 cm above the knee and 5 cm below the groin)
- Left buttock (upper, outer portion)
- Right buttock (upper, outer portion)

■ Change injection areas every time you inject yourself. Give the site time to recover from the last injection. This will help prevent injection site reactions.

■ Wait at least one week before reusing an area.

■ Do not use any areas where you feel lumps, depressions, pain or discoloration; talk to your doctor or nurse about anything you find.

■ Keep a record of when and where you are giving yourself injections. Use the BETASERON diary in your training kit.

STEP 2: INITIAL PREPARATION

1. Place the Betaseron single-use pack on a clean, flat surface in a well-lighted area. Ensure the pack contains:
   - Vial of Betaseron
   - Pre-filled diluent syringe
   - Three (3) alcohol wipes
   - Vial adapter with attached 27-gauge, ½ “ needle in blister pack

2. Wash your hands thoroughly with soap and water.

3. Open the pack by peeling off the label.

4. Take out all the contents.

   NOTE: *Be sure the vial adapter blister pack is sealed and the rubber cap is firmly attached to the diluent syringe.*

5. Check the expiry date on the Betaseron vial and the pre-filled diluent syringe.

6. Turn the single-use pack over, place the Betaseron vial in the well (vial holder) in the centre of the pack and place the pre-filled diluent syringe in the U-shaped trough.

STEP 3: RECONSTITUTING BETASERON

1. **Remove** the Betaseron vial from the vial holder and **take** the protective cap off the vial.

2. **Place** the vial back into the vial holder.

3. Use an alcohol wipe to **clean** the top of the vial. Move the wipe in one direction.
NOTE: *Leave the alcohol wipe on top of the vial until step 5.*

4. **Peel** off the vial adapter blister pack label but do not remove the vial adapter.
NOTE: *Be sure to avoid touching the vial adapter, in order to maintain its sterility.*

5. **Remove** the alcohol wipe on top of the Betaseron vial. **Place** the vial adapter (still in the blister packaging) on top of the Betaseron vial by pushing it until it pierces the rubber top of the Betaseron vial and snaps in place (Figure 1). Remove the blister packaging from the vial adapter.

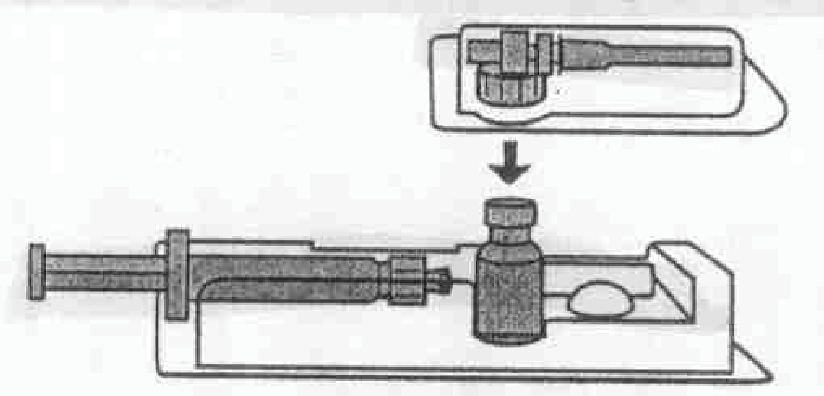

Figure 1

6. **Remove** the rubber cap from the diluent syringe with a twist and pull motion. Discard the rubber cap.

7. **Connect** the syringe with the vial adapter by turning clockwise and tighten carefully. This

will form the syringe assembly (Figure 2).

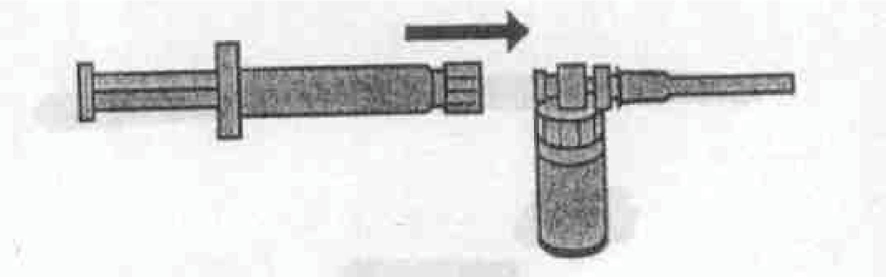

Figure 2

8. It is important to **slowly** **push** the plunger of the diluent syringe all the way in. This will transfer all of the diluent into the Betaseron vial (Figure 3). The plunger may return to its original position after release.

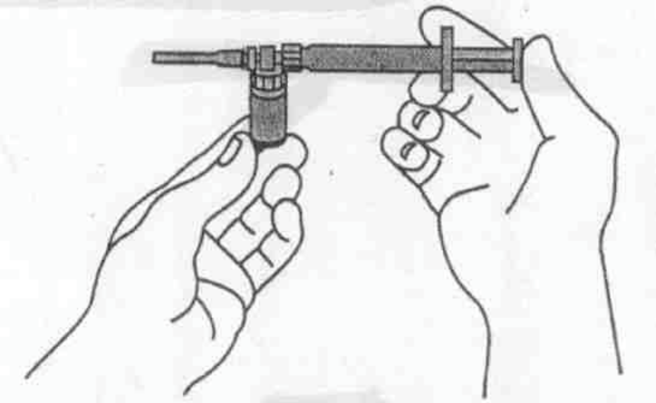

Figure 3

9. With the syringe assembly attached, **swirl** the vial **gently** to completely dissolve the white cake of Betaseron. **(DO NOT SHAKE.)**

10. **Look** closely at the Betaseron solution for particles. It should be clear.

NOTE: *If the mixture contains particles or is discoloured, discard it and start again. Foaming may occur during reconstitution, or if it is swirled or shaken too vigorously. If so, allow the vial to sit undisturbed until the foam settles.*

STEP 4: PREPARING THE INJECTION

1. **Push** the plunger in and hold it there, then turn the assembly upside down (i.e., 180 degrees) so that the vial is on top. The syringe remains horizontal (Figure 4).

2. Slowly **pull** the plunger back to withdraw the entire contents of the Betaseron vial into the syringe (Figure 4).

NOTE: *If 1 mL of clear solution cannot be withdrawn from the vial, discard the vial and syringe and start over.*

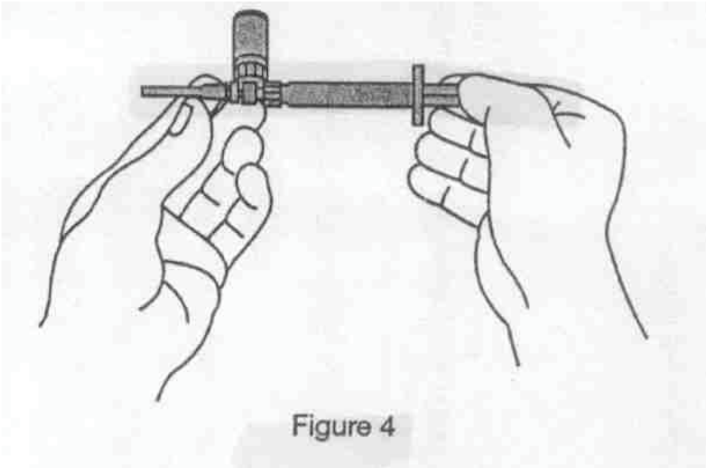

Figure 4

3. **Turn** the syringe assembly so that the needle end is pointing up. Remove any air bubbles (you may **gently** tap the outer wall of the syringe to free any bubbles) by slowly pushing the plunger to the 1 mL mark on the syringe.

NOTE: *If too much solution is expelled into the vial, repeat steps 1, 2 and 3.*

4. **Remove** the vial adapter and the vial from the syringe by twisting the vial adapter as shown in Figure 5. This will release the vial adapter, with the vial, from the syringe but leave the needle on the syringe (Figure 5).

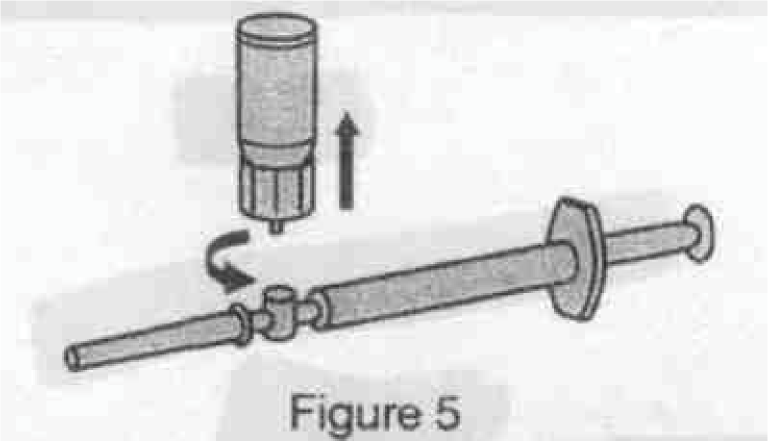

Figure 5

5. You have now reconstituted your Betaseron and are ready to be injected.

The injection should be administered immediately after mixing. If you are unable to give the injection immediately, you may refrigerate the medication in the syringe and inject within three hours. Do not freeze.

STEP 5: INJECTING BETASERON

1. Use a fresh alcohol wipe to **clean** the skin at the injection site. Use a circular motion from the centre of the injection site outward. Let the alcohol dry.

2. **Throw away** the wipe.

3. **Remove** the protective needle guard from the needle by pulling it without turning.

4. Gently **pinch** the skin around the site to lift it up a bit.

5. **Stick** the needle straight into the skin at a 90° angle with a quick, firm motion.

6. **Inject** the drug by using a slow, steady push (push the plunger all the way in until the syringe is empty).

7. **Remove** the needle from the skin.

8. Gently **massage** the injection site with a fresh alcohol wipe.

9. **Throw away** the syringe in the disposal unit.

10. **Discard** all other components.

PHARMACOLOGY

Pharmacokinetics: Given that serum concentrations of interferon beta-1b are low or not detectable following subcutaneous administration of 0.25 mg (8 MIU) or less of BETASERON (interferon beta-1b), pharmacokinetic information in MS patients receiving the recommended dose of BETASERON is not available. Following single and multiple daily subcutaneous administrations of 0.5 mg (16 MIU) BETASERON to healthy volunteers (n=12), serum interferon beta-1b concentrations were generally below 100 IU/mL. Peak serum interferon beta-1b concentrations occurred between 1 to 8 hours, with a mean peak serum interferon concentration of 40 IU/mL. Bioavailability, based on a total dose of 0.5 mg (16 MIU) BETASERON given as two subcutaneous injections at different sites, was approximately 50%.

After intravenous administration of BETASERON (0.006 mg [0.2 MIU] to 2.0 mg [64 MIU]), similar pharmacokinetic profiles were obtained from healthy volunteers (n=12) and from patients with diseases other than MS (n=142). In patients receiving single intravenous doses up to 2.0 mg (64 MIU), increases in serum concentrations were dose proportional. Mean serum clearance values ranged from 9.4 mL/min kg^{-1} to 28.9 mL/min kg^{-1} and were independent of dose. Mean terminal elimination half-life values ranged from 8.0 minutes to 4.3 hours and mean steady-state volume of distribution values ranged from 0.25 L/kg to 2.88 L/kg. Three-times-a-week intravenous dosing for 2 weeks resulted in no accumulation of interferon beta-1b in the serum of patients. Pharmacokinetic parameters after single and multiple intravenous doses of BETASERON were comparable.

TOXICOLOGY

Carcinogenicity: The carcinogenic potential of BETASERON (interferon beta-1b) was evaluated by studying its effect on the morphological transformation of the mammalian cell line BALBc-3T3. No significant increases in transformation frequency were noted. No carcinogenicity data are available in animals or humans.

Mutagenicity: BETASERON was not mutagenic when assayed for genotoxicity in the Ames bacterial test in the presence of metabolic activation.

REFERENCES

Blaschke TF, Horning SJ, Merigan TC et al. Recombinant β_{ser}-interferon inhibits antipyrine clearance in man. Clinical Research 1985; 33(1): 19A.

Carlin JM, Borden EC, Sondel PM et al. Biologic response modifier-induced indoleamine 2,3-dioxygenase activity in human peripheral blood mononuclear cell cultures. J Immunol 1987; 139 (7): 2414-2418.

Colby CB, Inoue M, Thompson M, et al. Immunologic differentiation between *E. coli* and CHO cell-derived recombinant and natural human β-interferons. J Immunol 1984; 133(6): 3091-3095.

De Maeyer E, De Maeyer-Guignard J; The interferon gene family. In: De Maeyer E, De Maeyer-Guignard J. Interferons and other regulatory cytokines. New York, Wiley and Sons, Inc. 1988; 5-38.

European Study Group on Interferon β-1b in secondary progressive MS. Placebo-controlled multicentre randomized trial of interferon β-1b in treatment of secondary progressive multiple sclerosis. The Lancet 1998; 352: 1491-1497.

Goldstein D, Sielaff KM, Storer BE, et al. Human biologic response modification by interferon in the absence of measurable serum concentrations: a comparative trial of subcutaneous and intravenous interferon-β serine. J Natl Cancer Inst 1989; 81(14): 1061-1068.

Knobler RL, Greenstein JI, Johnson KP et al. Systemic recombinant human interferon-β treatment of relapsing-remitting multiple sclerosis: pilot study analysis and six-year follow-up. J Interferon Res 1993; 13: 333-340.

Lengyel P. Biochemistry of interferons and their actions. Ann Rev Biochem 1982; 51: 251-282.

Paty DW, Li DKB et al. Interferon beta-1b is effective in relapsing-remitting multiple sclerosis. Neurology 1993; 43: 662-667.

Pestka S, Langer JA, Zoon KC et al. Interferons and their actions. Ann Rev Biochem 1987; 56: 727-777.

Poser CM, Paty DW, Scheinberg L, et al. New diagnostic criteria for multiple sclerosis: guidelines for research protocols. Ann Neurol 1983; 13(3): 227-231.

Rosenblum MG, Yung WKA, Kelleher PJ, Ruzicka F, et al. Growth inhibitory effects of interferon-β but not interferon-α on human glioma cells: correlation of receptor binding, 2',5'-oligoadenylate synthetase and protein kinase activity. J Interferon Res 1990; 10: 141-151.

Ruzicka FJ, Jach ME, Borden EC. Binding of recombinant-produced interferon βser to human lymphoblastoid cells. J Biol Chem 1987; 262(33): 16142-16149.

Schiller JH, Horisberger MA, Bittner G, et al. Effects of combinations of interferon-β_{ser} and interferon-γ on interferon-inducible proteins and on the cell cycle. J Biol Resp Mod 1990; 9: 368-377.

The IFNB Multiple Sclerosis Study Group. Interferon beta-1b is effective in relapsing-remitting multiple sclerosis. Neurology 1993; 43: 655-661.

The IFNB Multiple Sclerosis Study Group and the University of British Columbia MS/MRI Analysis Group. Interferon beta-1b in the treatment of multiple sclerosis: final outcome of the randomized controlled trial. Neurology 1995; 45: 1277-1285.

Uze G, Lutfalla G, Gresser I. Genetic transfer of a functional human interferon α receptor into mouse cells: cloning and expression of its cDNA. Cell 1990; 60: 225-234.

Witt PL, Spear GT, Helgeson DO, et al. Basal and interferon-induced 2',5'-oligoadenylate synthetase in human monocytes, lymphocytes, and peritoneal macrophages. J Interferon Res 1990; 10: 393-402.

Witt PL, Storer BE, Bryan GT, et al. Pharmacodynamics of biological response in vivo after single and multiple doses of interferon-β. J Immunotherapy 1993; 13: 191-200.

Betaseron®

Part 3 - Monographie (Canada / French-language document)

Revision Date: 28 July 2003

MONOGRAPHIE

PrBETASERON®

Interféron bêta-1b

CLASSE THÉRAPEUTIQUE

Immunomodulateur

Berlex Canada inc.
334, avenue Avro
Pointe-Claire (Québec)
H9R 5W5

Date de révision : 28 juillet 2003

La présente monographie est la propriété exclusive de Berlex Canada inc. et ne peut être citée, publiée ou reproduite sans l'autorisation écrite de Berlex Canada inc.

MONOGRAPHIE

^{Pr}BETASERON®

Interféron bêta-1b

CLASSE THÉRAPEUTIQUE

Immunomodulateur

ACTION ET PHARMACOLOGIE CLINIQUE

Description

BETASERON® (interféron bêta-1b) est un produit protéique purifié, stérile et lyophilisé, préparé par des techniques de l'ADN recombinant et destiné à l'injection. L'interféron bêta-1b est fabriqué par la fermentation bactérienne d'une souche d'*Escherichia coli* renfermant un plasmide créé par génie génétique et porteur du gène codant pour l'interféron bêta$_{ser17}$ humain. Le gène natif, obtenu à partir de fibroblastes humains, a été modifié de telle sorte que le résidu cystéine en position 17 est remplacé par de la sérine. L'interféron bêta-1b est une protéine hautement purifiée composée de 165 acides aminés. Sa masse moléculaire est d'environ 18 500 daltons. À la différence de la protéine naturelle, il est dépourvu de chaînes latérales de glucides.

Généralités

Les interférons forment une famille de protéines naturelles dont la masse moléculaire varie de 15 000 à 21 000 daltons. Trois grandes classes d'interférons ont été identifiées : alpha, bêta et gamma. Les activités biologiques respectives de l'interféron bêta, de l'interféron alpha et de l'interféron gamma se chevauchent, mais demeurent distinctes. L'activité de l'interféron bêta-1b est spécifique de l'espèce ; par conséquent, les données pharmacologiques les plus pertinentes sur BETASERON (interféron bêta-1b) sont issues de travaux effectués *in vivo* chez l'être humain ou sur des cellules humaines en culture.

Activité biologique

Il a été démontré que l'interféron bêta-1b est doué de propriétés antivirales et immunomodulatrices. On ne connaît pas précisément les modes d'action de BETASERON dans la sclérose en plaques (SEP). Néanmoins, on sait que la modification de la réponse biologique induite par l'interféron bêta-1b résulte de son interaction avec des récepteurs spécifiques présents à la surface des cellules humaines. La fixation de l'interféron bêta-1b à ces récepteurs déclenche l'expression de gènes induisant la synthèse d'un certain nombre de composés (ex. : 2',5'-oligoadénylate synthétase, protéine-kinase et indoléamine 2,3-dioxygénase) qui seraient des médiateurs de l'action biologique de l'interféron bêta-1b. Un certain nombre de ces composés ont été facilement décelés dans le sérum et dans la fraction cellulaire du sang de patients traités par l'interféron bêta-1b.

Études cliniques

L'efficacité de BETASERON administré par voie sous-cutanée à raison de 8 MUI tous les deux jours a été évaluée dans le cadre de deux études cliniques contrôlées par placebo, la première menée auprès de patients atteints de sclérose en plaques (SEP) rémittente (n = 124) et la deuxième, de patients atteints de SEP progressive-secondaire (n = 360).

1. SEP rémittente

L'efficacité de BETASERON dans la SEP rémittente a fait l'objet d'une étude clinique multicentrique (onze centres : quatre au Canada et sept aux États-Unis) parallèle, randomisée, à double insu et contrôlée par placebo, d'une durée de deux ans, menée auprès de patients atteints de SEP rémittente. Âgés de 18 à 50 ans, les sujets étaient ambulatoires (≤ 5,5 sur l'échelle étendue d'incapacité de Kurtzke [EDSS]), avaient une SEP cliniquement certaine et/ou biologiquement certaine conformément à la classification de Poser, et avaient eu au moins deux poussées au cours des deux années précédant l'étude, mais aucune au cours du dernier mois. Les patients qui avaient reçu un traitement immunosuppresseur ont été exclus.

Le protocole définissait une poussée comme l'apparition d'un nouveau symptôme/signe clinique persistant pendant au moins 24 heures, ou encore comme l'aggravation clinique d'un

symptôme/signe préexistant (demeuré stable pendant au moins 30 jours) persistant pendant au moins 24 heures.

Les patients ont été répartis au hasard en trois groupes, recevant respectivement un placebo (n = 123), 0,05 mg (1,6 MUI) de BETASERON (n = 125) ou 0,25 mg (8 MUI) de BETASERON (n = 124) auto-administrés par voie sous-cutanée tous les deux jours. Les résultats obtenus chez les 372 premiers patients randomisés ont été évalués après deux ans de traitement.

Les patients qui ont nécessité plus de trois corticothérapies de 28 jours ont été retirés de l'étude. La prise d'analgésiques mineurs (ex. : acétaminophène), d'antidépresseurs et de baclofène par voie orale était permise ; par contre, l'utilisation à long terme d'anti-inflammatoires non stéroïdiens (AINS) ne l'était pas.

Les paramètres d'évaluation primaires étaient : 1) fréquence des poussées par patient ; et 2) proportion de patients n'ayant eu aucune poussée. Les paramètres d'évaluation secondaires sont décrits au tableau 1.

En plus des épreuves cliniques, on a pratiqué un examen annuel par imagerie par résonance magnétique (IRM), dont les résultats ont servi à quantifier l'étendue de la maladie, définie comme la modification de la surface totale occupée par les lésions de SEP. Chez un sous-groupe de patients (n = 52) d'un centre, des examens IRM ont été pratiqués toutes les six semaines, et les résultats ont été quantifiés pour exprimer l'activité de la maladie, définie par la modification de la taille des lésions et de leur nombre.

Résultats à la fin de la période de traitement de deux ans définie par le protocole (voir tableau 1) : l'analyse a révélé une réduction de 31 % du taux annuel de poussées, qui s'élevait à 1,31 dans le groupe placebo et à 0,9 dans le groupe recevant 0,25 mg (8 MUI) de BETASERON (p = 0,0001). La proportion de patients n'ayant eu aucune poussée était de 16 % dans le groupe placebo, comparativement à 25 % dans le groupe recevant 0,25 mg (8 MUI) de BETASERON.

Parmi les 372 premiers patients randomisés, 72 (19 %) ont été retirés de l'étude avant la fin des deux années de traitement. Les raisons du retrait variaient selon le groupe de traitement. La prise excessive de corticostéroïdes a justifié 11 des 26 retraits dans le groupe placebo, mais seulement 1 des 25 retraits du groupe 0,25 mg (8 MUI). Par contre, les retraits en raison de réactions défavorables au traitement ont été plus fréquents chez les patients qui prenaient BETASERON : ils représentent respectivement 1 et 10 retraits dans les groupes placebo et 0,25 mg (8 MUI).

Au cours des deux ans, on a compté 25 hospitalisations attribuables à la SEP dans le groupe recevant 0,25 mg (8 MUI) de BETASERON, comparativement à 48 dans le groupe placebo. Le nombre d'hospitalisations sans rapport avec la SEP était comparable dans les deux groupes : 16 dans le groupe 0,25 mg (8 MUI) de BETASERON et 15 dans le groupe placebo. Le nombre moyen de jours de corticothérapie pour le soulagement de symptômes de SEP a été de 41 jours dans le groupe recevant 0,25 mg (8 MUI) de BETASERON et de 55 jours dans le groupe placebo ($p = 0,004$).

Tableau 1<br>Résultats de l'étude de deux ans<br>Paramètres d'évaluation primaires et secondaires							
**Paramètres d'efficacité**		**Groupes de traitement**			**Comparaisons statistiques** *p*		
**Paramètres d'évaluation clinique primaires**		**Placebo** (n = 123)	**0,05 mg (1,6 MUI)** (n = 125)	**0,25 mg (8 MUI)** (n = 124)	**Placebo** *vs* **0,05 mg (1,6 MUI)**	**0,05 mg (1,6 MUI)** *vs* **0,25 mg (8 MUI)**	**Placebo** *vs* **0,25 mg (8 MUI)**
**Taux annuel de poussées**		1,31	1,14	0,90	0,005	0,113	0,0001
**Proportion de patients n'ayant eu aucune poussée**†		16 %	18 %	25 %	0,609	0,288	0,094
**Fréquence des poussées par patient**	**0**†	20	22	29	0,151	0,077	0,001
	**1**	32	31	39			
	**2**	20	28	17			
	**3**	15	15	14			
	**4**	15	7	9			
	**≥ 5**	21	16	8			
**Paramètres d'évaluation secondaires**††							
**Nombre médian de mois avant la première poussée au cours de l'étude**		5	6	9	0,299	0,097	0,010
**Taux annuel de poussées modérées ou graves**		0,47	0,29	0,23	0,020	0,257	0,001
**Nombre moyen de jours de poussées modérées ou graves par patient**		44,1	33,2	19,5	0,229	0,064	0,001
**Modification moyenne de la cote sur l'EDSS**‡ **à la fin de la période d'évaluation**		0,21	0,21	-0,07	0,995	0,108	0,144
**Modification moyenne de la cote de Scripps**‡‡ **à la fin de la période d'évaluation**		-0,53	-0,50	0,66	0,641	0,051	0,126
**Durée médiane de chaque poussée (jours)**		36	33	35,5	NC	NC	NC
**Modification (%) de l'étendue moyenne des lésions à l'IRM à la fin de la période d'évaluation**		21,4 %	9,8 %	-0,9 %	0,015	0,019	0,0001

NC non calculé

† 14 patients n'ayant eu aucune poussée (0 du groupe placebo, 6 du groupe 0,05 mg et 8 du groupe 0,25 mg) se sont retirés de l'étude au cours des six premiers mois de traitement. Ces patients ont été exclus de l'analyse.

†† Les séquelles et la fonction neurologique, dont l'évaluation était exigée par le protocole, n'ont pas été analysées individuellement, mais se reflètent dans la cote sur l'EDSS.

‡ L'échelle EDSS s'étend de 0 à 10, les cotes plus élevées reflétant une plus grande incapacité.

‡‡ L'échelle de Scripps s'étend de 0 à 100, les cotes moins élevées reflétant une plus grande incapacité.

Les résultats des examens IRM ont aussi été analysés. On a obtenu la distribution de la fréquence des modifications, en pourcentage, de l'étendue des lésions après deux ans en regroupant les pourcentages en intervalles successifs d'égale grandeur. La figure 1 illustre sous forme d'histogramme la proportion de patients dans chaque intervalle. La modification médiane dans le groupe 0,25 mg (8 MUI) était de -1,1 %, soit considérablement moins que la médiane de 16,5 % dans le groupe placebo (p = 0,0001).

Dans un centre où 52 patients ont passé des examens IRM à intervalles plus rapprochés (toutes les six semaines), le pourcentage de clichés révélant de nouvelles lésions ou l'extension de lésions préexistantes était de 29 % dans le groupe placebo et de 6 % dans le groupe 0,25 mg (8 MUI) (p = 0,006).

Figure 1 : Distribution des modifications de l'étendue des lésions à l'IRM

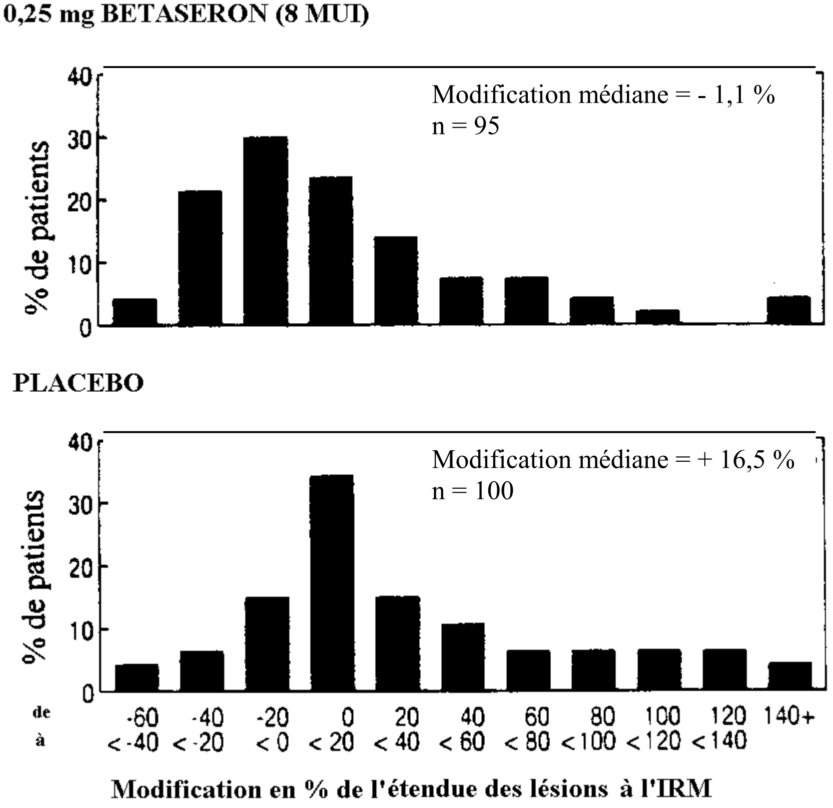

L'IRM est considérée comme une méthode utile pour visualiser les modifications de la substance blanche reflétant vraisemblablement les processus pathologiques qui, dans le système nerveux central (SNC), seraient à l'origine de certains des signes et symptômes caractéristiques de la SEP rémittente. On ne connaît pas le lien exact entre les observations à l'IRM et l'état clinique du patient. L'extension des lésions est souvent non corrélée aux poussées cliniques de la maladie, sans doute parce que de nombreuses lésions siègent dans des régions dites « silencieuses » du SNC. Par ailleurs, on ne peut prévoir avec certitude dans quelle proportion les lésions observées à l'IRM formeront des foyers de démyélinisation irréversible (c.-à-d. les plaques classiquement observées dans la substance blanche). La valeur pronostique des résultats de l'IRM dans cette étude n'a pas été évaluée.

À la fin des deux années, les patients avaient l'option de continuer le traitement à l'insu, et environ 80 % des patients de chaque groupe ont accepté. Bien que les patients des groupes BETASERON, particulièrement ceux du groupe 0,25 mg (8 MUI), aient semblé être avantagés pendant la troisième année de traitement, on n'a noté aucune différence statistiquement significative entre les groupes BETASERON et le groupe placebo en ce qui a trait à la fréquence des poussées ou aux paramètres secondaires décrits dans le tableau 1. Comme il a été mentionné plus haut, l'analyse des résultats des deux premières années a révélé une réduction de 31 % de la fréquence des poussées dans le groupe 0,25 mg (8 MUI) par rapport au groupe placebo. La valeur de *p* pour cette différence était de 0,0001. L'analyse des données de la troisième année a révélé une différence de 28 % entre les groupes de traitement, la valeur de *p* étant de 0,065. Le nombre plus faible de patients pourrait expliquer la perte de signification statistique ; de plus, l'impossibilité de comparer directement les groupes pour l'année de prolongation complique l'interprétation des résultats. Selon les données IRM pour la troisième année, il ne semblerait pas y avoir de bienfaits additionnels dans les groupes BETASERON par rapport au groupe placebo.

Tout au long de l'étude, on a procédé à des prélèvements sanguins pour vérifier l'apparition d'anticorps contre l'interféron bêta-1b. Parmi les patients recevant 0,25 mg (8 MUI) de BETASERON tous les deux jours (n = 124), 45 % ont présenté une activité sérique neutralisante dans au moins un prélèvement. Le tiers des patients ont présenté une activité neutralisante confirmée par au moins deux prélèvements consécutifs. L'apparition d'une activité neutralisante pourrait être associée à une baisse de l'efficacité clinique, mais le lien exact entre la formation d'anticorps et l'efficacité thérapeutique n'est pas établi.

2. SEP progressive-secondaire

L'efficacité de BETASERON administré par voie sous-cutanée à raison de 0,25 mg (8 MUI) tous les deux jours a été évaluée lors d'une étude multicentrique (32 sites) randomisée, à double insu et contrôlée par placebo, d'une durée de trois ans, effectuée à l'échelle européenne chez des patients souffrant de SEP progressive-secondaire.

L'étude portait sur des sujets âgés de 18 à 55 ans qui présentaient une SEP cliniquement certaine ou biologiquement certaine depuis au moins un an. La maladie devait être passée à la forme progressive-secondaire, et la détérioration ne pouvait pas être liée exclusivement à un rétablissement incomplet à la suite des poussées. Au moment de leur admission à l'étude, les patients devaient avoir une cote sur l'EDSS comprise entre 3,0 et 6,5 et avoir eu deux poussées clairement identifiées ou avoir présenté une détérioration d'au moins un point sur l'échelle EDSS (ou de 0,5 point entre les cotes 6,0 et 7,0) au cours des 24 mois précédents.

Le paramètre primaire d'évaluation était l'intervalle sans progression confirmée de la maladie. La progression se définissait par l'augmentation de un point sur l'échelle EDSS par rapport aux valeurs de départ, si la cote de départ était comprise entre 3,0 et 5,5, ou de 0,5 point, si la cote de départ était de 6,0 ou de 6,5. L'augmentation de la cote devait se maintenir pendant trois mois pour que la progression soit confirmée. Les paramètres secondaires d'évaluation de l'efficacité étaient l'intervalle précédant le confinement au fauteuil roulant (cote sur l'EDSS de 7,0) et le taux annuel de poussées.

Bien que l'étude ait été conçue pour durer trois ans, on a procédé à une analyse intermédiaire prédéterminée de l'efficacité chez tous les patients après deux ans de participation à l'étude. Cette analyse a amené un comité consultatif indépendant à mettre fin à l'étude prématurément. Au moment de l'analyse intermédiaire du critère primaire, on disposait d'environ 85 % de toutes les cotes sur l'EDSS pour l'étude de trois ans. L'analyse principale de l'efficacité portait sur tous les patients répartis aléatoirement dans les groupes (analyse en intention de traiter). La principale méthode d'analyse du paramètre primaire d'évaluation était une analyse non paramétrique de covariance avec stratification des centres et ajustement en fonction de la cote de départ sur l'EDSS. Les résultats présentés ci-dessous correspondent aux données à la fin de l'étude.

Pendant l'étude, l'évaluation de la cote sur l'EDSS était effectuée par un médecin qui ne participait pas au traitement du patient. Tous les médecins qui évaluaient la cote sur l'EDSS recevaient, à

intervalles réguliers, une formation visant à garantir une évaluation constante de cette cote. Tous les efforts nécessaires ont été déployés pour assurer un examen à l'insu (ex. : des vêtements standard étaient obligatoires afin de camoufler les points d'injection).

Au total, 718 patients (358 recevant un placebo et 360 recevant BETASERON) ont été inscrits à l'étude. Dans les deux groupes, il y avait plus de femmes que d'hommes (groupe placebo : 64,2 % *vs* 35,8 % ; groupe BETASERON : 58,1 % *vs* 41,9 %), mais la différence n'était pas statistiquement significative. La durée moyenne du traitement se chiffrait à 886 jours dans le groupe placebo et à 909 jours dans le groupe BETASERON. Quatre-vingt-huit (88) patients ont été perdus de vue ; les autres ont été suivis jusqu'à la fin de l'étude, qu'ils aient continué de prendre le médicament ou non. Pendant la période de trois ans, 117 patients (32,7 %) du groupe placebo et 103 patients (29,6 %) du groupe BETASERON ont abandonné prématurément le traitement. Le manque d'efficacité, les effets indésirables et l'inobservance étaient les motifs les plus fréquents de l'abandon prématuré, chez 15,6, 6,4 et 7,5 % des sujets du groupe placebo et chez 7,5, 14,2 et 3,3 % des sujets du groupe BETASERON, respectivement. Les groupes de traitement étaient bien équilibrés pour toutes les valeurs de départ pertinentes, y compris la cote de départ sur l'EDSS et le temps écoulé depuis le premier signe de SEP progressive-secondaire.

Sur le plan de l'intervalle sans progression de l'incapacité, on a observé une différence statistiquement significative en faveur de BETASERON (p = 0,0046), comme le montre le tableau 2. Le ralentissement de la progression, qui est devenu apparent après neuf mois de traitement, est devenu statistiquement significatif à partir du mois 12. La proportion de patients chez qui la progression de la maladie a été confirmée était de 60,9 % dans le groupe placebo comparativement à 51,9 % dans le groupe BETASERON (p = 0,0245).

L'efficacité du traitement était constante, quelle que soit la cote de départ sur l'EDSS. Cependant, la différence relative dans la proportion de patients, entre le groupe BETASERON et le groupe placebo, chez lesquels la progression de la maladie était confirmée était plus faible lorsque la cote de départ sur l'EDSS était ≥ 6,0 comparativement aux autres cotes de départ sur l'EDSS lorsqu'on estimait que la maladie avait progressé chez tous les patients perdus de vue (EDSS ≤ 3,5 : 27,1 % ; EDSS entre 4,0 et 5,5 : 17,8 % et EDSS ≥ 6,0 : 5,8 %). Par contre, lorsqu'on estimait que la maladie n'avait pas progressé chez ces derniers, les valeurs respectives étaient de 16,6, 15,5 et 14,2 % (voir tableau 3). Bien qu'il y ait légèrement plus d'hommes que de femmes dans le groupe BETASERON

chez lesquels la progression de la maladie a été confirmée, l'analyse de régression logistique par morceaux n'a révélé aucune modification significative des effets du traitement due au sexe des patients (p = 0,4335).

Une estimation de Kaplan-Meier des données (analyse *a posteriori*) est présentée à la figure 2. L'estimation de Kaplan-Meier du pourcentage de patients chez lesquels il y aura progression de la maladie à la fin des trois ans était de 53,9 % pour le groupe placebo et de 45,3 % pour le groupe BETASERON.

L'intervalle précédant le confinement au fauteuil roulant (cote sur l'EDSS de 7,0) a aussi été prolongé de façon significative (p = 0,0047) et la proportion de patients confinés au fauteuil roulant se chiffrait à 28,5 % dans le groupe placebo comparativement à 18,6 % dans le groupe BETASERON (p = 0,0069).

BETASERON a réduit la fréquence des poussées de 26,3 % pendant toute la durée de l'étude (p = 0,0034). On a observé une diminution de la proportion de patients présentant des poussées modérées ou graves, qui était de 54,2 % dans le groupe placebo et de 47,2 % dans le groupe BETASERON (p = 0,0508). Les taux annuels moyens de poussées modérées ou graves se chiffraient respectivement à 0,44 et à 0,31 dans les groupes placebo et BETASERON (p = 0,0037).

L'incidence des hospitalisations attribuables à la SEP a aussi baissé : 44,4 % des sujets du groupe placebo ont dû être hospitalisés en raison de la SEP comparativement à 36,1 % des sujets du groupe BETASERON (p = 0,0003). Le pourcentage de patients ayant reçu une corticothérapie se chiffrait respectivement à 73,2 % et à 62,5 % dans les groupes placebo et BETASERON (p = 0,0010).

En plus des mesures cliniques, des examens annuels d'imagerie par résonance magnétique (IRM) ont été effectués. Tous les patients ont subi un examen IRM pondéré en T2 au départ et une fois par an par la suite, et un sous-groupe de patients (groupe placebo, n = 61 ; groupe BETASERON, n = 64) a subi des examens mensuels pendant les mois 1 à 6 et 19 à 24 en plus des examens annuels prévus pour tous les patients de l'étude. Les résultats des paramètres secondaires et tertiaires mesurés par IRM ont permis d'observer des différences significatives entre les groupes de traitement en faveur de BETASERON (voir tableau 2). On ignore le lien exact entre les résultats des examens IRM et le statut clinique des patients.

On a prélevé des échantillons de sérum tout au long de l'étude afin de vérifier le développement d'anticorps neutralisants contre l'interféron bêta-1b. On a également effectué des analyses afin d'évaluer l'association entre la présence d'anticorps neutralisants (mesurée par dosage MxA) et la réponse au traitement, mesurée par l'analyse des résultats cliniques et des résultats des examens IRM. On a confirmé la présence d'anticorps neutralisants dans un ratio de 1:20, de 1:100 et de 1:400 chez 28, 14 et 8 % des patients, respectivement. Malgré un traitement continu par BETASERON, on a observé des titres négatifs après le premier développement de titres quantifiables confirmés chez 50 % des patients qui avaient déjà présenté des anticorps neutralisants. Le lien entre la formation d'anticorps et l'efficacité clinique est inconnu.

**Tableau 2<br>Résultats de l'étude sur la SEP progressive-secondaire<br>Résumé des paramètres d'évaluation clés**			
	**Groupes de traitement**		***p***
	**Placebo<br><br>(n = 358)**	**BETASERON 0,25 mg (8 MUI)<br><br>(n = 360)**	
**Paramètres primaires d'évaluation clinique**			
Intervalle sans progression confirmée de l'incapacité[1]			0,0046
**Année 1**	0,70	0,81	0,0032
**Année 2**	0,53	0,64	0,0013
**Mois 33**	0,44	0,53	0,0066
**Paramètres secondaires d'évaluation clinique**			
Intervalle précédant le confinement au fauteuil roulant[2]			0,0047
**Année 1**	0,90	0,96	0,0139
**Année 2**	0,81	0,86	0,0096
**Mois 36**	0,69	0,80	0,0047
Proportion de patients confinés au fauteuil roulant	28,5 %	18,6 %	0,0069
Taux annuel moyen de poussées	0,57	0,42	0,0034
IRM : pourcentage moyen de changement de taille des lésions observées en T2 (de la valeur de départ au dernier examen IRM)	15,4	-2,1	< 0,0001
IRM : nombre moyen de nouvelles lésions actives (mois 1 à 6)	10,24<br><br>(n = 61)	3,57<br><br>(n = 64)	< 0,0001
**Paramètres tertiaires d'évaluation clinique**			
Proportion de patients chez qui la progression de la maladie a été confirmée	60,9 %	51,9 %	0,0245
Cote moyenne sur l'EDSS à la fin de la période d'évaluation	5,93	5,58	0,0065
Intervalle médian avant la première poussée (jours)	385	644	0,0088
IRM : nombre moyen de lésions constamment rehaussées<br><br>(mois 1 à 6)	3,10<br><br>(n = 61)	1,02<br><br>(n = 64)	0,0009
IRM : nombre moyen de lésions constamment rehaussées<br><br>(mois 19 à 24)	3,04<br><br>(n = 53)	0,36<br><br>(n = 56)	0,0004

[1] Probabilité que la maladie ne progresse pas durant la période déterminée.
[2] Probabilité que le patient ne soit pas confiné au fauteuil roulant durant la période déterminée.

Tableau 3<br>Proportion de patients chez qui la progression de la maladie est confirmée stratifiée par catégorie de cote de départ sur l'EDSS							
Cote de départ sur l'EDSS	Groupe de traitement	Analyse A en intention de traiter[1]			Analyse B en intention de traiter[2]		
		% de patients chez qui la progression de la maladie est confirmée	Différence par rapport au groupe placebo (%)		% de patients chez qui la progression de la maladie est confirmée	Différence par rapport au groupe placebo (%)	
			Relative	Absolue		Relative	Absolue
≤ 3,5	Placebo<br>Betaseron	55,3<br>40,3	-27,1	-15,0	44,7<br>37,3	-16,6	-7,4
4,0 – 5,5	Placebo<br>Betaseron	63,4<br>52,1	-17,8	-11,3	54,9<br>46,4	-15,5	-8,5
≥ 6,0	Placebo<br>Betaseron	60,4<br>56,9	-5,8	-3,5	55,6<br>47,7	-14,2	-7,9
Toutes cotes confondues	Placebo<br>Betaseron	60,9<br>51,9	-14,8	-9,0	53,9<br>45,3	-16,0	-8,6

[1] Analyse A en intention de traiter : Selon l'hypothèse qu'il y avait progression confirmée de la maladie chez les patients perdus de vue au cours de la période de suivi de trois mois.

[2] Analyse B en intention de traiter : Selon l'hypothèse qu'il n'y avait pas de progression confirmée de la maladie à la fin de l'étude chez les patients perdus de vue.

Figure 2 : Début de la progression de l'incapacité par période dans l'étude (Méthodologie de Kaplan-Meier : analyse *a posteriori*)

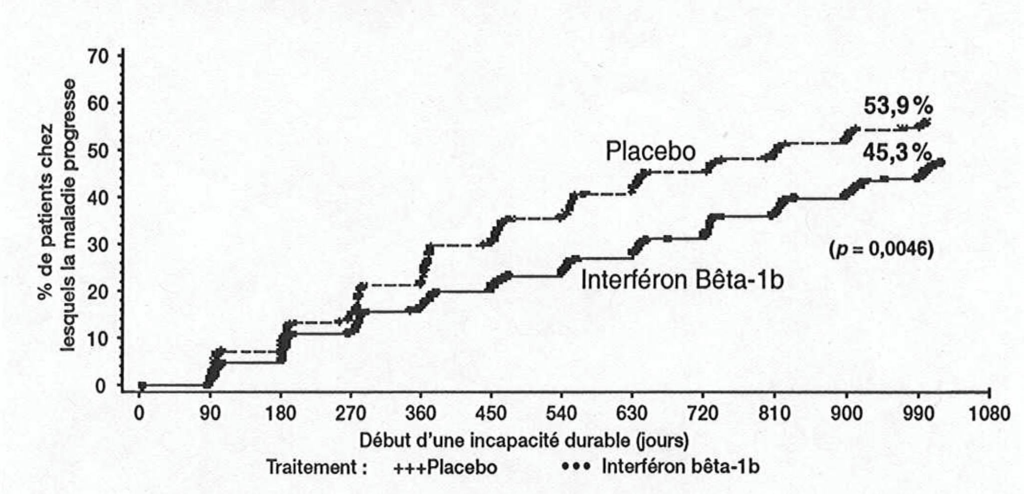

Estimation du pourcentage de patients chez lesquels la maladie progressera d'ici la fin des trois années.

Note : La valeur de p *de 0,0046 fait référence à la différence statistique entre la distribution globale des deux courbes et non à la différence entre les estimations à un moment donné.*

INDICATIONS ET USAGE CLINIQUE

BETASERON (interféron bêta-1b) est indiqué pour :

- réduire la fréquence des poussées cliniques chez les patients ambulatoires atteints de sclérose en plaques (SEP) rémittente. La SEP rémittente se caractérise par une alternance de poussées de dysfonction neurologique et de rémissions complètes ou incomplètes.
- ralentir la progression de l'incapacité et réduire la fréquence des poussées cliniques chez les patients atteints de SEP progressive-secondaire.

L'innocuité et l'efficacité de BETASERON dans la SEP progressive-primaire n'ont pas été évaluées.

CONTRE-INDICATIONS

BETASERON (interféron bêta-1b) est contre-indiqué chez les patients présentant des antécédents d'hypersensibilité à l'interféron bêta naturel ou recombinant, à l'albumine humaine USP ou à tout autre ingrédient de la préparation.

MISES EN GARDE

L'administration de cytokines à des patients présentant une gammopathie monoclonale a été associée à l'apparition d'un syndrome général de fuite capillaire accompagné de symptômes rappelant l'état de choc et à une issue fatale.

Au cours des trois années de l'étude clinique sur la SEP rémittente, il y a eu un cas de suicide et quatre tentatives de suicide parmi les 372 participants. Les cinq patients recevaient BETASERON (interféron bêta-1b) (trois étaient du groupe 0,05 mg [1,6 MUI], et les deux autres, du groupe 0,25 mg [8 MUI]). Aucune tentative de suicide n'est survenue chez les patients ne recevant pas BETASERON. Au cours de l'étude clinique sur la SEP progressive-secondaire, on a enregistré cinq tentatives de suicide dans le groupe placebo et trois dans le groupe BETASERON, y compris un suicide dans chaque groupe. Des cas de dépression et des suicides se sont produits chez des patients

traités par l'interféron alpha, un composé apparenté. Avant de recevoir BETASERON, le patient doit être prévenu de la possibilité de réactions dépressives ou d'idées suicidaires et de la nécessité d'avertir son médecin dès l'apparition de tels symptômes. Advenant des symptômes de dépression, il faut surveiller de près le patient et envisager l'interruption du traitement.

PRÉCAUTIONS

Généralités

À de rares occasions, des cas de cardiomyopathie ont été signalés. Dans ces circonstances, et si on soupçonne un lien avec BETASERON (interféron bêta-1b) , il faut interrompre le traitement.

De rares cas de dysfonction thyroïdienne (hyper- autant que hypothyroïdie) ont été associés à l'utilisation de BETASERON.

Les symptômes de syndrome grippal observés au cours de traitements par BETASERON peuvent se révéler stressants pour les patients souffrant de troubles cardiaques graves. Il faut suivre de près les patients atteints de cardiopathies telles qu'angine, insuffisance cardiaque congestive ou arythmie pour éviter que leur état clinique se détériore.

Renseignements à transmettre aux patients

Les patients doivent être informés des techniques d'injection pour qu'ils puissent s'auto-administrer BETASERON de façon sécuritaire. (Voir les renseignements ci-dessous et la section **INFORMATION À L'INTENTION DES PATIENTS.**)

Directives pour l'auto-administration **:** On recommande que la première injection soit faite par un médecin ou encore sous la supervision directe d'un médecin. Les patients doivent être informés des techniques aseptiques de reconstitution et d'auto-injection de BETASERON. Il est également recommandé de passer soigneusement en revue la section **INFORMATION À L'INTENTION DES PATIENTS**.

Il faut avertir les patients de ne pas réutiliser les aiguilles et les seringues et les informer des mesures à prendre pour l'élimination sécuritaire du matériel utilisé. Il faut aussi leur expliquer comment se

procurer un récipient à l'épreuve des perforations pour les aiguilles et les seringues utilisées, ainsi que la marche à suivre pour l'élimination des récipients pleins.

Globalement, 80 % des sujets des deux études cliniques contrôlées ont signalé des réactions au point d'injection au moins une fois au cours du traitement. Ces résultats ont été confirmés par le suivi post-commercialisation, qui a également révélé une faible fréquence de nécrose au point d'injection.

Les réactions de nécrose au point d'injection se produisaient habituellement au début du traitement (dans la plupart des cas signalés, dans les deux à trois premiers mois). Le nombre de réactions de nécrose au point d'injection variait selon les patients.

Dans de rares cas, la zone nécrosée s'étendait à la couche adipeuse sous-cutanée ou aux fascias. La réponse à l'antibiothérapie et/ou à la corticothérapie variait selon les patients. Chez certains d'entre eux, on a réalisé un débridement électif ou, plus rarement, une greffe de peau afin de favoriser la cicatrisation, qui pouvait prendre de trois à six mois.

Dans certains cas, les lésions nécrotiques se sont cicatrisées pendant le traitement par BETASERON, alors que dans d'autres, de nouvelles lésions sont apparues même après l'arrêt du traitement.

Il faut évaluer toute réaction avec soin pour en déterminer la nature et la gravité. Il faut aussi vérifier périodiquement si le patient comprend et applique les méthodes d'asepsie pour l'auto-administration.

Il n'est pas rare d'observer des symptômes pseudo-grippaux au début du traitement par BETASERON. Dans les études cliniques contrôlées sur la SEP, la prise d'acétaminophène était permise pour le soulagement de la fièvre ou de la myalgie.

Il faut aviser les patients de ne pas modifier la dose ou la fréquence d'administration sans avoir préalablement consulté leur médecin.

Information sur les effets indésirables **:** Les patients doivent être informés des effets indésirables courants de BETASERON, en particulier des réactions au point d'injection et du syndrome pseudo-grippal (voir **EFFETS INDÉSIRABLES**).

Il faut avertir les patients de signaler toute réaction dépressive ou toute idée suicidaire (voir **MISES EN GARDE**).

Les patients doivent être prévenus du potentiel abortif de BETASERON (voir **PRÉCAUTIONS, Grossesse**).

Épreuves de laboratoire

Les épreuves de laboratoire suivantes sont recommandées avant l'amorce du traitement par BETASERON et à intervalles réguliers par la suite : épreuves de la fonction thyroïdienne, hémoglobinométrie, numération et formule leucocytaires, numération plaquettaire et chimie sanguine, y compris analyses de la fonction hépatique. Un test de grossesse, une radiographie pulmonaire et un ECG sont également recommandés avant le début du traitement. Dans les études contrôlées sur la SEP, un examen de suivi avait lieu tous les trois mois. Le protocole des études stipulait que le traitement par BETASERON devait être interrompu si le nombre absolu de polynucléaires neutrophiles devenait inférieur à 750/mm^3. Une fois ce chiffre revenu à plus de 750/mm^3, le traitement pouvait être réinstauré à 50 % de la dose. Aucun patient n'a été retiré de l'étude ou n'a reçu une dose réduite en raison d'une neutropénie ou d'une lymphopénie.

De même, si les taux d'AST et d'ALT (SGOT et SGPT) dépassaient dix fois la limite supérieure de la normale, ou si la bilirubine sérique dépassait cinq fois la limite supérieure de la normale, le traitement était interrompu. Au cours de l'étude contrôlée sur la SEP, dans tous les cas d'élévation des enzymes hépatiques, les taux sont revenus à la normale après interruption du traitement. Une fois les taux abaissés aux valeurs prescrites, le traitement pouvait être réinstauré à 50 % de la dose, dans la mesure où la reprise du traitement était cliniquement justifiée. La dose a été réduite chez deux patients à cause d'une élévation des enzymes hépatiques ; l'un d'eux a continué le traitement et l'autre a finalement été retiré de l'étude.

Interactions médicamenteuses

Les interactions de BETASERON avec d'autres médicaments n'ont pas été évaluées. Bien qu'aucune étude n'ait été effectuée pour évaluer les interactions médicamenteuses, il est à noter qu'un traitement

par des corticostéroïdes ou par la corticotrophine (ACTH) a été administré pendant des périodes allant jusqu'à 28 jours pour le traitement de poussées chez des patients (n = 180) recevant BETASERON.

L'administration de BETASERON chez trois patients cancéreux à des doses variant de 0,025 mg (0,8 MUI) à 2,2 mg (71 MUI) a conduit à une inhibition de l'élimination d'antipyrine proportionnelle à la dose. L'effet d'une dose de 0,25 mg (8 MUI) de BETASERON administrée tous les deux jours sur le métabolisme des médicaments chez les patients atteints de SEP n'est pas connu.

Il a été signalé que les interférons réduisent l’activité des enzymes hépatiques dépendant du cytochrome P450 chez l’être humain et l’animal. Il faut donc user de prudence lorsqu’on administre BETASERON en association avec des agents qui ont un index thérapeutique étroit et dont la clairance dépend largement du cytochrome P450.

Altération de la fertilité

Chez des singes rhésus femelles dont le cycle menstruel était normal, l'administration de doses allant jusqu'à 0,33 mg (10,7 MUI)/kg/jour (soit 32 fois la dose recommandée chez l’être humain en tenant compte de la surface corporelle) pendant trois cycles menstruels consécutifs n'a eu aucun effet observable sur le cycle menstruel ni sur les fluctuations hormonales (progestérone et estradiol). On ignore si les effets observés chez l'animal à différentes doses sont applicables à l’être humain. Les effets de BETASERON sur la femme dont le cycle menstruel est normal ne sont pas connus.

Grossesse

BETASERON ne s'est pas révélé tératogène à des doses allant jusqu'à 0,42 mg (13,3 MUI)/kg/jour chez le singe rhésus, mais a montré un potentiel abortif proportionnel à la dose lors de l'administration de 0,028 mg (0,89 MUI)/kg/jour (soit 2,8 fois la dose recommandée chez l'être humain en tenant compte de la surface corporelle) à 0,42 mg (13,3 MUI)/kg/jour (soit 40 fois la dose recommandée chez l’être humain en tenant compte de la surface corporelle). On ignore si les effets observés chez l'animal à la suite de l’administration de différentes doses sont applicables à l’être humain. Aucune étude n'a été effectuée avec des doses plus faibles chez le singe. Des avortements spontanés sont survenus chez quatre des participantes à l'étude clinique sur BETASERON dans la SEP rémittente, alors qu'on a enregistré un avortement provoqué dans le groupe placebo et dans le groupe BETASERON durant l'étude sur la SEP progressive-secondaire. Administré à des singes

rhésus du 20e au 70e jour de gestation, BETASERON n'a pas eu d'effets tératogènes. On ignore cependant si BETASERON est tératogène chez l'être humain. Aucune étude satisfaisante et bien contrôlée n'a été effectuée chez la femme enceinte. Les femmes susceptibles de devenir enceintes doivent utiliser une méthode contraceptive fiable pendant le traitement. Si une patiente devient enceinte ou prévoit le devenir, elle doit cesser de prendre BETASERON. On ignore si les interférons ont un effet sur l'efficacité des contraceptifs oraux.

Allaitement

On ignore si BETASERON est excrété dans le lait maternel humain. Comme de nombreux médicaments le sont, il existe un risque d'effets indésirables graves chez le nourrisson ; on doit donc recommander aux patientes de cesser l'allaitement ou d'interrompre le traitement.

Enfants

L'innocuité et l'efficacité chez les patients de moins de 18 ans n'ont pas été établies.

Pharmacodépendance

Aucune donnée ni étude n'a laissé entrevoir la possibilité d'abus ou de dépendance au cours du traitement par BETASERON. Toutefois, le risque de pharmacodépendance n'a pas été systématiquement évalué.

EFFETS INDÉSIRABLES

On a observé les effets indésirables suivants au cours des études cliniques contrôlées par placebo sur BETASERON (interféron bêta-1b) administré à la dose recommandée de 0,25 mg (8 MUI) chez les patients souffrant de SEP rémittente (n = 124) et de SEP progressive-secondaire (n = 360).

1. SEP rémittente

Des réactions (85 %) et des cas de nécrose (5 %) au point d'injection sont survenus après l'administration de BETASERON. Les effets indésirables suivants ont été relevés de manière significative ($p < 0,05$) dans le groupe recevant 0,25 mg (8 MUI) de BETASERON, comparativement au groupe recevant un placebo : inflammation, douleur, hypersensibilité, nécrose et réactions non spécifiques. Seules l'inflammation, la douleur et la nécrose ont été signalées comme des effets indésirables graves. La fréquence des réactions au point d'injection a été calculée pour une

période de trois ans. Elle diminuait avec le temps, 79 % des patients ayant eu de telles réactions au cours des trois premiers mois de traitement, comparativement à 47 % au cours des six derniers mois. L'intervalle médian avant la première réaction au point d'injection était de sept jours. Les patients qui avaient des réactions au point d'injection ont signalé de telles réactions 183,7 jours par année. Trois patients du groupe recevant 0,25 mg (8 MUI) de BETASERON se sont retirés de l'étude en raison de douleurs au point d'injection.

Un syndrome pseudo-grippal a été signalé chez 76 % des patients recevant 0,25 mg (8 MUI) de BETASERON. Le syndrome pseudo-grippal était défini comme la présence concomitante d'au moins deux des symptômes suivants : fièvre, frissons, myalgie, malaises ou sudation. Seuls la myalgie, la fièvre et les frissons ont été déclarés de forte intensité chez plus de 5 % des patients. La fréquence du syndrome pseudo-grippal a aussi été calculée au cours des trois ans d'observation. La fréquence a diminué avec le temps, passant de 60 % des patients pendant les trois premiers mois de traitement à 10 % des patients au cours des six derniers mois. L'intervalle médian avant la première apparition du syndrome pseudo-grippal était de 3,5 jours et la durée médiane par patient était de 7,5 jours par année.

Les épreuves de laboratoire ont révélé les anomalies suivantes :

- nombre de lymphocytes < 1 500/mm^3 (82 %)
- taux d'ALT (SGPT) > 5 fois la valeur de départ (19 %)
- nombre absolu de polynucléaires neutrophiles < 1 500/mm^3 (18 %) (aucun patient n'a présenté un nombre absolu de polynucléaires neutrophiles < 500/mm^3)
- nombre de leucocytes < 3 000/mm^3 (16 %)
- taux de bilirubine totale > 2,5 fois la valeur de départ (6 %)

Trois patients du groupe recevant 0,25 mg (8 MUI) de BETASERON ont été retirés de l'étude en raison d'une élévation des enzymes hépatiques ; l'un des patients a été retiré après réduction de la dose (voir **PRÉCAUTIONS, Épreuves de laboratoire**).

Parmi les 76 femmes en âge de procréer du groupe 0,25 mg (8 MUI) de BETASERON, 21 (28 %) ont signalé des troubles menstruels, comparativement à 10 (13 %) des 76 femmes du groupe placebo. Les troubles signalés, tous d'intensité légère ou modérée, étaient notamment : métrorragies

et microrragies intermenstruelles, menstruations précoces ou retardées, menstruations plus courtes, et formation de caillots ou flux menstruel réduit durant les menstruations.

Des troubles mentaux tels que dépression, anxiété, labilité émotionnelle, dépersonnalisation, tentatives de suicide et confusion ont été observés chez des participants à l'étude. Deux patients ont été retirés de l'étude pour cause de confusion. On a également signalé un suicide et quatre tentatives de suicide. On ignore si ces symptômes sont dus à l'atteinte neurologique associée à la maladie, au traitement par BETASERON, ou aux deux. Des symptômes semblables ont été observés chez des patients traités par l'interféron alpha, et on croit que les deux interférons agissent par l'entremise du même récepteur. Advenant de tels symptômes, il faut surveiller le patient étroitement et envisager l'interruption du traitement.

D'autres effets indésirables ou résultats de laboratoire anormaux fréquents associés à l’administration de BETASERON sont énumérés dans les paragraphes suivants. Ces effets se sont manifestés à une fréquence d'au moins 5 % chez les 124 patients atteints de SEP recevant 0,25 mg (8 MUI) de BETASERON (interféron bêta-1b) tous les deux jours pendant une période allant jusqu'à trois ans dans le cadre de l'étude contrôlée, et à une fréquence au moins deux fois plus élevée que celle qui a été observée chez les 123 patients du groupe placebo. Les effets indésirables cliniques et biologiques couramment associés à BETASERON étaient :

- réaction au point d'injection (85 %)
- nombre de lymphocytes < 1 500/mm^3 (82 %)
- taux d'ALT (SGPT) > 5 fois la valeur de départ (19 %)
- nombre absolu de polynucléaires neutrophiles < 1 500/mm^3 (18 %)
- troubles menstruels (17 %)
- nombre de leucocytes < 3 000/mm^3 (16 %)
- palpitations (8 %)
- dyspnée (8 %)
- cystite (8 %)
- hypertension (7 %)
- douleur aux seins (7 %)
- tachycardie (6 %)
- troubles gastro-intestinaux (6 %)
- taux de bilirubine totale > 2,5 fois la valeur de départ (6 %)

• somnolence (6 %)
• laryngite (6 %)
• douleur pelvienne (6 %)
• ménorragie (6 %)
• nécrose au point d'injection (5 %)
• troubles vasculaires périphériques (5 %)

Au total, 277 patients atteints de SEP ont été traités par BETASERON à des doses de 0,025 mg (0,8 MUI) à 0,5 mg (16 MUI). Au cours des trois premières années de traitement, les effets indésirables cliniques ou les résultats de laboratoire anormaux non mentionnés ci-dessus et ayant conduit à des retraits étaient notamment :

• fatigue (2 %, six patients)
• arythmie cardiaque (< 1 %, un patient)
• urticaire allergique en réaction à l'injection (< 1 %, un patient)
• céphalées (< 1 %, un patient)
• réactions défavorables non spécifiques (< 1 %, un patient)
• sensation généralisée de malaise (< 1 %, un patient)

Le tableau suivant présente les effets indésirables et les résultats de laboratoire anormaux survenus à une fréquence d'au moins 2 % parmi les 124 patients recevant 0,25 mg (8 MUI) de BETASERON tous les deux jours pendant une période allant jusqu'à trois ans, dans le cadre de l'étude contrôlée. Leur fréquence était au moins 2 % plus élevée que celle qui a été observée chez les 123 patients du groupe placebo. Les effets indésirables signalés ont été regroupés conformément au glossaire standard COSTART pour réduire le nombre total de termes employés dans le tableau 4. Les effets décrits en termes trop imprécis, de même que les réactions vraisemblablement sans rapport avec le médicament ont été exclus du tableau.

TABLEAU 4

Effets indésirables et résultats de laboratoire anormaux

Effets indésirables	Placebo n = 123	0,25 mg (8 MUI) n = 124
**Corps entier**		
Réaction au point d'injection*	37 %	85 %
Céphalées	77 %	84 %
Fièvre*	41 %	59 %
Syndrome pseudo-grippal*	56 %	76 %
Douleur	48 %	52 %
Asthénie*	35 %	49 %
Frissons*	19 %	46 %
Douleur abdominale	24 %	32 %
Malaises*	3 %	15 %
Œdème généralisé	6 %	8 %
Douleur pelvienne	3 %	6 %
Nécrose au point d'injection*	0 %	5 %
Kyste	2 %	4 %
Nécrose	0 %	2 %
Tentative de suicide	0 %	2 %
**Appareil cardiovasculaire**		
Migraine	7 %	12 %
Palpitations*	2 %	8 %
Hypertension	2 %	7 %
Tachycardie	3 %	6 %
Troubles vasculaires périphériques	2 %	5 %
Hémorragie	1 %	3 %
**Appareil digestif**		
Diarrhée	29 %	35 %
Constipation	18 %	24 %
Vomissements	19 %	21 %
Troubles gastro-intestinaux	3 %	6 %
**Système endocrinien**		
Goitre	0 %	2 %
**Sang et système lymphatique**		
Lymphocytes < 1 500/mm^3	67 %	82 %
N^{bre} absolu de polynucléaires neutrophiles < 1 500/mm^{3*}	6 %	18 %
Leucocytes < 3 000/mm^{3*}	5 %	16 %
Adénopathies	11 %	14 %
**Troubles métaboliques et nutritionnels**		
ALT (SGPT) > 5 fois la valeur de départ*	6 %	19 %
Glucose < 55 mg/dL	13 %	15 %
Bilirubine totale > 2,5 fois la valeur de départ	2 %	6 %
Protéinurie > 1+	3 %	5 %
AST (SGOT) > 5 fois la valeur de départ*	0 %	4 %
Gain pondéral	0 %	4 %
Perte pondérale	2 %	4 %

TABLEAU 4<br>Effets indésirables et résultats de laboratoire anormaux (suite)		
**Effets indésirables**	**Placebo<br>n = 123**	**0,25 mg<br>(8 MUI)<br>n = 124**
**Appareil locomoteur**		
Myalgie*	28 %	44 %
Myasthénie	10 %	13 %
**Système nerveux**		
Étourdissements	28 %	35 %
Hypertonie	24 %	26 %
Dépression	24 %	25 %
Anxiété	13 %	15 %
Nervosité	5 %	8 %
Somnolence	3 %	6 %
Confusion	2 %	4 %
Troubles de la parole	1 %	3 %
Convulsions	0 %	2 %
Hyperkinésie	0 %	2 %
Amnésie	0 %	2 %
**Appareil respiratoire**		
Sinusite	26 %	36 %
Dyspnée*	2 %	8 %
Laryngite	2 %	6 %
**Peau et téguments**		
Sudation*	11 %	23 %
Alopécie	2 %	4 %
**Organes sensoriels**		
Conjonctivite	10 %	12 %
Troubles de la vision	4 %	7 %
**Appareil génito-urinaire**		
Dysménorrhée	11 %	18 %
Troubles menstruels*	8 %	17 %
Métrorragie	8 %	15 %
Cystite	4 %	8 %
Douleur aux seins	3 %	7 %
Ménorragie	3 %	6 %
Miction impérieuse	2 %	4 %
Mastopathie fibrokystique	1 %	3 %
Néoplasme du sein	0 %	2 %

* Association statistiquement significative avec le traitement par BETASERON ($p < 0,05$)

Il faut noter que les valeurs citées dans le tableau 4 ne peuvent servir à prédire la fréquence des effets indésirables dans la pratique médicale courante, puisque les caractéristiques des patients et d'autres facteurs diffèrent de ceux qui prévalaient au cours des études cliniques. Par contre, le médecin peut se servir des valeurs citées pour estimer la contribution relative du médicament à la fréquence d'effets indésirables dans la population étudiée.

2. SEP progressive-secondaire

Le tableau 5 présente l'incidence des effets indésirables survenus chez au moins 2 % des patients ayant reçu 8 MUI de BETASERON ou un placebo pendant une période maximale de trois ans ou celle des effets indésirables survenus à une fréquence supérieure d'au moins 2 % dans le groupe BETASERON par rapport au groupe placebo, dans le cadre de l'étude sur la SEP progressive-secondaire. Les effets indésirables observés dans les groupes BETASERON et placebo dont la fréquence était significative figurent également au tableau 5 ($p < 0,05$).

Tableau 5

Incidence des effets indésirables survenus chez ≥ 2 % des patients ou à une fréquence > 2 % (BETASERON *vs* placebo) dans l'étude sur la SEP progressive-secondaire

Effets indésirables	Placebo<br>n = 358	0,25 mg<br>(8 MUI)<br>n = 360
Corps entier		
Asthénie	58 %	63 %
Syndrome grippal*	40 %	61 %
Douleur	25 %	31 %
Fièvre*	13 %	40 %
Dorsalgie	24 %	26 %
Blessure accidentelle	17 %	14 %
Frissons*	7 %	23 %
Douleur aux extrémités	12 %	14 %
Infection	11 %	13 %
Douleur abdominale*	6 %	11 %
Malaises	5 %	8 %
Douleur dans le cou	6 %	5 %
Abcès*	2 %	4 %
Résultats de laboratoire anormaux	1 %	3 %
Réaction allergique	3 %	2 %
Frissons et fièvre*	0 %	3 %
Douleur thoracique	2 %	1 %
Appareil cardiovasculaire		
Vasodilatation	4 %	6 %
Troubles vasculaires périphériques	5 %	5 %
Douleur thoracique	4 %	5 %
Migraine	3 %	4 %
Hypotension	4 %	2 %
Hypertension*	2 %	4 %
Palpitations	3 %	2 %
Syncope	3 %	2 %
Hémorragie	2 %	2 %
Tachycardie	1 %	2 %
Appareil digestif		
Nausées	13 %	13 %
Constipation	12 %	12 %
Diarrhée	10 %	7 %
Gastro-entérite	5 %	6 %
Vomissements	6 %	4 %
Dysphagie	5 %	4 %
Troubles gastro-intestinaux	5 %	4 %

**Tableau 5**<br>**Incidence des effets indésirables survenus chez ≥ 2 % des patients ou à une fréquence > 2 % (BETASERON *vs* placebo) dans l'étude sur la SEP progressive-secondaire** (suite)		
**Effets indésirables**	**Placebo**<br>**n = 358**	**0,25 mg (8 MUI)**<br>**n = 360**
Appareil digestif (suite)		
Problème dentaire	4 %	4 %
Dyspepsie	4 %	4 %
Anorexie	2 %	4 %
Incontinence fécale	3 %	2 %
Test de la fonction hépatique anormal	1 %	3 %
Gastrite	2 %	2 %
Flatulences	1 %	3 %
Maux de gorge	1 %	2 %
Colite	2 %	0 %
Douleur gastro-intestinale	0 %	2 %
Gingivite	0 %	2 %
Sang et système lymphatique		
Leucopénie*	5 %	10 %
Anémie	5 %	2 %
Ecchymoses	2 %	1 %
Lymphadénopathie	1 %	3 %
Point d'injection		
Réaction au point d'injection*	10 %	46 %
Inflammation au point d'injection*	4 %	48 %
Douleur au point d'injection	5 %	9 %
Nécrose au point d'injection*	0 %	5 %
Hémorragie au point d'injection	2 %	2 %
Troubles métaboliques et nutritionnels		
Œdème périphérique	7 %	7 %
Perte pondérale	3 %	2 %
Augmentation de la SGPT	2 %	2 %
Hypercholestérolémie	2 %	1 %
Appareil locomoteur		
Myasthénie	40 %	39 %
Arthralgie	20 %	20 %
Myalgie*	9 %	23 %
Fracture (non spontanée)	5 %	3 %
Crampes musculaires	3 %	3 %
Fracture spontanée	3 %	3 %
Arthrite	1 %	2 %
Troubles articulaires	1 %	2 %

**Tableau 5**<br>**Incidence des effets indésirables survenus chez ≥ 2 % des patients ou à une fréquence > 2 % (BETASERON *vs* placebo) dans l'étude sur la SEP progressive-secondaire** (suite)		
**Effets indésirables**	**Placebo**<br>**n = 358**	**0,25 mg**<br>**(8 MUI)**<br>**n = 360**
Système nerveux		
Céphalées	41 %	47 %
Neuropathie	41 %	38 %
Paresthésies	39 %	35 %
Hypertonie*	31 %	41 %
Démarche anormale	34 %	34 %
Dépression	31 %	27 %
Ataxie	23 %	19 %
Étourdissements	14 %	14 %
Absence de coordination	13 %	11 %
Insomnie	8 %	12 %
Vertiges	12 %	8 %
Labilité émotionnelle	11 %	8 %
Paralysie	10 %	8 %
Somnolence	8 %	8 %
Tremblements	9 %	6 %
Augmentation de la sudation	6 %	6 %
Névralgie	7 %	5 %
Troubles de la motricité	6 %	5 %
Troubles du sommeil	5 %	6 %
Anxiété	5 %	6 %
Hypoesthésie	4 %	6 %
Nervosité	3 %	4 %
Troubles de la parole	5 %	2 %
Dysarthrie	4 %	2 %
Paralysie spastique	1 %	3 %
Convulsions	2 %	2 %
Hyperesthésie	2 %	2 %
Amnésie	3 %	1 %
Sécheresse de la bouche	2 %	1 %
Hémiplégie	2 %	1 %
Raisonnement anormal	2 %	1 %
Myoclonie	2 %	0 %
Appareil respiratoire		
Rhinite	32 %	28 %
Pharyngite	20 %	16 %
Bronchite	12 %	9 %
Augmentation de la toux	10 %	5 %
Sinusite	6 %	6 %
Pneumonie	5 %	5 %
Dyspnée	2 %	3 %
Infection des voies respiratoires supérieures	2 %	3 %
Asthme	2 %	1 %
Modification de la voix	2 %	1 %

**Tableau 5**<br><br>**Incidence des effets indésirables survenus chez ≥2 % des patients ou à une fréquence > 2 % (BETASERON *vs* placebo) dans l'étude sur la SEP progressive-secondaire** (suite)		
**Effets indésirables**	**Placebo**<br>**n = 358**	**0,25 mg (8 MUI)**<br>**n = 360**
Peau et téguments		
Éruption cutanée*	12 %	20 %
Prurit	6 %	6 %
Troubles cutanés	4 %	4 %
Eczéma	4 %	2 %
Herpès	2 %	3 %
Alopécie	2 %	2 %
Acné	2 %	2 %
Peau sèche	3 %	1 %
Hématome sous-cutané	3 %	1 %
Douleur aux seins	2 %	1 %
Zona	2 %	1 %
Séborrhée	2 %	1 %
Organes sensoriels		
Vision anormale	15 %	11 %
Amblyopie	10 %	7 %
Diplopie	9 %	7 %
Douleurs oculaires	5 %	4 %
Otite moyenne	3 %	2 %
Conjonctivite	3 %	2 %
Troubles oculaires	2 %	3 %
Surdité	3 %	1 %
Névrite optique	2 %	2 %
Troubles auriculaires	2 %	1 %
Acouphènes	2 %	1 %
Appareil génito-urinaire		
Infection des voies urinaires	25 %	22 %
Incontinence urinaire	15 %	8 %
Troubles des voies urinaires	10 %	7 %
Cystite	9 %	7 %
Miction impérieuse	7 %	8 %
Troubles menstruels	13 %	9 %
Augmentation de la fréquence urinaire	5 %	6 %
Métrorragie	6 %	12 %
Rétention urinaire	6 %	4 %
Vaginite	4 %	3 %
Aménorrhée	4 %	3 %
Dysurie	2 %	2 %
Impotence	4 %	7 %
Ménopause	4 %	2 %
Ménorragie	4 %	2 %
Nycturie	1 %	2 %
Candidose vaginale	2 %	2 %
Douleur rénales	2 %	0 %
Pyélonéphrite	0 %	2 %
Troubles de la prostate	1 %	2 %

* Association statistiquement significative avec le traitement par BETASERON ($p < 0,05$)

Soixante-quatorze (74) patients ont interrompu leur traitement en raison d'effets indésirables (23 dans le groupe placebo et 51 dans le groupe BETASERON). On a noté une association statistiquement significative entre les réactions au point d'injection et la fin prématurée du traitement dans le groupe BETASERON, comparativement au groupe placebo ($p < 0{,}05$). Les effets indésirables les plus fréquents qui ont entraîné l'abandon étaient liés au système nerveux, et la dépression (sept cas dans le groupe placebo et onze dans le groupe BETASERON) était l'effet le plus courant.

On a observé une élévation du taux d'ALT (SGPT) (> 5 fois la valeur de départ) chez un nombre significativement plus élevé de patients recevant le traitement actif (14,4 % *vs* 4,7 % dans le groupe placebo). On a également noté une élévation du taux d'AST (SGOT) et du taux de gamma-GT dans le groupe BETASERON pendant toute la durée de l'étude. Dans le groupe BETASERON, la plupart des anomalies relatives au taux d'ALT (SGPT) se sont résorbées spontanément au cours du traitement alors que d'autres ont disparu lorsqu'on a diminué la dose ou interrompu le traitement temporairement.

On a observé une lymphopénie (< 1 500/mm^3) chez 90,9 % des patients du groupe BETASERON comparativement à 74,3 % des patients du groupe placebo, de même qu'une neutropénie (< 1 400/mm^3) chez 18,0 % des patients du groupe BETASERON et 5,1 % des patients du groupe placebo.

Les paragraphes suivants font état d'autres effets indésirables observés chez 1 440 patients au cours des évaluations pré-commercialisation de BETASERON administré à diverses doses. La plupart de ces effets ayant été observés au cours d'études ouvertes et non contrôlées, le lien causal entre BETASERON et leur apparition ne peut être formellement établi.

Corps entier : abcès, adénome, réaction anaphylactoïde, ascite, cellulite, hernie, hydrocéphalie, hypothermie, infection, péritonite, photosensibilité, sarcome, septicémie et choc.

Appareil cardiovasculaire : angine de poitrine, arythmie, fibrillation auriculaire, cardiomégalie, arrêt cardiaque, hémorragie cérébrale, ischémie cérébrale, endocardite, insuffisance cardiaque, hypotension, infarctus du myocarde, épanchement péricardique, hypotension orthostatique,

embolie pulmonaire, angiome stellaire, hémorragie sous-arachnoïdienne, syncope, thrombophlébite, thrombose, varices, angiospasme, élévation de la pression veineuse, extrasystoles ventriculaires et fibrillation ventriculaire.

Appareil digestif : stomatite aphteuse, cardiospasme, chéilite, cholécystite, cholélithiase, ulcère duodénal, sécheresse de la bouche, entérite, œsophagite, fécalome, incontinence fécale, flatulences, gastrite, hémorragie digestive, gingivite, glossite, hématémèse, néoplasie hépatique, hépatite, hépatomégalie, iléus, ptyalisme, occlusion intestinale, mélæna, nausées, leucoplasie buccale, candidose buccale, pancréatite, abcès péridentaire, proctite, hémorragie rectale, hypertrophie des glandes salivaires, ulcère gastrique et ténesme.

Système endocrinien : syndrome de Cushing, diabète insipide, diabète sucré, hypothyroïdie et taux anormal de vasopressine.

Sang et système lymphatique : leucémie lymphoïde chronique, hémoglobine < 9,4 g/100 mL, pétéchies, plaquettes < 75 000/mm^3 et splénomégalie.

Troubles métaboliques et nutritionnels : intolérance à l'alcool, phosphatase alcaline > 5 fois la valeur de départ, azote uréique sanguin > 40 mg/dL, calcium > 11,5 mg/dL, cyanose, œdème, glucose > 160 mg/dL, glycosurie, réaction hypoglycémique, hypoxie, cétose et soif.

Appareil locomoteur : arthrite, arthrose, bursite, crampes dans les jambes, atrophie musculaire, myopathie, myosite, ptose et ténosynovite.

Système nerveux : démarche anormale, syndrome cérébral aigu, agitation, apathie, aphasie, ataxie, œdème cérébral, syndrome cérébral chronique, coma, délire, illusions, démence, dépersonnalisation, diplopie, dystonie, encéphalopathie, euphorie, paralysie faciale, pied tombant, hallucinations, hémiplégie, hypoalgésie, hyperesthésie, absence de coordination, hypertension intracrânienne, baisse de la libido, exaltation maniaque, méningite, névralgie, neuropathie, névrose, nystagmus, crise oculogyre, ophtalmoplégie, œdème papillaire, paralysie, réactions paranoïaques, psychose, baisse des réflexes, stupeur, hématome sous-dural, torticolis, tremblements et rétention urinaire.

Appareil respiratoire : apnée, asthme, atélectasie, carcinome pulmonaire, hémoptysie, hoquet, hyperventilation, hypoventilation, pneumonie interstitielle, œdème pulmonaire, épanchement pleural, pneumonie et pneumothorax.

Peau et téguments : dermatite de contact, érythème noueux, dermatite exfoliante, furonculose, hirsutisme, leucodermie, dermatite lichénoïde, rash maculopapuleux, psoriasis, séborrhée, néoplasme bénin de la peau, cancer de la peau, hypertrophie de la peau, nécrose de la peau, ulcère cutané, urticaire et rash vésiculobulleux.

Organes sensoriels : blépharite, cécité, surdité, œil sec, douleurs à l'oreille, iritis, kératoconjonctivite, mydriase, otite externe, otite moyenne, parosmie, photophobie, rétinite, perte ou altération du goût et altération du champ visuel.

Appareil génito-urinaire : anurie, balanite, engorgement mammaire, cervicite, épididymite, gynécomastie, hématurie, impuissance, calcul rénal, insuffisance rénale, néphropathie tubulaire, leucorrhée, néphrite, nycturie, oligurie, polyurie, salpingite, urétrite, incontinence urinaire, augmentation du volume d'un fibromyome utérin, néoplasme utérin et hémorragie vaginale.

POSOLOGIE ET ADMINISTRATION

(POUR ADMINISTRATION SOUS-CUTANÉE SEULEMENT)

BETASERON (interféron bêta-1b) ne doit être prescrit que par des médecins qui ont de l'expérience dans le diagnostic et la prise en charge de la sclérose en plaques (ou après consultation de tels médecins).

On recommande l'injection sous-cutanée de 0,25 mg (8 MUI) de BETASERON (interféron bêta-1b) tous les deux jours, à la fois pour les patients atteints de SEP rémittente et pour ceux qui sont atteints de SEP progressive-secondaire. Quelques données sur l'activité d'une dose plus faible dans la SEP rémittente sont présentées plus haut (voir **ACTION ET PHARMACOLOGIE CLINIQUE, Études cliniques**).

Dans l'étude sur la SEP progressive-secondaire, les patients ont commencé le traitement avec une demi-dose (4 MUI administrées par voie sous-cutanée tous les deux jours). Au bout de deux semaines, ils sont passés à la dose recommandée de 8 MUI (administrées par voie sous-cutanée tous les deux jours).

L'efficacité du traitement au-delà de deux ans n'a pas été démontrée de façon substantielle dans le cas de la sclérose en plaques rémittente. Dans le cas de la sclérose en plaques progressive-secondaire, on ne dispose pas de données sur l'innocuité et l'efficacité au-delà de trois ans.

Pour reconstituer BETASERON lyophilisé en vue de l'injection, utiliser l'adaptateur de flacon pour injecter tout le contenu de la seringue préremplie de diluant (chlorure de sodium à 0,54 %) dans le flacon de BETASERON. Faire rouler délicatement le flacon pour dissoudre le produit complètement ; ne pas agiter. La solution reconstituée doit faire l'objet d'un examen visuel et n'être utilisée que si elle est limpide et n'a pas changé de couleur. Après reconstitution avec le diluant, 1 mL de solution renferme 0,25 mg (8 MUI) d'interféron bêta-1b, 13 mg d'albumine humaine USP et 13 mg de mannitol USP.

Retirer du flacon 1 mL de la solution reconstituée au moyen de la seringue munie d'une aiguille d'un demi-pouce de calibre 27 utilisée pour ajouter le diluant et injecter la solution par voie sous-cutanée. Les endroits indiqués pour l'auto-injection sont l'abdomen, les fesses et les cuisses. Tous les éléments sont conçus pour une seule dose ; toute portion non utilisée doit être jetée. (Voir la section **INFORMATION À L'INTENTION DES PATIENTS**, **DIRECTIVES POUR L'AUTO-INJECTION**.)

INFORMATION PHARMACEUTIQUE

Dénomination commune :	interféron bêta-1b (USAN)
Masse moléculaire :	environ 18 500 daltons
Forme physique :	poudre stérile, lyophilisée
Composition (par flacon) :	0,3 mg (9,6 MUI) d'interféron bêta-1b<br>15 mg d'albumine humaine USP<br>15 mg de mannitol USP<br>L'activité spécifique de BETASERON est d'environ 32 millions d'unités internationales par mg (MUI/mg) d'interféron bêta-1b. La mesure unitaire provient de la comparaison de l'activité antivirale du produit à celle de l'interféron bêta humain recombiné utilisé comme étalon de référence par l'Organisation mondiale de la santé (OMS). Avant 1993, la teneur était déterminée à partir d'un étalon analytique différent selon lequel 54 MUI correspondaient à 0,3 mg d'interféron bêta-1b.
**Stabilité (avant reconstitution) :**	Conserver à la température ambiante, entre 15 et 30 °C (59 et 86 °F). Ne pas congeler. Ne pas utiliser après la date de péremption indiquée sur l'étiquette du flacon de Betaseron et de la seringue préremplie.
**Stabilité (après reconstitution) :**	Le produit reconstitué ne contient aucun agent de conservation. S'il n'est pas utilisé immédiatement, il doit être réfrigéré entre

2 et 8 °C (36 et 46 °F) et **utilisé dans les trois heures suivant la reconstitution**. Ne pas congeler.

PRÉSENTATION

BETASERON (interféron bêta-1b) se présente en flacons à usage unique, sous forme de poudre lyophilisée renfermant 0,3 mg (9,6 MUI) d'interféron bêta-1b, 15 mg d'albumine humaine USP et 15 mg de mannitol USP. BETASERON est fourni dans une boîte en carton contenant 15 trousses à usage unique. Chaque trousse à usage unique contient le matériel nécessaire pour préparer et injecter une seule dose de BETASERON : 1 flacon de médicament, 1 seringue préremplie de diluant (1,2 mL de chlorure de sodium à 0,54 %), 1 adaptateur de flacon muni d'une aiguille d'un demi-pouce de calibre 27 et 3 tampons d'alcool.

INFORMATION À L'INTENTION DES PATIENTS

BETASERON (interféron bêta-1b) doit être utilisé sous la surveillance d'un médecin. Votre médecin, ou un autre professionnel de la santé qu'il aura délégué, doit vous expliquer comment préparer BETASERON et vous montrer la technique d'auto-injection. Ne commencez pas le traitement sans avoir suivi une formation sur BETASERON.

Le traitement par BETASERON doit être suivi tel que prescrit par votre médecin. Toutefois, si vous oubliez une injection, administrez-vous le médicament aussitôt que vous y pensez. Rappelez-vous les renseignements suivants :

- **Avant la préparation de la solution :**
  Conservez BETASERON à la température ambiante, entre 15 et 30 °C (59 et 86 °F). Ne le congelez pas.

  Après la préparation de la solution :
  S'il n'est pas utilisé immédiatement, le produit doit être réfrigéré et **utilisé dans les trois heures suivant la reconstitution**. Ne le congelez pas.

- Gardez les seringues et les aiguilles hors de la portée des enfants. Ne réutilisez pas les seringues ni les aiguilles. Jetez les seringues et les aiguilles utilisées dans un récipient destiné à cette fin.

- **Grossesse :** Les femmes enceintes ou celles qui tentent de le devenir ne doivent pas prendre BETASERON. Pendant le traitement, les femmes en âge de procréer doivent utiliser une méthode contraceptive. Une femme qui envisage une grossesse au cours du traitement doit en parler à son médecin. En cas de grossesse, il faut cesser de prendre le médicament et appeler son médecin sans tarder.

- Les réactions au point d'injection sont fréquentes, notamment : rougeur, douleur, enflure et changement de coloration de la peau. Plus rarement, il peut se produire une nécrose au point d'injection (lésion de la peau et des tissus en dessous). Pour diminuer le risque de réaction, il faut changer de partie du corps à chaque injection et attendre une semaine avant de refaire une injection au même endroit. Ne vous donnez pas d'injections aux endroits où la peau est sensible, rouge ou dure. Ne choisissez pas les endroits où vous sentez une bosse, un creux ou de la douleur, ou encore les endroits où la peau a changé de couleur. Si vous découvrez l'une de ces particularités, avisez votre médecin ou un autre professionnel de la santé. Si vous constatez une plaie ou un écoulement de liquide à l'endroit de l'injection, consultez votre médecin.

- On observe aussi fréquemment des symptômes apparentés à la grippe (symptômes pseudo-grippaux) : fièvre, frissons, sueurs, fatigue et courbatures. Ces effets peuvent être moins incommodants si BETASERON est pris au coucher.

- Des symptômes de dépression, dont des tentatives de suicide, ont aussi été signalés par certains patients. Si vous ressentez l'un ou l'autre de ces symptômes, communiquez avec votre médecin sans tarder.

- Comme tout autre médicament, BETASERON peut entraîner des effets indésirables. Consultez votre médecin pour tout problème, même si vous ne pensez pas qu'il soit causé par BETASERON.

DIRECTIVES POUR L'AUTO-INJECTION

CONSEILS DE SÉCURITÉ

- N'utilisez que le matériel fourni dans votre trousse BETASERON.
- N'utilisez que le diluant de la seringue préremplie.
- Lavez-vous les mains soigneusement à l'eau et au savon avant de commencer.
- Gardez les éléments stériles. Ne touchez pas l'aiguille, l'extrémité perforante de l'adaptateur de flacon ou le dessus du flacon nettoyé.
- Assurez-vous que les éléments de la trousse n'ont été ni ouverts, ni endommagés.
- Ne réutilisez pas du matériel dont l'emballage a été ouvert. Jetez toute portion inutilisée de BETASERON et de diluant.
- Jetez les seringues et les aiguilles utilisées dans le collecteur d'aiguilles.

ÉTAPE 1 : CHOIX D'UN POINT D'INJECTION

BETASERON doit être injecté dans le tissu sous-cutané (entre la couche de gras, qui se trouve sous la peau, et le muscle). Les meilleures parties du corps pour l'injection sont des parties molles, loin des articulations.

■ **Choisissez un point d'injection parmi les parties suivantes :**

• Abdomen, au-dessus de la taille (à au moins 5 cm à gauche ou à droite du nombril)
• Cuisse droite (à au moins 5 cm au-dessus du genou et 5 cm au-dessous de l'aine)
• Cuisse gauche (à au moins 5 cm au-dessus du genou et 5 cm au-dessous de l'aine)
• Fesse gauche (partie supérieure, vers l'extérieur)
• Fesse droite (partie supérieure, vers l'extérieur)

■ Changez de partie du corps chaque fois que vous vous injectez le médicament pour permettre à la peau au point d'injection de récupérer. Cela vous aidera à prévenir les réactions au point d'injection.

■ Attendez au moins une semaine avant de faire une autre injection au même endroit.

■ Ne choisissez pas les endroits où vous sentez une bosse, un creux ou de la douleur, ou encore les endroits où la peau a changé de couleur. Si vous découvrez l'une de ces particularités, avisez votre médecin ou un autre professionnel de la santé.

■ Notez le jour de l'injection et le point d'injection que vous avez utilisé. Servez-vous de l'agenda fourni avec la trousse BETASERON.

ÉTAPE 2 : PRÉPARATION INITIALE

1. Placez la trousse BETASERON à usage unique sur une surface plane et propre dans un endroit bien éclairé. Vérifiez qu'elle contient :
   - 1 flacon de BETASERON
   - 1 seringue préremplie de diluant
   - 3 tampons d'alcool
   - 1 adaptateur de flacon muni d'une aiguille d'un demi-pouce de calibre 27 sous emballage-coque

2. Lavez-vous soigneusement les mains à l'eau et au savon.

3. Ouvrez la trousse en tirant sur la partie pelable.

4. Sortez tout le contenu.

   ***NOTE** : Assurez-vous que l'emballage-coque de l'adaptateur de flacon est scellé et que le capuchon en caoutchouc de la seringue de diluant est bien en place.*

5. Vérifiez la date de péremption sur le flacon de BETASERON et la seringue préremplie de diluant.

6. Retournez la trousse à usage unique, placez le flacon de BETASERON dans le creux (porte-flacon) au centre de la trousse et placez la seringue préremplie de diluant dans la rainure en forme de U.

ÉTAPE 3 : RECONSTITUTION DE BETASERON

1. **Retirez** le flacon de BETASERON du porte-flacon et **enlevez** la capsule protectrice.
2. **Replacez** le flacon de BETASERON dans le porte-flacon.

3. **Nettoyez** le dessus du flacon avec un tampon d'alcool en essuyant dans une seule direction. ***NOTE** : Laissez le tampon sur le dessus du flacon jusqu'à l'étape 5.*

4. **Ouvrez** l'emballage pelable de l'adaptateur de flacon, mais ne sortez pas l'adaptateur. ***NOTE** : Assurez-vous de ne pas toucher à l'adaptateur de flacon afin de maintenir sa stérilité.*

5. **Retirez** le tampon d'alcool sur le dessus du flacon de BETASERON. **Placez** l'adaptateur de flacon, toujours dans son emballage-coque, sur le flacon de BETASERON en poussant jusqu'à ce qu'il perce le bouchon en caoutchouc du flacon et se mette en place (Figure 1). Retirez l'emballage-coque de l'adaptateur de flacon.

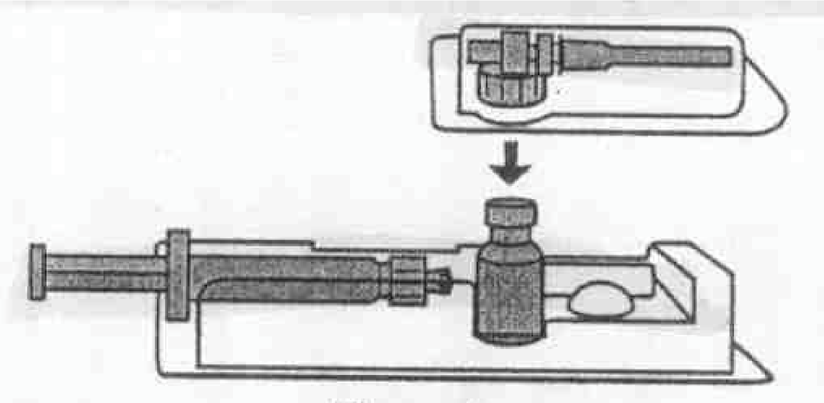

Figure 1

6. **Retirez** le capuchon en caoutchouc de la seringue de diluant en tournant et en tirant. Jetez-le.

7. **Vissez** la seringue à l'adaptateur de flacon en tournant dans le sens des aiguilles d'une montre et en resserrant avec précaution de façon à former un ensemble seringue/adaptateur (figure 2).

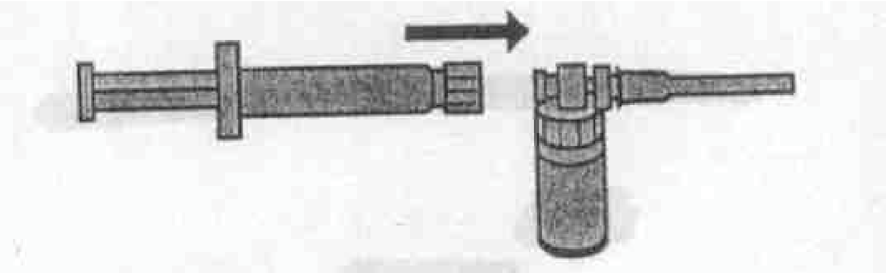

Figure 2

8. Il est important de **<u>pousser</u>** **<u>lentement</u>** le piston de la seringue de diluant jusqu'au bout. Cela fera passer tout le diluant dans le flacon de BETASERON (figure 3). Le piston relâché peut retourner à sa position originale.

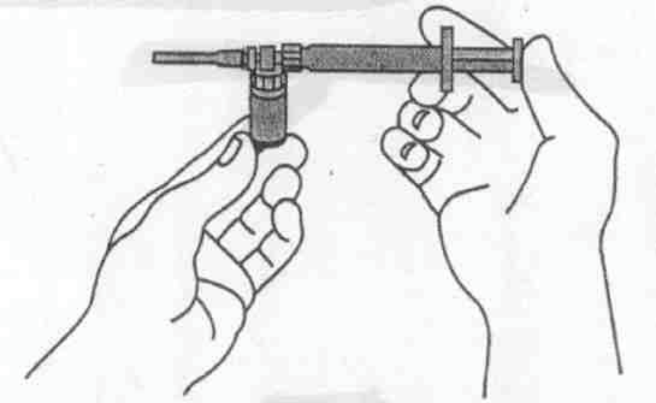

Figure 3

9. En gardant l'ensemble seringue/adaptateur fixé au flacon, **<u>faites tourner</u>** **<u>doucement</u>** ce dernier pour dissoudre complètement le médicament. (**NE L'AGITEZ PAS.**)

10. **<u>Examinez</u>** attentivement la solution. Elle devrait être claire et exempte de particules.
__NOTE__ : Si le mélange contient des particules ou est décoloré, jetez-le et recommencez. Il peut se produire de la mousse au cours de la reconstitution ou si vous avez fait tourner ou agité le flacon trop violemment. Dans ce cas, laissez reposer la solution jusqu'à ce que la mousse disparaisse.

ÉTAPE 4 : PRÉPARATION DE L'INJECTION

1. **Poussez** le piston et maintenez-le en position, puis retournez l'ensemble seringue/adaptateur/flacon à l'envers (c'est-à-dire à 180 degrés) de façon que le flacon soit sur le dessus. La seringue reste à l'horizontale (figure 4).

2. **Tirez** lentement le piston pour faire passer tout le contenu du flacon de BETASERON dans la seringue (figure 4).
   NOTE : S'il vous est impossible de prélever 1 mL de solution limpide dans le flacon, jetez le flacon et la seringue et recommencez.

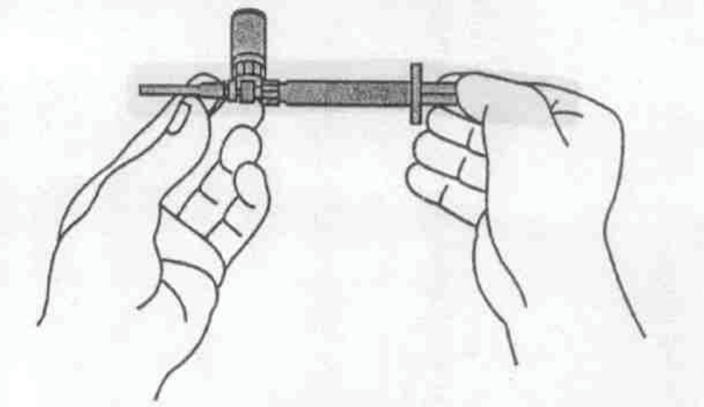

Figure 4

3. **Tournez** l'ensemble seringue/adaptateur/flacon de façon que l'aiguille pointe vers le haut. Expulsez les bulles d'air (vous pouvez tapoter **légèrement** la paroi extérieure de la seringue pour les libérer) en poussant lentement le piston jusqu'à la marque de 1 mL sur la seringue.
   NOTE : Si une quantité trop élevée de solution est expulsée dans le flacon, répétez les étapes 1, 2 et 3.

4. **Retirez** l'adaptateur de flacon et le flacon de la seringue en tournant l'adaptateur de flacon de la façon indiquée à la figure 5. Cela détachera l'adaptateur de flacon, avec le flacon, de la seringue, mais laissera l'aiguille sur la seringue (figure 5).

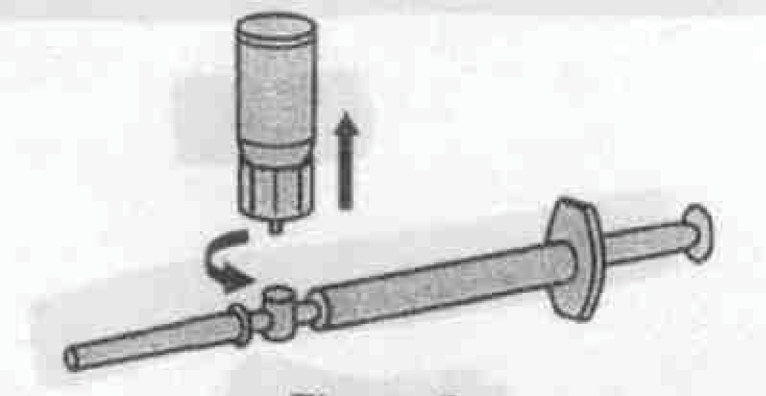

Figure 5

5. **Vous avez maintenant reconstitué votre BETASERON et êtes prêt pour l’injection.**

L’injection devrait être administrée immédiatement après sa préparation. Si vous êtes dans l’impossibilité de la faire immédiatement, vous pouvez réfrigérer le médicament dans la seringue et procéder à l’injection dans les trois heures. Ne le congelez pas.

ÉTAPE 5 : INJECTION DE BETASERON

1. **Désinfectez** le point d'injection avec un tampon d'alcool, avec un mouvement circulaire à partir du centre vers l'extérieur ; laissez sécher.

2. **Jetez** le tampon.

3. **Retirez** le capuchon protecteur de l'aiguille en le tirant sans le tourner.

4. **Pincez** légèrement la peau de chaque côté du point d'injection pour qu'elle soit légèrement surélevée.

5. **Enfoncez** l'aiguille dans la peau à un angle de 90°, d'un geste rapide et ferme.

6. **Injectez** le médicament en poussant lentement et régulièrement sur le piston jusqu'à ce que la seringue soit vide.

7. **Retirez** l'aiguille de la peau.

8. **Massez** doucement le point d'injection avec un nouveau tampon d'alcool.

9. **Jetez** la seringue dans le collecteur d’aiguilles.

10. **Jetez** tous les autres éléments de la trousse.

PHARMACOLOGIE

Pharmacocinétique : Étant donné que les concentrations sériques d'interféron bêta-1b sont faibles, voire non décelables, après administration sous-cutanée de 0,25 mg (8 MUI) ou moins de BETASERON (interféron bêta-1b), on ne dispose d'aucune donnée pharmacocinétique sur l'administration de la dose recommandée de BETASERON chez les patients atteints de SEP. Après administration de doses uniques ou de doses quotidiennes multiples de 0,5 mg (16 MUI) de BETASERON à des volontaires sains (n = 12), les concentrations sériques d'interféron bêta-1b étaient généralement inférieures à 100 UI/mL. Les concentrations sériques maximales d'interféron bêta-1b étaient observées de une à huit heures après l'administration, la concentration sérique maximale moyenne étant de 40 UI/mL. Avec une dose totale de 0,5 mg (16 MUI) de BETASERON administrée en deux injections sous-cutanées en deux endroits, la biodisponibilité était d'environ 50 %.

L'administration intraveineuse de BETASERON (de 0,006 mg [0,2 MUI] à 2,0 mg [64 MUI]) a donné lieu à des profils pharmacocinétiques comparables chez des volontaires sains (n = 12) et chez des patients atteints de maladies autres que la SEP (n = 142). Chez des patients recevant des doses uniques allant jusqu'à 2,0 mg (64 MUI), l'augmentation de la concentration sérique était proportionnelle à la dose. La clairance sanguine moyenne a varié de 9,4 $mL/min \cdot kg^{-1}$ à 28,9 $mL/min \cdot kg^{-1}$, sans rapport avec la dose administrée. La demi-vie d'élimination terminale moyenne a varié de 8,0 minutes à 4,3 heures, et le volume de distribution à l'état d'équilibre, de 0,25 L/kg à 2,88 L/kg. L'administration intraveineuse d'interféron bêta-1b trois fois par semaine pendant deux semaines n'a conduit à aucune accumulation sérique. Les paramètres pharmacocinétiques après administration intraveineuse d'une dose unique et de doses multiples de BETASERON étaient comparables.

TOXICOLOGIE

Carcinogenèse : Le potentiel carcinogène de BETASERON (interféron bêta-1b) a été évalué par l'étude de ses effets sur la transformation morphologique de la lignée cellulaire mammifère BALBc-3T3. Aucune augmentation significative de la fréquence de transformation n'a été enregistrée. On ne dispose d'aucune donnée sur le potentiel carcinogène de BETASERON chez l'animal ou chez l'être humain.

Mutagénicité : BETASERON n'a montré aucun signe de mutagénicité lors de l'évaluation de la génotoxicité par le test d'Ames chez la bactérie avec activation métabolique.

RÉFÉRENCES

Blaschke TF, Horning SJ, Merigan TC *et al.* Recombinant $ß_{ser}$-interferon inhibits antipyrine clearance in man. *Clinical Research* 1985; 33(1): 19A.

Carlin JM, Borden EC, Sondel PM *et al*. Biologic response modifier-induced indoleamine 2,3-dioxygenase activity in human peripheral blood mononuclear cell cultures. *J Immunol* 1987; 139 (7): 2414-2418.

Colby CB, Inoue M, Thompson M, *et al.* Immunologic differentiation between *E. coli* and CHO cell-derived recombinant and natural human ß-interferons. *J Immunol* 1984; 133(6): 3091-3095.

De Maeyer E, De Maeyer-Guignard J; The interferon gene family. *In*: De Maeyer E, De Maeyer-Guignard J. Interferons and other regulatory cytokines. New York, Wiley and Sons, Inc. 1988; 5-38.

European Study Group on Interferon β-1b in secondary progressive MS. Placebo-controlled multicentre randomized trial of interferon β-1b in treatment of secondary progressive multiple sclerosis. *The Lancet*, 1998; 352: 1491-1497.

Goldstein D, Sielaff KM, Storer BE, *et al.* Human biologic response modification by interferon in the absence of measurable serum concentrations: a comparative trial of subcutaneous and intravenous interferon-ß serine. *J Natl Cancer Inst* 1989; 81(14): 1061-1068.

Knobler RL, Greenstein JI, Johnson KP *et al.* Systemic recombinant human interferon-ß treatment of relapsing-remitting multiple sclerosis: pilot study analysis and six-year follow-up. *J Interferon Res* 1993; 13: 333-340.

Lengyel P. Biochemistry of interferons and their actions. *Ann Rev Biochem* 1982; 51: 251-282.

Paty DW, Li DKB *et al.* Interferon beta-1b is effective in relapsing-remitting multiple sclerosis. *Neurology* 1993; 43: 662-667.

Pestka S, Langer JA, Zoon KC *et al.* Interferons and their actions. *Ann Rev Biochem* 1987; 56: 727-777.

Poser CM, Paty DW, Scheinberg L, *et al.* New diagnostic criteria for multiple sclerosis: guidelines for research protocols. *Ann Neurol* 1983; 13(3): 227-231.

Rosenblum MG, Yung WKA, Kelleher PJ, Ruzicka F, *et al*. Growth inhibitory effects of interferon-ß but not interferon-α on human glioma cells: correlation of receptor binding, 2',5'-oligoadenylate synthetase and protein kinase activity. *J Interferon Res* 1990; 10: 141-151.

Ruzicka FJ, Jach ME, Borden EC. Binding of recombinant-produced interferon $ß_{ser}$ to human lymphoblastoid cells. *J Biol Chem* 1987; 262(33): 16142-16149.

Schiller JH, Horisberger MA, Bittner G, *et al.* Effects of combinations of interferon-$ß_{ser}$ and interferon-γ on interferon-inducible proteins and on the cell cycle. *J Biol Resp Mod* 1990; 9: 368-377.

The IFNB Multiple Sclerosis Study Group. Interferon beta-1b is effective in relapsing-remitting multiple sclerosis. *Neurology* 1993; 43: 655-661.

The IFNB Multiple Sclerosis Study Group and the University of British Columbia MS/MRI Analysis Group. Interferon beta-1b in the treatment of multiple sclerosis: final outcome of the randomized controlled trial. *Neurology* 1995; 45: 1277-1285.

Uze G, Lutfalla G, Gresser I. Genetic transfer of a functional human interferon a receptor into mouse cells: cloning and expression of its cDNA. *Cell* 1990; 60: 225-234.

Witt PL, Spear GT, Helgeson DO, *et al.* Basal and interferon-induced 2',5'-oligoadenylate synthetase in human monocytes, lymphocytes, and peritoneal macrophages. *J Interferon Res* 1990; 10: 393-402.

Witt PL, Storer BE, Bryan GT, *et al.* Pharmacodynamics of biological response in vivo after single and multiple doses of interferon-ß. *J Immunotherapy* 1993; 13: 191-200.

Version révisée – Mars 2003

Appendix 6

Magnevist®

Part 1 - Prescribing Information (USA)
Part 2 - Monograph (Canada / English-language document)
Part 3 - Monographie (Canada / French-language document)

Magnevist®

Part 1 - Prescribing Information (USA)

Revision Date: May 2000

BERLEX IMAGING®

MAGNEVIST®

(brand of gadopentetate dimeglumine)

Injection

6062605

Rx only

DESCRIPTION

MAGNEVIST® (brand of gadopentetate dimeglumine) Injection is the N-methylglucamine salt of the gadolinium complex of diethylenetriamine pentaacetic acid, and is an injectable contrast medium for magnetic resonance imaging (MRI). MAGNEVIST® Injection is provided as a sterile, clear, colorless to slightly yellow aqueous solution for intravenous injection.

MAGNEVIST® Injection is a 0.5-mol/L solution of 1-deoxy-1-(methylamino)-D-glucitol dihydrogen [*N,N*-bis[2-[bis(carboxymethyl)amino]ethyl]-glycinato-(5⁻)-]gadolinate(2⁻)(2:1) with a molecular weight of 938, an empirical formula of $C_{28}H_{54}GdN_5O_{20}$, and has the following structural formula:

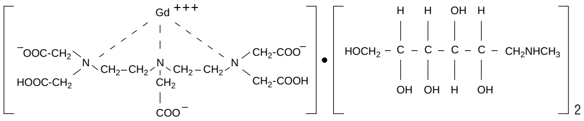

Each mL of MAGNEVIST® Injection contains 469.01 mg gadopentetate dimeglumine, 0.99 mg meglumine, 0.40 mg diethylenetriamine pentaacetic acid and water for injection. MAGNEVIST® Injection contains no antimicrobial preservative.

MAGNEVIST® Injection has a pH of 6.5 to 8.0. Pertinent physicochemical data are noted below:

PARAMETER		
Osmolality (mOsmol/kg water)		
	at 37°C	1,960
Viscosity (CP)		
	at 20°C	4.9
	at 37°C	2.9
Density (g/mL)		
	at 25°C	1.195
Specific gravity		
	at 25°C	1.208
Octanol: H_2O		
	at 25°C	
	and pH7	log P_W = - 5.4

MAGNEVIST® Injection has an osmolality 6.9 times that of plasma which has an osmolality of 285 mOsmol/kg water. MAGNEVIST® Injection is hypertonic under conditions of use.

CLINICAL PHARMACOLOGY

Pharmacokinetics

The pharmacokinetics of intravenously administered gadopentetate dimeglumine in normal subjects conforms to a two compartment open-model with mean distribution and elimination half-lives (reported as mean ± SD) of about 0.2 ± 0.13 hours and 1.6 ± 0.13 hours, respectively.

Upon injection, the meglumine salt is completely dissociated from the gadopentetate dimeglumine complex. Gadopentetate is exclusively eliminated in the urine with 83 ± 14% (mean ± SD) of the dose excreted within 6 hours and 91 ± 13% (mean ± SD) by 24 hours, post-injection. There was no detectable biotransformation or decomposition of gadopentetate dimeglumine.

The renal and plasma clearance rates (1.76 ± 0.39 mL/min/kg and 1.94 ± 0.28 mL/min/kg, respectively) of gadopentetate are essentially identical, indicating no alteration in elimination kinetics on passage through the kidneys and that the drug is essentially cleared through the kidney. The volume of distribution (266 ± 43 mL/kg) is equal to that of extracellular water and clearance is similar to that of substances which are subject to glomerular filtration.

In vitro laboratory results indicate that gadopentetate does not bind to human plasma protein. *In vivo* protein binding studies have not been done.

Pharmacodynamics

Gadopentetate dimeglumine is a paramagnetic agent and, as such, it develops a magnetic moment when placed in a magnetic field. The relatively large magnetic moment produced by the paramagnetic agent results in a relatively large local magnetic field, which can enhance the relaxation rates of water protons in the vicinity of the paramagnetic agent.

In magnetic resonance imaging (MRI), visualization of normal and pathological brain tissue depends in part on variations in the radiofrequency signal intensity that occur with 1) changes in proton density; 2) alteration of the spin-lattice or longitudinal relaxation time (T1); and 3) variation of the spin-spin or transverse relaxation time (T2). When placed in a magnetic field, gadopentetate dimeglumine decreases the T1 and T2 relaxation time in tissues where it accumulates. At usual doses the effect is primarily on the T1 relaxation time.

Gadopentetate dimeglumine does not cross the intact blood-brain barrier and, therefore, does not accumulate in normal brain or in lesions that do not have an abnormal blood-brain barrier, e.g., cysts, mature post-operative scars, etc. However, disruption of the blood-brain barrier or abnormal vascularity allows accumulation of gadopentetate dimeglumine in lesions such as neoplasms, abscesses, and subacute infarcts. The pharmacokinetic parameters of MAGNEVIST® in various lesions are not known.

CLINICAL TRIALS

MAGNEVIST® Injection was administered to 1272 patients in open label controlled clinical studies. The mean age of these patients was 46.4 years (range 2 to 93 years). Of these patients, 55% (700) were male and 45% (572) were female. Of the 1271 patients who received MAGNEVIST® Injection and for whom race was reported, 82.1% (1043) were Caucasian, 9.7% (123) were Black, 5.3% (67) were Hispanic, 2.1% (27) were Oriental/Asian, and 0.9% (11) were other. Of the 1272 patients, 550 patients were evaluated in blinded reader studies. These evaluated the use of contrast enhancement in magnetic resonance imaging of lesions in the head and neck, brain, spine and associated tissues, and body (excluding the heart). Of the 550 patients, all patients had a reason for an MRI and efficacy assessments were based on pre- and post-MAGNEVIST® injection film quality, film contrast, lesion configuration (border, size, and location), and the number of lesions. The protocols did not include systematic verification of specific diseases or histopathologic confirmation of findings.

Of the above 550 patients, 97 patients received 0.1 mmol/kg MAGNEVIST® Injection I.V. in two clinical trials of MAGNEVIST® MRI contrast enhancement for body imaging. Of these 97, 68 had MRIs of the internal organs/structures of the abdomen or thorax (excluding the heart); 8 had breast images and 22 had images of appendages. The results of MRIs before and after MAGNEVIST® use were compared blindly. Overall additional lesions were identified in 22/97 (23%) of the patients after MAGNEVIST® Injection. The mean number of lesions identified before (1.49/patient) and after MAGNEVIST® (1.75/patient) were similar. Seven (8%) of the patients had lesions seen before MAGNEVIST® that were not seen after MAGNEVIST.® Overall, after MAGNEVIST® Injection, 41% of the images had a higher contrast score than before injection; and 18% of the images had a higher contrast score before MAGNEVIST® Injection than after MAGNEVIST® Injection. MAGNEVIST® MRI of the 8 patients with breast images were not systematically compared to the results to mammography, breast biopsy or other modalities. In the 22 patients with appendage images (e.g., muscle, bone and intraarticular structures), MAGNEVIST® MRI was not systematically evaluated to determine the effects of contrast biodistribution in these different areas.

Of the above 550 patients, 66 patients received MAGNEVIST® 0.1 mmol/kg I.V. in clinical trials of MAGNEVIST® MRI contrast enhancement of lesions in the head and neck. A total of 66 MRI images were evaluated blindly by comparing each pair of MRI images, before and after MAGNEVIST® Injection. In these paired images, 56/66 (85%) had greater enhancement after MAGNEVIST® and 40/66 (61%) had better lesion configuration or border delineation after MAGNEVIST.® Overall, there was better contrast after MAGNEVIST® in 55% of the images, comparable enhancement in 44 (36%) before and after MAGNEVIST,® and better enhancement in 9% without MAGNEVIST.®

In the studies of the brain and spinal cord, MAGNEVIST® 0.1 mmol/kg I.V. provided contrast enhancement in lesions with an abnormal blood brain barrier.

In two studies, a total of 108 patients were evaluated to compare the dose response effects of 0.1 mmol/kg and 0.3 mmol/kg of MAGNEVIST® in CNS MRI. Both dosing regimens had similar imaging and general safety profiles; however, the 0.3 mmoL/kg dose did not provide additional benefit to the final diagnosis (defined as number of lesions, location and characterization).

INDICATIONS AND USAGE

Central Nervous System:

MAGNEVIST® Injection is indicated for use with magnetic resonance imaging (MRI) in adults, and pediatric patients (2 years of age and older) to visualize lesions with abnormal vascularity in the brain (intracranial lesions), spine and associated tissues. MAGNEVIST® Injection has been shown to facilitate visualization of intracranial lesions including but not limited to tumors.

Extracranial/Extraspinal Tissues:

MAGNEVIST® is indicated for use with MRI in adults and pediatric patients (2 years of age and older) to facilitate the visualization of lesions with abnormal vascularity in the head and neck.

Body:

MAGNEVIST® Injection is indicated for use in MRI in adults and pediatric patients (2 years of age and older) to facilitate the visualization of lesions with abnormal vascularity in the body (excluding the heart).

CONTRAINDICATIONS

None.

WARNINGS

As with various other intravenous administrations, caution must be used when administering MAGNEVIST® Injection in patients with predisposition to the development of thrombotic syndromes. (See **PRECAUTIONS**).

Deoxygenated sickle erythrocytes have been shown by *in vitro* studies to align perpendicular to a magnetic field which may result in vaso-occlusive complications *in vivo*. The enhancement of magnetic moment by gadopentetate dimeglumine may possibly potentiate sickle erythrocyte alignment. MAGNEVIST® Injection in patients with sickle cell anemia and other hemoglobinopathies has not been studied.

Patients with other hemolytic anemias have not been adequately evaluated following administration of MAGNEVIST® Injection to exclude the possibility of increased hemolysis.

Hypotension may occur in some patients after injection of MAGNEVIST® Injection. In clinical trials two cases were reported and in addition, there was one case of vasovagal reaction and two cases of pallor with dizziness, sweating and nausea in one and substernal pain and flushing in the other. These were reported within 25 to 85 minutes after injection except for the vasovagal reaction which was described as mild by the patient and occurred after 6-1/2 hours. In a study in normal volunteers one subject experienced syncope after arising from a sitting position two hours after administration of the drug. Although the relationship of gadopentetate dimeglumine to these events is uncertain, patients should be observed for several hours after drug administration.

Patients with a history of allergy, drug reactions, or other hypersensitivity-like disorders, should be closely observed during the procedure and for several hours after drug administration. (See PRECAUTIONS – General.)

PRECAUTIONS – General

As with various other injectable products, cases of phlebitis and thrombophlebitis have been reported also in association with MAGNEVIST® Injection. In most cases, symptoms presented during or shortly after injection, and generally within 24 hours of injection and responded to supportive treatment. However, in very rare cases of patients who may have underlying potential to develop thrombotic syndromes, thrombosis with fasciitis and surgical intervention (e.g., compartment release or amputation) of the dosed limb have been reported. The relationship of these events to pre-existing disease, concomitant medications, pre-existing vascular fragility, MAGNEVIST® Injection, or the injection procedure was not established. Patency and integrity of the intravenous line should be determined before administration. As with other intravenous injections, appropriate surveillance of the dosing limb for the development of local injection site reactions following administration of MAGNEVIST® Injection is recommended.

AS WITH ANY PARAMAGNETIC CONTRAST AGENT, MRI WITH MAGNEVIST® CONTRAST ENHANCEMENT MAY IMPAIR THE VISUALIZATION OF EXISTING LESIONS. SOME OF THESE LESIONS MAY BE SEEN ON UNENHANCED, NON-CONTRAST MRI. THEREFORE, CAUTION SHOULD BE EXERCISED WHEN CONTRAST ENHANCED SCAN INTERPRETATION IS MADE IN THE ABSENCE OF A COMPANION UNENHANCED MRI.

Diagnostic procedures that involve the use of contrast agents should be carried out under direction of a physician with the prerequisite training and a thorough knowledge of the procedure to be performed.

In a patient with a history of grand mal seizure, MAGNEVIST® Injection was reported to induce such a seizure.

Since gadopentetate dimeglumine is cleared from the body by glomerular filtration, caution should be exercised in patients with impaired renal function. MAGNEVIST® is not significantly eliminated by the hepatobiliary enteric pathway, but is dialyzable (See **Pharmacodynamics** Section). Caution should be exercised in patients with either renal or hepatic impairment.

The possibility of a reaction, including serious, life-threatening, or fatal anaphylactoid or cardiovascular reactions or other idiosyncratic reactions (see **ADVERSE REACTIONS**), should always be considered, especially in those patients with a known clinical hypersensitivity or a history of asthma or other allergic respiratory disorders.

Animal studies suggest that gadopentetate dimeglumine may alter red cell membrane morphology resulting in a slight degree of extravascular (splenic) hemolysis. In clinical trials 15-30% of the patients experienced an asymptomatic transient rise in serum iron. Serum bilirubin levels were slightly elevated in approximately 3.4% of patients. Levels generally returned to baseline within 24 to 48 hours. Hematocrit and red blood cell count were unaffected and liver enzymes were not elevated in these patients. While the effects of gadopentetate dimeglumine on serum iron and bilirubin have not been associated with clinical manifestations, the effect of the drug in patients with hepatic disease is not known and caution is therefore advised.

When MAGNEVIST® Injection is to be injected using nondisposable equipment, scrupulous care should be taken to prevent residual contamination with traces of cleansing agents. After MAGNEVIST® Injection is drawn into the syringe the solution should be used immediately.

Repeat Procedures: Data for repeated procedures are not available. If in the clinical judgment of the physician sequential or repeat procedures are required, a suitable interval of time between administrations should be observed to allow for normal clearance of the drug from the body.

Repeat Injections: (See **DOSAGE AND ADMINISTRATION**)

Information for Patients:

Patients scheduled to receive MAGNEVIST® Injection should be instructed to inform their physician if the patient:

1. Is pregnant or breast feeding.
2. Has any blood disorders; i.e., anemia, hemoglobinopathies, or diseases that affect red blood cells.
3. Has a history of renal or hepatic disease, seizure, asthma or allergic respiratory disorders.

LABORATORY TEST FINDINGS

Transitory changes in serum iron, bilirubin and transaminase levels have been reported in patients with normal and abnormal liver function (See **PRECAUTIONS** – General).

CARCINOGENESIS, MUTAGENESIS AND IMPAIRMENT OF FERTILITY

Long term animal studies have not been performed to evaluate the carcinogenic potential of gadopentetate dimeglumine.

A comprehensive battery of *in vitro* and *in vivo* studies in bacterial and mammalian systems suggest that gadopentetate dimeglumine is not mutagenic or clastogenic and does not induce DNA repair in rat hepatocytes or cause cellular transformation of mouse embryo fibroblasts. A dominant lethal effect on early spermatids was demonstrated *in vivo* in the mouse in one study after intravenous administration of 6 mmol/kg but was not verified in a follow up study.

When administered intra-peritoneally to male and female rats daily prior to mating, during mating and during embryonic development for up to 74 days (males) or 35 days (females), gadopentetate caused a decrease in number of corpora lutea at the 0.1 mmol/kg dose level. After daily dosing with 2.5 mmol/kg suppression of food consumption and body weight gain (males and females) and a decrease in the weights of testes and epididymis were also observed.

In a separate experiment in rats, daily injections of gadopentetate dimeglumine over 16 days caused spermatogenic cell atrophy at a dose level of 5 mmol/kg but not at a dose level of 2.5 mmol/kg. This atrophy was not reversed within a 16-day observation period following the discontinuation of the drug.

PREGNANCY CATEGORY C.

Gadopentetate dimeglumine retarded fetal development slightly when given intravenously for 10 consecutive days to pregnant rats at daily doses of 0.25, 0.75, and 1.25 mmol/kg (2.5, 7.5 and 12.5 times the human dose based on body weight) and when given intravenously for 13 consecutive days to pregnant rabbits at daily doses of 0.75 and 1.25 mmol/kg (7.5 and 12.5 times the human dose respectively, based on body weight) but not at daily doses of 0.25 mmol/kg. Congenital anomalies were noted in rats or rabbits.

Adequate and well controlled studies were not conducted in pregnant women. MAGNEVIST® Injection should be used during pregnancy only if the potential benefit justifies the potential risk to the fetus.

NURSING WOMEN

C^{14} labelled gadopentetate dimeglumine was administered intravenously to lactating rats at a dose of 0.5 mmol/kg. Less than 0.2% of the total dose was transferred to the neonate via the milk during the 24-hour evaluation period. It is not known to what extent MAGNEVIST® Injection is excreted in human milk. Because many drugs are excreted in human milk, caution should be exercised when MAGNEVIST® Injection is administered to a nursing woman.

PEDIATRIC USE

The use of MAGNEVIST® in imaging the Central Nervous System, Extracranial/Extraspinal tissues, and Body have been established in the pediatric population from the ages of 2 to 16 years on the basis of adequate and well controlled clinical trials in adults and safety studies in this pediatric population. (See Clinical Trials for details.)

Safety and efficacy in the pediatric population under the age of 2 years have not been established. MAGNEVIST® is eliminated primarily by the kidney. The pharmacokinetics of MAGNEVIST® in neonates and infants with immature renal function have not been studied.
(See **INDICATIONS** and the **DOSAGE AND ADMINISTRATION**)

ADVERSE REACTIONS

The mean age of the 1272 patients who received MAGNEVIST® Injection was 46.4 years (range 2 to 93 years). Of these patients, 55% (700) were male and 45% (572) were female. Of the 1271 patients who received MAGNEVIST® Injection and for whom race was reported, 82.1% (1043) were Caucasian, 9.7% (123) were Black, 5.3% (67) were Hispanic, 2.1% (27) were Oriental/Asian, and 0.9% (11) were other. The most common noted adverse event is headache with an incidence of 4.8%. The majority of headaches are transient and of mild to moderate severity. Nausea is the second most common adverse experience at 2.7%. Injection site coldness/localized coldness is the third most common adverse experience at 2.3%. Dizziness occurred in 1% of the patients.

The following additional adverse events occurred in fewer than 1% of the patients:

Body as a Whole: Injection site symptoms, namely, pain, localized warmth, and burning sensation; substernal chest pain, back pain, fever, weakness, generalized coldness, generalized warmth, localized edema, tiredness, chest tightness, trembling, shivering, tension in extremities, regional lymphangitis, pelvic pain, and anaphylactoid reactions (characterized by cardiovascular, respiratory and cutaneous symptoms) rarely resulting in death.

Cardiovascular: Hypotension, hypertension, arrhythmia, tachycardia, migraine, syncope, vasodilation, pallor, non-specific ECG changes, angina pectoris, death related to myocardial infarction or other undetermined causes, phlebitis, thrombophlebitis, deep vein thrombophlebitis, compartment syndrome requiring surgical intervention.

Digestive: Gastrointestinal distress, stomach pain, teeth pain, increased salivation, abdominal pain, vomiting, constipation, diarrhea.

Nervous System: Agitation, anxiety, thirst, anorexia, nystagmus, drowsiness, diplopia, stupor, convulsions (including grand mal), paresthesia.

Respiratory System: Throat irritation, rhinorrhea, sneezing, dyspnea, wheezing, laryngismus, cough, respiratory complaints.

Skin: Rash, sweating, pruritus, urticaria (hives), facial edema, erythema multiforme, epidermal necrolysis, pustules.

Special Senses: Tinnitus, conjunctivitis, visual field defect, taste abnormality, dry mouth, lacrimation disorder (tearing), eye irritation, eye pain, ear pain.

OVERDOSAGE

Systemic consequences associated with overdosage of MAGNEVIST® Injection have not been reported.

DOSAGE AND ADMINISTRATION

The recommended dosage of MAGNEVIST® Injection is 0.2 mL/kg (0.1 mmol/kg) administered intravenously, at a rate not to exceed 10 mL per 15 seconds. Dosing for patients in excess of 286 lbs have not been studied systematically.

DOSE AND DURATION OF MAGNEVIST® INJECTION BY BODY WEIGHT

BODY WEIGHT		Total Volume, mL*
lb	kg	
22	10	2
44	20	4
66	30	6
88	40	8
110	50	10
132	60	12
154	70	14
176	80	16
198	90	18
220	100	20
242	110	22
264	120	24
286	130	26

*Rate of Injection: 10 mL/15 sec

Drug Handling: To ensure complete injection of the contrast medium, the injection should be followed by a 5-mL normal saline flush. The imaging procedure should be completed within 1 hour of injection of MAGNEVIST® Injection.

As with other gadolinium contrast agents, MAGNEVIST® Injection has not been established for use in magnetic resonance angiography.

Parenteral products should be inspected visually for particulate matter and discoloration prior to administration. Do not use the solution if it is discolored or particulate matter is present.

Any unused portion must be discarded in accordance with regulations dealing with the disposal of such materials.

HOW SUPPLIED

MAGNEVIST® Injection is a clear, colorless to slightly yellow solution containing 469.01 mg/mL of gadopentetate dimeglumine in rubber stoppered vials. MAGNEVIST® Injection is supplied in the following sizes:

5 mL single-dose vials, rubber stoppered, in individual cartons, Boxes of 20	NDC 50419-188-05
10 mL single-dose vials, rubber stoppered, in individual cartons, Boxes of 20	NDC 50419-188-01
10 mL pre-filled disposable syringe, Boxes of 5	NDC 50419-188-30
15 mL single-dose vials, rubber stoppered, in individual cartons, Boxes of 20	NDC 50419-188-15
15 mL pre-filled disposable syringe, Boxes of 5	NDC 50419-188-31
20 mL single-dose vials, rubber stoppered, in individual cartons, Boxes of 20	NDC 50419-188-02
20 mL pre-filled disposable syringe, Boxes of 5	NDC 50419-188-32

STORAGE

MAGNEVIST® Injection should be stored at controlled room temperature, between 15°-30°C (59°-86°F) and protected from light. DO NOT FREEZE. Should freezing occur in the vial MAGNEVIST® Injection should be brought to room temperature before use. If allowed to stand at room temperature for a minimum of 90 minutes, MAGNEVIST® Injection should return to a clear, colorless to slightly yellow solution. Before use, examine the product to assure that all solids are redissolved and that the container and closure have not been damaged. Should solids persist, discard vial.

This product is covered by U.S. Patent No. 4,957,939. The use of this product is covered by U.S. Patent Nos. 4,647,447 and 4,963,344.

©2000, Berlex Laboratories
All rights reserved.

Manufactured for:
BERLEX® Laboratories
Wayne, New Jersey 07470
Manufactured in Germany

6062605 Revised May 2000 USA/Berlex
2016616

Magnevist®

Part 2 - Monograph (Canada / English-language document)

Revision Date: 21 March 2001

PRODUCT MONOGRAPH

MAGNEVIST®

Gadopentetate Dimeglumine Injection

469 mg/mL (0.5 mmol/mL)

For Intravenous Use

Therapeutic Classification

Contrast Enhancement Agent
for Magnetic Resonance Imaging (MRI)

BERLEX CANADA INC.
2260 32nd Avenue
Lachine, Québec
H8T 3H4

Date of Preparation:
August 11, 1993

Date of Revision:
March 21, 2001

® Registered Trademark for which Berlex Canada Inc., Lachine, Quebec, is a Registered User.

PRODUCT MONOGRAPH

MAGNEVIST®

Gadopentetate Dimeglumine Injection

469 mg/mL (0.5 mmol/mL)

For Intravenous Use

Therapeutic Classification

Contrast Enhancement Agent
for Magnetic Resonance Imaging (MRI)

ACTION AND CLINICAL PHARMACOLOGY

MAGNEVIST (gadopentetate dimeglumine) was developed as a contrast agent for diagnostic use in magnetic resonance imaging (MRI). Gadolinium is a rare earth element. Its ion (Gd^{+++}) has seven unpaired electrons and, therefore, shows paramagnetic properties. Gd^{+++} has a strong effect on the hydrogen-proton spin-lattice relaxation time (T_1), which causes the observed contrast enhancement in MRI scans. By chelation of Gd^{+++} with diethylenetriamine pentaacetic acid (DTPA), a strongly paramagnetic, well-tolerated, stable complex (gadopentetate dimeglumine salt) is obtained.

The free gadolinium ion is unsuitable for clinical use due to high toxicity, however, the metal chelate is metabolically inert. The organic component of the chelate is not measurably metabolized and the metal does not dissociate. After intravenous injection of

gadopentetate dimeglumine, the meglumine ion completely dissociates from the gadopentetate. The hydrophilic chelate is distributed only in the extracellular water and does not cross the intact blood-brain barrier. Gadopentetate is excreted unchanged in the urine. It is rapidly eliminated by the kidneys with a clearance identical to that of inulin (no tubular reabsorption).

The pharmacokinetic profile of intravenously administered gadopentetate dimeglumine in normal subjects conforms to a two compartment open model with a mean distribution half-life of about 0.2 hours and a mean elimination half-life of about 1.6 hours. Approximately 80% of the dose was excreted in the urine within 6 hours and 93% within 24 hours post injection of a 0.1 mmol/kg dose. Excretion in the faeces amounted to <0.1% over 5 days. There was no detectable biotransformation, dissociation or decomposition of gadopentetate.

MAGNEVIST has no pharmacodynamic effect when administered as indicated with the exception of slightly increased plasma osmolality.

INDICATIONS AND CLINICAL USE

MAGNEVIST (gadopentetate dimeglumine), by intravenous injection, is indicated for contrast enhancement during cranial and spinal MRI investigations in adults and children, to detect lesions associated with abnormal vascularity, or those thought to alter the blood-brain barrier.

MAGNEVIST is also indicated for use with MRI in adults to provide contrast enhancement and facilitate visualization of lesions with abnormal vascularity within the head (extracranial) and neck.

CONTRAINDICATIONS

MAGNEVIST (gadopentetate dimeglumine) should not be administered to patients who are known or suspected of being hypersensitive to it.

WARNINGS

The decision to use MAGNEVIST (gadopentetate dimeglumine) must be made after careful evaluation of the risk-benefit in patients with a history of allergic disposition or bronchial asthma, since experience shows that these patients suffer more frequently than others from hypersensitivity reactions.

In very rare cases anaphylactoid reactions, including anaphylactic shock, may occur after intravenous injection of MAGNEVIST. It is important for prompt action in the event of such incidents to be familiar with the practice of emergency measures. To permit immediate counter-measures to be taken in emergencies, appropriate drugs and instruments (e.g. endotracheal tube and ventilator) should be readily available.

Deoxygenated sickle cell erythrocytes have been shown in *in vitro* studies to align perpendicular to a magnetic field which may result in vaso-occlusive complications *in vivo*. The enhancement of magnetic moment by gadopentetate dimeglumine may possibly potentiate sickle erythrocyte alignment. MAGNEVIST in patients with sickle cell anemia and other hemoglobinopathies has not been studied.

No studies have been conducted in children with severe renal or hepatic dysfunction, clinically unstable or uncontrolled hypertension, or in premature infants.

MRI procedures which involve the use of MAGNEVIST by injection should be carried out by physicians who have the prerequisite training and a thorough knowledge of the particular procedure to be performed.

PRECAUTIONS

General

MAGNEVIST (gadopentetate dimeglumine) is to be administered strictly by intravenous injection. MAGNEVIST will cause tissue irritation and pain if administered extravascularly or if it leaks interstitially.

A sweet taste may be experienced briefly by patients receiving a bolus injection of MAGNEVIST intravenously.

Hemolytic States

Gadopentetate dimeglumine alters red blood cell morphology resulting in transient, slight, extravascular (splenic) hemolysis with increased serum iron and total bilirubin levels. Although this effect was of no clinical significance during clinical trials, caution is advised in patients with hepatic disease and/or hemolytic states.

Convulsive States

While there is no evidence suggesting that MAGNEVIST directly precipitates convulsion, the possibility that it may decrease the convulsive threshold in susceptible patients cannot be ruled out. Precautionary measures should be taken with patients predisposed to seizure, e.g. close monitoring and availability of injectable anticonvulsants (see **DOSAGE AND ADMINISTRATION**).

Pregnancy

There are no studies on the use of MAGNEVIST in pregnant women. MAGNEVIST should not be used during human pregnancy unless the potential benefit justifies the potential risk to the fetus.

Nursing Mothers

Transfer of MAGNEVIST into the milk of lactating mothers can occur. Thus breast feeding should be interrupted for 24 hours post administration of MAGNEVIST and the milk discarded during this period.

Use in the Elderly

No special precautions are required for elderly patients.

ADVERSE REACTIONS

General

Most adverse reactions to MAGNEVIST (gadopentetate dimeglumine) develop soon after injection, however the possibility of delayed reactions cannot be ruled out. The most frequently reported adverse reactions following administration of MAGNEVIST were:

Headache	8.7%*
in some cases severe	1.3%
Injection Site Discomfort	6.7%
Nausea	3.2%
Localized Pain in Other Parts of the Body (back, ear, eye, teeth)	2.8%
Hypersensitivity-Type Skin and Mucosal Reactions	2.1%
Dizziness	1.5%
Vomiting	1.2%
Paresthesia	1.2%

* 42.3% of all cases of headache were considered unrelated to MAGNEVIST

administration.

Adverse reactions occurred in 11 of 319 (3.4%) pediatric patients receiving MAGNEVIST in clinical trials (headache, vasodilatation, dizziness, diarrhea, ear pain, tachycardia, fever, edema, seizure, vomiting, nausea and urticaria). This adverse reaction profile is consistent with the adverse reaction profile observed in adults.

MAGNEVIST will cause tissue irritation and pain if administered extravascularly.

Transient increases or decreases in blood pressure may occur after the administration of MAGNEVIST. These changes are generally of little consequence although 3 clinically significant cases of hypotension have occurred 2-6 hours after MAGNEVIST injection. A relationship to the contrast medium could not be determined, however caution should be exercised by the patient when driving or operating machinery.

Serious or severe adverse effects associated with MAGNEVIST have been rare in clinical experience. Postmarketing anaphylactic reactions have been reported, but are very rare. Convulsions were reported in 4 patients with a history of seizures.

Laboratory Changes

Reversible mild elevations over baseline in serum iron and total bilirubin occur in most patients after receiving MAGNEVIST. These changes do not appear to be clinically relevant. Other disturbances in laboratory values (transient increases in liver function tests) have not been associated with the use of MAGNEVIST.

Adverse Drug Reaction Profile

The following adverse reactions, listed according to body system, have been reported after administration of MAGNEVIST:

Cardiovascular: hypotension, vasodilatation, pallor, phlebitis, non-specific ECG changes, substernal pain, angina

Central nervous system: headache, dizziness, agitation, paresthesia, tinnitus, visual field defect, convulsions, hyperesthesia

Gastrointestinal: nausea, vomiting, gastrointestinal distress, stomach pain, thirst, increased salivation, taste abnormality

Respiratory system: dry mouth, throat irritation, rhinorrhea, wheezing, sneezing, laryngismus, cough, dyspnea/apnea

Cutaneous/mucous membranes: rash, sweating, urticaria, pruritus

Miscellaneous: injection site discomfort (coldness, burning, warmth, pain), teeth pain, generalized weakness, fever, localised edema, tiredness, anaphylactoid reactions (characterised by cardiovascular, respiratory and cutaneous symptoms), conjunctivitis

Laboratory tests: transient elevation of serum iron and bilirubin levels

The following other adverse events were reported. A causal relationship has neither been established nor refuted:

Cardiovascular: hypertension, tachycardia, syncope, death related to myocardial infarction or other undetermined causes

Central nervous system: diplopia, migraine, anxiety, drowsiness, nystagmus, stupor

Gastrointestinal: constipation, diarrhea, anorexia

Cutaneous/mucous membranes: facial edema, erythema, epidermal necrolysis

Miscellaneous: localised pain (back, ear, eye)

SYMPTOMS AND TREATMENT OF OVERDOSAGE

In the event of inadvertent overdosage or in the case of severely impaired renal function, MAGNEVIST (gadopentetate dimeglumine) can be removed from the body by extracorporeal hemodialysis.

DOSAGE AND ADMINISTRATION

Special preparation of the patient for examination with MAGNEVIST (gadopentetate dimeglumine) is not required; however, precautionary measures should be taken with patients predisposed to seizure, e.g. close monitoring and availability of injectable anticonvulsants (see **PRECAUTIONS**). The usual safety rules for MRI (e.g. exclusion of ferromagnetic vascular clips) must be observed.

Young children, infants and neonates may require sedation prior to undergoing an MRI examination, in order to eliminate movement artifacts.

The following dosage guidelines apply to adults and children (including neonates and infants):

**Recommended Dose:**	0.2 mL/kg (0.1 mmol/kg)
**Route of Administration:**	intravenous (into a large vein, if possible)
**Rate of Administration:**	10 mL/min or as a bolus injection at 10 mL/15 sec
**Maximum Total Dose:**	20 mL

To ensure complete injection of the contrast medium, the injection should be followed by a 5 mL normal saline flush.

If strong clinical suspicion of an intracranial or intraspinal lesion persists, despite a normal MRI scan, the diagnostic yield of the examination may be increased by giving another injection of MAGNEVIST equivalent to the original total dose within 30 minutes and performing MRI again.

MAGNEVIST should not be drawn into the syringe until immediately before use. Any unused portion must be discarded upon completion of the procedure.

T_1 -weighted scanning sequences are particularly suitable for contrast-enhanced examinations.

MAGNEVIST has been shown to be effective in a wide range of field strengths (0.14 to 1.5 Tesla).

Important Note

The imaging procedure should be completed within **one hour**. Optimal contrast is generally observed in cranial investigations within 27 minutes following injection of MAGNEVIST and in spinal investigations during the early post administration phase (10 - 30 minutes).

In neonates and infants, optimal CNS contrast has been observed to persist for several hours after MAGNEVIST administration.

PHARMACEUTICAL INFORMATION

Drug Substance

Trade Name:	MAGNEVIST
Proper Name:	Gadopentetate dimeglumine (USAN)
Chemical Name:	Gadolinate(2-),[N,N-bis[2-[bis(carboxymethyl) amino]ethyl]glycinato(5-)]-, dihydrogen, compound with 1-deoxy-1-(methylamino) -D-glucitol(1:2)

Structural Formula :

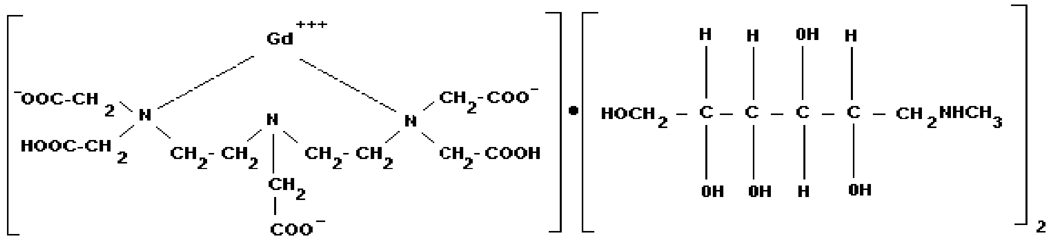

Molecular Formula: $C_{14}H_{20}GdN_3O_{10} \cdot (C_7H_{17}NO_5)_2$

Molecular Weight: 938.02

Solubility: Freely soluble in water

Osmolality: 1960 mOsm/kg H_2O at 37ºC

Composition

MAGNEVIST for intravenous injection is provided as a sterile, clear colorless to slightly yellow aqueous solution. Each mL contains 469.01 mg gadopentetic acid dimeglumine salt (equivalent to 0.5 mmol/mL), 0.99 mg meglumine and 0.40 mg diethylenetriamine pentaacetic acid.

Stability and Storage Recommendations

MAGNEVIST should be stored at 15-30ºC and protected from light.

AVAILABILITY OF DOSAGE FORMS

MAGNEVIST (gadopentetate dimeglumine) is provided as a sterile, clear colorless to slightly yellow aqueous solution. Each mL contains 469.01 mg gadopentetic acid dimeglumine salt (equivalent to 0.5 mmol/mL), 0.99 mg meglumine and 0.40 mg diethylenetriamine pentaacetic acid.

MAGNEVIST is supplied in 20 mL, 15 mL and 10 mL single dose vials packaged in individual cartons

MAGNEVIST should be stored at 15-30ºC and protected from light.

PHARMACOLOGY

Animal Studies

Neuropharmacology

The neuropharmacology of gadopentetate dimeglumine was evaluated in rats, following single pericerebral or intracisternal injection. The ED_{50}, based on postural anomalies, seizures or death, and the LD_{50} determinations indicated that gadopentetate dimeglumine is considerably less toxic than gadolinium chloride or meglumine diatrizoate. In a similar study, the addition of up to 1.0 mg of free DTPA/mL did not affect the neural tolerance of the gadopentetate dimeglumine (Table No. 1).

Table No. 1 A Comparison of the ED_{50} and LD_{50} of Gadopentetate Dimeglumine, Gadolinium Chloride and Meglumine Diatrizoate Following Pericerebral or Intracisternal Administration in Rats.

Compounds	Dose Level (µmol/kg)	ED_{50} (µmol/kg)	Dose Level (µmol/kg)	LD_{50} (µmol/kg)
**Pericerebral Administration**				
Gadopentetate Dimeglumine	25-296.3	96.6<br>97.1	463-1852	1141.4<br>1227.3
Gadopentetate Dimeglumine with 1.0 mg DTPA/mL	25-296.3	80.2	463-1852	1063.4
Gadolinium Chloride	5-25	10.8	6-100	14.9
Meglumine Diatrizoate	32-53	35.0	32-53	42.8
**Intracisternal Administration**				
Gadopentetate Dimeglumine	16.7-197.9	74.0<br>86.2	309-1233<br>a	654.9<br>a
Gadopentetate Dimeglumine with 0.15 mg DTPA/mL	16.7-197.9	80.0	a	a
Gadopentetate Dimeglumine with 1.0 mg DTPA/mL	16.7-197.9	85.0	a	a
Gadolinium Chloride	3.3-16.7	5.6	4-17	8.1
Meglumine Diatrizoate	4-21	11.2	32-126	54.9

a - Not evaluated in the study.

Cardiovascular and Hemodynamic Effects

The cardiovascular and hemodynamic effects of gadopentetate dimeglumine were assessed in healthy anesthetized dogs following intravenous administration of 0.25 or 1.25 mmol/kg of body weight. A slight increase in peripheral resistance was noted at the low-dose level. Those dogs receiving 1.25 mmol/kg initially displayed reduced peripheral resistance, lower blood pressure and heart rate and an increase in the left ventricular end-diastolic pressure, stroke volume and cardiac output. Thereafter, the peripheral resistance increased and there was a significant increase in blood pressure which persisted at the same level for the remainder of the experiment.

The hemodynamic effects of gadopentetate dimeglumine were also assessed in dogs with acute ischemia-induced heart failure using doses of 0.25 mmol/kg and 0.75 mmol/kg intravenously. The 0.25 mmol/kg dose elicited a slight decrease in diastolic blood pressure and peripheral resistance and a slight increase in left ventricular dp/dt, cardiac output and stroke index. All parameters returned to the normal range five to ten minutes after administration. The 0.75 mmol/kg dose also elicited a similar transient response in hemodynamic parameters.

Renal Tolerance

The renal tolerance of gadopentetate dimeglumine was examined in rabbits following an intravenous dose of 2 mmol/kg. A slight effect on urinary protein excretion was seen in comparison to a sorbitol control solution; however gadopentetate dimeglumine exhibited better renal tolerance than other X-ray contrast agents. No effect was seen on serum creatinine or urea-nitrogen levels which served as indicators of renal function. Furthermore, no histological effects could be detected in the kidneys after the one week observation period.

Physicochemical and Biochemical Properties

The pharmacological properties of gadopentetate dimeglumine were determined by a battery of *in vitro* and *in vivo* tests following intravenous administration in dogs, rabbits and baboons. Gadopentetate dimeglumine was shown to be highly hydrophilic and, consequently, had no protein binding ability and did not interfere with enzyme activity. In short, the compound was physiologically inert at concentrations anticipated for human use.

Effect on Coagulation

Gadopentetate dimeglumine was evaluated using thromboelastography and citrated dog blood for its *in vitro* effect on the coagulation process. Concentrations up to 29 mmol/L did not affect the coagulation process of citrated dog blood when compared with a control thromboelastogram obtained with normal saline.

Efficacy

The efficacy of gadopentetate dimeglumine was established in rats, rabbits and baboons following intravenous administration for diagnostic MRI. Intravenous doses of 0.01 - 1.0 mmol/kg of body weight enhanced the contrast between healthy and pathological tissue (infarcts, tumors and inflammations). Since gadopentetate dimeglumine was excreted in the urine, it also enhanced renal contrast in the rat at doses as low as 0.01 mmol/kg of body weight.

Pharmacokinetics

Gadopentetate dimeglumine was administered orally and/or intravenously in the rat (males, pregnant females or lactating females), rabbit (pregnant females), dog (females) and baboon (males) to investigate absorption, distribution, metabolism and excretion.

After oral administration, radiolabelled gadopentetate dimeglumine was not absorbed or very poorly absorbed from the gastrointestinal tract of rats and dogs and was excreted almost completely in the faeces (ca. 100% in the rat and 94% in the dog).

After intravenous injection, the compound was excreted primarily in the urine (90% in the rat and >96% in the dog). In renally-impaired rats, biliary excretion of radiolabelled gadopentetate accounted for 2% of the dose in four hours when both kidneys were occluded.

Intravenous doses of gadopentetate dimeglumine did not result in any significant accumulation in tissues studied in the rat, rabbit, dog or baboon. However, in rats with total renal impairment, 3.5% of the radiolabelled gadopentetate dimeglumine dose was secreted into the stomach and bowel four hours after intravenous administration. These results suggest that this compound can be secreted into the gastrointestinal tract, particularly when severe renal impairment exists.

Following single intravenous administrations of radiolabelled gadopentetate dimeglumine (0.5 mmol/kg) to pregnant rabbits, peak concentrations of radiolabelled gadolinium in the fetuses appeared after 30 minutes. In the dam plasma, liver, heart and uterus concentrations remained stable after 15 and 30 minutes. Fetal tissue concentrations were ca. 4% after 15 minutes and 8% after 30 minutes of that in the dams' plasma (corresponding to 0.11% and 0.26% of the total dose, respectively). By 120 minutes, fetal concentrations decreased to 1/4 of peak value. The fetal elimination half-life was 30-50 minutes, similar to that of maternal plasma and tissue.

Following intravenous administrations of radiolabellled gadopentetate dimeglumine to pregnant rats, the compound was shown to be rapidly distributed, did not pass the blood-brain or placental barriers and cleared within 24-hours post-administration.

In lactating rats that were given intravenous administrations of the radiolabelled gadopentetate dimeglumine less than 0.2% of the administered dose was transferred to the offspring via the maternal milk.

Intravenous doses of radiolabelled gadopentetate dimeglumine administered to dogs exhibited no evidence of any metabolism occurring during passage through the body. High performance liquid chromatography did not detect any unchelated gadolinium ion in the animals.

<u>Human Studies</u>

Pharmacokinetics

The pharmacokinetic profile of MAGNEVIST (gadopentetate dimeglumine) was investigated in male volunteers undergoing Magnetic Resonance Imaging (MRI) of the kidneys and urinary bladder during an open label safety and efficacy study conducted in Europe. A single dose of MAGNEVIST was administered intravenously into a cubital vein of each of 20 healthy male volunteers. Four dose levels, ranging from 0.005 mmol/kg to 0.25 mmol/kg were evaluated in groups of five subjects each.

Pharmacokinetic analysis of the plasma concentration versus time data for the two highest doses (0.1 and 0.25 mmol/kg) showed that the disposition of gadopentetate dimeglumine in the body follows a two compartment model with a mean distribution half-life of 0.2 hour and a mean elimination half-life of 1.6 hours. Dose dependent kinetics were not observed for the 0.1 and 0.25 mmol/kg doses. Gadopentetate is exclusively eliminated in the urine

with an average for all four doses of 83% excreted within 6 hours, and 91% of the dose excreted by 24 hours post-injection. No metabolites of gadopentetate were found in urine, indicating that gadopentetate, which forms the active ingredient of the MRI contrast agent, remains intact.

The urinary and plasma elimination rates (111 $\pm$ 19 mL/min and 122 $\pm$ 14 mL/min, respectively) for gadopentetate are essentially identical. The volume of distribution (266 $\pm$ 43 mL/kg) is equal to the calculated volume of extracellular water, and the clearance is similar to that of substances which are subject to glomerular filtration, e.g. inulin and CrEDTA. In man the plasma half-life (1.6 hours) is similar to that reported for dogs and also similar to the elimination characteristics of commonly used x-ray contrast agents for angio-urography.

Clinical Laboratory Evaluations

Clinical laboratory evaluations revealed elevations in serum iron and, in some cases, serum bilirubin levels, which were considered to be definitely drug-related. In about 15% of female and 30% of male patients, increases in serum iron levels above baseline were noted. The increases appeared within 2 to 4 hours post-injection and declined within 24 hours post-injection. By 48 hours post-injection the levels had returned to baseline. Hemoglobin, hematocrit, red blood cell count and liver function enzymes were unaffected. This effect is considered to be due to a slight degree of hemolysis, probably extravascular and too small to result in a change in hemoglobin, hematocrit, or red blood cell count.

Although MAGNEVIST is not a risk for patients with normal hematological status, it is possible that those patients with hematolytic anemia may be at an increased risk, since gadopentetate dimeglumine appears to exert an effect on red blood cell morphology. About 8% of the patients who show a rise in serum iron levels also show a rise in serum

bilirubin levels, apparently because these patients are somewhat less efficient in conjugating bilirubin resulting from hemolysis.

Clinical Studies in Adults with Cranial and Spinal Lesions

The efficacy of MAGNEVIST as an MRI contrast enhancement agent in the diagnosis and evaluation of brain lesions and lesions of the spine and associated tissues was demonstrated in six pivotal clinical trials and in 3 special studies in which films were read by independent evaluators.

In the six clinical trials, a total of 597 patients (571 MAGNEVIST, 26 placebo) were evaluated for efficacy. 196 of these patients (55 brain, 141 spine) were evaluated for inclusion in the radiologist-reader evaluations of MAGNEVIST.

Assessment of efficacy included global efficacy evaluations, intensity scores and film evaluations (including contrast, morphology and diagnosis).

Contrast enhancement: following the injection of MAGNEVIST, an increase in intensity scores was seen for all tissue types evaluated (healthy tissue, lesion, edema and necrosis). Comparative intensity scores, which showed the relative contrast between tissue types, were calculated for the pre- and post-MAGNEVIST scan. MAGNEVIST greatly increased the difference in intensity scores between lesion, edema and healthy tissue compared to the pre-treatment difference. Similar increases in contrast were seen for lesion-edema and lesion-necrosis comparisons.

In five of the six studies (cranial and spinal) contrast enhancement was assessed as an increase in intensity of a lesion compared to its surrounding environment. 292 (86%) of

339 patients showed enhancement after MAGNEVIST. None of the scans from 26 placebo patients showed enhancement.

In four of the six studies additional lesions were detected in 113 (24%) of 466 patients following the administration of MAGNEVIST.

Diagnostic ability: the diagnostic ability of the investigators was improved or facilitated with MAGNEVIST in 107 (66%) of 162 patients in the cranial studies. In the spinal studies diagnosis was facilitated in 131 (78%) of 169 patients.

Change in diagnosis: in the cranial and spinal studies a change in diagnosis was made by the investigators in 129 (41%) of 317 patients who showed enhancement with MAGNEVIST. Cranial lesions which were enhanced by MAGNEVIST were compatible with presenting symptoms in 95% of cases. The most common diagnostic changes in the cranial studies were: nonspecific neoplasms, meningiomas, metastases and glial cell tumors. In the spinal studies the most common change was increased differentiation of scar tissue from abnormal disc material (recurrent post-operative back pain studies) and a better delineation of spinal lesions (changes in lesion size, location and configuration) in patients with suspected spinal tumors.

Film evaluations: film evaluation revealed better contrast in 2/3 of patients with T_1 - weighted scans and more than 1/3 of patients with T_2 - weighted scans. From a group of 167 patients in the cranial studies for whom neither T_1 - weighted nor T_2 - weighted pre-MAGNEVIST scans were diagnostic, diagnosis became possible after the injection of MAGNEVIST in 122 patients (73%).

In the independent radiologist-reader evaluations of the cranial and spinal scans, a significant improvement in the number of lesions detected was observed after

MAGNEVIST. This would have a significant impact on prognosis or treatment, especially in patients where enhanced visualization results in a change of diagnosis, such as a change from negative to positive findings or from a solitary lesion to metastatic disease. The evaluation also showed that MAGNEVIST significantly increased diagnostic accuracy when compared with MRI alone or with computed tomography (CT).

Diagnostic mode (pulse sequence): T_1 - weighted scans provided better enhancement in 138 (93%) of 148 patients in the cranial studies. T_2 - weighted was the better diagnostic mode for 10 (7%) patients. In the spinal studies (post-operative back pain), the T_1 - weighted mode provided better enhancement in 55 (95%) of 58 patients and the T_2 - weighted mode provided better enhancement for 3 (5%) patients.

Time of the best scan: the time of the best scan in the cranial studies was determined both by global efficacy evaluation and by analysis of contrast score results after film evaluations. Both evaluations demonstrated that early post-injection images are best for diagnosis. Of 148 patients with contrast enhancement, 108 (73%) had the best image within 27 minutes of the injection of MAGNEVIST. Of these, more than half had the best scan within 14 minutes of the injection of contrast agent. In spinal investigations the early post-injection scans (10 - 30 minutes) also tended to provide the best images.

Clinical Studies in Children with Cranial and Spinal Lesions

The efficacy of MAGNEVIST was demonstrated in two pivotal clinical studies, involving 142 children with a preliminary diagnosis of CNS abnormality, based upon diagnostic methods other than MRI. Their ages ranged from newborn to 18 years. MRI was performed on all patients before and after the administration of 0.2 mL/kg (0.1 mmol/kg) MAGNEVIST. Some patients were given an additional 0.1 mmol/kg dose within 30 minutes of the first dose, if this was necessary to make a diagnosis.

Contrast evaluations: after MAGNEVIST injection the contrast-to-noise ratio of the magnetic resonance images increased notably, with a further increase in those patients receiving a second MAGNEVIST injection. The signal intensity ratio of lesion to normal tissue was significantly increased for head and spinal T1 scans after MAGNEVIST injection.

Investigator ratings of lesion contrast compared to normal tissue, and of lesion demarcation compared to surrounding tissue, improved after MAGNEVIST injection. Most ratings progressed from "none" or "poor" to "excellent".

Diagnostic usefulness: MAGNEVIST significantly improved the possibility of making a definitive diagnosis. For patients with demonstrated lesions (n=57) with the T1 or T2 scan, this possibility increased from 44% prior to MAGNEVIST injection, to 74% after MAGNEVIST injection. The diagnostic quality of both T1 and T2 scans significantly improved after MAGNEVIST injection, for patients with both normal and abnormal scans.

Lesion morphology was better characterized after MAGNEVIST administration in 11/70 (16%) patients, allowing a better assessment of cystic, necrotic, tumor, or blood components of the lesion. A gain of diagnostic information was documented for 22/40 (55%) patients, and was statistically significant.

MAGNEVIST was demonstrated to be useful in 40/70 (57%) patients. These include 14 patients who were found to have no abnormality after the final MRI, 14 patients in whom a lesion was observed post-MAGNEVIST only, 6 patients in whom a definitive diagnosis was only made possible post-MAGNEVIST, 3 patients in whom complete tumor resection was confirmed by absence of enhancement, 2 patients in whom the solid, cystic, or

necrotic component of the lesion was further characterized, and 1 patient in whom the lesion size was better defined.

Clinical Studies in Adults with Head and Neck Lesions

The efficacy of MAGNEVIST as an MRI contrast enhancement agent was evaluated in 87 patients with head (extracranial) and neck lesions. Film sets from 78 of these patients were additionally assessed by radiologists ("blinded readers") who had not participated in the clinical trials and were not apprised of patient history. Efficacy analyses consisted of comparisons between post-MAGNEVIST scans and corresponding pre-MAGNEVIST scans with respect to contrast enhancement, facilitation of visualization and contrast scores.

Post-MAGNEVIST contrast enhancement of lesions was demonstrated for 78 of 87 (90%) patients in the clinical trials. When evaluated by blinded readers, contrast enhancement was demonstrated for 56 of the 66 (85%) film sets included in the final data set.

Facilitation of visualization was demonstrated primarily by showing that the post-MAGNEVIST scans provided additional radiologic information concerning parameters such as lesion location, size, configuration, and differentiation from edema or necrosis. Post-MAGNEVIST MR scans provided additional radiologic information for 63 of 87 (72%) patients in the clinical trials. Additionally, there was a significant improvement ($p<0.001$) in lesion visualization of post-MAGNEVIST MR scans versus pre-MAGNEVIST MR scans by the blinded readers. Post-MAGNEVIST scans provided a better visualization of lesion configuration versus pre-MAGNEVIST scans for 40 of the 60 (67%) scans where lesion configuration could be determined. Additional radiologic information was observed in 48 of 66 (73%) post-MAGNEVIST scans viewed by the blinded readers.

Each patient's pre- and post-MAGNEVIST MR images were scored on a 4-point scale, measuring the relative intensity of a lesion in relation to its adjacent tissue (0 = no contrast; 1 = equivocal; 2 = good; 3 = excellent). For 63 of 86 (73%) patients in the clinical trials, post-MAGNEVIST contrast scores were higher than pre-MAGNEVIST scores ($p<0.001$). In the blinded reader evaluation, post-MAGNEVIST contrast scores were higher than pre-MAGNEVIST scores in 36 of 66 (55%) patients ($p<0.001$).

TOXICOLOGY

Acute Toxicity

Acute intravenous studies have been carried out with gadopentetate dimeglumine in mice, rats and dogs. Acute oral toxicity studies have been carried out in mice and rats.

Species Predominant Sex (number of animals / group)	Route of Administration, Dose (mmol/kg)	LD_{50} - Range (mmol/kg)	Clinical Findings
Mice, M (3)	oral, 0.25, 1.0, 5.0	> 5.0	None
Mice, M (3)	i.v., 2.5, 5.0, 6.25, 7.5, 10.0	5.0 - 7.5	Apathy, changes in respiration, disturbed gait
Mice, F (3)	i.v. 6.25, 10.0, 12.5, 15.0	6.25 - 12.5	
Rats, M (3)	oral, 0.2, 0.8, 4.0	> 4.0	None
Rats, M (3)	i.v., 10.0, 11.5, 13.5, 15.0	11.5 - 15.0	Prostration, apathy, accelerated respiration, disturbed gait
Rats, F (3)	i.v., 7.5, 10.0, 12.5, 15.0	10.0 - 15.0	
Dogs, M+F (3)	i.v., 6.0	>6.0	Reddening of mucosa and skin, licking, tremor, hematuria, disturbances of gait, retching, vomiting and bleeding at the injection site.

Subacute toxicity

Species	Route of Administration, Dose (mmol/kg)	Duration of Administration	Relevant Prominent Findings
Rats 10/sex/dose	i.v. 1.0, 2.5, 5.0	5 doses/week for 4 weeks	1.0 mmol\kg - without findings. From 2.5 mmol\kg onwards - Dose related apathy, increase in drinking water, consumption, recumbency, respiratory distress, vacuoles in epithelial cells of convoluted tubules and in liver parenchymal cells, slight decrease in hematological parameters, increased absolute and relative liver and kidney weights.<br><br>Additionally after 5 mmol\kg - Convulsion, decrease in body weight gain, half of the animals died.
Rats 5/males/dose	i.v. 2.5, 5.0	once or 5 doses/week for 4 weeks, with 8 and 16 day recovery period	Time-related and dose-related reversibility of renal and hepatic vacuolization.<br>After 5 mmol/kg - atrophy of the spermatogenic cells, not reversible within 15 days.
Dogs, Beagle 2/sex/dose	i.v. 0.25, 1.0, 2.5	5 doses/week for 4 weeks	0.25 mmol/kg - without findings.<br>From 1.0 mmol/kg onwards - dose related reddening of skin, vacuolization of proximal tubules.<br>2.5 mmol/kg - elevated kidney weights, decrease in body weight, increase in drinking water consumption.
Rats, pregnant 25/females/dose	i.v. 0.25, 0.75, 1.25	10 days, day 6-15 of gestation	0.25 - 0.75 mmol/kg - without findings.<br>1.25 mmol/kg - slight increase in wave-like curved ribs, slight retardation of ossification in the fetuses.
Rabbits, pregnant 21-22/females/dose	i.v. 0.25, 0.75, 1.25	13 days	0.25 mmol/kg - without findings.<br>0.75 - 1.25 mmol/kg - dose-related retardation of fetal development.

Mutagenicity Studies

Gadopentetate dimeglumine was evaluated for its mutagenic potential *in vitro* using both bacterial assays (S. typhimurium, E. coli) and mammalian tests (HGPRT test in V 79 cells, UDS test in hepatocytes, cellular transformation assay in C3H 10T1/2 cells); *in vivo*, the product was assessed using two different systems, namely the micronucleus test and dominant lethal assay.

There was no indication that gadopentetate dimeglumine possesses any mutagenic potential *in vitro* or *in vivo.*

Local Tolerance

Gadopentetate dimeglumine was evaluated for its ability to induce local irritation in rabbits following intravenous, paravenous, intramuscular and subcutaneous administration. Intravenous administration of gadopentetate dimeglumine elicited only very slight evidence of irritation. However, paravenous, intramuscular or subcutaneous injections resulted in moderate local irritation.

REFERENCES

Allen ED, Byrd SE, Darling CF et al: The Clinical and Radiological Evaluation of Primary Brain Neoplasms in Children, Part II: Radiological Evaluation. *J National Med Assoc* 1993; 85:546-553.

Brasch RC, Weinmann HJ, Wesby GE: Contrast-enhanced NMR Imaging: Animal Studies Using Gadolinium DTPA Complex. *AJR* 1984; 142:625-630.

Bydder GM, Brown J, Niendorf P, et al: Enhancement of Cervical Intraspinal Tumors in MR Imaging with Intravenous Gadolinium DTPA. *J Comput Assist Tomogr* 1985; 9(5):847-851.

Carr DH: The Use of Proton Relaxation Enhancers in Magnetic Resonance Imaging. *Magn Reson Imaging* 1985; 3(1):17-25.

Carr DH, Brown J, Bydder GM, et al: Intravenous Chelated Gadolinium as a Contrast Agent in NMR Imaging of Cerebral Tumors. *Lancet* 1984; 3:484-486.

Claussen C, Laniado M, Schorner W, et al: Computer Tomographic and Nuclear Magnetic Resonance Diagnoses of Cerebral Tumors Using Conventional and Paramagnetic Contrast Medium. *Rontgen-B1* 1985; 38:143-149.

Dietrich RB: MR and Gadolinium Define Pediatric Brain Pathology. *Diagnostic Imaging* 1989; February: 96-103.

Eldevik OP, and Brunberg JA: Gadopentetate Dimeglumine-enhanced MR of the Brain: Clinical Utility and Safety in Patients Younger than Two Years of Age. *Amer J Neuroradiol* 1994; 15:1001-1008.

Felix R, Schorner W, Laniado M, et al: Brain Tumors: MR Imaging with Gadolinium DTPA. *Radiology* 1985; 156:681-688.

Goldstein EJ, Burnett KR, Hansell JR, et al: Gadolinium DTPA (an NMR Proton Imaging Contrast Agent): Chemical Structure, Paramagnetic Properties and Pharmacokinetics. *Physiol Chem Phys Med NMR* 1984; 16: 97-104.

Nelson KL, Gifford LM, Lauber-Huber C et al: Clinical Safety of Gadopentetate Dimeglumine. *Radiology* 1995; 196:439-443.

Peterman SB, Steiner RE, Bydder GM, et al: Nuclear Magnetic Resonance Imaging (NMR), (MRI), of Brain Stem Tumors. *Neuroradiology* 1985; 27:202-207.

Runge VM, Clanton JA, Herzer WA, et al: Intravascular Contrast Agents Suitable for Magnetic Resonance Imaging. *Radiology* 1984; 153:171-176.

Runge VM, Clanton JA, Price AC, et al: The Use of Gd DTPA as a Perfusion Agent and Marker of Blood-Brain Barrier Disruption. *Magn Reson Imaging* 1985; 3(1):43-55.

Wesbey GE, Engelstad BL, Brasch RC: Paramagnetic Pharmaceuticals for Magnetic Resonance Imaging. *Physiol Chem Phys Med NMR* 1984; 16:145-155.

Magnevist®

Part 3 - Monographie (Canada / French-language document)

Revision Date: 21 March 2001

MONOGRAPHIE

MAGNEVIST®

Injection de gadopentétate de diméglumine

469 mg/mL (0,5 mmol/mL)

Pour administration intraveineuse

Produit de contraste
pour l'imagerie par résonance magnétique (IRM)

BERLEX CANADA INC.
2260, 32e Avenue
Lachine (Québec)
H8T 3H4

DATE DE PRÉPARATION : Le 11 août 1993

DATE DE RÉVISION : Le 21 mars 2001

®Marque déposée dont Berlex Canada inc., Lachine, Québec, est un usager inscrit.

MONOGRAPHIE

MAGNEVIST

Injection de gadopentétate de diméglumine

469 mg/mL (0,5 mmol/mL)

Pour administration intraveineuse

Classe thérapeutique

Produit de contraste
pour l'imagerie par résonance magnétique (IRM)

MODE D'ACTION ET PHARMACOLOGIE CLINIQUE

MAGNEVIST (gadopentétate de diméglumine) est un produit de contraste utilisé à titre diagnostique en imagerie par résonance magnétique (IRM). Le gadolinium est un élément appartenant à la classe des terres rares. Son ion (Gd^{+++}) possède sept électrons célibataires et est, par conséquent, doué de propriétés paramagnétiques. Le Gd^{+++} exerce un effet puissant sur le temps de relaxation longitudinale (T_1) des protons des atomes d'hydrogène, ce qui se traduit par une augmentation du contraste dans les images IRM. La chélation du Gd^{+++} par l'acide diéthylènetriamine pentaacétique (DTPA) permet d'obtenir un complexe stable fortement paramagnétique et bien toléré (gadopentétate de diméglumine).

L'ion gadolinium libre ne peut être utilisé cliniquement en raison de sa toxicité importante; toutefois, le métal chélaté est métaboliquement inerte. La composante organique du chélate n'est pas métabolisée de façon mesurable et le métal ne se dissocie pas. Après injection par voie intraveineuse de gadopentétate de diméglumine, l'ion méglumine se dissocie complètement du gadopentétate. Le chélate hydrophile est distribué seulement dans le liquide extracellulaire et ne franchit pas la barrière hémato-encéphalique intacte. Le gadopentétate est excrété sous forme inchangée dans les urines. Il est rapidement éliminé par les reins, sa clairance étant identique à celle de l'inuline (aucune réabsorption tubulaire).

Le profil pharmacocinétique du gadopentétate de diméglumine administré par voie intraveineuse chez des sujets sains obéit à un modèle ouvert bicompartimental; la demi-vie de distribution moyenne est d'environ 0,2 heure et la demi-vie d'élimination moyenne, d'environ 1,6 heure. Après injection de 0,1 mmol/kg, environ 80 % de la dose est excrétée dans les urines en moins de 6 heures, et 93 %, en moins de 24 heures. L'excrétion fécale est inférieure à 0,1 % sur une période de 5 jours. Aucune biotransformation, dissociation ou décomposition du gadopentétate n'est décelée.

Administré selon la posologie recommandée, MAGNEVIST n'a aucun effet pharmacodynamique à l'exception d'une légère élévation de l'osmolalité plasmatique.

INDICATIONS ET USAGE CLINIQUE

MAGNEVIST (gadopentétate de diméglumine), injectable par voie intraveineuse, est indiqué pour rehausser le contraste dans les examens IRM du crâne et du rachis chez l'adulte et l'enfant, et ainsi améliorer la détection des lésions liées à une vascularisation anormale ou susceptibles d'altérer la barrière hémato-encéphalique.

MAGNEVIST est également indiqué pour les examens IRM chez l'adulte pour rehausser le contraste et faciliter la visualisation des lésions dont la vascularisation est anormale dans la tête (examen extra-crânien) et le cou.

CONTRE-INDICATIONS

MAGNEVIST (gadopentétate de diméglumine) ne doit pas être administré aux patients dont l'hypersensibilité au produit est connue ou soupçonnée.

MISES EN GARDE

La décision d'administrer MAGNEVIST (gadopentétate de diméglumine) doit être prise après une évaluation minutieuse des risques et des avantages chez les patients prédisposés aux allergies ou à l'asthme bronchique, l'expérience ayant montré que les réactions d'hypersensibilité sont plus fréquentes chez ces patients.

Dans de très rares cas, des réactions anaphylactoïdes, y compris un choc anaphylactique, peuvent survenir après injection de MAGNEVIST (gadopentétate de diméglumine) par voie intraveineuse. Advenant une telle réaction, il importe de bien connaître les mesures d'urgence à prendre et d'avoir sous la main les médicaments et les appareils nécessaires (p. ex. sonde endotrachéale et respirateur) afin d'intervenir sans délai.

Des études *in vitro* ont démontré que les hématies falciformes désoxygénées s'alignent perpendiculairement à un champ magnétique, ce qui peut se traduire *in vivo* par des occlusions vasculaires. Il se pourrait que l'augmentation du moment magnétique par le gadopentétate de diméglumine accentue l'alignement des hématies falciformes. MAGNEVIST n'a pas été étudié chez les patients présentant une anémie à hématies falciformes ou d'autres hémoglobinopathies.

Aucune étude n'a été menée chez les enfants atteints d'insuffisance rénale ou hépatique grave, ou d'hypertension artérielle cliniquement instable ou non contrôlée, ni chez les nouveau-nés prématurés.

Les examens IRM avec MAGNEVIST doivent être réalisés par un médecin ayant reçu la formation nécessaire et possédant une connaissance approfondie de la marche à suivre.

PRÉCAUTIONS

Généralités

MAGNEVIST (gadopentétate de diméglumine) doit être administré uniquement en injection par voie intraveineuse. En cas d'administration extravasculaire ou de diffusion dans l'espace interstitiel, MAGNEVIST provoque de la douleur et une irritation des tissus.

Les patients qui reçoivent MAGNEVIST en bolus peuvent percevoir brièvement une saveur sucrée.

Affections hémolytiques

Le gadopentétate de diméglumine modifie la morphologie des érythrocytes, ce qui résulte en une légère hémolyse extravasculaire (splénique) passagère et en une augmentation des taux de fer sérique et de bilirubine totale. Bien que cet effet ne se soit pas révélé cliniquement important au cours des essais, la prudence est de rigueur chez les patients atteints d'une affection hépatique ou d'une maladie hémolytique.

Convulsions

Bien qu'aucune preuve n'indique que MAGNEVIST soit la cause directe de convulsions, la possibilité qu'il abaisse le seuil convulsif chez les patients prédisposés aux convulsions ne peut être exclue. Des mesures de précaution doivent être prises à l'endroit de ces patients; notamment, il faut exercer une surveillance étroite et se tenir prêt à injecter des anticonvulsivants au besoin (voir **POSOLOGIE ET ADMINISTRATION**).

Grossesse

L'innocuité de MAGNEVIST pendant la grossesse n'a pas encore été établie. MAGNEVIST ne devrait être utilisé pendant la grossesse que si les avantages éventuels pour la mère justifient le risque éventuel pour le foetus.

Allaitement

MAGNEVIST peut être excrété dans le lait maternel. Par conséquent, la mère devrait cesser d'allaiter pendant 24 heures après l'administration de MAGNEVIST et ne pas utiliser le lait extrait pendant cette période.

Personnes âgées

Aucune précaution particulière n'est requise chez les patients âgés.

EFFETS INDÉSIRABLES

Généralités

La plupart des effets indésirables de MAGNEVIST (gadopentétate de diméglumine) surviennent rapidement après l'injection. La possibilité d'une réaction tardive ne peut cependant pas être exclue. Les effets indésirables le plus fréquemment signalés après l'administration de MAGNEVIST sont :

Céphalées	(8,7 %)*
Céphalées intenses	(1,3 %)
Inconfort au point d'injection	(6,7 %)
Nausées	(3,2 %)
Douleur localisée en d'autres parties du corps (dos, oreilles, yeux, dents)	(2,8 %)
Réactions d'hypersensibilité de la peau et des muqueuses	(2,1 %)
Étourdissements	(1,5 %)
Vomissements	(1,2 %)
Paresthésies	(1,2 %)

* 42,3 % des céphalées n'étaient pas considérées comme liées à l'administration de MAGNEVIST.

Dans le cadre d'essais cliniques, des effets indésirables se sont manifestés chez 11 enfants sur 319 (3,4 %) qui avaient reçu MAGNEVIST (céphalées, vasodilatation, étourdissements, diarrhée, douleur à l'oreille, tachycardie, fièvre, oedème, convulsions, vomissements, nausées et urticaire). Ce

profil d'effets indésirables est compatible avec celui qui a été observé chez l'adulte.

MAGNEVIST provoque une irritation tissulaire et de la douleur en cas d'administration extravasculaire.

La tension artérielle peut augmenter ou diminuer de façon passagère après l'administration de MAGNEVIST. Ces variations sont généralement sans conséquence, bien que 3 cas d'hypotension cliniquement importante soient survenus de 2 à 6 heures après l'administration de MAGNEVIST. Aucun lien n'a pu être établi entre cet effet et le produit de contraste injecté, mais la prudence est conseillée aux patients qui doivent conduire un véhicule moteur ou manœuvrer des machines.

L'expérience clinique a révélé que MAGNEVIST est rarement associé à des effets indésirables graves. De très rares cas de réactions anaphylactiques ont été signalés après la commercialisation. Par ailleurs, quatre patients qui avaient des antécédents d'épilepsie ont manifesté des crises convulsives.

Modifications des constantes biologiques

Une légère élévation réversible des taux de fer sérique et de bilirubine totale par rapport aux valeurs de base est observée chez la plupart des patients après l'administration de MAGNEVIST. Ces modifications ne semblent pas cliniquement pertinentes. Aucun lien n'a été établi entre l'emploi de MAGNEVIST et d'autres perturbations des constantes biologiques (élévations transitoires des épreuves de la fonction hépatique).

Profil des effets indésirables

Les effets indésirables suivants, énumérés par système ou appareil, ont été signalés après l'administration de MAGNEVIST.

Appareil cardio-vasculaire : Hypotension, vasodilatation, pâleur, phlébite, modifications non spécifiques de l'ECG, douleur rétrosternale, angine

Système nerveux central : Céphalées, étourdissements, agitation, paresthésies, acouphènes, anomalies du champ visuel, convulsions, hyperesthésies

Appareil digestif : Nausées, vomissements, douleurs gastro-intestinales, gastralgies, soif, salivation accrue, altération du goût

Appareil respiratoire : Sécheresse de la bouche, irritation de la gorge, rhinorrhée, respiration sifflante, éternuements, laryngisme, toux, dyspnée/apnée

Membranes cutanéo-muqueuses : Éruption, transpiration, urticaire, prurit

Autres : Inconfort au point d'injection (sensation de froid, de brûlure, de chaleur, douleur), odontalgie, faiblesse généralisée, fièvre, oedème localisé, asthénie, réactions anaphylactoïdes (caractérisées par des symptômes cardio-vasculaires, respiratoires et cutanés), conjonctivite

Constantes biologiques : Élévation transitoire des taux de fer sérique et de bilirubine totale

Les effets indésirables suivants ont également été signalés. Un rapport causal n'a toutefois pas été établi ni réfuté.

Appareil cardio-vasculaire : Hypertension, tachycardie, syncope, décès lié à un infarctus du myocarde ou à d'autres causes indéterminées

Système nerveux central : Diplopie, migraine, anxiété, somnolence, nystagmus, stupeur

Appareil digestif : Constipation, diarrhée, anorexie

Membranes cutanéo-muqueuses : Oedème du visage, érythème, épidermolyse

Autres : Douleur localisée (dos, oreilles, yeux)

SYMPTÔMES ET TRAITEMENT DU SURDOSAGE

En cas de surdosage ou d'insuffisance rénale grave, MAGNEVIST (gadopentétate de diméglumine) peut être extrait par hémodialyse.

POSOLOGIE ET ADMINISTRATION

L'examen avec MAGNEVIST (gadopentétate de diméglumine) ne requiert aucune préparation particulière du patient. Cependant, des mesures de précaution doivent être prises dans le cas de sujets prédisposés aux convulsions; il faut notamment exercer une surveillance étroite et se tenir prêt à injecter des anticonvulsivants au besoin **(**voir **PRÉCAUTIONS)**. Les règles habituelles de sécurité en IRM doivent être observées (p. ex. exclusion des sujets porteurs d'agrafes vasculaires ferromagnétiques).

La sédation peut être nécessaire chez les jeunes enfants, les nourrissons et les nouveau-nés avant un examen par IRM, afin d'éviter que les images ne soient faussées par les mouvements du patient.

Les directives suivantes concernant la posologie s'appliquent aux adultes et aux enfants (y compris les nourrissons et les nouveau-nés) :

**Dose recommandée :**	0,2 mL/kg (0,1 mmol/kg)
**Voie d'administration :**	intraveineuse<br>(dans une grosse veine, si possible)
**Vitesse d'injection :**	10 mL/min<br>ou<br>10 mL/15 sec en bolus
**Dose maximale totale :**	20 mL

Pour assurer l'injection complète du produit de contraste, il est recommandé de faire suivre l'injection de 5 mL de sérum physiologique.

Si la présence d'une lésion intracrânienne ou intrarachidienne est toujours soupçonnée malgré des clichés IRM normaux, la puissance diagnostique de l'examen peut être améliorée par l'administration d'une injection additionnelle de MAGNEVIST. Le cas échéant, une dose additionnelle équivalant à la dose initiale totale doit être injectée dans un délai de 30 minutes et l'examen IRM doit être répété.

MAGNEVIST ne doit être retiré de son contenant qu'au moment de son utilisation. Toute portion non utilisée doit être jetée après l'examen.

Les images pondérées en T_1 conviennent particulièrement aux examens avec produit de contraste.

MAGNEVIST s'est révélé efficace à des intensités de champ magnétique très variables (0,14 à 1,5 Tesla).

Note importante

L'examen IRM doit être réalisé en moins d'**une heure.** Le contraste optimal est généralement observé en moins de 27 minutes après injection de MAGNEVIST dans les examens du crâne, et pendant la phase suivant immédiatement l'injection (10 à 30 minutes) dans les examens du rachis.

Chez les nouveau-nés et les nourrissons, un contraste optimal du SNC a été observé pendant plusieurs heures après l'administration de MAGNEVIST.

INFORMATION PHARMACEUTIQUE

Substance médicamenteuse

Nom commercial : MAGNEVIST

Dénomination commune : Gadopentétate de diméglumine (USAN)

Dénomination chimique : Gadolinate(2-),[N,N-bis[2-[bis(carboxyméthyl)amino]éthyl]glycinato(5-)]-, dihydrogène, combiné à 1-déoxy-1-(méthylamino)-D-glucitol(1:2)

Formule développée :

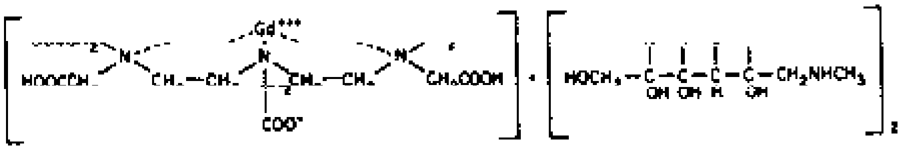

Formule moléculaire : $C_{14}\ H_{20}\ Gd\ N_3\ O_{10} \cdot (C_7\ H_{17}\ NO_5)_2$

Masse moléculaire : 938,02

Solubilité : Très soluble dans l'eau

Osmolalité : 1960 mOsm/kg H_2O à 37 °C

Composition

MAGNEVIST, injectable par voie intraveineuse, se présente sous forme de solution aqueuse, stérile et limpide, incolore ou jaune clair. Un mL contient 469,01 mg de sel de diméglumine de l'acide gadopentétique (équivalant à 0,5 mmol/mL), 0,99 mg de méglumine et 0,40 mg d'acide diéthylènetriamine pentaacétique.

Stabilité et entreposage

MAGNEVIST doit être conservé à une température entre 15 et 30 °C et à l'abri de la lumière.

PRÉSENTATION

MAGNEVIST (gadopentétate de diméglumine) est une solution aqueuse, stérile et limpide, incolore ou jaune clair.

MAGNEVIST est présenté en flacons à dose unique de 20 mL, 15 mL et 10 mL emballés individuellement.

MAGNEVIST doit être conservé à une température entre 15 et 30 °C et à l'abri de la lumière.

PHARMACOLOGIE

Études chez l'animal

Neuropharmacologie

La neuropharmacologie du gadopentétate de diméglumine a été évaluée chez le rat après une injection unique péricérébrale ou intracisternale. La détermination de la DE_{50}, basée sur les anomalies de posture, les crises épileptiques ou la mort, et de la DL_{50} ont indiqué que le gadopentétate de diméglumine est considérablement moins toxique que le chlorure de gadolinium ou le diatrizoate de méglumine. Dans une étude similaire, la tolérance neurale au gadopentétate de diméglumine n'a pas été modifiée par l'addition de 1,0 mg ou moins de DTPA libre/mL (voir le tableau ci-dessous).

Comparaison de la DE_{50} et de la DL_{50} de gadopentétate de diméglumine, de chlorure de gadolinium et de diatrizoate de méglumine après administration péricérébrale ou intracisternale chez des rats.

Produits	Doses administrées (µmol/kg)	DE_{50} (µmol/kg)	Doses administrées (µmol/kg)	DL_{50} (µmol/kg)
**Administration péricérébrale**				
Gadopentétate de diméglumine	25-296,3	96,6<br>97,1	463-1852	1141,4<br>1227,3
Gadopentétate de diméglumine et 1,0 mg de DTPA/mL	25-296,3	80,2	463-1852	1063,4
Chlorure de gadolinium	5-25	10,8	6-100	14,9
Diatrizoate de méglumine	32-53	35,0	32-53	42,8
**Administration intracisternale**				
Gadopentétate de diméglumine	16,7-197,9	74,0<br>86,2	309-1233<br>a	654,9<br>a
Gadopentétate de diméglumine et 0,15 mg de DTPA/mL	16,7-197,9	80,0	a	a
Gadopentétate de diméglumine et 1,0 mg de DTPA/mL	16,7-197,9	85,0	a	a
Chlorure de gadolinium	3,3-16,7	5,6	4-17	8,1
Diatrizoate de méglumine	4-21	11,2	32-126	54,9

a - Non évaluée dans l'étude

Effets cardio-vasculaires et hémodynamiques

Les effets cardio-vasculaires et hémodynamiques du gadopentétate de diméglumine ont été évalués chez des chiens sains anesthésiés ayant reçu par voie intraveineuse 0,25 ou 1,25 mmol/kg de poids corporel. À la dose la plus faible, une légère augmentation de la résistance périphérique a été notée. Chez les chiens ayant reçu 1,25 mmol/kg, le gadopentétate de diméglumine a entraîné initialement une diminution de la résistance périphérique, de la tension artérielle et de la fréquence cardiaque, de même qu'une augmentation de la pression télédiastolique du ventricule gauche, du volume systolique et du débit cardiaque. Par la suite, on a noté une augmentation de la résistance périphérique et une élévation sensible de la tension artérielle qui s'est maintenue au même niveau pendant le reste de l'expérience.

Les effets hémodynamiques du gadopentétate de diméglumine en présence d'une insuffisance cardiaque aiguë d'origine ischémique ont également été évalués chez des chiens qui avaient reçu des doses de 0,25 mmol/kg et 0,75 mmol/kg par voie intraveineuse. À la dose de 0,25 mmol/kg, on a noté, d'une part, une légère diminution de la pression diastolique et de la résistance périphérique, et, d'autre part, une faible augmentation de la vitesse de variation de la pression intraventriculaire gauche (dp/dt), du débit cardiaque et de l'index systolique. Tous les paramètres sont revenus à la normale cinq à dix minutes après

l'administration. À la dose de 0,75 mmol/kg, une réaction passagère similaire a été observée au chapitre des paramètres hémodynamiques.

Tolérance rénale

La tolérance rénale au gadopentétate de diméglumine a été étudiée chez des lapins à qui on avait administré 2 mmol/kg par voie intraveineuse. Chez les lapins ayant reçu du gadopentétate de diméglumine, on a constaté une légère modification de la protéinurie comparativement au groupe témoin qui avait reçu une solution de sorbitol; toutefois, on a observé une meilleure tolérance rénale au gadopentétate de diméglumine qu'aux autres produits de contraste utilisés en imagerie par rayons X. Aucune modification des taux sériques de créatinine ou d'azote uréique n'a été observée, lesquels ont servi d'indicateurs de la fonction rénale. En outre, aucune modification tissulaire des reins n'a été décelée après la période d'observation de une semaine.

Propriétés physico-chimiques et biochimiques

Les propriétés pharmacologiques du gadopentétate de diméglumine ont été évaluées au moyen d'une série d'épreuves *in vitro* et *in vivo* chez le chien, le lapin et le babouin après injection intraveineuse. Ces épreuves ont démontré que le gadopentétate de diméglumine était très hydrophile et, par conséquent, qu'il ne pouvait pas se lier aux protéines ni exercer d'effet sur l'activité

enzymatique. En résumé, le produit était physiologiquement inerte aux concentrations susceptibles d'être utilisées chez l'homme.

Effet sur la coagulation

L'effet *in vitro* du gadopentétate de diméglumine sur la coagulation a été évalué par une thrombo-élastographie pratiquée sur du sang citraté de chien. La thrombo-élastrographie a révélé que les doses allant jusqu'à 29 mmol/L n'influaient pas sur la coagulation du sang citraté de chien comparativement à une thrombo-élastographie témoin réalisée au moyen d'une solution saline isotonique.

Efficacité

L'efficacité du gadopentétate de diméglumine a été démontrée chez le rat, le lapin et le babouin, après injection par voie intraveineuse aux fins d'IRM. Des doses de 0,01 à 1,0 mmol/kg de poids corporel administrées par voie intraveineuse ont rehaussé le contraste entre le tissu sain et le tissu pathologique (infarctus, tumeurs et inflammations). Comme le gadopentétate de diméglumine était excrété dans les urines, il a également rehaussé le contraste au niveau rénal chez le rat, à des doses aussi faibles que 0,01 mmol/kg de poids corporel.

Pharmacocinétique

Le gadopentétate de diméglumine, administré par voie orale ou intraveineuse, ou les deux, a fait l'objet d'études chez le rat (mâles et femelles en période de gestation ou de lactation), le lapin (femelles gravides), le chien (femelles) et le babouin (mâles) visant à l'évaluer aux titres de l'absorption, de la distribution, du métabolisme et de l'excrétion.

Après administration orale, le gadopentétate de diméglumine marqué n'a pas été absorbé ou ne l'a été qu'en très faible proportion par le tractus gastro-intestinal chez le rat et le chien, et a été excrété presque entièrement dans les fèces (environ 100 % chez le rat et 94 % chez le chien).

Après injection intraveineuse, le produit a été excrété principalement dans les urines (90 % chez le rat et > 96 % chez le chien). Chez le rat, en présence d'occlusion rénale bilatérale, 2 % de la dose de gadopentétate marqué a été excrétée par voie biliaire en 4 heures.

Les injections intraveineuses de gadopentétate de diméglumine n'ont entraîné aucune accumulation notable dans les tissus étudiés chez le rat, le lapin, le chien ou le babouin. Toutefois, chez le rat présentant une insuffisance rénale totale, 3,5 % de la dose de gadopentétate de diméglumine marqué a été sécrétée

dans l'estomac et l'intestin 4 heures après l'administration par voie intraveineuse. Ces résultats semblent indiquer que le gadopentétate de diméglumine peut être sécrété dans le tractus gastro-intestinal, particulièrement en présence d'insuffisance rénale grave.

Après administration par voie intraveineuse de doses uniques de gadopentétate de diméglumine marqué (0,5 mmol/kg) chez des lapines gravides, la concentration maximale de gadolinium marqué chez le foetus a été atteinte en 30 minutes. Chez la mère, les concentrations dans le plasma, le foie, le coeur et l'utérus sont demeurées stables après 15 et 30 minutes. Les concentrations dans les tissus foetaux équivalaient, après 15 minutes, à environ 4 % des concentrations plasmatiques chez la mère, et, après 30 minutes, à environ 8 % (ce qui correspond respectivement à 0,11 % et à 0,26 % de la dose totale). À 120 minutes, les concentrations foetales avaient diminué pour s'établir à 1/4 des valeurs maximales. Chez le foetus, la demi-vie d'élimination était comparable à celle qui est observée dans le plasma et les tissus maternels, soit 30 à 50 minutes.

Après injections intraveineuses chez des rates gravides, le gadopentétate de diméglumine marqué s'est distribué rapidement, n'a pas franchi les barrières hémato-encéphalique et placentaire et a été éliminé en moins de 24 heures.

Chez les rates ayant reçu pendant l'allaitement des injections intraveineuses de gadopentétate de diméglumine marqué, moins de 0,2 % de la dose administrée est passée dans le lait maternel.

Chez le chien, rien n'indique que le gadopentétate de diméglumine marqué administré par voie intraveineuse est métabolisé lors du passage dans l'organisme. Une chromatographie en phase liquide haute performance n'a détecté aucun ion gadolinium non chélaté chez les animaux.

Études chez l'homme

Pharmacocinétique

Le profil pharmacocinétique de MAGNEVIST (gadopentétate de diméglumine) a été étudié auprès de sujets volontaires de sexe masculin dont les reins et la vessie ont été examinés en imagerie par résonance magnétique (IRM) au cours d'une étude ouverte sur l'innocuité et l'efficacité du produit menée en Europe. Une dose unique de MAGNEVIST a été injectée dans la veine cubitale chez 20 volontaires sains de sexe masculin. Quatre doses différentes, allant de 0,005 à 0,25 mmol/kg, ont été évaluées chez des groupes de 5 participants.

Pour les deux doses les plus élevées (0,1 et 0,25 mmol/kg), les données pharmacocinétiques sur les concentrations plasmatiques

en fonction du temps ont révélé que le devenir du gadopentétate de diméglumine dans l'organisme obéit à un modèle bicompartimental; la demi-vie de distribution moyenne était de 0,2 heure, et la demi-vie d'élimination moyenne, de 1,6 heure. Aux doses de 0,1 et 0,25 mmol/kg, la cinétique du médicament n'était pas fonction de la dose. Le gadopentétate est éliminé uniquement par voie urinaire, l'excrétion moyenne pour les quatre doses étant de 83 % en moins de 6 heures et de 91 % en l'espace de 24 heures après l'injection. Aucun métabolite du gadopentétate n'a été décelé dans les urines, ce qui indique que le gadopentétate, ingrédient actif du produit de contraste pour l'IRM, demeure inchangé.

Les taux d'élimination urinaire et plasmatique (111 ± 19 mL/min et 122 ± 14 mL/min, respectivement) du gadopentétate sont essentiellement identiques. Le volume de distribution (266 ± 43 mL/kg) équivaut au volume de liquide extracellulaire, et la clairance est comparable à celle de substances soumises à la filtration glomérulaire, c'est-à-dire l'inuline et le CrEDTA. Chez l'homme, la demi-vie d'élimination plasmatique (1,6 heure) est comparable à celle qui a été observée chez le chien et à celle des produits de contraste classiques généralement utilisés en urographie et en angiographie.

Épreuves de laboratoire

Les épreuves de laboratoire révèlent une élévation du taux de fer sérique et, dans quelques cas, du taux de bilirubine sérique, nettement liée à la prise du médicament. Chez environ 15 % des patientes et 30 % des patients, une augmentation des taux de fer sérique au delà des valeurs de base a été constatée. Les taux ont augmenté en l'espace de 2 à 4 heures suivant l'injection et se sont abaissés en moins de 24 heures après l'injection. Quarante-huit heures après l'injection, les taux étaient revenus aux valeurs de départ. Le taux d'hémoglobine, l'hématocrite, la numération érythrocytaire et le taux d'enzymes hépatiques n'ont pas été perturbés. L'absence de modification de ces paramètres s'expliquerait par une hémolyse, probablement extravasculaire, dont l'importance est négligeable et qui n'a pas d'effet sur le taux d'hémoglobine, l'hématrocrite et la numération érythrocytaire.

Bien que l'administration de MAGNEVIST ne soit pas risquée chez les patients dont l'état hématologique est normal, elle peut l'être chez les patients qui présentent une anémie hémolytique, étant donné que le gadopentétate de diméglumine semble exercer un effet sur la morphologie des érythrocytes. Environ 8 % des patients qui ont une augmentation du taux de fer sérique présentent également une élévation du taux de bilirubine sérique, ce qui s'expliquerait par une diminution, chez ces patients, de la conjugaison de la bilirubine résultant de l'hémolyse.

Essais cliniques chez l'adulte présentant des lésions du crâne et du rachis

L'efficacité de MAGNEVIST (gadopentétate de diméglumine) comme produit de contraste utilisé en IRM pour le diagnostic et l'évaluation des lésions du cerveau, du rachis et des tissus connexes a été démontrée dans six essais cliniques clés et dans trois essais particuliers où les clichés ont été interprétés par des radiologistes indépendants.

Dans les six essais cliniques, l'efficacité du produit a été évaluée auprès de 597 patients au total (571 d'entre eux ayant reçu MAGNEVIST, et 26, un placebo). Chez 196 des patients ayant reçu Magnevist (55 en imagerie du crâne et 141 en imagerie du rachis), les clichés ont été réévalués par des radiologistes indépendants.

L'efficacité a été évaluée par différents moyens : mesures de l'intensité du signal, évaluation de l'efficacité globale ainsi que de la qualité des clichés (sur le plan du contraste, du détail morphologique et de l'apport diagnostique).

Rehaussement du contraste : Après injection de MAGNEVIST, on a noté une augmentation de l'intensité du signal pour tous les types de tissus évalués (tissu sain, lésions, œdème et nécrose). Des mesures comparatives de l'intensité du signal, qui indiquaient

le contraste relatif entre les types de tissu, ont été faites pour les examens, avant et après injection de MAGNEVIST. MAGNEVIST a augmenté de façon importante la différence des mesures d'intensité du signal entre les lésions, l'œdème et le tissu sain comparativement à la différence qui existait entre ces tissus dans les examens prétraitement. Des augmentations similaires du contraste ont été observées dans les comparaisons lésion - œdème et lésion - nécrose.

Dans cinq des six essais (imageries du crâne et du rachis), le rehaussement du contraste a été évalué d'après l'augmentation de l'intensité du signal d'une lésion par rapport aux tissus qui l'entourent. On a noté un rehaussement chez 292 patients sur 339 (86 %) après injection de MAGNEVIST. Aucun rehaussement n'a été relevé dans les examens réalisés chez les 26 patients ayant reçu un placebo.

Dans quatre des six essais, d'autres lésions ont été décelées chez 113 patients sur 466 (24 %) après administration de MAGNEVIST.

Apport diagnostique : L'utilisation de MAGNEVIST a amélioré ou facilité le diagnostic chez 107 patients sur 162 (66 %) dans les

examens du crâne. Dans les examens du rachis, l'établissement du diagnostic a été facilité dans 131 cas sur 169 (78 %).

Modifications du diagnostic : Dans les études sur l'imagerie du crâne et du rachis, le rehaussement de contraste obtenu à l'examen avec MAGNEVIST a amené les expérimentateurs à modifier le diagnostic chez 129 patients sur 317 (41 %). Les lésions crâniennes rehaussées par MAGNEVIST étaient compatibles avec le tableau clinique dans 95 % des cas. Les modifications du diagnostic les plus fréquentes en imagerie crânienne portaient sur les néoplasmes non spécifiques, les méningiomes, les métastases et les gliomes. En imagerie rachidienne, les changements étaient attribuables le plus souvent à une meilleure différenciation entre le tissu cicatriciel et le tissu discal anormal (études sur les lombo-dorsalgies post-opératoires) et à une meilleure délimitation des lésions rachidiennes (taille, siège et configuration modifiés) chez les patients qu'on soupçonnait d'être porteurs d'une tumeur rachidienne.

Évaluation des clichés : L'évaluation des clichés a révélé une amélioration du contraste chez 2/3 des patients sur les images pondérées en T_1 et chez plus de 1/3 des patients sur les images pondérées en T_2. Dans les études sur l'imagerie crânienne, l'injection de MAGNEVIST a rendu le diagnostic possible chez

122 patients sur 167 (73 %) pour qui des clichés antérieurs pondérés en T_1 et en T_2 n'avaient eu aucune valeur diagnostique.

L'évaluation des clichés du crâne et du rachis faite par les radiologistes indépendants révèle une amélioration considérable du nombre de lésions détectées après injection de MAGNEVIST. Cette amélioration peut avoir des conséquences importantes sur le pronostic et le traitement, particulièrement chez les patients où le rehaussement de contraste amène une modification du diagnostic fondée, par exemple, sur la découverte de résultats positifs auparavant négatifs ou de résultats dénotant un cancer métastatique plutôt qu'une lésion unique. On a également constaté que l'emploi de MAGNEVIST accroît sensiblement la précision diagnostique comparativement à l'IRM sans produit de contraste ou à la tomodensitométrie (TDM).

Modalité diagnostique (séquence d'impulsions) : En imagerie crânienne, les images pondérées en T_1 ont permis un meilleur contraste chez 138 patients sur 148 (93 %). Chez les 10 autres patients (7 %), la pondération en T_2 s'est avérée la modalité diagnostique optimale. En imagerie rachidienne (lombo-dorsalgies post-opératoires), les images pondérées en T_1 ont donné un meilleur contraste chez 55 patients sur 58 (95 %), alors que les images pondérées en T_2 ont donné un meilleur contraste chez les 3 autres patients (5 %).

Moment du meilleur cliché : Dans les études sur l'imagerie crânienne, le moment optimal pour la prise des clichés a été déterminé sur la base de l'évaluation de l'efficacité globale et de la qualité du contraste des clichés. L'évaluation de ces deux paramètres a démontré que les meilleures images au titre de l'apport diagnostique sont obtenues peu après l'injection. Chez 108 des 148 patients qui ont passé un examen avec produit de contraste (73 %), le meilleur cliché a été obtenu moins de 27 minutes après l'injection de MAGNEVIST. Pour plus de la moitié de ces 108 patients, le meilleur cliché a été pris moins de 14 minutes après l'injection du produit de contraste. En imagerie rachidienne, ce sont également les clichés pris peu après l'injection (10 à 30 minutes) qui ont été les meilleurs.

Études cliniques chez l'enfant présentant des lésions du crâne et du rachis

L'efficacité de Magnevist a été démontrée au cours de deux études cliniques pivots portant sur 142 enfants âgés de 0 à 18 ans. Chez tous les sujets, un diagnostic préliminaire d'anomalie du SNC avait été posé à la suite d'examens par des méthodes autres que l'IRM. Tous les patients ont subi un examen par IRM avant et après l'administration de 0,2 mL/kg (0,1 mmol/kg) de MAGNEVIST. Certains sujets ont reçu une dose additionnelle de

0,1 mmol/kg dans les 30 minutes suivant la première dose si cela s'avérait nécessaire pour poser le diagnostic.

Évaluation du contraste : Après l'injection de MAGNEVIST, le rapport contraste/bruit des images obtenues par résonance magnétique a sensiblement augmenté, augmentation d'autant plus marquée chez les patients recevant une deuxième injection de MAGNEVIST. Le rapport de l'intensité du signal dans les lésions et dans les tissus sains a significativement augmenté pour les examens de la tête et du rachis pondérés en T1 après l'injection de MAGNEVIST.

D'après les évaluations des investigateurs, le contraste entre les lésions et les tissus sains, ainsi que la démarcation des lésions par rapport aux tissus environnants, se sont améliorés après l'injection de MAGNEVIST. Dans la plupart des cas, la cote attribuée est passée de «nul» ou «faible» à «excellent».

Utilité diagnostique : MAGNEVIST a considérablement amélioré la possibité de poser un diagnostic définitif. Dans les cas où des lésions ont été constatées sur les clichés pondérés en T1 ou en T2 (n=57), cette possibilité est passée de 44 % avant l'injection de MAGNEVIST à 74 % après l'injection de MAGNEVIST. La qualité diagnostique des clichés pondérés en T1 et en T2 a considérablement augmenté après l'injection de MAGNEVIST,

qu'il s'agisse de clichés normaux ou de clichés révélant des lésions.

Chez 11 patients sur 70 (16 %), la morphologie des lésions était mieux caractérisée après l'injection de MAGNEVIST. Ainsi, il était possible de mieux évaluer la composante kystique, nécrotique, tumorale ou vasculaire de la lésion. Un gain statistiquement significatif de l'information utile sur le plan diagnostique a été documenté chez 22 patients sur 40 (55 %).

MAGNEVIST s'est avéré utile chez 40 patients sur 70 (57 %). Dans 14 de ces 40 cas, l'IRM final a permis de conclure à l'absence d'anomalie; dans 14 autres cas, une lésion a été constatée seulement après l'injection de MAGNEVIST; dans 6 autres cas, le diagnostic final n'a pu être établi qu'après l'injection de MAGNEVIST; dans 3 cas, une résection tumorale complète a été confirmée par l'absence de contraste; dans 2 cas, la composante solide, kystique ou nécrotique de la lésion a été caractérisée plus précisément; finalement, dans un cas, la taille de la lésion a été précisée.

Études cliniques chez l’adulte présentant des lésions de la tête et du cou

L’efficacité de MAGNEVIST comme produit de contraste utilisé en IRM a été évalué chez 87 patients présentant des lésions de la tête (extra-crâniennes) et du cou. Des jeux de clichés provenant

de 78 de ces patients ont été également évalués par des radiologues (en aveugle) qui n’avaient pas participé aux études cliniques et ne connaissaient pas les antécédents des patients. L’efficacité a été mesurée grâce à des analyses comparatives de l’amélioration du contraste, de la facilitation de la visualisation et de la qualité du contraste des clichés pris après administration de MAGNEVIST et des clichés correspondant pris avant l’administration de MAGNEVIST.

Au cours des études cliniques, l’amélioration du contraste des lésions après administration de MAGNEVIST a été démontrée chez 78 (90 %) patients sur 87. Lorsqu’elle a été évaluée par des radiologues en aveugle, elle a été démontrée pour 56 (85 %) jeux de clichés sur les 66 qui ont été inclus dans les données finales.

On a démontré que la visualisation des lésions était facilitée principalement en montrant que les clichés pris après l’administration de MAGNEVIST fournissent de l’information radiologique additionnelle sur des paramètres comme le lieu, la taille et la forme de la lésion et permettent de différencier un œdème d’une nécrose. Dans les études cliniques, les clichés pris après administration de MAGNEVIST ont fourni de l’information radiologique additionnelle pour 63 (72 %) patients sur 87. De plus, les radiologues qui ont travaillé en aveugle ont noté une amélioration significative ($p < 0,001$) dans la visualisation des lésions sur les clichés RM pris après administration de MAGNEVIST par rapport à ceux qui ont été pris avant administration de MAGNEVIST. Les clichés pris après

administration de MAGNEVIST permettaient de mieux visualiser les lésions que ceux pris avant dans le cas de 40 (67 %) clichés sur les 60 sur lesquels on pouvait déterminer la configuration des lésions. On a observé de l'information radiologique additionnelle sur 48 (73 %) clichés sur les 66 pris après administration de MAGNEVIST examinés par des radiologues en aveugle.

Les clichés RM pris chez chaque patient avant et après administration de MAGNEVIST ont été évalués sur une échelle de 4 points, l'intensité relative de la lésion étant mesurée par rapport au tissu (0 = absence de contraste; 1 = équivoque; 2 = bon; 3 = excellent). Chez 63 (73 %) patients sur les 86 qui ont participé aux études cliniques, la qualité du contraste après l'administration de MAGNEVIST était plus élevée qu'avant l'administration de MAGNEVIST (p < 0,001). Dans l'évaluation faite en aveugle, la qualité du contraste après administration de MAGNEVIST était plus élevée qu'avant l'administration de MAGNEVIST chez 36 (55 %) patients sur les 66 (p < 0,001).

TOXICOLOGIE

Toxicité aiguë

Des études sur la toxicité aiguë du gadopentétate de diméglumine ont été réalisées chez la souris, le rat et le chien après administration par voie intraveineuse, et chez la souris et le rat après administration par voie orale (voir le tableau à la page suivante).

TOXICITÉ AIGUË

Espèces, sexe prédominant (nombre d'animaux par groupe)	Mode d'adminis-tration, Dose (mmol/kg)	Valeurs de la DL_{50} (mmol/kg)	Observations cliniques
Souris, M (3)	oral; 0,25; 1,0; 5,0	> 5,0	Aucune
Souris, M (3)	i.v.; 2,5; 5,0; 6,25;7,5;10,0	5,0 à 7,5	Apathie, modification de la respiration, démarche pertur-bée
Souris, F (3)	i.v.; 6,25; 10,0; 12,5; 15,0	6,25 à 12,5	
Rats, M (3)	oral; 0,2; 0,8; 4,0	> 4,0	Aucune
Rats, M (3)	i.v.; 10,0 11,5; 13,5; 15,0	11,5 à 15,0	Prostration, apathie, respiration accélérée, démarche perturbée
Rats, F (3)	i.v.; 7,5,; 10,0; 12,5; 15,0	10,0 à 15,0	
Chiens, M+F (3)	i.v.; 6,0	> 6,0	Rubéfaction de la peau et des muqueuses, léchage, tremblements, hématurie, démarche perturbée, haut-le-coeur, vomissements et saignement au point d'injection.

Toxicité subaiguë

Espèces	Mode d'administration, Dose (mmol/kg)	Durée d'administration	Principales observations pertinentes
Rats<br>10/sexe/dose	i.v.;<br>1,0; 2,5; 5,0	5 doses/semaine<br>pendant 4 sem.	1,0 mmol/kg - Aucune observation.<br>2,5 mmol/kg et plus - Apathie, augmentation de la consommation d'eau, décubitus, détresse respiratoire, vacuoles dans les cellules épithéliales des tubes contournés et dans les cellules du parenchyme hépatique, légère diminution des paramètres hématologiques, augmentation pondérale absolue et relative du foie et des reins. Ces manifestations étaient toutes liées à la dose.<br>> 5 mmol/kg - Convulsions, diminution du gain de poids corporel, mort de la moitié des animaux.
Rats<br>5/mâle/dose	i.v.;<br>2,5; 5,0	1 ou 5 doses/sem.<br>pendant 4 sem.;<br>période de récupération de 8 et 16 jours	Vacuolisation rénale et hépatique liée à la dose et réversible avec le temps<br>> 5 mmol/kg - Atrophie des cellules spermatogènes non réversible en l'espace de 15 jours.
Chiens beagle<br>2/sexe/dose	i.v.;<br>0,25; 1,0; 2,5	5 doses/semaine<br>pendant 4 sem.	0,25 mmol/kg - Aucune observation.<br>1,0 mmol/kg et plus - Rubéfaction cutanée et vacuolisation des tubes proximaux en fonction de la dose.<br>2,5 mmol/kg - Augmentation du poids des reins, diminution du poids corporel, augmentation de la consommation d'eau.
Rates gravides<br>25/femelle/dose	i.v.;<br>0,25; 0,75; 1,25	1 dose/jour<br>pendant 10 jours<br>(6e au 15e jour de gestation)	0,25 à 0,75 mmol/kg - Aucune observation.<br>1,25 mmol/kg - Augmentation de la fréquence de malformations des côtes et léger retard de l'ossification chez le foetus.
Lapines gravides<br>21-22/femelle/dose	i.v.;<br>0,25; 0,75; 1,25	1 dose/jour<br>pendant 13 jours	0,25 mmol/kg - Aucune observation.<br>0,75 à 1,25 mmol/kg - Retard du développement foetal lié à la dose.

Études sur la mutagénicité

Le pouvoir mutagène du gadopentétate de diméglumine a été évalué *in vitro* au moyen de tests sur bactéries (*S. typhimurium, E. coli*) et de tests sur cultures de cellules de mammifères (recherche de mutation au locus HGPRT sur cellules V 79, test de réparation non programmée d'ADN sur hépatocytes, test de transformation cellulaire sur cellules C3H 10T1/2); *in vivo*, le produit a été évalué par le test du micronucléus et le test de dominance létale.

Aucun indice n'a permis de supposer que le gadopentétate de diméglumine possède un pouvoir mutagène, que ce soit *in vitro* ou *in vivo*.

Tolérance au point d'injection

Le gadopentétate de diméglumine a été évalué quant au risque d'irritation au point d'injection chez des lapins auxquels le produit avait été administré par voies intraveineuse, paraveineuse, intramusculaire et sous-cutanée. L'administration du gadopentétate de diméglumine par voie intraveineuse n'a entraîné que très peu de signes d'irritation. Toutefois, les injections paraveineuses, intramusculaires et sous-cutanées ont entraîné une irritation locale modérée.

RÉFÉRENCES

Allen ED, Byrd SE, Darling CF et coll. : The Clinical and Radiological Evaluation of Primary Brain Neoplasms in Children, Part II: Radiological Evaluation. *J National Med Assoc* 1993; 85: 546 - 553.

Brasch RC, Weinmann HJ, Wesby GE : Contrast-enhanced NMR Imaging: Animal Studies Using Gadolinium DTPA Complex. *AJR*, 1984; 142 : 625 - 630.

Bydder GM, Brown J, Niendorf P et coll. : Enhancement of Cervical Intraspinal Tumors in MR Imaging with Intravenous Gadolinium DTPA. *J Comput Assist Tomogr*, 1985; 9(5) : 847 - 851.

Carr DH : The Use of Proton Relaxation Enhancers in Magnetic Resonance Imaging. *Magn Reson Imaging*, 1985; 3(1) : 17 - 25.

Carr DH, Brown J, Bydder GM et coll. : Intravenous Chelated Gadolinium as a Contrast Agent in NMR Imaging of Cerebral Tumors. *Lancet*, 1984; 3 : 484 - 486.

Claussen C, Laniado M, Schorner W et coll. : Computer Tomographic and Nuclear Magnetic Resonance Diagnoses of Cerebral Tumors Using Conventional and Paramagnetic Contrast Medium. *Rontgen-B1*, 1985; 38 : 143 - 149.

Dietrich RB : MR and Gadolinium Define Pediatric Brain Pathology. *Diagnostic Imaging* 1989; February: 96 - 103.

Eldevik OP et Brunberg JA : Gadopentetate Dimeglumine-enhanced MR of the Brain: Clinical Utility and Safety in Patients Younger than Two Years of Age. *Amer J Neuroradiol* 1994; 15: 1001 - 1008.

Felix R, Schorner W, Laniado M et coll. : Brain Tumors: MR Imaging with Gadolinium DTPA. *Radiology*, 1985; 156 : 681 - 688.

Goldstein EJ, Burnett KR, Hansell JR et coll. : Gadolinium DTPA (an NMR Proton Imaging Contrast Agent): Chemical Structure, Paramagnetic Properties and Pharmacokinetics. *Physiol Chem Phys Med NMR*, 1984; 16 : 97 - 104.

Nelson KL, Gifford LM, Lauber-Huber C et coll. : Clinical Safety of Gadopentetate Dimeglumine. *Radiology* 1995; 196: 439 - 443.

Peterman SB, Steiner RE, Bydder GM et coll. : Nuclear Magnetic Resonance Imaging (NMR), (MRI), of Brain Stem Tumors. *Neuroradiology*, 1985; 27 : 202 - 207.

Runge VM, Clanton JA, Herzer WA et coll. : Intravascular Contrast Agents Suitable for Magnetic Resonance Imaging. *Radiology*, 1984; 153 : 171-176.

Runge VM, Clanton JA, Price AC et coll. : The Use of Gd DTPA as a Perfusion Agent and Marker of Blood-Brain Barrier Disruption. *Magn Reson Imaging*, 1985; 3(1) : 43 - 55.

Wesbey GE, Engelstad BL, Brasch RC : Paramagnetic Pharmaceuticals for Magnetic Resonance Imaging. *Physiol Chem Phys Med NMR*, 1984; 16 : 145 - 155.

Appendix 7

Protocol TB01-35686 (US version)

IV / 3291

TRITON BIOSCIENCES INC.

CLINICAL STUDY PROTOCOL

April 8, 1988
Amendment I October 7, 1988
Amendment II May 24, 1990

BB-IND NUMBER:	1846
STUDY NUMBER:	TB01-35686
DRUG:	Recombinant Beta Interferon, Human IFN-β_{ser} (Betaseron)
DOSE FORM:	Subcutaneous
STUDY TITLE:	Double-Blind Placebo-Controlled Phase Study to Evaluate the Safety and Efficacy of Betaseron Given Subcutaneously in Patients with Relapsing-Remitting Multiple Sclerosis
INVESTIGATIONAL SITE:	MULTICENTER
PROJECT PHYSICIAN:	Louis Randall Bucalo, M.D.<br>Triton Biosciences Inc.<br>1501 Harbor Bay Parkway<br>Alameda, CA 94501<br>(415) 769-5358

IV / 3292

TABLE OF CONTENTS

PAGE

1. TITLE 1
2. OBJECTIVES 1
3. STUDY DESIGN AND DURATION 1
4. BACKGROUND AND RATIONALE 2
5. SELECTION OF PATIENTS 12
   - 5.1 Inclusion Criteria 13
   - 5.2 Exclusion Criteria 14
   - 5.3 Withdrawal Criteria 16
6. CONDUCT OF STUDY 18
   - 6.1 Schedule of Activities and Evaluations 18
   - 6.2 Screening Visit 18
   - 6.3 Randomization 20
   - 6.4 Dose Regimen 21
   - 6.5 Treatment Visits 21
   - 6.6 Termination and Follow-up Visits 31
   - 6.7 Procedure for Treatment by the Patient 36
   - 6.8 Concomitant Therapy 37
   - 6.9 Evaluation of Safety 38
   - 6.10 Dosing Modification in Event of Toxicity 39
7. WITHDRAWAL OF PATIENTS FROM THE STUDY 40
8. DRUG SUPPLY 41
   - 8.1 Supply and Formulation 41
   - 8.2 Reconstitution 41
   - 8.3 Labelling 42
   - 8.4 Storage 42
   - 8.5 Dose Administration 43
   - 8.6 Drug Accountability 43

IV / 3293

9. LABORATORY PROCEDURES 45
   - 9.1 Urinalysis 45
   - 9.2 Hematologic Tests 45
   - 9.3 Serum Chemistries 46
   - 9.4 Lipid Profile 46
   - 9.5 Endocrinological Tests 46
   - 9.6 Serum for Antibody Response To Interferon and Diagnostics Testing 46
10. STATISTICS 47
   - 10.1 Baseline Comparability 47
   - 10.2 Safety Evaluations 47
   - 10.3 Efficacy Evaluation 48
   - 10.4 Statistical Methodology 48
   - 10.5 Scheduled Efficacy Analyses 50
   - 10.6 Maintenance of the Study Blind 52
11. ETHICAL AND REGULATORY CONSIDERATIONS 53
   - 11.1 Case Report Forms 53
   - 11.2 Adverse Event Reporting 53
12. REFERENCES 55

APPENDIX I Functional Capacity and Exercise Tolerance of Cardiac Patients........ 61
APPENDIX II Clinical Toxicity Scale........ 62
APPENDIX III Schedule of Activities........ 65
APPENDIX IV Visual Evoked Potential Protocol for Multiple Sclerosis Therapeutic Trials... 66
APPENDIX V Functional Neurologic Status Evaluation... 69
APPENDIX VI Kurtzke Disability Status Scale in Multiple Sclerosis........ 72
APPENDIX VII Scripps Scale........ 74
APPENDIX VIII Procedure for Collection and Shipment of Serum Specimens for Measurement of Antibody Response to Interferon and Serum for Diagnostics Testing........ 75

IV / 3294

APPENDIX IX Patient Instructions for Subcutaneous Self Administration of Study Drug (Beta Interferon/Placebo)........ 80
APPENDIX X MRI Scanning Protocol........ 83
APPENDIX XI Assessment of Reproductive Function in Women with MS that are Treated with Betaseron (Human Recombinant Beta Interferon)..... 90

IV / 3295

1. TITLE Double-Blind Placebo-Controlled Phase III Study to Evaluate the Safety and Efficacy of Betaseron® Given Subcutaneously in Patients with Relapsing-Remitting Multiple Sclerosis

2. OBJECTIVES

2.1 To compare the safety and tolerance of Betaseron at 45 X 10^6 IU, Betaseron at 9 x 10^6 IU, and placebo when given in an alternate day regimen by subcutaneous injections for 104 weeks to patients with relapsing-remitting multiple sclerosis (MS).

2.2 To compare the therapeutic efficacy of 45 x 10^6 IU of Betaseron, 9 x 10^6 IU of Betaseron, and placebo when administered in the stated regimen to patients with MS.

3. STUDY DESIGN AND DURATION

3.1 This double-blind, placebo-controlled Phase III study evaluates the safety and efficacy of Betaseron in the treatment of patients with relapsing-remitting MS. A total of 330 patients (between two studies; TB01-35686, TB01-35886) will be randomly assigned to receive either 45 x 10^6 IU of Betaseron, 9 x 10^6 IU of Betaseron, or placebo. A subcutaneous injection of the assigned medication will be given on alternate days for 104 weeks.

IV / 3296

3.2 Patients may continue on therapy during the study as long as none of the Withdrawal Criteria (Section 5.3) apply.

3.3 This study protocol is identical to Triton study protocol TB01-35886. Protocol TB01-35686 governs sites in the United States and protocol TB01-35886 governs sites in Canada. Enrollment will continue under both protocols until a total of three hundred and thirty patients have been enrolled. The data from both protocols will be pooled and analyzed as one multicenter study.

4. BACKGROUND AND RATIONALE

Multiple sclerosis is a chronic demyelinating disease of the central nervous system that afflicts 250,000 people in the United States. The demyelinated lesions characteristic of MS are found throughout the central nervous system, although they occur most commonly in the optic nerves, spinal cord, medulla and periventricular white matter of the cerebral hemispheres. Plaques also may involve the cerebral cortex and deep cerebral gray matter.

Many investigators have presented evidence, albeit inconclusive, that a viral infection is responsible for MS.[1] Most recently, a retrovirus related to HTLV I has been cited

IV / 3297

as suspect.[2] Levels of specific antibody to measles virus are significantly higher in the serum and CSF of MS patients as compared to controls.[3]

Interferons are biologically active molecules which generate resistance to acute and persistent virus infection by inducing an antiviral state at the cellular level and by modulating immune responses in the whole organism.[4] Peripheral blood leukocytes from MS patients have a suboptimal response to interferon inducers and an impaired ability to produce interferon in response to virus infection.[3,5] This impaired ability to produce interferon may contribute to persistence of the virus in the CSF.

The activity of natural killer (NK) cells, which have the ability to recognize and destroy virus-infected cells, has been reported to be impaired in MS patients.[6] Studies have indicated that NK cell activity is low in relapsing-remitting MS patients in remission and may remain low for many months after an exacerbation.[7] NK cell activity may also improve in MS patients treated with interferon.[8] Since NK cells are important to host defense against viral infections, an NK cell defect may represent a crucial immunologic defect in MS. NK cell dysfunction in MS remains an area of controversy and of investigation.

Early clinical trials with natural alpha and natural beta interferons yielded encouraging, though inconclusive results.[9,10] Jacobs et al have confirmed results with natural interferon beta in a larger study that showed a significant

IV / 3298

reduction in exacerbations in patients who received intrathecal injections.[11] In contrast, therapy with gamma interferon appears to provoke exacerbations.[12] Interferon gamma, by induction of Ia determinants on endothelial cells and astrocytes, [13,14] may be implicated in the pathogenesis of experimental allergic encephalomyelitis, an animal model of MS.[15] The observed prominence of Ia+ endothelial cells, macrophages, and astrocytes in MS plaques[16,17] raises the possibility that interferon gamma is important in the pathogenesis of disease activity. Interferon beta has been reported to counteract the Ia antigen-inducing effect of interferon gamma, adding to its potential therapeutic value in MS.[18,19]

The clinical trial described in this protocol will evaluate subcutaneous therapy with Betaseron, a recombinant beta interferon, in the treatment of MS at two different dosage levels, 9 x 10^6 IU and 45 x 10^6 IU. The doses were selected on the basis of preliminary results from an ongoing pilot study. The pilot trial demonstrated that MS patients tolerate Betaseron therapy well at the 45 x 10^6 IU dose level and at lower dose levels, and that exacerbations were not provoked by Betaseron therapy at any of the dose levels studied.

A total of 30 patients are enrolled in the pilot study, which began in June of 1986. The study uses a double-blind, placebo-controlled design with five parallel treatment groups. Treatment consists of three-times-weekly subcutaneous injections with either placebo or Betaseron. During the first 24 weeks, patients who received active drug were treated at

one of four dose levels (4.5, 22.5, 45, or 90 x 10^6 IU). From the 25th week on, all patients on active drug have been treated at 45 x 10^6 IU.

An interim analysis was completed of the data for the first 24 weeks. In the 30 patients, 15 exacerbations were reported. A 16th exacerbation that began prior to study entry and was concealed by the patient was not included. Distribution of the 15 evaluable exacerbations by dose is as follows: placebo group, five; 4.5 x 10^6 IU group, three; 22.5 x 10^6 IU group, five; 45 x 10^6 IU group, two; and 90 x 10^6 IU, zero. A subsequent analysis at 36 weeks showed an additional 2 exacerbations: 1 in the placebo group and 1 in a patient receiving 45 million units of Betaseron.

Betaseron therapy was well tolerated, especially at lower dosages. Some patients experienced flu-like symptoms commonly associated with interferon therapy. The only adverse experiences that correlated statistically with dose were erythema at the injection site (73%) and pain at the injection site (56%).

Clinical laboratory values remained in the normal range or were not clinically significant in 83% or more of the total group of 30 patients for all parameters during the first 36 weeks of therapy. No grade III or IV values were reported in either the placebo or active-drug groups for this period.

Although the pilot study provides the only data for evaluating Betaseron therapy in MS patients, tolerance to Betaseron has also been evaluated extensively in phase I and II

antineoplastic and antiviral clinical trials.[20] To date, more than 1,100 subjects have participated in studies evaluating Betaseron therapy. This interferon has been given intravenously, intramuscularly, subcutaneously, intracerebrally, topically, intranasally, and intravesically. Overall, clinical studies to date demonstrate that Betaseron is well tolerated.

Doses in Betaseron trials have ranged from 0.1 to 990 x 10^6 IU, with maximum tolerated doses (MTD) of as high as 720 x 10^6 IU. More than 135 patients have been treated at doses of 100 x 10^6 IU or greater. Therapy over the long term is well tolerated. At least 25 patients have been treated for a year or longer, most at doses of 90 x 10^6 IU or greater.

Safety and efficacy have been studied in a wide range of neoplastic disorders.[21-39] Tumor regression has been seen in patients with renal cell carcinoma, glioma, melanoma, bladder cancer, AIDS-related Kaposi's sarcoma, hairy cell leukemia, lung cancer, nasopharyngeal cancer, and colorectal carcinoma. Ongoing phase I and II trials are evaluating activity in a number of cancers. Both escalating- and fixed-dose regimens are being used, with doses ranging from 22.5 to 990 x 10^6 IU. Betaseron is being given intravenously, subcutaneously, intracerebrally, and intravesically.

The antiviral properties of Betaseron have been evaluated in rhinovirus infection, chronic hepatitis B, condyloma acuminata, and herpes labialis.[40-46] Activity in rhinovirus and hepatitis B infection has been demonstrated, but analysis

of data has not been completed for condyloma and herpes labialis studies. In ongoing phase I and II antiviral studies, Betaseron is being given by the intravenous, subcutaneous, or intranasal route. Doses range from 12 to 90 x 10^6 IU.

In subjects treated parenterally with Betaseron, the most frequently reported adverse experiences are the flu-like symptoms commonly associated with interferon therapy. They include fatigue, chills, fever, headache, myalgia, and arthralgia. These symptoms are seldom dose limiting, are generally well controlled with acetaminophen therapy, and usually abate with continuing therapy.

Anorexia, nausea, vomiting, diarrhea, dizziness, paresthesia, diaphoresis, and malaise are less common than the flu-like symptoms, and are rarely dose limiting. In subcutaneously treated subjects, injection site reactions are most commonly mild, and range from pain, erythema, and inflammation to infrequent ulceration, cellulitis, and necrosis. However, necrosis is less common in the multiple sclerosis population than in subjects with HIV infection. Local reactions are seldom reported in subjects treated by routes other than subcutaneous.

Cardiac problems observed have included cardiomyopathy, arrhythmias, myocardial infarction, and congestive heart failure.

Hypotension, transient visual disturbances, and confusion may occur but are rare. Seizures have occurred rarely. Dementia has been reported in subjects with brain tumors.

Laboratory abnormalities reported in parenterally treated subjects include anemia, neutropenia, leukopenia, thrombocytopenia, proteinuria, elevation of liver enzymes, hypertriglyceridemia, and rarely, nonfasting hyperglycemia, usually in subjects with known underlying risk factors.

Neutralizing-antibody activity to Betaseron has been detected in some patients treated with Betaseron. Data are still preliminary but indicate that the incidence is less than 10%.[20] Currently, no correlation between antibody formation and either the safety or efficacy of Betaseron therapy has been demonstrated.

Results of completed antineoplastic trials with Betaseron are summarized in the following sections.

Maximum tolerated single dose (MTSD) study. The initial phase I trial was designed to define intramuscular and intravenous MTSD in advanced cancer patients.[21,22] Intramuscular MTSDs ranged from 60 to 300 x 10^6 IU, while intravenous MTSDs ranged from 10 to 400 x 10^6 IU. No responses were seen, but disease stabilized for 3 months or longer in three of 14 patients with measurable disease at entry. In one patient with non-Hodgkin's lymphoma, disease remained stable for more than 18 months and therapy was tolerated for over 2 years at dose levels of 60 or 100 x 10^6 IU.

Completed escalating-dose intravenous studies. Three intravenous escalating-dose regimens have been completed under National Cancer Institute sponsorship.[23-25] Two were phase I studies in advanced cancer patients; the other was a phase I/II trial in patients with renal cell carcinoma. Doses ranged from 0.006 to 600 x 10^6 IU/m². Betaseron was given twice weekly, by 4-hour intravenous infusion in two studies and by intravenous push in the other.

No conventional MTD was defined in either phase I study despite escalation to as high as 400 x 10^6 IU/m² in one trial and 500 x 10^6 IU/m² in the other.[23,24] In the phase II study, an initial MTD of 150 x 10^6 IU/m² was defined, but 72% of patients were escalated to this MTD and tolerated twice weekly dosing at this level or higher.[25] Twenty-two percent of patients tolerated dosing in the 450 to 600 x 10^6 IU/m² range.

In one of the phase I trials, two minor responses were recorded in four evaluable melanoma patients.[23] Total enrollment was 14. In the phase II renal cell carcinoma trial, two partial responses, one minor response, and five patients with stable disease were reported in 15 evaluable patients.[25] No responses were documented in the other phase I trial.

Only two of the escalating-dose studies being conducted by Triton have been completed. One compared Betaseron and native interferon beta therapy in patients with advanced cancer.[26]

The interferons were given twice weekly and the dose escalated weekly through four levels (1, 10, 30, and 60 x 10^6 IU). No responses were seen.

The other completed study evaluated therapy in melanoma patients.[27] The dose escalated weekly through five levels (30, 60, 90, 180, and 270 x 10^6 IU). At lower doses (30 and 60 x 10^6 IU), Betaseron was given once daily for 5 days each week. When higher levels were achieved, the dose was given twice weekly. All patients treated were terminated because of progressive disease. No MTD was defined.

Completed fixed-dose intravenous studies. In all other completed phase I and II intravenous studies, individual patients were treated at fixed doses, although the dose escalated between groups of patients in some MTD-defining trials.[28-35] Doses ranged from 3 to 300 x 10^6 IU and a variety of schedules were evaluated, including three times weekly dosing and repetitive daily dosing with an extended washout (e.g., 5 days weekly for 2 weeks with a 2-week washout).

In most phase I studies, therapy was evaluated in patients with advanced cancer. Phase I/II and II studies included patients with breast cancer, colorectal cancer, hairy cell leukemia, head and neck neoplasms, lung cancer, lymphoma, melanoma, myeloma, and renal cell carcinoma. Two patients from early phase I studies continue on therapy more than 2 1/2 years after starting treatment. Both are receiving 30 x 10^6

IU on a M-F schedule followed by a 9-day washout. One has renal cell carcinoma that has stabilized; the other has hairy cell leukemia with a documented partial response.

Preliminary results in an ongoing trial of intravenous therapy in patients with recurrent malignant glioma have been reported.[35] Eight patients had been enrolled at the time of the report. One patient had tumor regression and another had stable disease for three months. These patients were treated at a fixed dose of 90 x 10^6 IU, given three times weekly. More patients are to be enrolled and a dose-escalation schema will be used to define maximum tolerated dose.

Completed subcutaneous studies. Although a number of studies using subcutaneous administration are underway, only one study has been completed. Patients in this trial were treated at fixed doses of either 10 or 100 x 10^6 IU without toxicity.[36,37] During the initiation period, Betaseron was given daily for 42 days; a MWF dosing schedule was used for maintenance therapy. A partial response was seen in one patient with melanoma. He is disease free after surgical excision of one lesion early in maintenance therapy and continues on study at a dose of 100 x 10^6 IU after nearly 3 years.

Completed intramuscular study. The intramuscular route was evaluated in a phase I trial of fixed dosing at 10 or 100 x 10^6 IU on a M-F schedule.[38] A partial response was documented in three patients, one with AIDS-related Kaposi's sarcoma, one with renal cell carcinoma, and one with melanoma. The

melanoma patient with a partial response and another renal cell patient with stable disease received more than a year of therapy at 10 x 10^6 IU on a MWF maintenance schedule.

Intravesical and intracerebral studies. Studies evaluating dosing by these routes are ongoing. Betaseron is being given by intravesical infusion in patients with bladder cancer, and by intracerebral infusion in glioma patients. Preliminary results reported for one intracerebral study show an enrollment of 15, with three patients terminated because of infection and eight terminated because of progressive disease.[39] Two of the seven patients who continue on therapy have disease that has stabilized for three months or longer.

5. SELECTION OF PATIENTS

A total of three hundred and thirty patients will be enrolled into the two parallel studies, TB01-35686 and TB01-35886. Patient enrollment will continue at all active sites until study enrollment is complete. Once study enrollment completion is assured, the project physician will notify all sites to stop enrollment. As of the enrollment stop date, only patients who have signed an informed consent and for whom screening procedures have begun may be enrolled. These patients must begin treatment within 21 days of the enrollment stop date.

To be eligible for enrollment, patients must meet all inclusion and exclusion criteria listed in Sections 5.1 and 5.2.

5.1 Inclusion Criteria

5.1.1 Patients must have clinically definite MS or laboratory-supported definite MS as defined by Poser criteria for not less than 1 year.

5.1.2 Patients must have a history of clearly identified relapses and remissions.

Relapse shall be defined as the appearance of a new neurologic abnormality or the reappearance of a neurologic abnormality at any time after the initial attack. This change in clinical state shall not be considered a relapse if it does not last at least 24 hours, and if it is not immediately preceded by a stable or improving neurologic state in the 30 days before deterioration.

A remission is defined as the complete disappearance or significant decrease in severity followed by stability for at least 1 month of a neurologic abnormality that had lasted for at least 24 hours.

5.1.3 Patients will have had at least two relapses in the 2 years before entry into the study.

5.1.4 Patients will have stable disease for at least one month at time of the screen and baseline evaluations.

5.1.5 Patients will be between 18 and 50 years of age inclusive, and be considered a legal adult in the state or province in which the study is conducted.

5.1.6 Patients must show objective neurologic evidence of disease that reflects predominantly white matter (fiber-tract) damage. Symptoms alone cannot be accepted as diagnostic.

5.1.7 The patient's signs and symptoms cannot be better explained by another disease process.

5.1.8 Patients must have a friend or family member who can assist in carrying out the requirements of this protocol should progressive disability impair ability to comply.

5.1.9 Patient must be ambulatory, grade 0 through 5.5 as defined in the Kurtzke Disability Status Scale (Appendix VI).

5.1.10 Patients must have signed an approved informed consent prior to initiating screening procedures.

5.2 Exclusion Criteria
Any of the following conditions will exclude the patient from the study:

5.2.1 Pregnancy or lactation. Women no longer capable of childbearing (i.e., post-hysterectomy, tubal ligation or 1 year post-menopausal) are eligible for study enrollment. Women of childbearing potential must be using and agree to continue to use an IUD, birth control pills, or adequate barrier contraception.

5.2.2 Medical or psychiatric conditions that compromise the patient's ability to give informed consent or complete the study.

5.2.3 New York Heart Classification III or IV (Appendix I).

5.2.4 Uncontrolled angina pectoris.

5.2.5 Evidence of clinically significant, uncontrolled cardiac dysrhythmias.

5.2.6 Prior therapy with alpha, beta or gamma interferon.

5.2.7 Need for concomitant therapy with corticosteroids or ACTH or therapy with these agents within 30 days prior to entry into the study.

5.2.8 Previous immunosuppressive therapy with cytotoxic chemotherapy.

5.2.9 Observation by the principal investigators or their staff at participating institutions cannot be assured for the duration of the study.

5.2.10 Upper extremity disability that prevents self-administration of subcutaneous medication, and the absence of a friend or family member who can reliably administer the subcutaneous injections.

5.2.11 Patients who are in relapse or who have entered a progressive phase of their illness at time of entry into the study.

5.2.12 Allergy to acetaminophen.

5.2.13 Need for chronic concurrent therapy with aspirin or non-steroidal anti-inflammatory drugs.

5.2.14 Use of other investigational drugs in the 30 days preceding study entry.

5.3 Withdrawal Criteria
The patient shall be removed from the study and administration of study medication terminated if any of the following conditions occur:

5.3.1 Interruption of scheduled dosing for greater than 2 weeks, unless as a result of resolution of investigational drug toxicity or exacerbation.

IV / 3311

5.3.2 Intolerable adverse effects.

5.3.3 Patient decision to discontinue treatment because of adverse effects or for any other reason.

5.3.4 Second occurrence of grade 3 or 4 toxicity (Appendix II).

5.3.5 Loss of patient to follow-up.

5.3.6 Pregnancy.

5.3.7 Poor compliance or unauthorized use of investigational agent.

5.3.8 Use of other investigational or experimental therapies for MS or use of steroids other than allowed by protocol. **All types of corticosteroids in clinical use for the treatment of acute exacerbations may be used at the investigator's discretion for not more than 28 days per exacerbation. No more than 3 courses of corticosteroid therapy for treatment of exacerbations will be permitted in any 12 month period.**

5.3.9 A phase of increasing disability that progresses unremittingly for 6 consecutive months.

5.3.10 Need for chronic concurrent therapy with aspirin or non-steroidal anti-inflammatory drugs.

IV / 3312

5.3.11 Termination of study.

5.3.12 Patient has been unblinded.

6. CONDUCT OF STUDY

6.1 Schedule of Activities and Evaluations (Appendix III)
All procedures, tests, and examinations required for each visit are listed. Additional evaluations and testing may be done at the discretion of the investigator.

Endocrinologic studies will be conducted on female patients at designated sites (Appendix XI).

An additional subset of patients at one site will have MRI's on an every six week basis.

6.2 Screening Visit

6.2.1 Patient screening may occur anytime within 21 days prior to the initial dose, as long as all required procedures are completed and all inclusion and exclusion criteria are met prior to the initial dose. Results of all screening evaluations which assure that all inclusion and exclusion criteria have been satisfied must be reviewed prior to the first dose of Betaseron.

IV / 3313

Before screening begins, the patient must be thoroughly informed about all aspects of the study, including the study visit and activities schedule. Informed consent must be signed before screening procedures begin.

A checklist for the screening visit follows:
- Eligibility checklist
- Signed informed consent
- Complete history of the neurologic illness, including:
  - duration of disease
  - number of exacerbations since onset
  - number of exacerbations in past 2 years
  - history of progression
  - all attempts at therapeutic intervention
  - age at onset
  - medications given for relief of symptoms
- Complete medical history (including but not limited to atopy, asthma, known allergies, viral and other infectious diseases, autoimmune disorders, cardiovascular disease and gastrointestinal disorders)
- Complete physical and neurologic examination, including:
  - Functional Neurologic Status Evaluation (Appendix V)
  - Kurtzke Disability Status Scale (Appendix VI)

IV / 3314

  - completion of Scripps Neurologic Rating Scale worksheet (Appendix VII)
  - height and weight
  - vital signs (oral temperature, pulse, respiratory rate, and blood pressure)
  - chest x-ray
  - EKG
  - menstrual cycle history
- Specialized testing, including:
  - visual acuity
  - visual evoked potential (Appendix IV)
  - MRI where available; must be done as per Appendix X
- Laboratory studies, including:
  - urinalysis (Section 9.1)
  - hematology (Section 9.2)
  - serum chemistries (Section 9.3)
  - negative serum pregnancy test (females)
- Concomitant therapy

6.3 Randomization
The 330 patients enrolled into the two parallel studies, TB01-35686 and TB01-35886, will be divided randomly into three treatment groups of 110 patients each. Each group will receive either 45 X 10^6 IU of Betaseron, 9 x 10^6 IU of Betaseron, or placebo. After meeting the criteria for admission, each patient will be assigned a patient number. The pre-determined sponsor-prepared randomization schedule will determine medication

assignment. The double-blinded supplies will be packaged so that each patient receives the appropriate treatment.

6.4 Dose Regimen
Starting on day 1 patients will receive 0.5 ml of either the low-dose preparation Betaseron (4.5 x 10^6 IU), 0.5 ml of the higher-dose preparation Betaseron (22.5 x 10^6 IU), or 0.5 ml of placebo subcutaneously, once a day, on alternate days for 2 weeks (7 doses). A new vial must be utilized for each dose. The used vial containing a residual 0-5 ml, must be returned. Starting on day 15 dosing will be increased to 1.0 ml of the low-dose preparation of Betaseron (9 x 10^6 IU), 1 ml of the high-dose preparation (45 x 10^6 IU), or 1 ml of placebo. The study will continue for 104 weeks. All used vials must be returned.

6.5 Treatment Visits
On the first two treatment days, patients and their support person(s) will report to the study center to be instructed in self-administration of the study medication and for the patient to receive the subcutaneous injections. On the third treatment day if the injection technique has been mastered, the patient may self-administer their injection at home. On day 15 (week 3) the patients will report to the study center for a brief interview and examination, a single 1 ml subcutaneous injection, and instruction on how to

administer the injections to be given for the remainder of the study (102 weeks). Patients will be given all necessary supplies and a detailed instruction sheet, which will be reviewed with the patient and the support person at the time of the visit. In addition, patients will be instructed to contact the study center if questions or problems arise. Patient diaries will be provided for the patients to report adverse experiences, concomitant therapy and drug administration.

Patients will return to the clinic at weeks 5 and 7 for evaluation. Thereafter, they will return for evaluation every 6 weeks for 5 visits (weeks 13, 19, 25, 31, 37) and then at 12 week intervals until study completion.

Checklists for the treatment visits follow. All procedures, tests, and examinations listed are the minimum required at each visit. Procedures listed before the SQ dose are to be done prior to dosing. At the investigator's discretion, additional procedures or more thorough physical examinations may be done to ensure the patient's well being.

Two separate blinded physicians will be responsible for the evaluation of each patient. The first physician will conduct the neurologic examination and record the neurologic symptoms and findings as required. The second physician will be responsible for evaluating the patient's subjective findings, including symptoms either

related to or unrelated to MS or investigational agent therapy. The second physician will also be responsible for managing the patient's overall medical care.

A nurse or data manager may assist with the evaluation of possible adverse experiences. Patients will be asked in a consistent manner about presence or absence of symptoms known to be associated with interferon therapy such as fever or malaise.

6.5.1 Week 1, Day 1 Baseline
- Brief physical examination to update the findings of the screening period examination
- Baseline symptoms
- Intercurrent medical events
- Weight
- Urinalysis
- Hematology
- Serum chemistries
- Lipid profile
- Serum for antibody response to interferon and diagnostics testing
- 1 hour vital signs (predose and at 30 and 60 minutes after dosing)
- First SQ dose of investigational agent (0.5 ml)
- Adverse experience evaluation
- Concomitant therapy
- Drug accountability
- Patient instruction

6.5.2 Week 1, Day 3
- Second SQ dose investigational agent (0.5 ml)
- Adverse experience evaluation
- Concomitant therapy
- Drug accountability
- Intercurrent medical event
- Patient instruction

6.5.3 Week 1, Day 5; Week 2 (Doses 3-7)
Patients will self administer investigational agent (i.e. 0.5 ml) on alternate day schedule in their home. Patients will have been instructed to contact the study center if questions or problems arise. Patient diaries will be provided for patients to report the following:
- Adverse experiences
- Concomitant therapy
- Drug accountability
- Intercurrent medical event

6.5.4 Week 3, Day 15
- Patient visit to clinic for dosing instructions
- Brief physical exam
- SQ dose investigational agent (1.0 ml)
- Adverse experience evaluation
- Concomitant therapy
- Drug accountability
- Intercurrent medical events
- Patient instruction

6.5.5 Self administration
- Patients will self administer investigational agent (i.e. 1.0 ml) in their home on an alternate day schedule with visits to the clinic as specified. Patient diaries will be provided for patients to report the following:
- Adverse experiences
- Concomitant therapy
- Drug accountability with study completion
- Intercurrent medical events

6.5.6 Week 5
- Brief physical exam
- Hematology
- Serum chemistries
- Urinalysis
- Adverse experience evaluation
- Concomitant therapy
- Drug accountability
- Intercurrent medical events

6.5.7 Week 7
- Brief physical examination
- Weight
- Vital Signs
- Neurologic assessment using Functional Neurologic Status Evaluation (Appendix V), Kurtzke Disability Status Scale (Appendix VI), and Scripps Neurologic Rating Scale (Appendix VII)

- Urinalysis
- Hematology
- Serum chemistries
- Serum for antibody response to interferon and diagnostics testing (Appendix VIII)
- Adverse experience evaluation
- Concomitant therapy
- Drug accountability
- Intercurrent medical events

6.5.8 Week 13 (and weeks 25, 37, 61, 73, 85, 97)
- Brief physical examination
- Menstrual Cycle information, females (only at designated sites)
- Weight
- Vital Signs
- Neurologic assessment using:
  • Functional Neurologic Status Evaluation (Appendix V)
  • Kurtzke Disability Status Scale (Appendix VI)
  • Scripps Neurologic Rating Scale (Appendix VII)
- Urinalysis
- Hematology
- Serum chemistries
- Lipid profile (also done at weeks 25, 49, 73 and termination)
- Serum for antibody response to interferon and diagnostics testing (Appendix VIII)

- Adverse experience evaluation
- Concomitant therapy
- Drug accountability
- Intercurrent medical events

6.5.9 Week 19 (and Week 31)
- Brief physical exam
- Weight
- Vital Signs
- Urinalysis
- Hematology
- Serum chemistries
- Serum for antibody response to interferon and diagnostics testing (Appendix VIII)
- Adverse experience evaluation
- Concomitant therapy
- Drug accountability
- Intercurrent medical events

6.5.10 Week 49
- Complete physical exam
- Menstrual cycle information - females (only at designated sites)
- Weight
- Vital signs
- Neurological assessment using:
  • Functional Neurologic Status Evaluation (Appendix V)
  • Kurtzke Disability Status Scale (Appendix VI)

  • Scripps Neurologic Rating Scale (Appendix VII)
- MRI where available, must be done as per Appendix X
- Urinalysis
- Hematology
- Serum Chemistries
- Lipid profile
- Serum for antibody response to interferon and diagnostics testing (Appendix III)
- SQ Dose of investigational agent (1.0 ml)
- Adverse experience evaluation
- Concomitant therapy
- Drug accountability
- Intercurrent medical events

6.5.11 Unscheduled Visit
An unscheduled visit can occur at anytime during the study. The date of and reason for the visit and any data generated should be recorded on the Unscheduled Visit section of the Case Report Forms. Should the visit be related to an exacerbation, this will be clearly indicated on the Case Report Forms, and a complete neurologic evaluation will be performed and recorded, as in Section 6.5.13 below. Unscheduled visit forms should be placed in chronological order within the case report form binder.

6.5.12 Hospitalization

The investigator may hospitalize any patient in whom symptoms warranting treatment or close monitoring develop. Investigational agent administration should continue unless the clinical investigator recommends that it be stopped. The project manager must be informed when therapy is discontinued.

6.5.13 Exacerbations

Patients will be instructed to contact the study center immediately should any symptoms suggestive of an exacerbation appear. The study center will evaluate these patients within 24 hours. Exacerbations will be classified as mild, moderate or severe based on the change in the Scripps NRS Score (Appendix VII). Mild exacerbations include those with a change of 7 points or less, moderate exacerbations include those with a change of 8-14 NRS points and severe exacerbations include those with a change of 15 NRS points or more. The examining physician will note the anatomic location of the lesion(s) responsible for the new clinical finding(s) (i.e., right or left cerebral hemisphere, optic nerve, brain stem, cerebellum or spinal cord). Neurologic assessment will include Functional Status Evaluation (see Appendix V), Kurtzke Disability Status Scale

(see Appendix VI) and Scripps Neurologic Rating Scale (see Appendix VII). Follow-up visits to monitor the course of the exacerbation will be made at the investigator's discretion.

In addition, when, in the investigators opinion, the exacerbation has begun to remit, the investigator will assess the global severity of the exacerbation at its worst point as either mild, moderate, or severe. This evaluation will be based on the investigator's clinical judgement of the severity of the event at its worst point. This evaluation will occur in addition to the evaluation based on the Scripps Score. This severity designation and the date of the assessment will be entered on the exacerbation (EA) case report form.

The Exacerbation Case Report Forms will be completed and inserted into the Case Report Form notebook at the time of each visit.

A study center nurse or data manager will telephone the patient during the exacerbation at weekly intervals to inquire about the patient's clinical status. The information will be recorded in the Case Report Forms.

Prednisone or ACTH therapy as outlined in Section 5.3.8 may be given at the discretion of the investigator and must be recorded on the Exacerbation Case Report Forms.

6.6 Termination and Follow-up Visits

6.6.1 Termination Visit

A termination visit occurs whenever a patient completes the 104 weeks of treatment or withdraws prematurely (Section 5.3). **In the event that the next Betaseron trial for multiple sclerosis is not enrolling at the time that subjects wishing to participate in the new trial are near study completion (104 weeks), these subjects will be notified by Triton to remain on study (i.e. continue receiving study medication) and delay termination until the new trial begins. In such event, these subjects will have their week 104 visit as scheduled with all procedures as per protocol. However, the TER case report form will not be completed, and the subject will not be terminated at that visit.** In the event of premature termination, the final visit must take place within 2 weeks after the last dose of investigational agent. The MRI is required if the patient has been in the study for greater than 6 months but less than 1 year duration.

- **Termination reason**
- Complete physical examination

- Menstrual cycle information - females (only at designated sites)
- Weight
- Vital signs
- Neurologic assessment using:
  - Functional Neurologic Status Evaluation (Appendix V)
  - Kurtzke Disability Status Scale (Appendix VI)
  - Scripps Neurologic Rating Scale (Appendix VII)
- MRI where available; must be done as per Appendix X
- Visual acuity
- Visual evoked potentials (Appendix IV)
- EKG
- Chest x-ray
- Urinalysis
- Hematology
- Serum chemistries
- Lipid profile
- Serum antibody response to interferon and diagnostics testing response to interferon
- Adverse experience evaluation
- Concomitant therapy
- Drug accountability
- Intercurrent medical events

IV / 332

6.6.2 Subjects, who have completed approximately 104 weeks of drug therapy under this protocol, will participate in the next Betaseron study for multiple sclerosis, and will not require a follow-up visit under this study.

6.6.2.1 For subjects who have prematurely terminated or who terminate at week 104 for any reason (including adverse events), the following activities will be performed approximately every 3 months at each follow-up visit until the subject has reached 104 weeks from the date of their first dose. This follow-up should continue through week 104 and for at least 3 months following termination.

IV / 3328

Additionally, subjects who terminated with adverse events and/or toxicities possibly related to the study medication: (1) should be evaluated within 2 weeks after the last dose of study medication; and, (2) should have a follow-up visit at least every 3 months until resolution of the events/toxicities.

Activities performed at each follow-up visit will include, but are not limited to:
- Vital signs
- Hematology
- Serum chemistries
- Brief Physical Exam
- Neurologic assessment using:
  - Functional Neurologic Status Evaluation (Appendix V)
  - Kurtzke Disability Status Scale (Appendix VI)
  - Scripps Neurological Rating Scale (Appendix VII)
- Serum for antibody response to interferon and diagnostics testing (Appendix VIII)

IV / 332

6.6.2.2 Should any symptoms suggestive of an exacerbation appear at any time during the follow-up period, subjects will be asked to contact the study center immediately. The study center will evaluate these subjects as soon as possible, but no later than 72 hours after contact. Exacerbations will be classified as mild, moderate or severe based on the change in the Scripps NRS Score (Appendix VII). Mild exacerbations include those with a change of 7 points or less, moderate exacerbations include those with a change of 8-14 NRS points, and severe exacerbations include those with a change of 15 NRS points or more. The examining physician will note the anatomic location of the lesion(s) responsible for the new clinical finding(s) (i.e., right or left cerebral hemisphere, optic nerve, brain stem, cerebellum or spinal cord). Neurologic assessment will include Functional Status Evaluation (see Appendix V), Kurtzke Disability Status Scale (see Appendix VI) and Scripps Neurologic Rating Scale (see Appendix VII). Follow-up visits to monitor the course of the exacerbation will be made at the investigator's discretion.

IV / 3330

In addition, when, in the investigators opinion, the exacerbation has begun to remit, the investigator will assess the global severity of the exacerbation at its worst point as either mild, moderate, or severe. This evaluation will be based on the investigator's clinical judgement of the severity of the event at its worst point. This evaluation will occur in addition to the evaluation based on the Scripps Score. This severity designation and the date of the assessment will be entered on the case report form.

The follow-up Exacerbation Case Report Forms will be completed and inserted into the Case Report Form notebook at the time of each visit.

6.7 Procedure for Treatment by the Patient

The investigational agent must be transported to the patient's home in the insulated containers provided by Triton (Section 8.1). The patient must provide refrigerated storage (2° to 8° C). When the patient returns to the primary research center for the next scheduled visit, he/she must return the insulated container with the used (and/or unused) investigational agent vials inside.

The patient must also be given one of the instruction packets provided by Triton. These packets will contain reconstitution and dosing instructions.

A patient diary will be provided. The patient will note the date of each injection, any adverse experiences or intercurrent medical events, and any changes in concomitant medication in the diary. The completed diary is to be returned to the primary research site at the next scheduled visit. The data manager or investigator will review the diary entries and transcribe all pertinent data onto the case report forms. The diary will then be maintained as part of the study documentation on site and be available for review by the study monitor.

6.8 Concomitant Therapy

6.8.1 Acetaminophen may be given concomitantly with investigational agent for relief of fever and/or myalgias. All such use must be documented on the appropriate case report form.

6.8.2 While active in this study, patients may not receive concomitant therapy with any of the following:
- Chemotherapeutic agents
- Other investigational drugs
- Chronic steroid therapy; ACTH and prednisone is allowed as outlined in Section 5.3.8.

6.9 Evaluation of Safety
Special attention will be given to detecting known adverse effects of Betaseron therapy. These include fever, chills, fatigue, headache, malaise, anorexia, nausea, vomiting, diarrhea, dizziness, paresthesias, diaphoresis, myalgias and arthralgias. Injection site reactions are most commonly mild, and range from pain, erythema and inflammation to infrequent ulceration, cellulitis and necrosis. However, necrosis is less common in the multiple sclerosis population then in subjects with HIV infection. Cardiac problems have included cardiomyopathy, arrhythmias, myocardial infarction, and congestive heart failure.

Hypotension, transient visual disturbances, and confusion may occur but are rare. Seizures have occurred rarely. Dementia has been reported in subjects with brain tumors.

Laboratory abnormalities include anemia, neutropenia, leukopenia, thrombocytopenia, proteinuria, elevation of liver enzymes, hypertriglyceridemia, and rarely, nonfasting hyperglycemia.

6.9.1 Systemic Reactions
- Objective (abnormal findings)
Abnormal vital signs, other signs of systemic effects, and clinically significant abnormal

laboratory test results will be recorded, and the patient followed until findings or values return to baseline.

- Subjective (symptoms)
Any symptoms occurring during the dosing period will be noted. Date of onset, duration, degree of severity, and any action taken with outcome will be recorded.

6.9.2 Local Reaction
Signs or symptoms of local irritation at the injection site will be recorded, along with date of onset, duration, degree of severity, and probable relationship to the study medication.

6.10 Dosing Modification in Event of Toxicity
In the event of grade 3 or 4 toxicity (as defined in the Clinical Scale for Interferon Toxicity - Appendix II) or other severe toxicity, the dosing must stop. When the toxicity level has fallen to grade 2 or less, therapy may be reinstituted at a 50% reduction (i.e. 0.5 ml) for each subsequent dose unless toxicity occurs during doses 1-7 (i.e. in such cases, patient is terminated from study). Dosing will remain at the 50% level unless toxicity increases to grade 3 or 4 again, at which time the patient must be terminated from the study. This study does not allow for re-escalation of dosage following dosage reduction.

NOTE: Whenever a dosage reduction is required, Triton Biosciences must be notified immediately. Contact either the study monitor or project physician.

7. WITHDRAWAL OF PATIENTS FROM THE STUDY (Premature Termination)

A patient will be withdrawn from the study if any of the Withdrawal Criteria (Section 5.3) occur.

Any patient with either an allergic reaction or a severe degree of intolerance to the study medication should be observed as long as necessary by the treating physician.

The day treatment is withdrawn, for whatever reason, will be considered the "day study drug terminated". All examinations, laboratory tests, etc. required at this time are delineated under the Termination Visit on the Conduct of Study (Section 6.6.1). Laboratory tests showing significant changes in results should be repeated until values return to normal or baseline or are commensurate with ongoing disease processes.

Patients removed from the study for toxicity should be evaluated within 2 weeks after the last investigational agent injection and followed until the toxicity has resolved.

8. DRUG SUPPLY

8.1 Supply and Formulation

Supplies of Betaseron and placebo that are identical in appearance and packaging will be provided by Triton in individual patient boxes. Study medications will be shipped in insulated containers to maintain necessary refrigeration and additional containers will be supplied for use when treatments are self-administered by the patient at home.

Each individual patient box will contain 7 vials of sterile lyophilized cake of either 0.06 mg of Betaseron (the low-dose preparation) or 0.30 mg of Betaseron (the higher-dose preparation), or placebo. All vials will be labelled with the study number and the patient number.

Single dose vials of diluent will be provided.

8.2 Reconstitution

To reconstitute the contents of one vial, remove the flip-off plastic cap and swab the target area of the stopper with antiseptic. Aseptically inject 1.2 ml of Sterile Diluent into the vial to dissolve the lyophilized cake. Since the vial contents are under vacuum, the diluent should be directed against the side of the vial to avoid excess foaming. After reconstitution, the vials will provide either .05 mg/ml Betaseron (low-dose preparation) with a nominal potency

of 9 x 10^6 IU/ml, or 0.25 mg/ml Betaseron (higher-dose preparation) with a nominal potency of 45 x 10^6 IU/ml, or placebo.

8.3 Labelling

A double-blind two-piece tear-off label will be attached to each box of 7 study medication vials. The tear-off portion of the label is to be removed and attached to the appropriate Case Report Form at the time the study medication is dispensed. The patient must never have access to the tear-off portion of the label. In an emergency, the clinical investigator may break the double-blind code by scraping the silver coating off the label to reveal the contents of the box of vials. The project monitor must be notified immediately if the code is broken.

8.4 Storage

8.4.1 Lyophilized Powder

Vials must be kept refrigerated at 2° to 8° C (36° to 46° F) in a secure location. Stability studies indicate that lyophilized Betaseron is stable for up to 36 months if kept refrigerated at the recommended temperature.

8.4.2 Reconstituted Solution

Because none of the study formulations contain a preservative, the reconstituted products should be administered immediately or held at 2 to 8° C for not more than 3 hours to ensure sterility.

8.5 Dose Administration

To create a single dose of the investigational agent, withdraw the appropriate amount of solution (Section 8.5.1) from the vial of reconstituted investigational agent. Use a syringe fitted with a 27-gauge needle, 1/2 inch in length. The investigational agent is then given by subcutaneous injection as a bolus.

8.5.1 For doses 1-7, the amount of investigational drug withdrawn is 0.5 ml from one vial (i.e., 4.5 x 10^6 IU Betaseron, 22.5 x 10^6 IU of Betaseron, or placebo).

For doses 8 through study completion, the amount of investigational drug withdrawn is 1.0 ml from one vial (i.e., 9 x 10^6 IU of Betaseron, 45 x 10^6 IU of Betaseron, or placebo).

8.6 Drug Accountability

Each shipment of drug supplies for a study will contain an investigational drug invoice identifying the shipment by patient number and listing the quantity of drug shipped. The investigator or designee must sign the invoice verifying the quantity received, condition in which it was received and then return the original to the study monitor.

The drug supply will be packaged and shipped in containers designed to maintain refrigeration (2° to 8° C). If the drug supply appears to be damaged or

nonrefrigerated on receipt, contact the sponsor immediately and do not use the drugs without the sponsor's authorization. Drug supplies must be kept in a secure, limited-access storage area under recommended storage conditions (Section 8.4). Only authorized persons may have access to the drug. During the course of the study, the following information must be noted when drug supplies are used: the identification code of the patient to whom drug is to be administered, and the date(s) and quantity of drug dispensed. Inventory records must be available for inspection by the study monitor or Regulatory Agencies at any time.

When drug supplies are returned either before or at study completion, the investigator or designee must verify in writing that all used or unused supplies have been returned. No study supplies are to remain with the investigator. If all drug supplies are not returned, the reason must be given and any attempts to recover them documented.

The investigator must retain a copy of all inventory record sheets and the return statement. The original inventory sheets are to be included in the return shipment of drug supplies. All supplies and accountability documentation are to be returned to:

IV / 3339

Paul Romig
Director, Development & Manufacturing
Triton Biosciences Inc.
1501 Harbor Bay Parkway
Alameda, CA 94501

9. LABORATORY PROCEDURES

Blood samples and urine specimens must be obtained at the appropriate visits, prior to dosing. Nonfasting samples should be obtained. If results are clinically significant, follow-up fasting tests should be done.

9.1 Urinalysis
- Protein
- pH
- Specific gravity
- Sugar
- Ketones
- Microscopic examination of sediment

9.2 Hematologic Tests
- WBC
- Differential count
- Platelet count (not estimate)
- Hemoglobin
- Hematocrit

IV / 3340

9.3 Serum Chemistries
- BUN
- Creatinine
- Aspartate aminotransferase (AST) or SGOT
- SGPT
- Alkaline phosphatase
- Bilirubin
- Phosphorous
- Total serum protein
- Albumin
- Calcium
- Uric acid
- Glucose
- Serum electrolytes (Na+, K+, Cl-, Bicarbonate)
- LDH

9.4 Lipid Profile
- Cholesterol
- Triglycerides
- LDL
- HDL

9.5 Endocrinological Tests To be Done at Designated Sites (Appendix XI)

9.6 Serum for Antibody Response To Interferon and Diagnostics Testing
See Appendix VIII for instructions.

IV / 3341

10. STATISTICS

Studies TB01-35686 and TB01-35886 are identical parallel studies. Protocol TB01-35686 governs sites in the United States and protocol TB01-35886 governs sites in Canada. The data from both protocols will be pooled and analyzed as one multicenter study.

The two multicenter trials will be randomized, double-blind, placebo-controlled studies with three parallel treatment groups. Subject treatment will last 104 weeks. Each arm will enroll approximately 110 subjects for a total of approximately 330 subjects.

10.1 Baseline Comparability

Baseline variables, including demographic variables and history of disease, will be listed by subject within treatment group and compared by summary tables and statistical tests as appropriate to assess comparability of the three treatment groups.

10.2 Safety Evaluations

The following variables observed during and after treatment will be listed by subject within treatment group and be compared between treatment groups by summary tables, charts, and statistical tests as appropriate: physical examination findings, adverse

IV / 3342

experiences, concomitant medications, vital signs, weights, serum antibody titers to Betaseron and clinical laboratory parameters.

10.3 Efficacy Evaluation

Primary efficacy evaluations will be based on reduction in frequency of exacerbations per subject and proportion of exacerbation-free subjects.

Secondary efficacy evaluations will be based on severity and duration of exacerbations, time to first exacerbation, sequelae of exacerbations, size and number of lesions as determined by annual Magnetic Resonance Imaging, Kurtzke Neurologic Ratings, Scripps Neurologic Rating Scores, and Functional Neurologic Status.

Efficacy variables observed before, during, and after treatment will be listed by subject within treatment group and will be compared between treatment groups by summary tables, charts, and statistical tests as appropriate. Subgroup analysis according to baseline Kurtzke rating will be done.

10.4 Statistical Methodology

All subjects who are randomized and have received treatment (i.e., at least one dose) will be considered eligible for the intent-to-treat analyses. Two intent-to-treat analyses will be performed. The primary

IV / 3343

analyses will exclude the information collected on subjects after they have terminated. The secondary intent-to-treat analysis will include all data collected, including data from post-termination visits, for all subjects randomized (the original protocol did not call for this data to be collected). An additional analysis will be performed on a selected subset of subjects who are determined to be evaluable for efficacy according to specified criteria developed under blinded conditions.

Statistical significance will be assessed by Chi-square test or Fisher's exact test for proportions without stratification, by Mantel-Haenszel test for proportions with stratification, and by analysis of variance (ANOVA) of ranks for ordered and continuous variables. For comparisons without covariates, the ANOVA of ranks is the same as the two-sample Wilcoxon test. These tests are robust tests in that very large or very small values for a few subjects do not unduly influence the results. These tests are also nonparametric tests as they are not sensitive to distribution assumptions.

Baseline variables will be tested for association with outcome within treatment groups and, if associated, will be incorporated as covariates in tests between treatment groups.

IV / 3344

The model which will be used in the ANOVA of ranks analyses will incorporate site and country effects and interactions. The model which will be used is:

Y = TRT CNTRY SITE(CNTRY) TRT*CNTRY TRT*SITE(CNTRY) error

where:

Y = Dependent variable
TRT = Treatment
CNTRY = Country
SITE = Study site

10.5 Scheduled Efficacy Analyses

Two efficacy analyses are scheduled for this trial: one interim analysis and the final analysis at the completion of the study. The interim may be used to potentially file an early submission of a product licensing application. In any event, the study will continue until all subjects have either completed 104 weeks of treatment or have prematurely terminated.

The interim analysis will be based on one year of subject experience for the first 338 subjects. This will occur when subject information through July 31, 1990, is collected and verified. The final analysis will be done when these subjects have either completed 104 weeks of treatment or prematurely terminated.

IV / 3345

On July 31, 1989, the 330th subject was enrolled. Eight additional subjects were also enrolled on this day, making study enrollment 338 subjects as of the day the 330th subject was enrolled. For this reason, the subject population will consist of these 338 subjects.

The trial is designed so that, with one third of the subjects enrolled in each treatment arm, 330 subjects total, the power of pair-wise treatment comparisons (the probability of attaining a statistically significant result) is approximately 85%, assuming a placebo rate of 0.4 exacerbations per year and a 50% reduction in exacerbations due to the active treatment at a two-sided alpha level of 0.05. Assuming a placebo rate of 0.8 attacks per year raises the power to approximately 95% when keeping all other parameters the same. The power calculation takes into account early subject termination and multiple sites.

The O'Brien-Fleming method of sequential analysis is used to define the significance level, using a two-tailed test, for both of the efficacy analyses. Table 10.5.1 displays the probability of finding significant results (power) in each of the two analyses.

Table 10.5.1

Analysis	Two-sided Significance Level	Expected # Attacks for Subjects on Placebo	Expected # of Attacks for Subjects on Active Drug	Power
1 (interim)	0.005	0.80	0.4	57 %
2 (final)	0.048	1.60	0.8	95 %

IV / 3346

10.6 Maintenance of the Study Blind

The randomization code was produced by Triton's project statistician. The project statistician and supporting programmers are the only individuals with access to the randomization codes, with the exception of the drug packaging and labelling department, which also has one copy of the code to use in the labelling and packaging of the drug.

During the analysis of the data for the interim report, all listings that associate subject number with treatment group have the treatment group labelled by 1,2, or 3 rather than actual treatment assignment. No one except the project statistician and the support programmers have access to the code. In addition, all output included in the report will be censored by the project statistician wherever the result could potentially lead to a compromise of the blind for an individual subject. For example, an adverse event observed in only one or two groups would not be displayed by treatment group. Only the total number of subjects, without treatment identification, would be indicated for such an event. In keeping with this philosophy, no descriptive statistics, such as range, will be displayed in the interim report; group statistics only will be presented.

In the event the analysis shows significance at the 0.005 level (criterion established by O'Brien-Fleming see section 10.5), a meeting will be scheduled with the FDA to

determine the possibility of filing a PLA on the strength of the observed results. In no event will the blind be broken in-house prior to this meeting.

11. ETHICAL AND REGULATORY CONSIDERATIONS

The investigator will ensure that the study complies with all Regulatory and institutional requirements, including those for patient privacy, informed consent, institutional review board approval, and record retention.

11.1 Case Report Forms
Three-part case report forms will be supplied for each patient. These must be completed on an ongoing basis according to the procedures outlined by the sponsor. Original patient records and laboratory results and copies of the case report forms must be available at the study site for sponsor and regulatory inspection.

11.2 Adverse Event Reporting
Serious or unexpected adverse events and deaths occurring on study or during the 30 days after the last dose will be reported to the sponsor immediately even if the event is not considered drug related. The initial report to the project monitor and/or project physician may be made by telephone or telegram, but a written description of the event and sequelae must follow within 10 days of the initial report. If applicable, a copy of the death certificate and autopsy report will be forwarded as soon

as available. The institutional review board will also be notified of deaths and adverse events within 10 days; a copy of the report to the board will be sent to the project monitor.

12. REFERENCES

1. Cook D, Dowling PC: Multiple Sclerosis and viruses: An overview. Neurology July, 1980;30(2):80-91.
2. Koprowski H, DeFreitas ECC, et al. Multiple Sclerosis and human T-cell lymphotropic retroviruses. Nature 318:154-160 1985.
3. Adams JM, Imagawa DT,: Measles antibodies in multiple sclerosis. Proc Soc Exp Biol Med 1962;111:562.
4. Stewart WE: In: The Interferon System, Springer - Verlag, New York, 1979.
5. Kamin-Lewis R, Panitch HS, Merigan TC, et al: Decreased interferon synthesis and responsiveness to interferon by leukocytes from multiple sclerosis patients given natural alpha interferon. J Interferon Res 1984;4:423-432.
6. Benczur M, Petranyi GG, et al: Dysfunction of natural killer cells - multiple sclerosis, a possible pathogenic factor. Clin Exp Immunol 1980;39:657-62.
7. Hirsch RL, Johnson KP: The Effect of recombinant alpha-2-interferon on defective natural killer cell activity in multiple sclerosis. Neurol 1985;35(4):597-600.
8. Kamin-Lewis RM, Panitch HS, Johnson KP: Leukocytes from multiple sclerosis patients respond to alpha and gamma interferons. J Neuroimmunol 1985;9:221-227.
9. Knobler RL, Panitch HS, et al: Clinical trial of natural alpha interferon in multiple sclerosis. Ann NY Acad Sci 1984;436:382-388.
10. Jacobs L, O'Malley J, Freeman A, et al: Intrathecal interferon in multiple sclerosis. Arch Neurol 1982;39:609-15.

11. Jacobs L, Salazar AM, Herndon R, et al: Intrathecally administered natural human fibroblast interferon reduces exacerbations of multiple sclerosis. Arch Neurol 1987;44:589-595.
12. Panitch HS, Hirsh RL, Schindler J, et al: Treatment of multiple sclerosis with gamma interferon: exacerbations associated with activation of the immune system. Neurol 1987; 37:1097-1102.
13. Pober JS, Gimbone MA, Cotran RS, et al: Ia expression by vascular endothelium is inducible by activated T cells and by human interferon. J Exp Med 1983; 157:1339-1353.
14. Fierz W, Endler B, Reske K, et al: Astrocytes as antigen-presenting cells, I. Induction of Ia antigen expression on astrocytes by T cells via immune interferon and its effect on antigen presentation. J Immunol 1985; 134:3785-3793.
15. Sobel RA, Blanchette BW, Bhan AK, et al: The immunopathology of experimental allergic encephalomyelitis. II. Endothelial cell Ia increases prior to inflammatory cell infiltration. J Immunol 1984; 132:2402-2407.
16. Traugott U, Reinherz EL, Raine CS: Multiple sclerosis: distribution of T cells, T cell subsets and Ia-positive macrophages in lesions of different ages. J Immunol 1983; 4:201-221.
17. Traugott U, Raine CS: Multiple sclerosis: evidence for antigen presentation in situ by endothelial cells and astrocytes. J Neurol Sci 1985; 69:365-370.
18. Ling PD, Warren MK, Vogel SN: Antagonistic effect of interferon-β on the interferon-γ-induced expression of Ia antigen in murine macrophages. J Immunol 1985; 135(3):1857-1863.

19. Inaba K, Kitaura M, Kato T, et al: Contrasting effect of α/β- and γ-interferons on expression of macrophage Ia antigens. J Exp Med 1986; 163:1030-1035.
20. Investigator Brochure (April 15, 1987). On file, Triton Biosciences Inc, Alameda, CA.
21. Hawkins MJ, Horning S, Konrad M, et al: Phase I evaluation of a synthetic mutant of β-interferon. Cancer Res 1985;45:5941-5919.
22. Hawkins MJ, Davis TE, Merigan T: Phase I clinical study of recombinant human interferon beta in patients with advanced cancer (CSC-1983). Report on file, Triton Biosciences Inc, Alameda, CA.
23. Sarna G, Pertcheck M, Figlin R, et al: Phase I study of recombinant β ser 17 interferon in the treatment of cancer. Cancer Treat Rep 1986; 70(12):1365-1372.
24. Grunberg SM, Kempf RA, Venturi CL, et al: Phase I study of recombinant β-interferon given by four-hour infusion. Cancer Res 1987; 47:1174-1178.
25. Rinehart J, Malspeis L, Young D, et al: Phase I/II trial of human recombinant β-interferon serine in patients with renal cell carcinoma. Cancer Res 1986; 46:5364-5367.
26. Ozer H, Keegan P, Han T, et al: Comparison trial of natural and recombinant beta human interferon (IFN-β_{ser}) in patients with cancer (CSC-3984). Report on file, Triton Biosciences Inc, Alameda, CA.
27. Sarna G: Protocol on file (TB01-25185), Triton Biosciences Inc, Alameda, CA.
28. Sarna G: Open-label phase II study to evaluate the safety and efficacy of IFN-β_{ser} in patients with malignant melanoma (TB01-21784). Report on file, Triton Biosciences Inc, Alameda, CA.

29. Hu E, Horning SJ: Phase I study of recombinant human interferon beta in patients with advanced cancer. J Biol Resp Mod 1987; 47:1174-1178.
30. Lillis PK, Brown TD, Beougher K, et al: A phase II trial of recombinant beta interferon in advanced colorectal cancer. Report on file, Triton Biosciences Inc, Alameda, CA.
31. Ratanatharathorn V, Karanes C, Al-Sarraf M, et al: Phase I trial of serine substituted recombinant DNA beta interferon (β-IFN). Proc AACR 1985; 26:274 (1079).
32. Ratanatharathorn V, Karanes C, Martin S, et al: Phase I study of human recombinant beta interferon (IFN-β_{ser}) in human malignancies (CSC-1984). Report on file, Triton Biosciences Inc, Alameda, CA.
33. Borden EC, Hawkins MJ, Trump DL, et al: Phase I trial of human recombinant interferon (IFN-β_{ser}) in patients with advanced cancer (CSC-5984). Report on file, Triton Biosciences Inc, Alameda, CA.
34. Leavitt R, Sarna G, chang A, et al: A study of safety and clinical efficacy of the intravenous administration of beta-interferon (IFN-β_{ser}) in patients with miscellaneous neoplastic diseases (Jan 1986). Interim report on file, Triton Biosciences Inc, Alameda, CA.
35. Yung WKA, Castellanos AM, Moser RP: Intravenous recombinant beta interferon (IFN-β_{ser}) in patients with malignant gliomas. Neurol 1987: 37 (Supp 1):334.
36. Chang AYC, Pandya KJ, Asbury RF, et al: Phase I study of recombinant beta interferon ser (r-IFN-β_{ser}) in cancer patients by subcutaneous injection. Proc ASCO 1985; 4:226 (C-880).

37. Chang AYC, Bennett JM: Phase I study of human recombinant beta interferon (IFN-β_{ser}) in patients with malignant disease (CSC-2984). Report on file, Triton Biosciences Inc, Alameda, CA.
38. Chang AYC, Asbury RF, McCune C, et al: Phase I study of human recombinant beta interferon (IFN-β_{ser}) in cancer patients by intramuscular injection (CSC-10984). Report on file, Triton Biosciences Inc, Alameda, CA.
39. Fetell MR, Housepian EM, Oster MW, at al: Intratumoral administration of β-interferon in glioblastoma: phase I study. Neurol 1987; 37 (Supp 1):334.
40. Merigan T, Eisenberg M, Gregory P, et al: Pharmacokinetics, tolerance and effect on viral markers of multiple ascending doses of human recombinant beta$_{ser}$ interferon in patients with chronic hepatitis B (CSV-1184). Report on file, Triton Biosciences Inc, Alameda, CA.
41. Eisenberg M, Rosno S, Garcia G, et al: Preliminary trial of recombinant fibroblast interferon in chronic hepatitis B virus infection. Antimicrob Agents Chemother 1986; 29(1):122-126.
42. Higgins PG, Al-Nakib W, Willman J, et al: Interferon-β_{ser} as prophylaxis against experimental rhinovirus infection in volunteers. J Interferon Res 1986; 6:153-159.
43. Tyrrell DAJ: Phase I clinical study for the assessment of the clearance and tolerance of intranasally administered recombinant interferon beta in human subjects (CSV-1183). Report on file, Triton Biosciences Inc, Alameda, CA.
44. Hayden FG: Phase I intranasal tolerance study of IFN-β_{ser} in healthy normal volunteers (TB01-14285). Protocol on file, Triton Biosciences Inc, Alameda, CA.

45. Hayden FG, Innes DJ Jr, Mills SE, et al: Long-term tolerance of intranasal recombinant interferon-beta$_{serine}$ in man (abstract). presented at the meeting of the ISIR, Espoo, Finland, September 1986.
46. Tyrrell DAG, Higgins PG, Wellman JS, et al: Assessment of the effectiveness of intranasally administered interferon recombinant beta human in the prevention of experimental rhinovirus infections in human subjects (CSC-2184). Report on file, Triton Biosciences Inc, Alameda, CA.

Appendix 8

Investigator-administered neuropsychological tests

Content

1. Rosenbaum pocket vision screener 2
2. Wechsler Test of Adult Reading Ability (WTAR)..................................3
3. Controlled Oral Word Association Test (COWAT)................................4
4. California Verbal Learning Test-II..4
5. Symbol Digit Modality Test-Version A...6
6. Delis-Kaplan Executive Function System-free sorting task.....................8
7. The MACFIMS publication 14

A comparable set of French-language test will be provided in a seperate document.

1. Rosenbaum pocket vision screener

The Rosenbaum pocket vision screener is a hand-held eye chart to determine whether a patient's visual acuity is adequat to perform tests in the MACFIMS. Small print characters presented during the testing are similar to the characters presented at the 20/50-70 threshold when the Rosenbaum card is positioned 14 inches from the corrected eye.

Instruction:

Card is held in good light 14 inches from the corrected eye. Record vision for both eyes with glasses or contact lenses (should the patient use such devices).

Patient shall read the first line (i.e. 9, 5), then the second line and so forth.

Say: "Please, read aloud the first line!"
"Please, read aloud the second line!"
.....
and so forth

Please, look at the score-column called "distance equivalent". Write down the score-number shown in that line which the patient is not able to read (e.g. "20/30"). If the patient is able to read even the last line, score him "20/20". If the patient is unable to read symbols at 20/70, do not administer SDMT, D-KEFS, and WTAR.

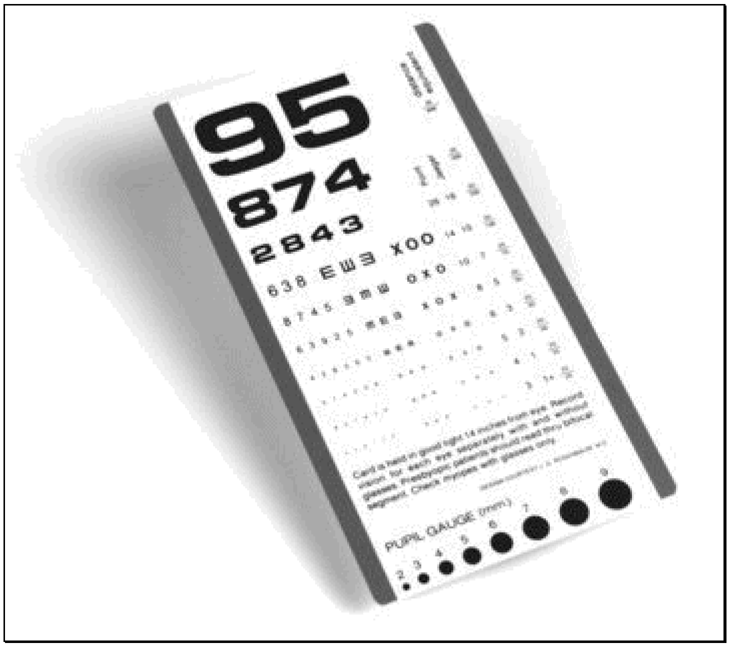

The Rosenbaum Pocket Visual Screener

2. Wechsler Test of Adult Reading Ability (WTAR)

The WTAR is applied to investigate the pre-morbid cognitive ability and as such serves the purpuse to evaluate potentially confounding factors during the neuropsychological testing.

Instructions for WTAR:

Begin administration of the WTAR by saying:

I will show you some words that I will ask you to pronounce

Place the WTAR word card (cf. below) in front of the examinee. As you point to the card, say:

Beginning with the first word on the list, pronounce each word aloud. Start with this word (point to Item 1), and go down this column, one right after the other, without skipping any. When you finish this column, go to the next column (point to the second column). Pronounce each word even if you are unsure. Do you understand?

If the examinee does not understand the instructions, you may repeat the instructions, paraphrasing if necessary.

When you are sure that the examinee understands the task, say,

Ready? Begin.

Recording and Scoring:

Acceptable pronunciations, including alternate pronunciations, for the WTAR words are provided on the record form and pronunciations tape. The examinee is required to give only one pronunciation of a word. Award one point for each correct response and 0 points for each incorrect response. Sum the points to obtain the WTAR raw score. The maximum score is 50.

WTAR word card

again	vengeance	homily	paradigm
address	prestigious	malady	perspicuity
cough	wreathe	subtle	plethora
preview	gnat	fecund	lugubrious
although	amphitheatre	palatable	treatise
most	lieu	menagerie	dilettante
excitement	grotesque	obfuscate	vertiginous
know	iridescent	liaison	ubiquitous
plumb	ballet	exigency	hyperbole
decorate	equestrian	xenophobia	insouciant
fierce	porpoise	ogre	hegemony
knead	aesthetic	scurrilous	
aisle	conscientious	ethereal	

3. Controlled Oral Word Association Test (COWAT)

The COWAT is applied to investigate the verbal fluency. For further justification of inclusion into the test battery, cf. Section 7 (the MACFIMS publication: Benedict RH, Fischer JS, Archibald CJ et al. Minimal neuropsychological assessment of MS patients: a consensus approach. Clin Neuropsychol 2002;16:381-97).

Instructions

The subject is told,

> *I'm going to give you a category name and I want you to tell me as many words as you can that are consistent with this category. For example, if the category was 'tools', you could say 'saw', 'hammer', 'pliers' etc.*
>
> *I do not want you to use the same word over again by adding a different ending and saying 'hammers', 'hammering', etc. Just tell me as many words as you can. You will have 90 seconds to do this. I'll tell you when to start and stop.*

The examiner signals the subject to begin and end each trial. Record responses verbatim on the answer sheet. If the subject stops before 90 seconds are up, encourage her/him to think of some more words.

In this study, Form A is used. The category 'fruits and vegetables' is used. The following instructions should be provided by the examiner:

> *The category is 'fruits and vegetables'. You may begin.*

Scoring

Scoring consists of summing the admissible responses. Inflections of the same word (cow, cows) are counted as one response. Words that are clearly not within the category are also not counted.

4. California Verbal Learning Test-II

The COWAT is applied to investigate memory. For further justification of inclusion into the test battery, cf. Section 7 (the MACFIMS publication: Benedict RH, Fischer JS, Archibald CJ et al. Minimal neuropsychological assessment of MS patients: a consensus approach. Clin Neuropsychol 2002;16:381-97). In this study, only the short-delay recall function will be investigated.

Instructions:

TRIAL 1 – The subject is told,

> *I'm going to read a list of words to you. Listen carefully, because when I'm through, I want you to tell me as many words as you can. You can say them in any order, just say as many of them as you can. Are you ready?*

Before proceeding, make sure that the examinee understands the task. Then read aloud the target words of List A at an even pace at a rate slightly longer than one second per word. The entire list should take a total of 18 to 20 seconds to read.

After reading the last word on the list, say:

> *Go ahead.*

Write the examinee's responses, including repetitions and intrusions, verbatim in the order in which they are recalled, in the column labeled 'TRIAL 1' on the record form.

Approximately 15 seconds after the examinee appears to have given his or her last response on a trial, or when the examinee indicates that he or she cannot remember any more words, provide a single prompt, such as:

> *Anything else?*

Mark 'Q' on the next blank line in the response column to indicate when the prompt was given. Record any responses reported by the examinee after the prompt.

TRIAL 2 – Say:

> *I'm going to read the same list again. Like before, tell me as many of the words as you can, iln any order. Be sure to also say words from the list that you told me the first time.*

Again, read the words of List A aloud at an even pace at a rate slightly longer than one second per word. The entire list should take a total of 18 to 20 seconds to read. Record the examinee's responses in the column labeled TRIAL 2 on the record form. Follow the same procedures for recording and prompting that were used for TRIAL 1.

For TRIALS 3 and 4, say:

> *I'm going to read the same list again. Like before, tell me as many of the words as you can, in any order, including words from the list you've said before.*

Follow the same procedures for reading the List A words, recording responses, and prompting as were used for the previous trials. Record the examinee's responses in the columns labeled TRIAL 3 and TRIAL 4 on the Record Form.

TRIAL 5 – Say:

I'm going to read the same list one more time. Like before, tell me as many of the words as you can, in any order, including words from the list you've said before.

Follow the same procedures for reading the List A words, recording responses, and prompting as were used for the previous trials. Record the examinee's responses in the column labeled TRIAL 5 on the record form.

<u>Word list</u>

Truck	Onion	Coach	Desk
Spinach	Motorcycle	Lamp	Boat
Giraffe	Cabinet	Celery	Squirrel
Bookcase	Zebra	Cow	Cabbage

5. Symbol Digit Modality Test-Version A

The COWAT is applied to investigate selective attention. For further justification of inclusion into the test battery, cf. Section 7 (the MACFIMS publication: Benedict RH, Fischer JS, Archibald CJ et al. Minimal neuropsychological assessment of MS patients: a consensus approach. Clin Neuropsychol 2002;16:381-97).

The subject is told,

*Look at these boxes (point to key). Notice that each has a mark in the upper part and a number in the lower part. Each mark has a different number. Now look down here (point), where the boxes have marks in the top part but the lower part of the boxes is blank. I'd like you to tell me which number should go in each box, like this. '**I***

Demonstrate with first two items.

"Now, when I say "BEGIN", you tell me which number should go in each of these boxes, up to this double line (point to double line).

Fill in subject's answers to the sample items, correcting himher after any incorrect response. Once the sample items are correctly completed, say

If When I say "BEGIN", work as quickly as you can, telling me which number should go in each box. Begin here, and do as many as you can, without skipping any. You may want to keep track of where you are with your finger. When you finish one row, just go on to the next. Keep working as quickly as you can, without making any mistakes, until I say "STOP".

Allow subject to work for 90 seconds. The test boxes of List A are shown below:

Symbol Digital Modalities Test / A
Patient Form

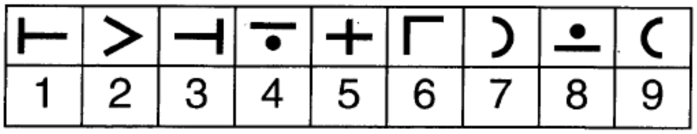

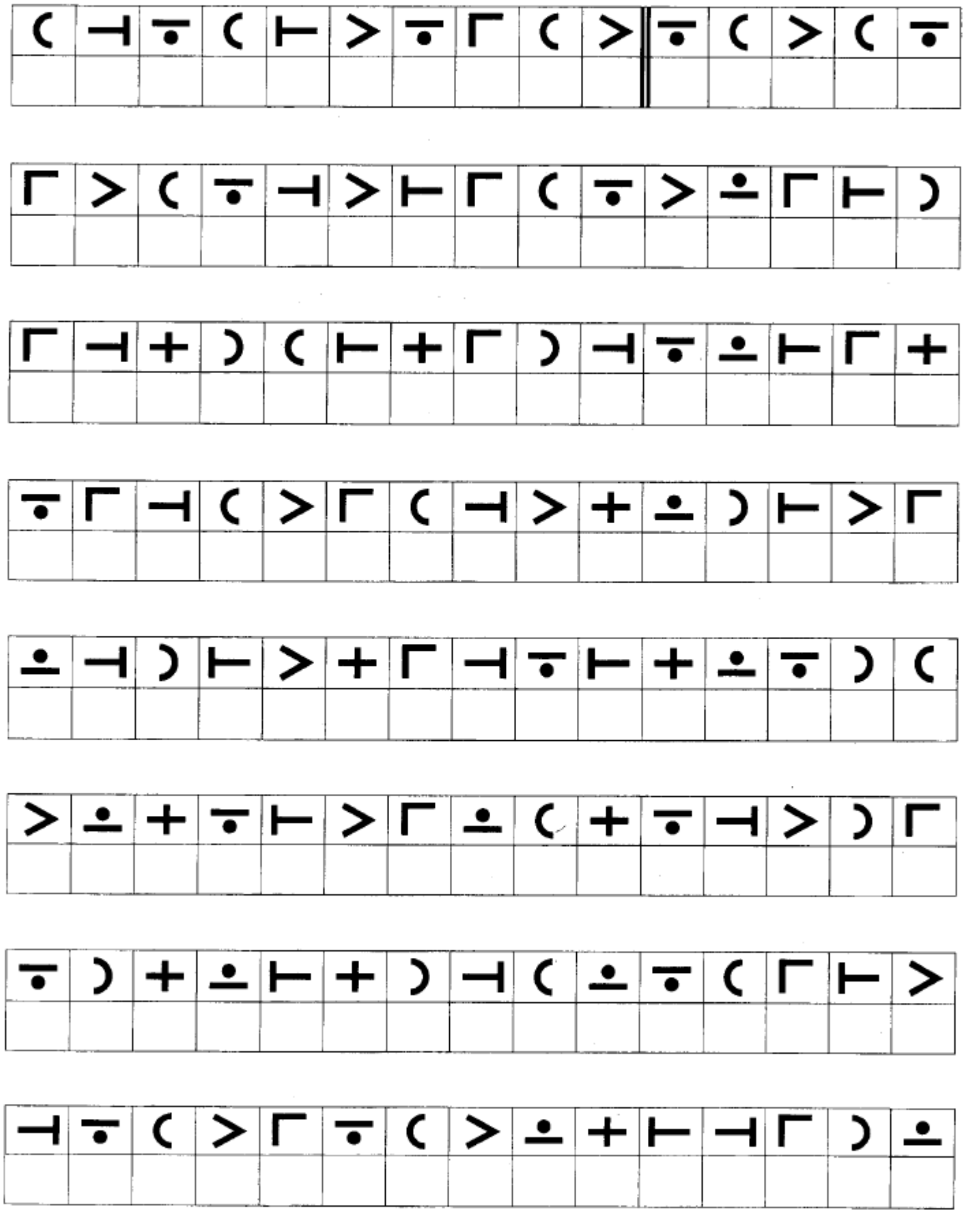

6. Delis-Kaplan Executive Function System-free sorting task (D-KEFS)

The D-KEFS is applied to investigate executive function. For further justification of inclusion into the test battery, cf. Section 7 (the MACFIMS publication: Benedict RH, Fischer JS, Archibald CJ et al. Minimal neuropsychological assessment of MS patients: a consensus approach. Clin Neuropsychol 2002;16:381-97).

<u>Instructions for Practice Card Set</u>

Condition 1: free sorting

Say,

> *I'm going to show you six cards that can be sorted in different ways. I want to see how many different ways you can sort the cards. Let me show you what I mean with these cards.*

Place the six cards of the Practice Card Set in a random, oval arrangement (an example is shown in the figure below) on the table of the examinee. Position the cards about one inch apart from one another. Make sure the words on the cards are facing the examinee.

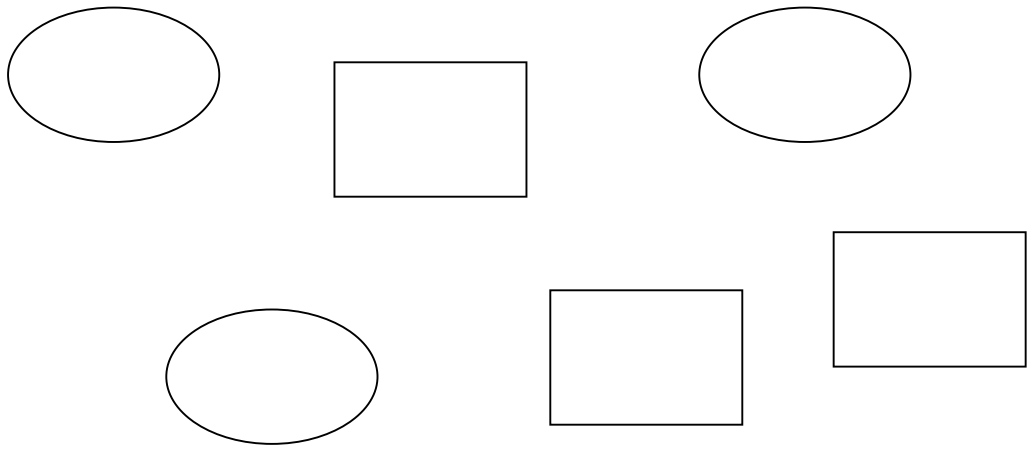

Example of a Presentation Arrangement for the D-KEFS Sorting Cards

Say,

> *Look at these cards. Watch how I sort them into two groups, with three cards in each group.*

Sort the cards into the following two groups: circles and squares. Align the two groups of three cards each in two vertical columns, side by side, about one foot apart, as shown in the figure below:

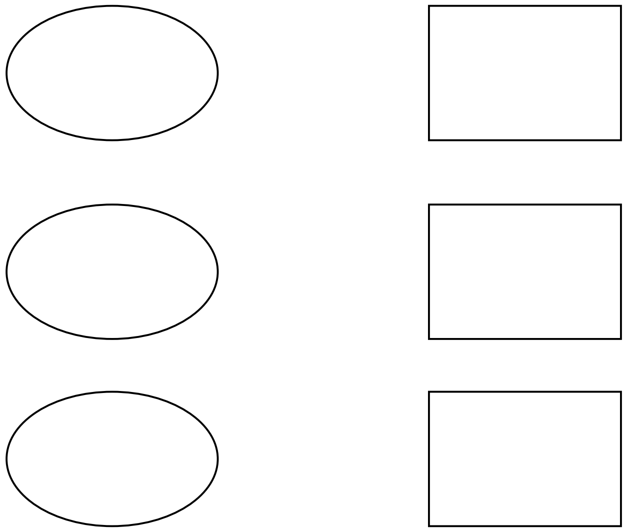

Arrangement of first sort of the Practice Card Set

Pointing to the appropriate groups in turn, say,

> *Next I'll explain how I sorted them by saying, this group has circles, and this group has squares. Notice how I explained both groups, not just one of them.*

Provide additional explanation as necessary. Then, mix up the six cards and place them in a random, oval arrangement before the examinee, with the words facing the examinee.

Say,

> *Now watch while I sort them another way, again with two groups and three cards in each group.*

Sort the cards into the following two groups: male names (John, Steve and Mike) and female names (Maria, Jane, and Diana). Pointing to the appropriate groups in turn, say,

> *I will explain how I sorted them by saying, this group has boys' names, and this group has girls' names. Do you have any questions about how I did this?*

Answer any questions. Remove the Practice Card Set cards

Instructions for screening pretest:

The screening pretest serves to ensure, that the examinee can read and understand the stimulus words printed on the sorting cards (the words are second-grade reading level). Open the stimulus booklet to the screening pretest and place it in an easel position so that the examinee can easily read the word list. Say,

> *I'd like you to read these words out loud and tell me if there are any words you would like me to explain. Go ahead.*

If the examinee reads a word incorrectly, record the word in the designated space on the record form and provide the correct pronunciation. After the examinee reads all of the words, say,

> *Do you know the meanings of all of these words?*

If the examinee does not know the meaning of a word, read the definition provided in the D-KEFS-word description list. Do not allow the examinee to see the list. You may repeat these definitions if the examinee asks you to do so. In the space provided in the record form, record any word with which the examinee has difficulty understanding. Sum (a) the number of words that the examinee read incorrectly and (b) the number of words that the examinee failed to understand and for which he or she required explanation.

D-KEFS word descriptions

Airplane	a vehicle that flies in the air with wings
Bus	a large motor vehicle that can carry many people
Car	a motor vehicle moving on four wheels
Coffee	a hot drink often used to start the day
Duck	a swimming bird with a bill and webbed feet
Eagle	a bird of prey known for ist sharp vision and flying ability
Ears	parts of the body used for hearing
Hat	something worn on the head for warmth or shade
Heat wave	a period of time when it is unusually hot
Iced tea	a cold drink prepared from tea leaves
Milk shake	a drink usually made from milk, syrup, and ice cream
Mouth	a part of the head used for eating and speaking
Rice	a white or brown grain that is often eaten with vegetables or sauces
River	a large stream of flowing water
Rocks	hard pieces of mineral or stone
Sandwich	two slices of bread containing food such as meat or cheese
Sea	a large body of salt water
Shoe	something worn on the foot for warmth and protection
Snow	water that freezes into soft, white flakes and falls from the sky
Socks	knitted or woven material worn on the foot along with shoes
Soup	a liquid food usually with meat, fish, or vegetables flavor and usually served hot
Sunshine	light or rays from the sun
Tiger	a large, meat-eating animal that belongs to the cat family
Toes	the five front parts of a foot

Instructions for Test

Card Set 1 (Standard Form) / Card Set 3 (Alternate Form)

Use Card Set 1 for the Standard Form. Leave the stimulus booklet in its easel position.

Use the following prompts as indicated. If 30 seconds elapse with no sorting response, say,

Try to sort them into two groups.

Provide the prompt only once for each card set administered.

If a description response is not given after a sort, say,

How did you make the two groups?

Explain only once each new rule violation made during the entire sorting test. For only the first overly abstract description, say,

Good, but how did you specifically sort each group?

Provide this prompt only once for the entire Sorting test.

The examinee may be reminded of previous description responses for a card set but only if he or she explicitly requests that information. Say,

I'm going to show you six new cards that can be sorted in many different ways. I'd like to see how many different ways you can sort these cards. Each time, make only two groups with three cards in each group. The three cards in each group should be the same in some way. After you sort the cards into two groups, tell me how you did it. Be sure to tell me how you sorted both groups, not just one of them. Once you sort the cards one way, do not sort them that way again. Work as quickly as you can.

Turn the page in the stimulus booklet to display the summarized instructions to the examinee. Point to the summarized instructions on the stimulus page and say,

Here is a page that will help you remember the rules.

Review the instructions while pointing to the five rules. Leave the summarized instructions in the examinee's view throughout the remainder of the Free Sorting condition.

Place the six cards from Card Set 1 (Standard form) in a random, oval arrangement in front of the examinee. Make sure the words on the cards are facing the examinee. Say,

Now try sorting these cards in as many different ways as you can. Ready? Begin.

Start timing. Allow the examinee to sort the cards into two groups. The examinee is not required to arrange the card groups into vertical columns but simply to cluster them together in some way.

Stop the stopwatch when the examinee has completed his or her sort and begins to describe his or her first sorting strategy. Record verbatim the examinee's description, the sorting response made, and the elapsed sorting time (not description time). Do not reset the stopwatch to zero; the previous sorting time is added to all subsequent sorting times within each card set.

Then mix up the cards and place them in a random, oval arrangement in front of the examinee, with the words on the cards facing the examinee. Say,

Now try to sort them in a different way.

Start timing. Repeat the procedures for each sort produced by the examinee until a discontinue criterion is met. After recording the total cumulative sorting time for the first card set administered, reset the stopwatch to zero.

Remove the Card Set 1 or Card Set 3.

Card Set 2 (Standard Form) / Card Set 4 (Alternate Form)

Use Card Set 2 for the Standard Form or Card Set 4 for the Alternate Form. Leave the stimulus booklet in its easel position. Say,

I'm going to show you six new cards that can be sorted in many different ways. Like before, I'd like to see how many different ways you can sort these cards. Each time, make only two groups with three cards in each group. The three cards in each group should be the same in some way. After you sort the cards into two groups, tell me how you did it. Be sure to tell me how you sorted both groups, not just one of them. Once you sort the card one way, do not sort them that way again. Work as quickly as you can.

Point to the summarized instructions on the stimulus page and say,

Again, here is the page that will help you remember these rules.

Review the instructions if necessary. Leave the summarized instructions in the examiner's view throughout the remainder of the Free Sorting condition.

Place the six cards from the Card Set 2 (Standard Form) or Card Set 4 (Alternate Form) in a random, oval arrangement in front of the examinee. Make sure the words on the cards are facing the examinee. Say,

Now try sorting these cards in as many different ways as you can. Ready? Begin.

Start timing. Allow the examinee to sort the cards into two groups. The examinee is not required to arrange the card groups into vertical columns but simply to cluster them together in some way.

Stop the stopwatch when the examinee has completed his or her sort and begins to describe his or her first sorting strategy. Record verbatim the examinee's description, the sorting response made, and the elapsed sorting time (not description time). Do not reset the stopwatch to zero; the previous

sorting time is added to all subsequent sorting times within each card set. T hen mix up the cards and place them in a random, oval arrangement in front of the examinee, with the words on the cards facing the examinee. Say,

Now try to sort them in a different way.

Start timing. Repeat these procedures for each sort produced by the examinee until a discontinue criterion is met. After recording the total cumulative sorting time for the second card set administered, reset the stopwatch to zero.

Remove the Card Set 2 or Card Set 4 cards.

7. The MACFIMS publication

Benedict RH, Fischer JS, Archibald CJ et al. Minimal neuropsychological assessment of MS patients: a consensus approach. Clin Neuropsychol 2002;16:381-97.

The Clinical Neuropsychologist
2002, Vol. 16, No. 3, pp. 381–397
1385-4046/02/1603-381$16.00
© Swets & Zeitlinger

Minimal Neuropsychological Assessment of MS Patients: A Consensus Approach

Ralph H.B. Benedict[1,2], Jill S. Fischer[3], Cate J. Archibald[4], Peter A. Arnett[5], William W. Beatty[6], Julie Bobholz[7], Gordon J. Chelune[8], John D. Fisk[9], Dawn W. Langdon[10], Lauren Caruso[11], Fred Foley[12], Nicholas G. LaRocca[13], Lindsey Vowels[14], Amy Weinstein[15], John DeLuca[16], Stephen M. Rao[7], and Frederick Munschauer[1,2]

[1]Department of Neurology, State University of New York (SUNY) at Buffalo, Buffalo, NY, USA, [2]Jacobs Neurological Institute, Buffalo, NY, USA, [3]Independent Consulting Practice, Chicago, IL, USA, [4]Foothills Medical Center, Calgary, Alberta, Canada, [5]Psychology Department, Penn State University, University Park, PA, USA, [6]Psychiatry Department, Oklahoma University Health Sciences Center, Oklahoma City, OK, USA, [7]Neurology Department, Medical College of Wisconsin, Milwaukee, WI, USA, [8]Mellen Center for MS Treatment and Research, Cleveland, OH, USA, [9]Department of Psychology, Queen Elizabeth Health Sciences Center, Halifax, Nova Scotia, Canada, [10]Department of Psychology, University of London, Surrey, UK, [11]Department of Neurology, New York University Medical School, New York, NY, USA, [12]Department of Psychology, Yeshiva University, Bronx, NY, USA, [13]National MS Society, New York, NY, USA, [14]MS Society of Victoria, Victoria, Australia, [15]Department of Neurology, University of Rochester Medical Center, Rochester, NY, USA, and [16]Neuropsychology and Neuroscience Laboratory, Kessler Medical Rehabilitation Research and Education Corporation, West Orange, NJ, USA

ABSTRACT

Cognitive impairment is common in multiple sclerosis (MS), yet patients seen in MS clinics and neurologic practices are not routinely assessed neuropsychologically. In part, poor utilization of NP services may be attributed to a lack of consensus among neuropsychologists regarding the optimal approach for evaluating MS patients. An expert panel composed of neuropsychologists and psychologists from the United States, Canada, United Kingdom, and Australia was convened by the Consortium of MS Centers (CMSC) in April, 2001. Our objectives were to: (a) propose a minimal neuropsychological (NP) examination for clinical monitoring of MS patients and research, and (b) identify strategies for improving NP assessment of MS patients in the future. The panel reviewed pertinent literature on MS-related cognitive dysfunction, considered psychometric factors relevant to NP assessment, defined the purpose and optimal characteristics of a minimal NP examination in MS, and rated the psychometric and practical properties of 36 candidate NP measures based on available literature. A 90-minute NP battery, the Minimal Assessment of Cognitive Function in MS (MACFIMS), emerged from this discussion. The MACFIMS is composed of seven neuropsychological tests, covering five cognitive domains commonly impaired in MS (processing speed/working memory, learning and memory, executive function, visual-spatial processing, and word retrieval). It is supplemented by a measure of estimated premorbid cognitive ability. Recommendations for assessing other factors that may potentially confound interpretation of NP data (e.g., visual/sensory/motor impairment, fatigue, and depression) are offered, as well as strategies for improving NP assessment of MS patients in the future.

Cognitive dysfunction is common in MS, occurring in nearly half of all patients (Heaton, 1985; Rao, Leo, Bernardin, & Unverzagt, 1991). The adverse impact of NP impairment is well documented in studies of employment (Beatty, Blanco, Wilbanks, & Paul, 1995; Rao, Leo,

Address correspondence to: Ralph H.B. Benedict, PhD, Department of Neurology, Buffalo General Hospital, Suite D-6, 100 High Street, Buffalo, NY 14203, USA. Tel.: +1-716-859-1403. Fax: +1-716-859-1419. E-mail: benedict@buffalo.edu
Accepted for publication: September 9, 2002.

Ellington, et al., 1991; Rao, Leo, Haughton, Aubin-Faubert, & Bernardin, 1989), social and avocational activity (Rao, Leo, Ellington, et al., 1991) and instrumental activities of daily living (Amato et al., 1995; Higginson, Arnett, & Voss, 2000; Rao, Leo, Bernardin, et al., 1991). NP impairment may also hinder a patient's capacity to benefit from rehabilitative therapies (Langdon & Thompson, 1999).

Unfortunately, MS patients' cognitive deficits are often difficult to detect during an interview or neurological examination, no matter how skilled the clinician (Fischer, 1989; Fischer et al., 1994; Peyser, Edwards, Poser, & Filskov, 1980). MS-related cognitive deficits are often focal rather than global, may be subtle in their presentation, and can vary from patient to patient (Fischer, 2001). Striking abnormalities of language and visual agnosias are rare (Fischer, 2001). Processing speed/working memory and learning/memory are the most commonly compromised functions, but deficits in executive functions and visual-spatial abilities also occur reasonably often, either in isolation or in combination with other deficits (Fischer, 2001; Rao et al., 1991). NP test performance is only weakly correlated with disease duration and physical disability (Beatty, Goodkin, Hertsgaard, & Monson, 1990; Rao, Leo, Bernardin, et al., 1991). Although correlations between NP performance and T2 lesion burden on magnetic resonance imaging (MRI) are stronger (Rao et al., 1989) they are still too weak to allow prediction in an individual case.

Despite the acknowledged difficulty of detecting cognitive impairment in an interview or neurologic examination, objective NP testing is not routinely employed in MS clinics. A variety of factors may account for this. NP assessment is not required to diagnose MS (McDonald et al., 2001; Poser et al., 1983) and cognitive deficits that might trigger a NP referral may not be evident during a follow-up office visit. NP testing may not be readily available in some settings, and even if available, may be costly and time consuming. With the advent of disease-modifying medications for MS and emphasis on early intervention and treatment, detection of cognitive impairment at its earliest stage becomes particularly important. Routine NP testing of MS patients is feasible, but only if it can be performed in a cost-effective manner.

NP assessment lies on a continuum ranging from brief *screening* measures used to identify patients in need of further testing, to *comprehensive evaluation* of a patient's deficits and strengths which is indicated when an individual is facing complex decisions regarding employment, driving, or capacity for financial decision-making. Routine NP testing would require an intermediate approach, a *minimal NP examination* that would assess the principal features of MS-related cognitive dysfunction. Several brief batteries for assessment of MS patients have been proposed (Franklin, Heaton, Nelson, Filley, & Seibert, 1988; Beatty et al., 1995; Basso, Beason-Hazen, Lynn J., & Bornstein, 1996). As is evident in Table 1, these limited NP batteries are too narrow for routine clinical assessment due to their omission of key cognitive domains in the interest of brevity. In contrast, the collaborative NP research battery proposed by Peyser, Rao, LaRocca, and Kaplan (1990) is inefficient for routine clinical NP assessment in that it incorporates measures of cognitive functions that are typically spared by MS (see Table 2). Furthermore, the Peyser battery includes non-standardized or abbreviated measures that would be difficult to apply clinically on a widespread basis.

Recognizing that approaches to NP assessment often differ across examiners and settings, the Consortium of MS Centers convened an expert panel to recommend a minimal set of NP tests for routine clinical assessment of MS patients. Methods used by previous consensus groups that focused on a core collaborative NP research battery (Peyser et al., 1990), gaps in the empirical literature for clinical NP practice in MS (Fischer et al., 1994), and attempts to develop a functional composite outcome measure for MS clinical trials (Fischer, Rudick, Cutter, & Reingold, 1999a; Rudick, Antel, Confavreux, et al., 1996), served as models for this effort. The panel's primary objectives were to: (a) identify a parsimonious set of currently available, psychometrically sound NP measures for assessing MS patients; and (b) develop strategies for improving the proposed battery in the future.

Table 1. NP Screening Batteries Proposed for MS.

Screening battery	Reference	Tests included
Neuropsychological Screening Battery (NSB)	Franklin et al. (1988)	Symbol-Digit Modalities Test (SDMT); Trails A, B; Numerical Attention Test; Prose learning (story from Wechsler Memory Scale); Visual learning and copy (part of Rey–Osterreith Complex Figure); Visual Naming, Aural Comprehension of Words and Phrases, and Speech Articulation Rating from Multilingual Aphasia Examination; Western Aphasia Battery Commands with Auditory Sequencing; Phonemic fluency (written and oral)
Brief Neuropsychological Battery (BNPB)[1]	Rao et al. (1991)	Paced Auditory Serial Addition Test (PASAT); Buschke Verbal Selective Reminding Test (12 items × 12 trials, with delay); 7/24 Spatial Recall Test; Controlled Oral Word Association Test (COWAT)
Screening Examination for Cognitive Impairment (SEFCI)	Beatty et al. (1995)	Verbal learning (10 items × 3 trials); Shipley Institute of Living Scale (SILS); SDMT (oral version)
Basso Screening Battery	Basso et al. (1996)	Seashore Rhythm Test; Wechsler Memory Scale – Revised Logical Memory; Controlled Oral Word Association Test; Halstead–Reitan Graphesthesia and Stereognosis

[1]A slightly modified version of this battery, termed the Brief Repeatable Neuropsychological Battery, has also been proposed, which includes the Selective Reminding Test (12 items × 6 trials); the 10/36 Spatial Recall Test; PASAT; SDMT; and phonemic fluency (3 items).

Table 2. Peyser et al. (1990) Core Battery of Neuropsychological Tests.

Cognitive domain	Test(s)
Global dementia screen	Mini-Mental State Examination (MMSE)
Processing speed/working memory	Auditory As, Trails A; Paced Auditory Serial Addition Test (PASAT); Symbol-Digit Modalities test (SDMT); Modified Stroop Test
Learning and memory	California Verbal Learning Test (CVLT); Wechsler Memory Scale – Revised (WMS–R) Logical Memory; 7/24 Spatial Recall Test
Reasoning and executive functions	Wisconsin Card Sorting Test (WCST); Raven's Standard Progressive Matrices; WAIS–R Comprehension
Visual-spatial abilities	Abbreviated Hooper Visual Organization Test (HVOT); Modified WAIS–R Block Design
Language	Abbreviated Token Test; Abbreviated Boston Naming Test; Controlled Oral Word Association Tests

Note. Auditory As, Trails A and the Raven's Standard Progressive Matrices were considered optional tests.

PROCESS OF DERIVING A MINIMAL NP EXAMINATION

Phase 1: Panel Selection and Conference Preparation

The expert panel was co-chaired by Drs. Benedict and Fischer. Neuropsychologists and clinical psychologists who had published extensively on neuropsychological aspects of MS, and experienced clinicians providing neuropsychological and/or psychological services in major MS clinics, were identified. Of the 19 individuals invited to the April, 2001 conference in New Orleans, 14 agreed to participate, 2 could not attend but agreed to offer comments on the results of the meeting, and 3 declined due to conflicting commitments. Panel members represented four countries: the United States, Canada, Australia, and the United Kingdom. In preparation for the conference, participants read a core literature on NP deficits in MS and psychometric considerations in NP assessment and reviewed lists of NP measures used in published MS studies (copies of the lists of articles and measures are available from the senior author).

Phase 2a: Defining the Features of an Optimal NP Examination in MS

At the conference, participants first agreed that an optimal minimal NP examination in MS should ideally:

- Be sensitive enough to detect mild cognitive impairment and be able to discriminate patients with subtle deficits from healthy controls.
- Monitor changes in cognitive function over time, both natural changes related to disease or normal aging and treatment-related changes (i.e., be *responsive* to change).
- Facilitate clinical decision-making, including treatment planning (with treatment broadly defined to include medication, rehabilitation, or counseling).
- Be applicable across a wide range of settings (i.e., be "translatable" to other languages and cultures, minimize the use of "high tech" equipment if possible, and minimize motor demands on the patient).
- Be parsimonious (i.e., administered in 1–2 hr).

After presentations on MS-related cognitive impairment, longitudinal studies of cognitive dysfunction, and the contribution of non-cognitive factors (e.g., depression, fatigue), five cognitive domains were deemed essential to include in the battery: (a) processing speed/working memory; (b) learning and memory; (c) visual-spatial processing; (d) executive function; and (e) language/other verbal abilities.

Phase 2b: Establishing Criteria for Evaluating Candidate Measures

The panel then considered psychometric issues in NP assessment, particularly those pertinent to repeated assessment (e.g., use of alternate forms). Following presentations on test-retest reliability and validity, participants discussed psychometric and practical criteria against which to evaluate candidate NP measures, ultimately arriving at a consensus on the criteria summarized in Table 3. Other desirable features of candidate measures identified by conference participants included construct validity (i.e., the extent to which a test measures what it purports to measure, as evidenced by its pattern of correlations with similar and dissimilar measures), efficiency (i.e., the number of distinct, meaningful scores that it yields), and ability to identify and separate underlying cognitive processes.

Phase 2c: Nominating and Evaluating the Merits of Specific Candidate Measures

Participants then nominated 36 measures to be considered for inclusion in the minimal NP examination (10 processing speed/working memory, 10 learning/memory, 7 executive function, 6 visual-spatial ability, and 3 language) (the list of candidate measures is available from the first author). Referring to published data in journal articles, test manuals, and standard NP references (e.g., Lezak, 1995; Spreen & Strauss, 1998), participants then evaluated how well each candidate measure fulfilled each of the criteria summarized in Table 3 using a 3-point scale (adequate [1], inadequate [−1], or equivocal/unclear [0]). In rare cases when published data were not available, unpublished data from work in progress by panel members were considered.

Table 3. Consensus Criteria for Evaluating Adequacy of Candidate Measures for the Minimal NP Battery.

Criterion	Explanation
1. Standardized stimulus materials and administration	Standardized test stimuli and instructions for test administration should be readily available.
2. Normative data	Published normative data on the test performance of a large sample of healthy adults in the age range frequently encountered in MS (20–55) should be available.
3. Adequate range	The test should be free of ceiling and floor effects.
4. Reliability	Test scores should have moderate to high test-retest reliability. Data pertaining to internal consistency and inter-rater reliability may also be available, but are less critical.
5. Criterion validity	The test should be able to discriminate MS patients (particularly mildly impaired patients) from healthy controls with reasonable, if not high, sensitivity and specificity.
6. Alternate forms	The test should have alternate forms of equivalent difficulty. This standard may not be equally applicable to all cognitive domains.
7. Practical	The test should be brief and easily administered in a wide variety of settings with minimal equipment. Test performance should not be strongly confounded by basic motor and sensory functions unless there is no other way to assess the function of interest.

Note. The adequacy of the 36 candidate measures was separately assessed on each criterion, using a 3-point scale (adequate [1], inadequate [−1], or equivocal/inconclusive data [0]).

Phase 3: Derivation of Minimal NP Exam

After the conference, the co-chairs prepared detailed tables containing scores for each candidate measure on each of the psychometric and practical criteria identified in Phase 2b (Table 3). Panel members then rank-ordered the five cognitive domains identified in Phase 2a in terms of their relative importance in MS assessment, and within each domain, rank-ordered the candidate measures based on consensus criteria. Descriptive statistics (sum, median, mode, minimum, and maximum) were used to summarize the distributions of panelists' rankings. These statistics were presented to the expert panel for review and comment. A recommended test had to: (a) be ranked first or second within its cognitive domain; (b) have a median ranking of 1 or 2; (c) have a modal ranking of 1 or 2; and (d) not be ranked as "unacceptable" by more than two panelists. This algorithm yielded clear choices in four of the five cognitive domains. However, no visual-spatial tests fulfilled this standard, and the rankings of the top two tests were very similar. Consequently, panel members were asked which of these two tests they preferred at which point a strong consensus emerged.

Phase 4: Postconference Work Groups

Three work groups reviewed the pertinent literature and offered recommendations regarding: (a) methods for assessing factors that may potentially confound interpretation of MS patients' NP performance (e.g., depression; fatigue; and visual, sensory, or motor impairments) (P.A., J.D.F., D.L.); (b) promising experimental methods for assessing processing speed/working memory (C.A., J.D.F.); and (c) alternative approaches to assessing verbal fluency that would be comparable across languages and cultures (W.B., D.L.). These work groups circulated summaries of their findings and recommendations to the entire panel for consideration, and consensus recommendations were incorporated into this article.

EXPERT PANEL RECOMMENDATIONS

Proposed Minimal NP Examination

There was nearly unanimous agreement about the rankings of cognitive domains, as processing speed/working memory and learning/memory were ranked first or second by nearly all panelists: processing speed/working memory (rank sum = 17, range = 1–3), learning and memory (rank sum = 25, range = 1–3), executive function (rank sum = 37, range = 2–4); visual-spatial ability (rank sum = 54, range = 4–5); language and other abilities (rank sum = 62, range = 3–5). These rankings parallel data on the prevalence of cognitive impairment in MS patients across domains (Rao, Leo, Bernardin, et al., 1991). The proposed minimal NP examination included two tests in each of the domains that the expert panel ranked as most important to assess (processing speed/working memory and learning/memory), and one test in each of the other domains. Table 4 presents this minimal NP examination for MS

Table 4. Tests in the Minimal Assessment of Cognitive Function in MS (MACFIMS).

Test	Median rank	% Panelists Ranking Test 1 or 2	Comment
Processing speed/Working memory			
Paced Auditory Serial Addition Test	1	86	Rao Version, using a 3.0 and 2.0 inter-stimulus interval. Normative data based on 100 healthy controls in peer-reviewed publication (Rao, Leo, Ellington, et al., 1991).
Symbol Digit Modalities Test	2	71	Oral administration only. Normative data based on 100 healthy controls in peer-reviewed publication (Rao, Leo, Ellington, et al. 1991).
Learning and memory			
California Verbal Learning Test–II	1	86	There are two equivalent alternate forms.
Brief Visuospatial Memory Test – Revised	2	71	There are six equivalent alternate forms.
Executive functions			
D-KEFS Sorting Test	1	79	The test is commercially available as of August, 2001. There are two administration formats. To conserve time, panel recommends administration of free sort condition only, for which separate norms are available.
Visual perception/Spatial processing			
Judgment of Line Orientation Test	1	93	The two forms (V, H) described in the Benton et al. (1994) manual are actually the same test items administered in a different order. Although odd/even item splits of the JLO have been evaluated, the panel recommends the full length JLO in the interest of ensuring test reliability and validity.
Language/other			
Controlled Oral Word Association Test	1	86	Two alternate forms, letters C, F, L and P, R, W. Suggested normative data provided by Benton and Hamsher (1989).

patients, referred to as the Minimal Assessment of Cognitive Function in Multiple Sclerosis (MACFIMS). Each test is briefly described below within a general discussion of its respective cognitive domain.

Deficits in information processing speed, and in the capacity to temporarily store and simultaneously manipulate verbal or visuospatial information in working memory, are well documented in MS patients (Archibald & Fisk, 2000; Beatty, Goodkin, Monson, & Beatty, 1989; Camp et al., 1999; D'Esposito et al., 1996; DeLuca, Johnson, & Natelson, 1993; Demaree, DeLuca, Gaudino, & Diamond, 1999; Fisk & Archibald, 2001; Franklin et al., 1988; Litvan, Grafman, Vendrell, & Martinez, 1988a; Litvan, Grafman, Vendrell, & Martinez, 1988b; Kujala, 1994; Rao, 1989). Available clinical measures unfortunately do not distinguish the relative contributions of processing speed and components of working memory. According to the expert panel's rankings, the Paced Auditory Serial Addition Test (PASAT) is the best of the currently available clinical measures at fulfilling the psychometric and practical criteria established for selecting measures for the minimal NP exam for MS.

First developed by Gronwall to assess patients recovering from concussion (Gronwall, 1977), the PASAT requires patients to monitor a series of 61 audiotaped digits while adding each consecutive digit to the one immediately preceding it. The PASAT requires both rapid information processing and simultaneous allocation of attention to two tasks, as well as reasonably intact calculation ability. In its original format, the PASAT was administered at four inter-stimulus intervals (2.4, 2.0, 1.6, and 1.2 s). The number of inter-stimulus intervals and presentation rates were subsequently modified by Rao and colleagues for use with MS patients (3.0 and 2.0 s) (Rao, Leo, Bernardin et al., 1991). This version was selected to be a component of the MS Functional Composite (MSFC), a clinical outcome measure composed of quantitative measures of leg, arm/hand, and cognitive function (Cutter et al., 1999; Fischer, Rudick et al., 1999; Rudick et al., 1996). The standard dependent variable on the PASAT is the total number of correct responses at each presentation rate (or a variant of this such as processing rate, which aggregates performance across presentation rates). The expert panel strongly recommended work on a more detailed analysis of PASAT performance that accounts for strategies used to perform well (e.g., dyad scoring) (Fisk & Archibald, 2001). Two equivalent alternate forms of the Rao version of the PASAT are available.

The expert panel also ranked a second measure of processing speed/working memory highly, the Symbol Digit Modalities Test (SDMT; Smith, 1982). The SDMT presents a series of nine symbols, each of which is paired with a single digit in a key at the top of an 8.5 × 11 in. sheet. The remainder of the page has a pseudo-randomized sequence of these symbols and the subject must respond with the digit associated with each of these as quickly as possible. Visual scanning and, to a lesser extent, secondary memory are involved in that patients must either rapidly locate the correct pairing on the key or recall these symbol-digit pairings. The SDMT was originally designed to permit either written or oral responses, but the panel recommended the oral administration with MS patients to minimize confounds due to upper extremity weakness or incoordination. The primary dependent variable on the SDMT is the total number of items correct in 90 s. Numerous alternate forms were developed by a National MS Society Cognitive Function Study Group (National Multiple Sclerosis Society Cognitive Functions Study Group, 1990) although a recent study suggests that these forms may not be equivalent (Boringa et al., 2001).

Poor recent memory is perhaps the most common NP complaint of MS patients, so it is not surprising that deficits on tests requiring recall of recently acquired information are often observed (Beatty, Goodkin, Monson, Beatty, & Hertsgaard, 1988; Fischer, 1988; Grant, McDonald, Trimble, Smith, & Reed, 1984; Minden, 1990; Rao, 1989; Rao, Hammeke, McQuillen, Khatri, & Lloyd, 1984). The California Verbal Learning Test, which has recently undergone revision (CVLT-II) (Delis, Kramer, Kaplan, & Ober, 2000), was the measure of learning and memory ranked most highly by nearly all members of the expert panel. Learning of a 16-word list (List A) is first examined over the course of five trials, then recall of a

new 16-word list (List B) is assessed on an interference trial, after which recall of List A is reassessed. Delayed recall and recognition (yes/no and forced choice) of List A are assessed again after a 25-min delay interval. The total number of words recalled on Trials 1–5 and on Delayed Free Recall are likely to be most sensitive to MS-associated cognitive impairment (Delis, Kramer, Kaplan, & Ober, 1987; Scarrabelotti & Carroll, 1998). The CVLT-II provides a rich array of additional variables that permit the examiner to examine cognitive processes that may affect overall performance (e.g., semantic clustering, susceptibility to interference, and learning curve), although scores on these variables may not be as stable statistically as the summary scores. One equivalent alternate form of the CVLT-II is available.

The Brief Visuospatial Memory Test – Revised (BVMT–R; Benedict, 1997; Benedict, Schretlen, Groninger, Dobraski, & Shpritz, 1996) is an efficient measure of spatial learning and memory that was also ranked highly by the majority of the panelists. Learning of a matrix of six simple abstract designs (presented for 10 s) is examined over three trials, and then delayed recall and recognition (yes/no) are assessed after 25 min. The BVMT–R is sensitive to cerebral disease in general (Benedict, 1997) and has also been used with MS patients (Benedict, Priore, Miller, Munschauer, & Jacobs, 2001). Some manual dexterity is required to produce the designs, but subjects are allowed as much time as necessary to complete them and motor problems evident on a copy trial are taken into account when scoring design accuracy. The BVMT–R offers six alternate forms, the equivalence of which is well established.

The term "executive functions" refers to a collection of related abilities comprising abstract reasoning, conceptual flexibility, and the planning and organization of behavior. Although less well studied than deficits in processing speed/working memory or learning/memory, deficits in executive functions occur with considerable frequency in MS (Beatty, 1995; Beatty & Monson, 1994; Beatty & Monson, 1996; Foong et al., 1999; Foong et al., 1997; Rao, Leo, Bernardin et al., 1991b). Moreover, impairments in executive functions can affect performance on tests of other cognitive abilities (Beatty & Monson, 1996; Foong et al., 1997; Troyer, Graves, & Cullum, 1994). The D–KEFS Sorting Test (DST; Delis, Kaplan, & Kramer, 2001), a measure of conceptual reasoning that permits the differentiation of concept formation from conceptual flexibility, emerged as the test of executive function most strongly favored by the expert panel. Standardized administration of the DST calls for both free sorting and structured (cued) sorting conditions, but the free sorting condition can be administered alone to reduce total testing time. Numerous dependent variables can be examined (e.g., number of sorts attempted, number of correct verbal concepts, number of correct perceptual concepts, number of perseverations, and types of rule violations). The DST has two alternate forms, an important quality for a measure of novel problem-solving that will be used to monitor changes in function over time.

Deficits in visual-spatial abilities are nearly as common as executive dysfunction in MS (Beatty et al., 1989; Rao, Leo, Bernardin et al., 1991; Ryan, Clark, Klonoff, Li, & Paty, 1996; Van den Burg, Van Zomeron, Minderhoud, Prange, & Meijer, 1987; Vleugels et al., 2000). The expert panel selected Benton's Judgement of Line Orientation Test (JLO; Benton, Sivan, Hamsher, Varney, & Spreen, 1994) as the best available measure of visual-spatial ability for the minimal NP examination. Brief versions of the JLO can be formed by administering only odd or even items (Woodard et al., 1996; Woodard et al., 1998) but the expert panel recommended the full-length JLO for the minimal NP exam. The JLO requires subjects to identify the angle defined by two stimulus lines from among those defined by a visual array of lines covering 180 degrees. The dependent variable on the JLO is the total number of correct responses.

MS patients may also present with subtle language problems, the most common of which is deficient word retrieval (Beatty et al., 1989; Rao, Leo, Bernardin et al., 1991b). The Controlled Oral Word Association Test (COWAT; Benton & Hamsher, 1989) a measure of phonemic fluency, was selected for the minimal NP exam based on its strong psychometric properties and

sensitivity to impairment in MS. On this task, subjects have 1 min to generate as many words as they can that begin with each of three different stimulus letters, which are presented individually. Thus, the COWAT cannot be considered a "pure" language measure test because performance is strongly affected by the efficiency and speed of searching one's lexicon. The principal dependent variable is the total number of words generated across all three trials. Strategies used to perform the COWAT (e.g., clustering, switching) can also be examined (Troyer, 2000; Troster et al., 1998), although the statistical reliability of these scores is not well established. Two equivalent alternate forms are available. Norms for other languages and cultures and the equivalence of these forms in other languages remain to be established.

Assessing Potential Confounds to NP Test Interpretation

Interpretation of the NP test performance of MS patients can be confounded by a number of factors, including variation in premorbid intellectual ability, depression, fatigue, sensory/motor impairment, and medications. The panel strongly recommended that: (a) a culturally appropriate measure of premorbid ability be administered the first time a patient is assessed; (b) a measure of depression be administered routinely in conjunction with the minimal NP exam; and (c) measures of visual/sensory/motor defects and fatigue be employed when indicated.

The influence of premorbid cognitive ability on NP test results is well established and clinicians routinely compare current test performance with an estimated baseline capacity (Lezak, 1995). Educational level and occupation are often used as surrogates for premorbid abilities, either informally, or formally through the application of regression formulas (Barona, Reynolds, & Chastain, 1984). However, educational and occupational attainment can be influenced by cultural expectations, particularly among older adults. Several tests used as surrogates for premorbid ability were suggested by the panel, with the acknowledgement that none is likely to be applicable across all countries and cultures. Most panel members favored the North American Adult Reading Test (NAART; Blair & Spreen, 1989) or the National Adult Reading Test in the UK and Australia (Nelson & Willison, 1991) to assess premorbid function. Selected verbal subtests from the WAIS-III (Wechsler, 1997; Information, Vocabulary, or the Verbal Comprehension Index) or the Reading subtest from the Wide Range Achievement Test, third edition (WRAT-III; Wilkinson, 1993) were also proposed by some members. These measures can be used to set general expectations for patient performance, bearing in mind that test scores are subject to regression to the mean (making subjects appear more "average" than they were premorbidly) and that premorbid ability may be underestimated in MS patients with global impairment (Friend & Grattan, 2000).

Depression is common in MS and can adversely affect NP performance, particularly on capacity-demanding tasks (Arnett, Higginson, Voss, Wright, et al., 1999; Arnett, Higginson, Voss, Bender, et al., 1999). Mood disturbance can also affect the accuracy of patients' perceptions of their performance (Fischer, 1989). The expert panel recommended the 42-item Chicago Multiscale Depression Inventory (CMDI; Nyenhuis et al., 1995), a self-report measure of depression, for use in conjunction with the MACFIMS. Although it may be less familiar than some measures, the CMDI has separate subscales for the Vegetative, Mood, and Evaluative components of depression, allowing the clinician to determine whether the total score may be "contaminated" by MS symptoms that overlap with those of depression (Mohr & Goodkin, 1999). The seven-item Beck Depression Inventory – Fast Screen (BDI–FS; Beck, Steer, & Brown, 2000), designed to screen medical patients for depression, is also promising but needs to be validated in an MS sample before it can be recommended.

Brief standardized measures of sensory and motor functions aid clinicians in interpreting NP test findings. Hand-held eye charts (e.g., Rosenbaum Pocket Vision Screener) can be used to determine whether a patient's visual acuity is adequate to perform tests in the MACFIMS. Small print characters presented during NP testing are similar to the characters presented at the 20/50–70 threshold when the Rosenbaum card is positioned 14 in. from the

corrected eye. By design, most tests in the MACFIMS require an oral rather than a manual motor response. Scoring on the BVMT–R, on which the patient must manually render a matrix of visual designs from memory, can be informally corrected for mild tremor by using the patient's copied designs as a reference (Benedict, 1997). The panel recommends the use of quantitative tests of upper extremity motor speed and coordination such as the 9-Hole Peg Test (Smith + Nephew, Notts, UK), which has been incorporated into the MS Functional Composite measure (Cutter et al., 1999; Fischer, Rudick, et al., 1999; Rudick et al., 1996). Finally, performance on tests requiring rapid oral responses can be confounded by dysarthria in some patients. The Maximum Repetition Rate of Syllables and Multisyllabic Combinations (Kent, Kent, & Rosenbek, 1987) is a brief series of tasks requiring the rapid repetition of syllables (e.g., "pa," "ta," and "ka") in separate 10-s trials, with the last trial involving three syllables repeated in sequence (e.g., "pa, ta, ka..pa, ta, ka"). This test was recommended to evaluate the degree to which a primary articulatory speed output problem is contributing to deficient performance on tests requiring rapid oral responses (i.e., PASAT, SDMT, and COWAT).

Early MS research suggested that testing-induced fatigue had little effect on NP test performance (Van den Burg et al., 1987). However, recent investigation suggests that fatigue can compromise performance on measures placing significant demands on processing speed and working memory (Johnson, Lange, DeLuca, Korn, & Natelson, 1997; Krupp, 2000). Consequently, measures such as the PASAT and the SDMT should be separated by measures of other cognitive functions during examination (see proposed order of administration in Table 5). Clinicians who suspect that fatigue may substantially affect a patient's test performance are encouraged to administer the Fatigue Impact Scale (FIS; Krupp, 2000). Scores of 75 or greater on the original (40-item) FIS are associated with significant functional limitations (Fisk, Pontefract, Ritvo, Archibald, & Murray, 1994). Both the original version of the FIS, and a 21-item version developed for the MS Quality of Life Inventory (MSQLI) by eliminating redundant items (Fischer, LaRocca, et al., 1999), contain a reliable Cognitive Fatigue subscale. However, as this subscale is highly correlated with other FIS subscales, it should not be interpreted as a "pure" measure of "cognitive fatigue."

Table 5. Suggested Order of Administration for Minimal NP Exam.

Measure	Administration time (min.)
CMDI (+ FIS if appropriate)	Variable
Record review/clinical interview	
Visual/sensory/motor confound measures as appropriate	
Visual acuity	
9-HPT	
MRRSMC	
COWAT	5
BVMT–R Learning Trials	7
PASAT	10
JLO	10
BVMT–R delayed recall and recognition	3
SDMT (oral administration)	5
CVLT-II learning trials and short-delay recall	15
DST (free sorting condition)	25
CVLT-II long-delay recall and recognition	10
(If initial assessment)	
Measure of premorbid abilities (NAART, NART, WRAT-3 Reading, or selected WAIS-III verbal subtests)	Variable

Note. CMDI = Chicago Multiscale Depression Inventory; FIS = Fatigue Impact Scale; 9-HPT = 9-Hole Peg Test; MRRSMC = Maximum Repetition Rate of Syllables and Multisyllabic Combinations; COWAT = Controlled Oral Word Association Test; BVMT–R = Brief Visuospatial Memory Test – Revised; PASAT = Paced Auditory Serial Addition Test; JLO = Judgment of Line Orientation; SDMT = Symbol-Digit Modalities Test; CVLT-II = California Verbal Learning Test – Second Edition; DST = D–KEFS Sorting Test; NAART = North American Adult Reading Test; NART = National Adult Reading Test; WRAT-3 = Wide Range Achievement Test – 3rd edition; WAIS-III = Wechsler Adult Intelligence Scale III.

DISCUSSION

The Minimal Assessment of Cognitive Function in MS (MACFIMS) is a clinically oriented NP examination of cognitive functions in MS. To be sure, deficits in these domains are not unique to MS, but rather, can be present in patients with many other conditions known to affect the cerebral white matter or cortical-subcortical circuits. This battery represents the consensus of an expert panel of 16 neuropsychologists and clinical psychologists with extensive experience in MS research and/or clinical care. The designation of these proposed tests as the 'Minimal Assessment of Cognitive Function in MS' should by no means imply that these are the only tests appropriate for assessing MS patients. Rather, these specific tests were selected because, in the view of the expert panel, they come closest to meeting psychometric and practical criteria established a priori for a minimal NP examination in MS. The MACFIMS is intended to be used both to detect cognitive impairment in MS patients and to monitor changes in cognitive function over time.

This battery can be administered by a trained examiner in approximately 90 min, although additional time is required for record review and clinical interview, administration of the self-report measure of depression, and formal assessment of other factors that may potentially confound NP test interpretation. While practical for the routine monitoring of MS patients in clinical settings and clinical trials, the MACFIMS is not intended for differential diagnosis of MS and other neurologic conditions. These tests can be supplemented by additional NP measures when a more detailed assessment of cognitive functions is desired, or when specific clinical questions require more comprehensive assessment (e.g., differential diagnosis, educational and occupational planning, and disability determination). As in any NP exam, appropriate clinical application and interpretation requires adequate training in clinical neuropsychology and familiarity with the quality of cognitive impairment associated with the suspected underlying disease.

The MACFIMS differs from the NP battery of Peyser et al. (Peyser et al., 1990) in that it targets only those functions commonly disrupted in MS, includes only clinical measures for which published norms are available, and employs tests selected using explicit psychometric and practical criteria. The MACFIMS is distinct also from the Brief Repeatable Battery proposed by Rao and colleagues (Rao, Leo, Bernardin, et al., 1991) and other brief NP batteries (Basso, et al., 1996; Beatty et al., 1995) in its broader coverage of cognitive functions. Guided by a conceptual consensus-driven approach to the assessment of specific cognitive functions, the expert panel consulted the literature to identify measures with demonstrated reliability and validity. This approach was deemed the most empirically sound approach possible, given the absence of multicenter NP datasets that would permit empirical comparisons of the psychometric properties of one test with another in the same population. In the future, it will be important to collect data on these measures at multiple sites to obtain uniform norms (for both single and repeated assessments) and to confirm the validity of the various tests over time.

Practice effects (i.e., improvement in task performance due to prior exposure to task stimuli and/or procedures when no real change in underlying abilities has taken place) are ubiquitous in NP assessment. Practice effects can complicate interpretation of NP performance when tests are readministered to monitor changes in cognitive function over time (Bever, Grattan, Panitch, & Johnson, 1995). In group studies, a control group can be used to estimate the magnitude of practice effects, as well as other factors unrelated to the treatment of interest (i.e., sources of systematic error, in statistical terms) (Fischer, 1999; Fischer et al., 2000). However, the clinician assessing an individual patient typically does not have such a reference group for estimating practice effects. Alternate forms have often been recommended as a method of minimizing the effects of practice on test performance. Indeed, the use of alternate forms can mitigate practice effects to some extent and can help preserve construct validity on some measures (Benedict & Zgaljardic, 1998). Unfortunately, the equivalence of alternate forms is often assumed, but it may not be confirmed when explicitly investigated (Boringa et al., 2001). Even when the equivalence of alternate forms is well established, test/retest reliability

may be attenuated from one test session to another, which increases the error term in a statistical analysis and potentially makes it more difficult to detect a reliable change in test performance. Furthermore, it is impossible to eliminate practice effects entirely when prior exposure to the task procedures themselves (not just the stimuli) facilitates subsequent task performance. We propose that more research is needed before the use of alternate forms of NP measures can be unconditionally recommended when assessment of change is paramount. Normative studies using the MACFIMS (both cross-sectional and longitudinal) are planned.

As NP assessment evolves, the MACFIMS must be flexible enough to incorporate improvements in its component measures and to allow new tests to be substituted if they are psychometrically superior. Processing speed/working memory is the cognitive domain in which improvements are most sorely needed. The calculation component of the PASAT may potentially confound interpretation of test performance in patients with premorbid calculation difficulties. Some patients may "chunk" stimulus items as they perform the task, especially during the faster paced trials, which alters the PASAT's working memory demands (Fisk & Archibald, 2001). Furthermore, clinical experience with the PASAT suggests that it is perceived as challenging by most patients and as unpleasant by some. The SDMT is better received, but SDMT performance can also be contaminated by difficulties with visual acuity or visual scanning. The ideal measure of processing speed/working memory would be sensitive to subtle defects and posses the psychometric strengths of the PASAT and the SDMT.

Three promising experimental paradigms have been used to assess working memory in MS: (a) Baddeley and Hitch's dual task paradigm (Baddeley & Hitch, 1974) applied to MS patients by D'Esposito and colleagues (D'Esposito et al., 1996); (b) the Keeping Track Task (Salthouse, 1992; Salthouse, Babcock, & Shaw, 1991) which assesses two related components of working memory; and (c) the *n*-back procedure, as applied in MS (Wishart et al., 2001). Dual task experiments typically require patients to perform primary and secondary tasks first alone, and then together, to evaluate decrements in performance associated with allocation of attention to two tasks. While this methodology may be sensitive to cognitive dysfunction in MS, it has not been well standardized. The Salthouse procedure is sensitive to subtle deficits in MS patients that are not evident on standard clinical NP measures (Archibald & Fisk, 2000). However, like the PASAT, it is confounded by calculation ability. The *n*-back procedure may be more promising to develop as a clinical measure. In this task, subjects are asked simply to report whether the current stimulus is the same as that presented "*n*" (i.e., 1, 2, or 3) items previously. Like the PASAT, the *n*-back procedure requires temporary storage, rehearsal, and updating of information held in working memory. Unlike the PASAT or the SDMT, however, other cognitive operations such as calculation and visual scanning are not involved. Stimuli can potentially be presented either aurally or visually, and response alternatives can be simplified to a dichotomous yes/no or go/no-go response. Computerized versions of the *n*-back paradigm have been employed widely in functional neuroimaging studies (Botvinick, Nystrom, Fissell, Carter, & Cohen, 1999; Braver et al., 1997; Cohen et al., 1997) demonstrating modality-specific lateralized activation of anterior cortical regions (Smith, Jonides, & Koeppe, 1996). In a recent pilot study of MS patients, frontal cortex activity during an *n*-back procedure was related to total lesion volume (Wishart et al., 2001). To be widely applicable clinically, however, the *n*-back paradigm must have standardized administration procedures and stimuli (preferably in a "low tech" version), its psychometric properties must be documented, its sensitivity to MS-associated impairment must be compared with that of established measures (i.e., PASAT and SDMT), and normative data must be collected.

Assessment of verbal fluency also needs to be developed further in order to achieve the expert panel's goal of "translatability" of the MACFIMS. Although phonemic fluency tests, which require subjects to generate words rapidly based on their lexical attributes (e.g., COWAT) are sensitive to MS-related cognitive impairment (Pozzilli et al., 1991; Rao, Leo, Bernardin, et al.,

1991) and are balanced for word frequency in English, they are not necessarily equivalent across languages. One solution might be to develop a new phonemic fluency test using letters that yield high word output across different languages and countries. A different approach is to shift to a semantic fluency measure with categories that have a large number of exemplars in many or all languages, thereby avoiding floor effects. In an investigation of the COWAT and two semantic fluency tasks (animals and parts of body) in a U.S. sample of 203 MS patients and 87 healthy controls (Beatty, 2001), receiver operating characteristic (ROC) curve analyses indicated that sensitivity and specificity values for discriminating patients from controls were quite similar for all three fluency measures, particularly when globally impaired patients were excluded from the analysis. This finding, if replicated in other countries, could lead to normative data collection on fluency measures that are truly equivalent across languages and cultures, at least in mildly to moderately impaired patients. To be sure, even tests of functions other than verbal abilities may have different psychometric properties when applied to patients with different languages and cultures. Demonstration of the 'translatability' of the MACFIMS must await collection of normative data from patients differing in language and culture.

In conclusion, cognitive impairment in MS is common, functionally disabling, and difficult to detect without formal NP assessment. In this paper, we propose a practical, rationally derived, clinically oriented minimal NP battery that is based upon expert consensus regarding the cognitive functions that are most important to assess in MS. It is flexible enough to accommodate improvements in its component measures and to allow substitution of new tests if they are psychometrically superior to current tests. The MACFIMS can be applied routinely with MS patients to detect deficits in core cognitive domains, particularly subtle deficits in patients who may benefit from early treatment, and to monitor changes in cognitive function over time. Thus, it fills a crucial gap between brief NP screening measures used to identify patients in need of further testing and comprehensive NP evaluations that are often impractical for routine assessment.

ACKNOWLEDGEMENTS

This project was supported by the Consortium of Multiple Sclerosis Centers (CMSC) through an unrestricted educational grant from Biogen, Inc. The authors thank June Halper, MSN, ANP, FAAN, Tina Trott, and Kelly Sallinger for their assistance in organizing the panel and meeting.

REFERENCES

Amato, M.P., Ponziani, G., Pracucci, G., Bracco, L., et al. (1995). Cognitive impairment in early-onset multiple sclerosis: Pattern, predictors, and impact on everyday life in a 4-year follow-up. *Archives of Neurology, 52*, 168–172.

Archibald, C.J., & Fisk, J.D. (2000). Information processing efficiency in patients with multiple sclerosis. *Journal of Clinical and Experimental Neuropsychology, 22*, 686–701.

Arnett, P.A., Higginson, C.I., Voss, W.D., Bender, W.I., Wurst, J.M., & Tippin, J.M. (1999). Depression in multiple-sclerosis: Relationship to working memory capacity. *Neuropsychology, 13*, 546–556.

Arnett, P.A., Higginson, C.I., Voss, W.D., Wright, B., Bender, W.I., & Wurst, J.M. (1999). Depressed mood in multiple sclerosis: Relationship to capacity-demanding memory and attentional functioning. *Neuropsychology, 13*, 434–446.

Baddeley, A., & Hitch, G. (1974). Working memory. In G.A. Bower (Ed.), *Recent advances in learning and motivation* (pp. 47–90). New York: Academic Press.

Barona, A., Reynolds, C.R., & Chastain, R. (1984). A demographically based index of pre-morbid intelligence for the WAIS–R. *Journal of Consulting and Clinical Psychology, 52*, 885–887.

Basso, M.R., Beason-Hazen, S., Lynn, J., Rammohan, K., & Bornstein, R.A. (1996). Screening for cognitive dysfunction in multiple sclerosis. *Archives of Neurology, 53*, 980–984.

Beatty, W.W. (1995). Verbal abstraction deficit in multiple sclerosis. *Neuropsychology, 9*, 198–205.

Beatty, W.W. (2001). Verbal fluency in multiple sclerosis: Which measure is best? *Multiple Sclerosis*.

Beatty, W.W., Blanco, C.R., Wilbanks, S.L., & Paul, R.H. (1995). Demographic, clinical, and cognitive characteristics of multiple sclerosis patients who

continue to work. *Journal of Neurologic Rehabilitation, 9*, 167–173.

Beatty, W.W., Goodkin, D.E., Hertsgaard, D., & Monson, N. (1990). Clinical and demographic predictors of cognitive performance in multiple sclerosis: Do diagnostic type, disease duration, and disability matter? *Archives of Neurology, 47*, 305–308.

Beatty, W.W., Goodkin, D.E., Monson, N., & Beatty, P.A. (1989). Cognitive disturbances in patients with relapsing remitting multiple sclerosis. *Archives of Neurology, 46*, 1113–1119.

Beatty, W.W., Goodkin, D.E., Monson, N., Beatty, P.A., & Hertsgaard, D. (1988). Anterograde and retrograde amnesia in patients with chronic progressive multiple sclerosis. *Archives of Neurology, 45*, 611–619.

Beatty, W.W., & Monson, N. (1994). Picture and motor sequencing in multiple sclerosis. *Journal of Clinical and Experimental Neuropsychology, 16*, 165–172.

Beatty, W.W., & Monson, N. (1996). Problem solving by patients with multiple sclerosis: Comparison of performance on the Wisconsin and California Card Sorting Test. *Journal of the International Neuropsychological Society*, 2, 134–140.

Beatty, W.W., Paul, R.H., Wilbanks, S.L., Hames, K.A., Blanco, C.R., & Goodkin, D.E. (1995). Identifying multiple sclerosis patients with mild or global cognitive impairment using the Screening Examination for Cognitive Impairment (SEFCI). *Neurology, 45*, 718–723.

Beck, A.T., Steer, R.A., & Brown, G.K. (2000). *BDI-Fast Screen for Medical Patients: Manual*. San Antonio, TX: Psychological Corporation.

Benedict, R.H.B. (1997). *Brief Visuospatial Memory Test – Revised: Professional manual*. Odessa, Florida: Psychological Assessment Resources.

Benedict, R.H.B., Priore, R.L., Miller, C., Munschauer, F.E., & Jacobs, L.D. (2001). Personality disorder in MS correlates with cognitive impairment. *Journal of Neuropsychiatry and Clinical Neurosciences, 13*, 70–76.

Benedict, R.H.B., Schretlen, D., Groninger, L., Dobraski, M., & Shpritz, B. (1996). Revision of the Brief Visuospatial Memory Test: Studies of normal performance, reliability, and validity. *Psychological Assessment, 8*, 145–153.

Benedict, R.H.B., & Zgaljardic, D.J. (1998). Practice effects during repeated administrations of memory tests with and without alternate forms. *Journal of Clinical and Experimental Neuropsychology, 20*, 339–352.

Benton, A.L., & Hamsher, K. (1989). *Multilingual Aphasia Examination*. Iowa City: AJA Associates.

Benton, A.L., Sivan, A.B., Hamsher, K., Varney, N.R., & Spreen, O. (1994). *Contributions to neuropsychological assessment* (2nd ed.). New York: Oxford University Press.

Bever, C., Grattan, L., Panitch, H.S., & Johnson, K.P. (1995). The Brief Repeatable Battery of Neuropsychological Tests for multiple sclerosis: A preliminary serial study. *Multiple Sclerosis, 1*, 165–169.

Blair, J.R., & Spreen, O. (1989). Predicting premorbid IQ: A revision of the National Adult Reading Test. *Clinical Neuropsychologist, 3*, 129–136.

Boringa, J.B., Lazeron, R.H.C., Reuling, I.E.W., Ader, H.J., Pfennings, L., Lindeboom, J., de Sonneville, L.M., Kalkers, N.F., & Polman, C.H. (2001). The Brief Repeatable Battery of Neuropsychological Tests: Normative values allow application in multiple sclerosis clinical practice. *Multiple Sclerosis, 7*, 263–267.

Botvinick, M., Nystrom, L.E., Fissell, K., Carter, C.S., & Cohen, J.D. (1999). Conflict monitoring versus selection-for-action in anterior cingulate cortex. *Nature, 402*, 179–181.

Braver, T.S., Cohen, J.D., Nystrom, L.E., Jonides, J., Smith, E.E., & Noll, D.C. (1997). A parametric study of prefrontal cortex involvement in human working memory. *Neuroimage, 5*, 49–62.

Camp, S.J., Stevenson, V.L., Thompson, A.J., Miller, D.H., Borras, C., Auriacombe, S., Brochet, B., Falautano, M., Filippi, M., Herisse-Dulo, L., Montalban, X., Parreira, E., Polman, C.H., De Sa, J., & Langdon, D.W. (1999). Cognitive function in primary progressive and transitional progressive multiple sclerosis: A controlled study with MRI correlates. *Brain, 122*, 1341–1348.

Cohen, J.D., Perlstein, W.M., Braver, T.S., Nystrom, L.E., Noll, D.C., Jonides, J., & Smith, E.E. (1997). Temporal dynamics of brain activation during a working memory task [see comments]. *Nature, 386*, 604–608.

Cutter, G.R., Baier, M.L., Rudick, R.A., Cookfair, D.L., Fischer, J.S., Petkau, J., Syndulko, K., Weinshenker, B.G., Antel, J.P., Confavreux, C., Ellison, G.W., Lublin, F., Miller, A.E., Rao, S.M., Reingold, S., Thompson, A., & Wiloughby, E. (1999). Development of a multiple sclerosis functional composite as a clinical trial outcome measure. *Brain, 122*, 871–882.

Delis, D.C., Kaplan, E., & Kramer, J.H. (2001). *California Sorting Test*. Psychological Corporation.

Delis, D.C., Kramer, J.H., Kaplan, E., & Ober, B.A. (1987). *California Verbal Learning Test: Adult version*. San Antonio: Psychological Corporation.

Delis, D.C., Kramer, J.H., Kaplan, E., & Ober, B.A. (2000). *California Verbal Learning Test manual: Second edition, Adult version*. San Antonio, TX: Psychological Corporation.

DeLuca, J., Johnson, S.K., & Natelson, B.H. (1993). Information processing efficiency in chronic fatigue

syndrome and multiple sclerosis. *Archives of Neurology*, *50*, 301–304.

Demaree, H.A., DeLuca, J., Gaudino, E.A., & Diamond, B.J. (1999). Speed of information processing as a key deficit in multiple sclerosis: Implications for rehabilitation. *Journal of Neurology, Neurosurgery, and Psychiatry*, *67*, 661–663.

D'Esposito, M., Onishi, K., Thompson, H., Robinson, K., Armstrong, C., & Grossman, M. (1996). Working memory impairments in multiple sclerosis: Evidence from a dual-task paradigm. *Neuropsychology*, *10*, 51–56.

Fischer, J.S. (1988). Using the Wechsler Memory Scale – Revised to detect and characterize memory deficits in multiple sclerosis. *Clinical Neuropsychologist*, *2*, 149–172.

Fischer, J.S. (1989). Objective memory testing in multiple sclerosis. In K. Jensen, L. Knudsen, E. Stenager, & I. Grant (Eds.), *Mental disorders and cognitive deficits in multiple sclerosis* (pp. 39–49). London: John Libbey.

Fischer, J.S. (1999). Assessment of neuropsychological function. In R.A. Rudick & D.E. Goodkin (Eds.), *Multiple sclerosis therapeutics* (pp. 31–47). London: Martin Dunitz.

Fischer, J.S. (2001). Cognitive impairment in multiple sclerosis. In S.D. Cook (Ed.), *Handbook of multiple sclerosis* (pp. 233–255). New York: Marcel Dekker.

Fischer, J.S., Foley, F.W., Aikens, J.E., Ericson, G.D., Rao, S.M., & Shindell, S. (1994). What do we really know about cognitive dysfunction, affective disorders, and stress in multiple slcerosis? A practitioner's guide. *Journal of Neurologic Rehabilitation*, *8*, 151–164.

Fischer, J.S., LaRocca, N.G., Miller, D.M., Ritvo, P.G., Andrews, H., & Paty, D. (1999). Recent developments in the assessment of quality of life in multiple sclerosis (MS). *Multiple Sclerosis*, *5*, 251–259.

Fischer, J.S., Priore, R.L., Jacobs, L.D., Cookfair, D.L., Rudick, R.A., Herndon, R.M., Richert, J.R., Salazar, A.M., Goodkin, D.E., Granger, C.V., Simon, J.H., Grafman, J.H., Lezak, M.D., O'Reilly Hovey, K.M., Perkins, K.K., Barilla-Clark, D., Schacter, M., Shucard, D.W., Davidson, A.L., Wende, K.E., Bourdette, D.N., & Kooijmans-Coutinho, M.F. (2000). Neuropsychological effects of interferon beta-1a in relapsing multiple sclerosis. Multiple Sclerosis Collaborative Research Group . *Annals of Neurology*, *48*, 885–892.

Fischer, J.S., Rudick, R.A., Cutter, G.R., & Reingold, S.C. (1999). The Multiple Sclerosis Functional Composite Measure (MSFC): An integrated approach to MS clinical outcome assessment. National MS Society Clinical Outcomes Assessment Task Force. *Multiple Sclerosis*, *5*, 244–250.

Fisk, J.D., & Archibald, C.J. (2001). Limitations of the Paced Auditory Serial Addition Test as a measure of working memory in patients with multiple sclerosis. *Journal of the International Neuropsychological Society*, *7*, 363–372.

Fisk, J.D., Pontefract, A., Ritvo, P.G., Archibald, C.J., & Murray, R.J. (1994). The impact of fatigue on patients with multiple sclerosis. *Canadian Journal of Neurological Science*, *21*, 9–14.

Foong, J., Rozewicz, L., Davie, C.A., Thompson, A.J., Miller, D.H., & Ron, M.A. (1999). Correlates of executive function in multiple sclerosis: The use of magnetic resonance spectroscopy as an index of focal pathology. *Journal of Neuropsychiatry and Clinical Neurosciences*, *11*, 45–50.

Foong, J., Rozewicz, L., Quaghebeur, G., Davie, C.A., Kartsounis, L.D., Thompson, A.J., Miller, D.H., & Ron, M.A. (1997). Executive function in multiple sclerosis. The role of frontal lobe pathology. *Brain*, *120*, 15–26.

Franklin, G.M., Heaton, R.K., Nelson, L.M., Filley, C.M., & Seibert, C. (1988). Correlation of neuropsychological and MRI findings in chronic/progressive multiple sclerosis. *Neurology*, *38*, 1826–1829.

Friend, K.B., & Grattan, L. (2000). Use of the North American Adult Reading Test to estimate premorbid intellectual function in patients with multiple sclerosis. *Journal of Clinical and Experimental Neuropsychology*, *20*, 846–851.

Grant, I., McDonald, W.I., Trimble, M.R., Smith, E., & Reed, R. (1984). Deficient learning and memory in early and middle phases of multiple sclerosis. *Journal of Neurology, Neurosurgery, and Psychiatry*, *47*, 250–255.

Gronwall, D.M.A. (1977). Paced auditory serial addition task: A measure of recovery from concussion. *Perceptual and Motor Skills*, *44*, 367–373.

Heaton, R.K. (1985). Neuropsychological findings in relapsing-remitting and chronic-progressive multiple sclerosis. *Journal of Consulting and Clinical Psychology*, *53*, 103–110.

Higginson, C.I., Arnett, P.A., & Voss, W.D. (2000). The ecological validity of clinical tests of memory and attention in multiple sclerosis. *Archives of Clinical Neuropsychology*, *15*, 185–204.

Johnson, S.K., Lange, G., DeLuca, J., Korn, L.R., & Natelson, B.H. (1997). The effects of fatigue on neuropsychological performance in patients with chronic fatigue syndrome, multiple sclerosis, and depression. *Applied Neuropsychology*, *4*, 145–153.

Kent, R.D., Kent, J.F., & Rosenbek, J.C. (1987). Maximum performance tests of speech production. *Journal of Speech and Hearing Disorders*, *52*, 367–387.

Krupp, L.B. (2000). Fatigue and declines in cognitive functioning in multiple sclerosis. *Neurology, 55*, 934–939.

Kujala, P. (1994). Automatic and controlled information processing in multiple sclerosis. *Brain, 117*, 1115–1126.

Langdon, D.W., & Thompson, A.J. (1999). Multiple sclerosis: A preliminary study of selected variables affecting rehabilitation outcome. *Multiple Sclerosis, 5*, 94–100.

Lezak, M. (1995). *Neuropsychological assessment* (2nd ed.). New York: Oxford University Press.

Litvan, I., Grafman, J., Vendrell, P., & Martinez, J. (1988a). Multiple memory deficits in patients with multiple sclerosis: Exploring the working memory system. *Archives of Neurology, 45*, 607–610.

Litvan, I., Grafman, J., Vendrell, P., & Martinez, J. (1988b). Slowed information processing in multiple sclerosis. *Archives of Neurology, 45*, 281–285.

McDonald, W.I., Compston, A., Edan, G., Goodkin, D.E., Hartung, H., Lublin, F., McFarland, H.F., Paty, D.W., Polman, C.H., Reingold, S.C., Sandberg-Wollheim, M., Sibley, W.A., Thompson, A., van der Noort, S., Weinshenker, B.Y., & Wolinsky, J.S. (2001). Recommended diagnostic criteria for multiple sclerosis: Guidelines from the international panel on the diagnosis of multiple sclerosis. *Annals of Neurology, 50*, 121–127.

Minden, S.L. (1990). Memory impairment in multiple sclerosis. *Journal of Clinical and Experimental Neuropsychology, 12*, 566–586.

Mohr, D.C., & Goodkin, D.E. (1999). Treatment of depression in multiple sclerosis: Review and meta-analysis. *Clinical Psychology: Science and Practice, 6*, 1–9.

National Multiple Sclerosis Society Cognitive Functions Study Group. (1990). *A manual for the Brief Repeatable Battery of Neuropsychological Tests in MS*. New York: National Multiple Sclerosis Society.

Nyenhuis, D.L., Rao, S.M., Zajecka, J.M., Luchetta, T., Bernardin, L., & Garron, D.C. (1995). Mood disturbance versus other symptoms of depression in multiple sclerosis. *Journal of the International Neuropsychological Society, 1*, 291–296.

Peyser, J.M., Edwards, K.R., Poser, C.M., & Filskov, S.B. (1980). Cognitive function in patients with multiple sclerosis. *Archives of Neurology, 37*, 577–579.

Peyser, J.M., Rao, S.M., LaRocca, N.G., & Kaplan, E. (1990). Guidelines for neuropsychological research in multiple sclerosis. *Archives of Neurology, 47*, 94–97.

Poser, C.M., Paty, D.W., Scheinberg, L., McDonald, W.I., Davis, F.A., Ebers, G.C., Johnson, K.P., Sibley, W.A., Silberberg, D.H., & Tourtellotte, W.W. (1983). New diagnostic criteria for multiple sclerosis: Guidelines for research protocols. *Annals of Neurology, 13*, 655–661.

Pozzilli, C., Passafiume, D., Bernardi, S., Pantano, P., Incoccia, C., Bastianello, S., Bozzao, L., Lenzi, G.L., & Fieschi, C. (1991). SPECT, MRI and cognitive functions in multiple sclerosis. *Journal of Neurology, Neurosurgery, and Psychiatry, 54*, 110–115.

Rao, S.M. (1989). On the nature of memory disturbance in multiple sclerosis. *Journal of Clinical and Experimental Neuropsychology, 11*, 699–712.

Rao, S.M., Hammeke, T.A., McQuillen, M.P., Khatri, B.O., & Lloyd, D. (1984). Memory disturbance in chronic progressive multiple sclerosis. *Archives of Neurology, 41*, 625–631.

Rao, S.M., Leo, G.J., Bernardin, L., & Unverzagt, F. (1991). Cognitive dysfunction in multiple sclerosis I. Frequency, patterns, and prediction. *Neurology, 41*, 685–691.

Rao, S.M., Leo, G.J., Ellington, L., Nauertz, T., Bernardin, L., & Unveragt, F. (1991). Cognitive dysfunction in multiple sclerosis II. Impact on employment and social functioning. *Neurology, 41*, 692–696.

Rao, S.M., Leo, G.J., Haughton, V.M., Aubin-Faubert, P.S., & Bernardin, L. (1989). Correlation of magnetic resonance imaging with neuropsychological testing in multiple sclerosis. *Neurology, 39*, 161–166.

Rudick, R., Antel, J., Confavreux, C., et al. (1996). Clinical outcomes assessment in multiple sclerosis. *Annals of Neurology, 40*, 469–479.

Ryan, L., Clark, C.M., Klonoff, H., Li, D., & Paty, D. (1996). Patterns of cognitive impairment in relapsing-remitting multiple sclerosis and their relationship to neuropathology on magnetic resonance images. *Neuropsychology, 10*, 176–193.

Salthouse, T.A. (1992). Influence of processing speed on adult age differences in working memory. *Acta Psychologica, 79*, 155–170.

Salthouse, T.A., Babcock, R.L., & Shaw, R.J. (1991). Effects of adult age on structural and operational capacities in working memory. *Psychology and Aging, 6*, 118–127.

Scarrabelotti, M., & Carroll, M. (1998). Awareness of remembering achieved through automatic and conscious processes in multiple sclerosis. *Brain and Cognition, 38*, 183–201.

Smith, A. (1982). *Symbol Digit Modalities Test: Manual*. Los Angeles: Western Psychological Services.

Smith, E.E., Jonides, J., & Koeppe, R.A. (1996). Dissociating verbal and spatial working memory using PET [published erratum appears in Cereb Cortex 1998 Dec;8(8):262]. *Cerebral Cortex, 6*, 11–20.

Spreen, O., & Strauss, E. (1998). *A compendium of neuropsychological tests: Administration, norms, and commentary* (2nd ed.). New York: Oxford University Press.

Troster, A.I., Fields, J.A., Testa, J.A., Blanco, C.R., Hames, K.A., Salmon, D.P., & Beatty, W.W. (1998). Cortical and subcortical influences on clustering and switching in the performance of verbal fluency tasks. *Neuropsychologia*, *36*, 295–304.

Troyer, A.K. (2000). Normative data for clustering and switching on verbal fluency tasks. *Journal of Clinical and Experimental Neuropsychology*, *22*, 370–378.

Troyer, A.K., Graves, R.E., & Cullum, C.M. (1994). Executive functioning as a mediator of the relationship between age and episodic memory in healthy aging. *Aging Neuropsychology and Cognition*, *1*, 45–53.

Van den Burg, W., Van Zomeron, A.H., Minderhoud, J.M., Prange, A.J.A., & Meijer, N.S.A. (1987). Cognitive impairment in patients with multiple sclerosis and mild physical disability. *Archives of Neurology*, *44*, 494–501.

Vleugels, L., Lafosse, C., van Nunen, A., Nachtergaele, S., Ketelaer, P., Charlier, M., & Vandenbussche, E. (2000). Visuoperceptual impairment in multiple sclerosis patients diagnosed with neuropsychological tasks. *Multiple Sclerosis*, *6*, 241–254.

Wechsler, D. (1997). *Wechsler Adult Intelligence Scale* (3rd ed.). San Antonio: Psychological Corporation.

Wilkinson, G.S. (1993). *Wide Range Achievement Test administration manual: 1993 edition*. Wilmington: Wide Range.

Wishart, H., Saykin, A.J., Mamourian, A., Flashman, L.A., Ford, J.C., Fadul, C., Ryan, K., & Kasper, L. (2001). Working memory in multiple sclerosis: Relation of fMRI activation to lesion volume. *NeuroImage*, *13*, S762–S762.

Woodard, J.L., Benedict, R.H.B., Roberts, V.J., Goldstein, F.C., Kinner, K.M., Capruso, D.X., & Clark, A.N. (1996). The reliability and validity of alternate short forms for the Benton Judgement of Line Orientation Test. *Journal of Clinical and Experimental Neuropsychology*, *18*, 898–904.

Woodard, J.L., Benedict, R.H.B., Salthouse, T.A., Toth, J.P., Zgaljardic, D.J., & Hancock, H.E. (1998). Normative data for Odd and Even Parallel Forms of the Judgement of Line Orientation Test. *Journal of Clinical and Experimental Neuropsychology*, *20*, 457–462.

Appendix 9

Patient Reported Outcomes:

Health-related Quality of Life and Resource Use

Hospital Anxiety and Depression Scale

Content

1. Introduction 2
2. Functional assessment of multiple sclerosis (FAMS) 5
3. EQ-5D (EuroQoL)....................6
4. Multiple Sclerosis Standard Health Care Survey 8
5. Hospital Anxiety and Depression Scale 10
6. Abbreviations 13
7. Literature 14

1. Introduction

General

Collection of data for quality of life (QoL) and patient well-being is gaining increased importance in clinical trials serving as additional endpoints to traditional clinical outcomes. This is represented by a shift from "patient-based assessment" to "Patient Reported Outcomes" (PRO). In PRO, the patient is no longer casted in a passive, observer role, but is the primary source of information. The concept of health-related quality of life (HRQoL) refers to the level of well-being and satisfaction associated with an individual's life and how this is affected by disease and treatment. A broad consensus has emerged that at least four dimensions should be included in an HRQoL assessment:

1. Physical health,
2. Functional health,
3. Psychological health,
4. Social health.

In general, there are different instruments that are used to measure patient-reported HRQoL in clinical trials and clinical practice:

- Generic and utility-based generic instruments

  Examples: 36-item Short Form Health Survey Questionnaire (SF-36)[1]
  Sickness Impact Profile (SIP)[2]
  EuroQoL EQ-5D (utility-based)[3]

- Disease-specific instruments

  Examples: Multiple Sclerosis Quality of Life MSQoL-54[4]
  Functional Assessment of Multiple Sclerosis (FAMS)[5]

Generic instruments enable comparisons with other conditions and to the general population; disease-specific instruments are generally more sensitive to changes from interventions or disease progression, however such instruments may not be available for all conditions.[6] Often two or more questionnaires are used together as 'test-battery' to address different aspects of well-being and functioning.

The evaluation of HRQoL in patients undergoing treatment may add aspects of effectiveness usually not covered by clinical outcomes. HRQoL data are highly relevant in patients with chronic diseases with high mortality and morbidity, since one goal of treatment is to enhance well-being and to maximize function. Depending on the method used for assessment, this data can also be used for economic evaluations, e.g., utility-based HRQoL instruments for cost-utility analyses. These analyses are often used as a basis of decision making in health care resource allocation.

Patient Reported Outcomes in MS

There are very few studies on the impact of treatment or rehabilitation on HRQoL in MS[7,8]. Cross-sectional observation studies[9,10,11] using generic questionnaires have established evidence that the HRQoL of patients with MS is much impaired compared to the general population and that HRQoL markedly deteriorates as the disease progresses. Impairment is mainly seen in the physical domain; treatment or rehabilitation also mainly have a beneficial effect on this domain. There are no published data using a disease-specific questionnaire in a large randomized clinical trial.

In the underlying study, HRQoL is an effectiveness-related variable. The main objective of the outcomes research part within this study is to obtain exploratory data on HRQoL in the treated MS patients in the long run (up to 16 years), and to obtain utility data from these patients. The data may also serve as a basis for possible subsequent evaluations.

The cost of MS has been subject of a number of (observational) studies in different countries; the main objectives were to estimate the economic burden for society as a whole and on different payers as well as to generate cost data for cost-effectiveness analyses. The general findings of all studies are

1. Costs dramatically increase as patients progress to higher levels of disability,
2. Indirect cost (lost productivity) is the dominant overall cost driver,
3. In-patient care is the dominant cost driver within direct costs.

Another significant finding is that a large proportion of the economic burden is not incurred within the health care system, but borne by social systems, communities or patients and their families.

However, the current knowledge on the impact of new and existing treatments on the economic burden of MS in the long run is very limited. The objective of the resource use collection within the underlying study hence is to collect detailed cost data using a patient questionnaire in treated MS patients in the long run (up to 16 years). The data may also serve as a basis for possible subsequent evaluations. This questionnaire[12] was used in international (Germany, Sweden, United Kingdom, USA) cost-of-illness studies in more than 3,700 patients with MS[13,14,15,16]. The natural units of resource use collected through this questionnaire (e.g. number of consultations with a physiotherapist) will be multiplied by their unit cost, i.e. the tariff or opportunity cost. Based on these highly relevant data, an epidemiologic model for patients suffering from MS could be further developed[17 18].

Data collection

The instruments used for outcomes research in this study will be

- Functional Assessment in Multiple Sclerosis (FAMS)
- EuroQoL (EQ-5D) for assessment of QoL
- Multiple Sclerosis Standard Health Care Survey (MS-SHCS) for resource use.

All instruments will be self-administered by the patient. I.e., the data collected will result in patient-reported outcomes (PRO), using the patient as the exclusive source of information.

For all instruments, native language versions will be used. QoL questionnaires should be filled out when the patients come to the trial site for a scheduled visit before any test or physical examination in order to avoid bias from these findings. The questionnaire will be handed out to the patients by a study nurse. It needs to be ensured that patients are able to complete the questionnaire. Patients need sufficient time and privacy to complete the questionnaire at their regular appointments. At least at baseline, the questionnaires will be explained to the patient. Assistance will be provided in patients who have difficulty with reading. The patients will be asked to fill out the questionnaires as completely and accurately as possible. It needs to be checked by the study nurse afterwards that for each question only one box has been ticked, and that every question is answered.

The patient is responsible for the completion of the questionnaire; i.e., others, e.g. the accompanying person, should not complete the questionnaire for him/her. Family members in particular should not help the patient with completion of the questions as the burden on family members is known to be a source of bias for self-completed questionnaires.

The average time to complete the questionnaires is approximately 15 minutes.

In patients who are unable to read, an interview-administered version should be used (to be recorded if questionnaire was done as an interview); the interview should then preferably be done by the study nurse. NB: Interviews and posted questionnaires may give different results.

Data analysis and reporting

Details on the variables and their statistical analysis will be provided in the CORE Statistical Analysis Plan which will be finalized and signed off before data release. This analysis plan will be appended to the general Statistical Analysis Plan of the study.

2. Functional assessment of multiple sclerosis (FAMS)

The FAMS QoL instrument is a standardized, independent and widely accepted HRQoL questionnaire for use in patients diagnosed with MS. The response period is the past 7 days. The questionnaire is a self-report multidimensional index of HRQoL comprising a total of 44 items on six subscales:

1.	Mobility	7 items	
2.	Symptoms	7 items	
3.	Emotional well-being	7 items	
4.	General contentment	7 items	
5.	Thinking and fatigue	9 items	
6.	Family / social well-being	7 items	
7.	Additional concerns	14 items	(not scored)

Each item is rated on a 5-point scale (0-4); The 14 items for ‘additional concerns’ are currently not scored.

For most items, a higher score reflects a higher QoL. However, for reasons of balancing psychometric properties over items, some items are reversed, i.e. a score of 0 indicates a higher QoL; these items must be reversed by subtracting the response from 4.

If there are missing data, subscale scores can be prorated, provided that at least 50% of the items (of the respective subscale) were answered. The following formula is used:

$$\text{prorated subscale score} = \frac{\text{(sum of item scores) x (n of items in subscale)}}{\text{(n of items answered)}}$$

The sum of the 44 scored items is the total score, thus ranging from 0 to 176, with higher numbers reflecting a higher QoL. This total score is the primary criterion by which the effect of treatment on QoL will be assessed. The totals based on each subscale will be secondary criteria of QoL.

For this study, version 4 of FAMS will be used; the 14 items for “additional concerns” are currently not scored.

The measure has been validated according to state-of-the-art measurement standards. The FAMS is considered to be an acceptable indicator of the patients’ QoL, provided the following requirements regarding the proportion of items answered are fulfilled:

- ≥ 50% of items on each subscale
- ≥ 80% of total items.

If the above requirements are not fulfilled, the QoL assessment for that patient at that evaluation visit will be considered as 'missing'.

3. EQ-5D (EuroQoL)

The EQ-5D is a multidimensional measure of HRQoL, capable of being expressed as a single index value within the range of 0 to 100. The response period is the day of assessment only.

The EQ-5D has been used in large observation studies on the QoL of patients with MS to generate utilities for cost-effectiveness analyses[13,14,15,19]. It has been validated in multiple languages and can therefore be used in a multinational trial.

Assessments can be done by using a rating scale (Health State Classification) and a visual analogue scale (VAS). In this study, both methods will be used.

Rating scale

The Health State Classification comprises five dimensions selected from literature and from existing QoL measures. For each dimension, the patient is asked to indicate his/her current health state by ticking the most appropriate of three statements about each of the dimensions. Each statement has an increasing degree of severity, e.g. 'no problems' (= 1), 'some problems'(= 2), 'extreme problems'(= 3). The classification therefore defines a total of (3^5 =) 243 theoretically possible health states plus 'dead' and 'unconscious', ranging from '11111' to '33333'. An overview is given below:

Dimension	State 1	State 2	State 3
**Mobility**	*I have no problems in walking about.*	*I have some problems in walking about.*	*I am confined to bed.*
**Self-care**	*I have no problems with self–care.*	*I have some problems washing or dressing myself.*	*I am unable to wash or dress myself.*
**Usual activities** (e.g. work, study, housework, family or leisure activities)	*I have no problems with performing my usual activities.*	*I have some problems with performing my usual activities.*	*I am unable to perform my usual activities.*
**Pain / discomfort**	*I have no pain or discomfort.*	*I have moderate pain or discomfort.*	*I have extreme pain or discomfort.*
**Anxiety / depression**	*I am not anxious or depressed.*	*I am moderately anxious or depressed.*	*I am extremely anxious or depressed.*

Based on large population surveys, an algorithm had been developed to combine the recordings for each of these five dimensions into one single HRQoL score, ranging from +1 ('11111', best

condition) to –0.59 ('33333', worst condition)[20]. The algorithm is provided below. For ease of use, the corresponding score for each of the 243 theoretically possible health states can be read off a table provided for analysis.

Two examples are given below:

	Example: State '22311'	Example: State '22231'
**Mobility**	*I have some problems in walking about.*	*I have some problems in walking about.*
**Self-care**	*I have some problems washing or dressing myself.*	*I have some problems washing or dressing myself.*
**Usual activities**	*I am unable to perform my usual activities.*	*I have some problems with performing my usual activities.*
**Pain / discomfort**	*I have no pain or discomfort.*	*I have extreme pain or discomfort.*
**Anxiety / depression**	*I am not anxious or depressed.*	*I am not anxious or depressed.*
**HRQoL Score**	**0.38**	**0.06**

Valuations for each of the EuroQoL health states (utilities) are available for many countries using time trade-off methodology; utilities are used in economic evaluations to calculate Quality-adjusted life years (QALYs).). A QALY links the lifetime (in years) to the quality of life (expressed as a benefit).

EQ-5D Visual analogue scale

The EQ-5D VAS ('EQ-5D Thermometer', standard height 20 cm), generates a self-rating of current HRQoL. It has endpoints of 100 (best imaginable health state) at the top, and 0 (worst imaginable health state) at the bottom. The respondent rates his/her current health state by drawing a line from the box marked 'your health state today' to the appropriate point on the EQ-5D VAS. A three-digit number (including leading zeros) is read off the scale from the point where the respondent's line crosses the scale.

Missing values will be coded as 996 = 'not done', ambiguous responses (e.g. marking more than one dimensional level or more than one point on the scale) will be coded as 995 = 'not assessable'.

Algorithm for the calculation of the EQ-5D HRQoL score

The records for the five dimensions of the EQ-5D (EuroQoL) as given in Section 3 are to be combined into the HRQoL score using an algorithm summarized below [20].

Step 1: Take the value 1.0 (equivalent to full health '11111').

Step 2: Subtract 0.081 if the state is different from '11111'.

Step 3: Subtract for each dimension the appropriate value for Level 2 or Level 3 as given in the table below (no subtraction for Level 1).

EuroQol Dimension	Level 2	Level 3
Mobility	0.069	0.314
Self-care	0.104	0.214
Usual activities	0.036	0.094
Pain / discomfort	0.123	0.386
Anxiety / depression	0.071	0.236

Step 4: Subtract 0.269 if any dimension has a record of Level 3.

Thus, the HRQoL score can range between +1 ('11111', best condition) and –0.59 ('33333', worst condition).

Example: The HRQoL score for the state '11223' is given by

Step 1	*Step 2*	*Step 3*	*Step 4*		***HRQoL score***
1.0	minus 0.081	minus 0.036<br>minus 0.123<br>minus 0.236	minus 0.269	→	**0.255**

4. Multiple Sclerosis Standard Health Care Survey

Resource use will be assessed by a resource-use questionnaire for longitudinal evaluation of the economic impact of the disease, the Multiple Sclerosis Standard Health Care Survey (MS-SHCS). The questionnaire will be completed by the patient. In case there are discrepancies between important data such as age, level of EDSS (expanded disability status scale), concomitant medication, or hospitalizations compared to the data documented by the physician in the CRF, the data from the CRF will be used for statistical analysis if issue cannot be resolved otherwise.

The questionnaire includes:

- Hospitalizations (past 12 months)
- Outpatient visits to physicians, nurses, physiotherapists, psychologists, incontinence advisors, optician, social worker, and other health care professionals (past 3 months)
- Drugs and (alternative) procedures (past 3 months)
- Community and other services (home help, child care, meals on wheels, etc; past 3 months)
- Investments (house adaptations, devices, etc; past 12 months)
- Employment situation and related questions (past 3 months)

- Health care system specific questions like insurance coverage etc.
- Disease questions

For health care resources and community services, patients will be asked for their consumption during the past 3 months as they occur more frequent and a time window of maximum 3 months has shown to be reliable in retrospective data collection. Large investments such as transformations to the house etc. and change in work status – events which occur less frequently - will be related to the past 6 months. Patients will be reminded at each question what the relevant time frame to consider is.

5. Hospital Anxiety and Depression Scale

The HADS[21] does not belong to the HRQoL and resource-use questionnaires but can be classified as a PRO and is therefore included in this appendix. The HADS is applied in this study as a qualifying instrument to investigate potentially confounding factors for the test performance of patients during the neuropsychological testing. Depression is common in MS patients and can adversely affect the test performance, particularly on capacity-demanding tasks. Mood disturbances can also affect the accuracy of patients' perception of their performance.

The HADS is a 14-item scale designed to detect anxiety and depression in general medical out-patient populations. It is also intended to measure the severity of emotional disorder. Despite the use of the word 'hospital' in the title it is valid in community work. It consists of seven depression items and seven anxiety items. These were selected to distinguish the effects of physical illness from mood disorders, and so symptoms likely to be present in both (e.g. dizziness and headaches) were not included. Care was taken to distinguish between the symptoms of anxiety and depression.

As the HAD Scale was designed for use in general hospital outpatient departments it can be completed in a short space of time. To this end, the instructions encourage the client to underline their immediate reaction to each item, rather than attempting a long thought-out response. The possible responses are set out in terms of the frequency or intensity of the client's experience. The client is instructed to complete the scale in order to reflect his or her state in the past week.

All 14 items in the HAD Scale are rated on a four-point scale, ranging from the absence of a symptom or the presence of positive features (scoring 0) to maximal symptomatology or the absence of positive features, which score 3. Therefore, the higher the score the more severe the disorder. Examples of symptoms are. 'I feel tense or wound up' (score 3 for 'most of the time' and 0 for 'not at atl') and 'I have lost interest in my appearance' (score 3 for 'definitely' and 0 for 'I take as much care as ever'). Positive features include items concerning enjoyment, sense of humor and feeling cheerful. The maximum for each subscale is 21 (7 items x maximum 3 score).

Interpretation

The table below provides a summary of the data Zigmond and Snaith (1983) gathered on 100 clients. This indicates that scores of less than eight on either subscale are likely to come from clients with no clinically significant anxiety or depression, as judged by psychiatric interview (i.e. ‘non-cases’). Scores between 8 and 10 are likely to be obtained by mildly disturbed clients (‘doubtful cases‘); whereas scores between 1 and 21 are likely to indicate definite anxiety and depression (i.e. ‘cases, or clients whose condition merits psychiatric assessment).

Numbers of patients in each category according to subscale scores on the HADS

Depression				Anxiety			
Scale scores	*Non-cases*	*Doubtful cases*	*Cases*	*Scale scores*	*Non-cases*	*Doubtful cases*	*Cases*
0–7	57	11	1	0–7	41	4	1
8–10	8	7	3	8–10	10	9	1
11–21	1	4	8	11–21	5	15	14

Psychometric status

The HADS face validity is good, clients finding it very acceptable, and easy to complete. The content validity was based on the use of items found to be important in other instruments and from the authors' own research.

Concurrent validation was with the authors' 20-minute interview of clients, blind to HADS results. This yielded significant correlations (0.54 for anxiety and 0.79 for depression). Criterion validity was assessed by considering a subsample of clients judged by interview to be presenting with either anxiety or depression (n=17). As predicted, there were nonsignificant associations between the client's anxiety and depression scores, as measured by the HAD Scale.

Finally, as a measure of internal consistency. Zigmond and Snaith (1983) carried out item-subscale correlations on the replies of 50 clients, finding significant associations of between 0.76 and 0.41 for the anxiety items; and between 0.60 and 0.30 for the depression ones. One weakly correlated item was removed from each subscale bringing the HAD Scale down from 16 to 14 items. No reliability data have been presented.

In summary, the HADs is a valid instrument for assessing anxiety and depression in general hospital out-patients and for estimating severity. The authors suggest that the scale may also be valid for other groups, but should be supplemented by a brief interview.

The HADS is employed in this study as a brief self-report measure and includes the following instructions and questions:

"Clinicians are aware that emotions play an important part in most illnesses. If your clinician knows about these feelings she or he will be able to help you more.

This questionnaire is designed to help your clinician to know how you feel. Ignore the numbers printed on the left of the questionnaire. Read each item and underline the reply which comes closest to how you have been feeling in the past week.

Don't take too long over your replies; your immediate reactioni to each item will probably be more accurate than a long thought-out response.

1. I feel tense or "wound up":
   - Most of the time
   - A lot of the time
   - From time to time, occasionally
   - Not at all

2. I still enjoy the things I used to enjoy:
   - Definitely as much
   - Not quite so much
   - Only a little
   - Hardly at all

3. I get a sort of frightened feeling as if something awful is about to happen:
   - Very definitely and quite badly
   - Yes, but not too badly
   - A little, but it doesn't worry me
   - Not at all

4. I can laugh and see the funny side of things:
   - As much as I always could
   - Not quite so much now
   - Definitely not so much now
   - Not at all

5. Worrying thoughts go through my mind
   - A great deal of the time
   - A lot of the time
   - From time to time but not too often
   - Only occasionally

6. I feel cheerful:
   - Not at all
   - Not often
   - Sometimes
   - Most of the time

7. I can sit at ease and feel relaxed:
   - Definitely
   - Usually
   - Not often
   - Not at all

8. I feel as if I am slowed down:
   - Nearly all the time
   - Very often
   - Sometimes
   - Not at all

9. I get a sort of frightened feeling like "butterflies" in the stomach:
   - Not at all
   - Occasionally
   - Quite often
   - Very often

10. I have lost interest in my appearance:
    - Definitely
    - I don't take as much care as I should
    - I may not take quite as much care
    - I take just as much care as ever

11. I feel restless as if I have to be on the move:
    - Very much indeed
    - Quite a lot
    - Not very much
    - Not at all

12. I look forward with enjoyment to things:
    - As much as ever I did
    - Rather less than I used to
    - Definitely less than I used to
    - Hardly at all

13. I get sudden feelings of panic:
    - Very often indeed
    - Quite often
    - Not very often
    - Not at all

14. I can enjoy a good book or radio or TV programme:
    - Often
    - Sometimes
    - Not often
    - Very seldom

Now check that you have answered all the questions!

6. Abbreviations

EDSS	Expanded disability status scale
FAMS	Functional assessment of multiple sclerosis
HRQoL	Health-related quality of life
MS	Multiple sclerosis
MSQoL	Multiple sclerosis quality of life
MSQLI	Multiple sclerosis quality of life inventory
MS-SHCS	Multiple sclerosis standard health care survey
PRO	Patient-reported outcomes
QALY	Quality-adjusted life year
QoL	Quality of life
SIP	Sickness impact profile
VAS	Visual analogue scale

7. Literature

1 Ware JE, Sherbourne CD. The MOS 36-item short-form health survey (SF-36). Conceptual framework and item selection. Medical Care 1992; 30: 473-83

2 Bergner M, Bobbit RA, Carter WB et al. The Sickness Impact Profile: Development and final revision of a health status measure. Med. Care 1981; 19: 787-805

3 EuroQoL group. EuroQoL - a new facility for the measurement of health-related quality of life. Health Policy 1990; 16: 199-208

4 Vickery B et al. A health-related quality of life measure for multiple sclerosis. Quality of Life Research 1995; 4: 187-200

5 Cella DF, Dineen K, Arnason B, Reder A, Webster KA, karabatsos G, Chang C, Lloyd S, Steward J, Stefoski D. The validation of the Functional Assessment in Multiple Sclerosis questionnaire. Neurology 1996; 47: 129-139

6 Miltenburger C, and Kobelt G. Quality of life and cost of multiple sclerosis. Clin Neurol Neurosurg 2002;104(3):272-275.

7 Petajan JH et al. Impact of aerobic training on fitness and quality of life in multiple sclerosis. Ann Neurol 1996; 39: 432-441

8 Freeman J et al. Interferon Beta-1b (IFNB-1b) delays sustained deterioration in Health Related Quality of Life (HRQoL) in Secondary Progressive Multiple Sclerosis (SP-MS). Poster PO1.086 American Academy of Neurology 2000, San Diego

9 The Canadian Burden of Illness Study Group. Burden of Illness of Multiples Sclerosis: Part II: Quality of Life. Canadian Journal of Neurological Science 1998; 25: 31-38

10 Henriksson F, Fredrikson S, Masterman T, Jonsson B. Costs, Quality of Life and disease severity in Multiple Sclerosis – a cross-sectional study in Sweden. European Journal of Neurology 2001; 8: 27-35

11 Duff SB, and Mordin MM. Strategies for assuring health economic and quality of life outcomes in multiple sclerosis. Expert Rev. Pharmacoeconomics Outcomes Res 2002; 2(6): 577-587.

12 Kobelt G, Miltenburger C, Kaskel P, Jönsson B. The MS Standard Health Care Survey Questionnaire (SHCS): a patient-reported measure for resource use assessment in multiple sclerosis. Presentation at ECTRIMS Vienna, Abstract 351. Multiple Sclerosis 2004, in press

13 Kobelt G, Lindgren P, Parkin D, Francis D, Johnson M, Bates D, Jönsson B. Costs and Quality of Life in Multiple Sclerosis. A cross-sectional observational study in the UK. Stockholm School of Economics. EFI, The Economic Research Institute. Working Paper No. 398, August 2000

14 The German Cost of MS Study Group. Cost and quality of life in multiple sclerosis. An observational study in Germany. Europ J Health Economics 2001; 2: 60-68

15 Henriksson F, Fredrikson S, Jönsson B. Costs, Quality of Life and disease severity in Multiple Sclerosis – a population-based cross-sectional study in Sweden. Stockholm School of Economics. EFI, The Economic Research Institute. Working Paper No. 361, March 2000

16 Schering AG, Berlin, Germany, data on file

17 Kobelt G, Jönsson L, Miltenburger C, and Jönsson B. Cost-utility analysis of interferon beta-1b in secondary progressive multiple sclerosis using natural history disease data. Int J Technol Health Assess Health Care 2002;18(1):127-138.

[18] Kobelt G, Jönsson L, Fredrikson S. Cost-utility of interferon beta-1b in the treatment of patients with active relapsing-remitting or secondary progressive multiple sclerosis. Eur J Health Economics 2003; 4: 50-59

[19] Kobelt G, Lindgren P, Smala A, Jönsson B, and the German Cost of MS Study Group. Costs and Quality of Life in Multiple Sclerosis. A cross-sectional observational study in Germany. Stockholm School of Economics. EFI, The Economic Research Institute Working Paper No. 399, August 2000

[20] Dolan P. Modeling valuations for EuroQol health states. Med Care 1997; 35(11): 1095-108

[21] Zigmond P, Snaith S. The Hospital Anxiety and Depression Scale. Acta Psychiatr Scand 1983;13:141-7.

Appendix 10

Guidelines for Kurtzke's Functional Systems and Expanded Disability Status Scale (EDSS)

Content

1. Overview 2

2. Functional systems: scoring aids 3
   2.1 Optical functions 3
   2.2 Brainstem functions 3
   2.3 Pyramidal functions 4
   2.4 Cerebellar functions 5
   2.5 Sensory functions 6
   2.6 Vegetative disturbance / bowel and bladder function 6
   2.7 Cerebral functions 7

3. Expanded disability status scale (EDSS)..8

Overview

The EDSS assessment is based on the standardized neurological examination according to Kurtzke's functional systems and the standard measure of neurological status in MS (Kurtzke 1983). The neurological examination covers the following functional systems:

- Visual (optic) functions
- Brain stem functions / cranial examination
- Pyramidal functions
- Cerebellar functions
- Sensory functions
- Vegetative (bladder and bowel) functions
- Cerebral functions / mental status examination
- Ambulation (walking ability).

Each functional system is represented by a number of items; for each item, the level of impairment will be scored. In addition, for each functional system (except ambulation), the Kurtzke Functional System Score ranging from grade 0 (normal) to grade 5 or grade 6 will be recorded.

Integrating the assessments of all functional systems, the EDSS score will be determined, ranging from 0.0 (normal) to 10.0 (dead).

These neurological examinations will be performed by the examining physician; only at screening this may be carried out by the treating physician.

Functional systems: scoring aids

1.1 Optical functions

Visual acuity

The visual acuity score is based upon the line on the Snellen chart at 20 feet (6.1 m) for which the patient makes no more than one error (use best available correction).

Fields

signs only	Deficits present only on formal testing
moderate	Patient aware of deficit, but incomplete hemianopsia on examination
marked	Complete homonymous hemianopsia or equivalent

Scotoma

small	Detectable only on formal (confrontational) testing
large	Spontaneously reported by patient

1.2 Brainstem functions

Impairment / disability

mild	Clinically detectable numbness, facial weakness, dysarthria or cranial nerve deficits of which patient is aware
moderate	Diplopia with incomplete paralysis of any eye movement, impaired discrimination of sharp/dull in 1 or 2 trigeminal branches, trigeminal neuralgia, weakness of eye closure, cannot hear finger rub and/or misses several whispered numbers, obvious dysarthria during ordinary conversation impairing comprehensibility
	Severe complete loss of movement of either eye in one direction, impaired discrimination of sharp/dull or complete loss of sensation in the entire distribution of one or both trigeminal nerves, unilateral or bilateral facial palsy

	with lagophthalmus or difficulty with liquids, sustained difficulty with swallowing, incomprehensible voice

Nystagmus

mild	Patient feels disturbed
moderate	Sustained nystagmus on 30° horizontal or vertical gaze, but not in primary position
severe	Sustained nystagmus in primary position or coarse persistent nystagmus in any direction interfering with visual acuity, complete internuclear ophthalmoplegia with sustained nystagmus of abducting eye, oscillopsia

1.3 Pyramidal functions

Limb strength

The weakest muscle in each group defines the score for that group. Each movement should be tested, but only pathological findings should be noted using the BMRC grades. Use of functional tests like jumping with one foot, walking on toes or heels are recommended in order to assess grades 3-5 BMRC.

BMRC rating scale

0 = No activity
1 = Visible contraction without visible joint movement
2 = Visible movements with elimination of gravity
3 = Movements against gravity possible but impaired
4 = Movements against resistance possible but impaired
5 = Normal strength

Limb spasticity

mild	Barely increased muscular tone after rapid flexion of an extremity
severe	Barely surmountable increased spastic tonus after rapid flexion of an extremity

1.4 Cerebellar functions

The presence of severe gait ataxia alone results in a grade of 3 in the cerebellar FS. If weakness interferes with the testing of ataxia, score the patient's actual performance, but also indicate the possible role of weakness by marking the respective box.

Truncal ataxia

mild	Swaying with eyes closed
moderate	Swaying with eyes open
severe	Unable to sit without assistance

Limb ataxia

mild	Tremor or clumsy movements seen easily, minor interference with function
moderate	Tremor or clumsy movements interfere with function in all spheres
severe	Most functions are very difficult

Gait ataxia

mild	Abnormal balance only on heel or toe walking, or walking along a line
moderate	Abnormal balance on ordinary walking or while seated
severe	Unable to walk more than a few steps or requires support by another person or walking aid because of ataxia

Romberg test

mild	Mild insecurity with eyes closed
moderate	Not stable with eyes closed
severe	Not stable with eyes open

1.5 Sensory functions

Superficial sensation – touch/ pain

mild	Patient is aware of impaired light touch or pain, but able to discriminate sharp/dull
moderate	Impaired discrimination of sharp/dull
severe	No discrimination of sharp/dull and/or unable to feel light touch

Vibration sense

mild	Graded tuning fork 5–7 of 8 (alternatively) detects more than 10 sec. but less than examiner
moderate	Graded tuning fork 1–4 of 8 (alternatively) detects more than 2 sec. but less than 11 sec.
marked	Complete loss of vibration sense

Position sense

mild	1–2 incorrect responses on testing, only distal joints affected
moderate	Misses many movements of fingers or toes, proximal joints affected
marked	No perception of movement

1.6 Vegetative disturbance / bowel and bladder function

Hesitancy / retention

mild	No major impact on lifestyle
moderate	Urine retention, frequent UTI
severe	Requires catheterization

Urgency / incontinence

mild	No major impact on lifestyle
moderate	Rare incontinence, no more than once a week, must wear pads
severe	Frequent incontinence, several times a week up to once daily, must wear

	urinal

Bowel

mild	No incontinence, no major impact on lifestyle, constipation
moderate	Must wear pads or alter lifestyle to be near lavatory
severe	In need of intermittent enemata

1.7 Cerebral functions

Depression / euphoria

present	Patient complains of depression or is considered depressed or euphoric by the investigator or “significant other”.

Decrease in mentation

mild	Difficulties apparent to patient and “significant other” such as impaired ability to follow a rapid course of association and of surveying complex matters, impaired judgement in certain demanding situations, able to handle the daily routine, but no tolerance for additional stressors, intermittently symptomatic to even normal levels of stress, reduced performance, tendency toward negligence due to obliviousness or fatigue. However, not apparent while taking the history or performing the routine neurological examination.
moderate	Definite abnormalities on formal mental status testing, but still oriented to time, place and person
marked	Not oriented in 1 or 2 spheres of time, place or person, marked effect on lifestyle

Fatigue

mild	Not interfering with daily activities
moderate	Interfering but not limiting daily activities
severe	Significantly limiting daily activities

Expanded disability status scale (EDSS)

The results for the individual functional systems will be integrated to form the EDSS score, ranging from 0.0 (normal) to 10.0 (dead) as depicted below.

Score	Explanation (Note: Mental function's grade 1 does not contribute to EDSS-step definitions)
0.0	Normal neurological exam (i.e. grade 0 in all Functional Systems [FS]).
1.0	No disability, minimal signs (i.e. grade 1) on 1 FS.
1.5	No disability, minimal signs (i.e. grade 1) on > 1 FS.
2.0	Minimal disability (i.e. grade 2) in 1 FS, all others grade ≤ 1.
2.5	Minimal disability (i.e. grade 2) in 2 FSs, all others grade ≤ 1.
3.0	- Moderate disability (i.e. grade 3) in 1 FS, all others grade ≤ 1, though fully ambulatory.<br>*or*<br>- Mild disability (i.e. grade 2) in 3 to 4 FSs, all others grade ≤ 1, though fully ambulatory.
3.5	- Fully ambulatory but with moderate disability (i.e. grade 3) in 1 FS and mild disability (i.e. grade 2) in 1 or 2 FSs, all others grade ≤ 1.<br>*or*<br>- Fully ambulatory but with mild disability (i.e. grade 2) in 5 FSs, all others grade ≤ 1.
4.0	Ambulatory without aid or rest for > 500 m, up and about 12 hrs a day despite relatively severe disability consisting of grade 4 in 1 FS (all others grade ≤ 1), or combinations of lesser grades exceeding limits of previous steps.
4.5	Ambulatory without aid or rest for > 300 m, up and about much of the day; characterized by relatively severe disability usually consisting grade 4 in 1 FS or combinations of lesser grades exceeding limits of previous steps.
5.0	Ambulatory without aid or rest for > 200 m (usually grade 5 in 1 FS, others grade ≤ 1; or combinations of lesser grades usually exceeding specifications for step 4.5).
5.5	Ambulatory without aid or rest > 100 m.
6.0	Unilateral assistance (cane or crutch) required to walk at least 100 m with or without resting.
6.5	Constant bilateral assistance (canes or crutches) required to walk at least 20 m without resting.
7.0	Unable to walk 20 m even with aid, essentially restricted to wheelchair; wheels self and transfers alone; up and about in wheelchair some 12 h a day.
7.5	Unable to take more than a few steps; restricted to wheelchair; may need some help in transfer and in wheeling self.
8.0	Essentially restricted to bed or chair or perambulated in wheelchair, but out of bed most of day; retains many self-care functions; generally has effective use of arms.
8.5	Essentially restricted to bed much of the day; has some effective use of arm(s); retains some selfcare functions.
9.0	Helpless bed patient, can communicate and eat.
9.5	Totally helpless bed patient; unable to communicate effectively or eat /swallow.
10.0	Death due to MS.

Notes

Note 1: EDSS steps below 4 refer to patients who are fully ambulatory, and the precise step is defined by the functional systems (FS) score(s).

EDSS steps between 4.0 and 5.0 are defined by both FS-scores and walking range. In general, the worst of both should determine the score.

Steps 5.5-8.0 are exclusively defined by ability to ambulate or use wheelchair.

Note 2: EDSS should not change by 1.0 step unless there is a change in the same direction of at least one step in at least one FS.

Note 3: “signs only” is noted when the examination reveals signs of which the patient is unaware.

Note 4: A score of 1 in the Functional Systems implies that the patient is not aware of the deficit and that the deficit or sign does not interfere with normal daily activities (with the exceptions of optic, vegetative and cerebral functions).

Note 5: In the definitions of EDSS grades 6.0 and 6.5 both a description of assistance required and of the walking range are included.

In general, the distinction of bilateral versus unilateral assistance required to walk overrules the walking range.

However, the following exceptions apply:

- If a patient is able to walk considerably longer than 100 m with two sticks, crutches or braces he is in grade 6.0.
- If a patient is able to walk more than 10 m and less than 100 m with two sticks, crutches or braces he is in grade 6.5.
- If a patient needs assistance by another person (as opposed to one stick, crutch or brace) and/or is not able to walk more than 50 m with one stick, crutch or brace he is in grade 6.5.

Note 6: Usually the EDSS-score cannot be lower than the highest score of the FS, with the following exceptions:

- Visual FS – For calculation of the EDSS the score is to be converted as follows, 6 = 4; 5 = 3; 4 = 3; 3 = 2; 2 = 2; 1 = 1
- Cerebral FS – The presence of depression and/or euphoria alone results in a score of 1, but does not affect the EDSS score.
- Bladder/Bowel FS – For calculation of the EDSS the score is to be converted as follows, 6 = 5; 5 = 4; 4 = 3; 3 = 3; 2 = 2; 1 = 1

Note 7: Symptoms which are not MS-related will not be taken into consideration for assessments.

Appendix 11

Guidelines for the Multiple Sclerosis Functional Composite (MSFC) measure

Content

1. General MSFC-instructions 3
   - 1.1 Standardizing MSFC administration 3
   - 1.2 Testing environment 4
   - 1.3 Establishing rapport 4
   - 1.4 Recording responses 4
   - 1.5 Providing feedback to the patient about his/her performance 5

2. Timed 25-foot walk 5
   - 2.1 Description 5
   - 2.2 Materials needed 5
   - 2.3 Time limit per trial 5
   - 2.4 Discontinuation rules 6
   - 2.5 Administration 6
   - 2.6 Assistive devices 6
   - 2.7 Completing the record form 7
   - 2.8 Questions and answers 7

3. 9-hole peg test (9-HPT) 9
   - 3.1 Description 9
   - 3.2 Materials needed 10
   - 3.3 Time limit per trial 10
   - 3.4 Discontinuation rules 10
   - 3.5 Administration 10
   - 3.6 Completing the record form 12
   - 3.7 Questions and answers 12

4. Paced auditory serial addition test (PASAT) 14
   - 4.1 Description 14
   - 4.2 Discontinuation rules 15
   - 4.3 Administration 15
   - 4.4 Completing the record form 16
   - 4.5 Questions and answers 17

1. General MSFC-instructions

(Taken from the "Administration and Scoring Manual for the Multiple Sclerosis Functional Composite Measure (MSFC)" by Fischer JS, Jak JS, Kniker JE, Rudick RA and Cutter G. 1999.)

The MSFC is based of three different sub-tests assessing upper and lower extremities function as well as cognitive function:

- Timed 25-foot (7.62 m)-walk,
- 9-hole peg-test (9-HPT),
- 3'' paced auditory serial addition test (PASAT).

Thereby, the MSFC is based on the concept that scores for these three dimensions (lower / upper extremities and cognitive function) are combined to create a single score that can be used to detect changes over time. This is done by computing z-scores for each component of the MFSC and averaging them to generate an overall composite score (details of this procedure will be described in the statistical analysis plan).

1.1 Standardizing MSFC administration

The MSFC should be administered as close to the beginning of a study visit as possible, but definitely before the patient does a distance walk.

MSFC components should be administered in the following order:

1. Trial 1, Timed 25-foot walk
2. Trial 2, Timed 25-foot walk
3. Trial 1, Dominant hand, 9-HPT
4. Trial 2, Dominant hand, 9-HPT
5. Trial 1, Non-dominant hand, 9-HPT
6. Trial 2, Non-dominant hand, 9-HPT
7. PASAT

An individual component of the MSFC should be discontinued only if the patient meets the discontinue criteria for that component. Other components should still be administered.

Instructions for each component measure should be given exactly as they appear in the manual "Administration and Scoring Manual for the MSFC". As patients gain more experience with the tasks, many may want to skip the instructions or the PASAT practice trials. To ensure standardized

administration, full instructions should be given for each task, and at least three complete practice trials should be given before the first application.

1.2 Testing environment

Every effort should be made to use the same testing room and the same designated area for the Timed 25-Foot Walk at every visit. It is essential that the potential for external distractions be kept to a minimum. All necessary materials should be assembled prior to the patient's arrival. If the space designated for the Timed 25-Foot Walk is a public hallway, complete privacy may be impossible; however, every attempt should be made to keep the patient's path clear of obstacles (human or inanimate). No one other than the examiner and the patient should be in the testing room during 9-HPT and PASAT. Unplug the phones (or turn off their ringers), and turn off beepers (or switch to non-audible notification). Establish some way to indicate to others that testing is in progress and you are not to be disturbed (*e.g.*, a sign on the door stating "TESTING IN PROGRESS – DO NOT DISTURB").

1.3 Establishing rapport

The examiner should be thoroughly familiar with all of the measures prior to their administration so that s/he can focus only on establishing rapport, observing the patient's behavior, and recording the patient's times and responses. The MSFC can be challenging for patients, so it is important to establish good rapport early on. Offer to answer any questions the patient may have about the measures. Patients may ask things such as, "What will I be doing today?" or "How long will this take?" You may tell the patient things such as, "I'll be testing your upper and lower extremity function as well as cognitive function" or, "You'll be doing three tasks today, which will take about 20 minutes to complete."

1.4 Recording responses

Use a clipboard as your writing surface and hold it in such a way that the patient cannot see what you are writing. Please write legibly. Any deviation from the standard instructions due to examiner error or external interferences should be noted in the appropriate section of each Record Form. In general, any circumstances that may have affected the patient's performance should be recorded.

1.5 Providing feedback to the patient about his/her performance

Do not provide the patient with test answers and do not indicate whether answers are correct or incorrect (either verbally or non-verbally), except on practice items. Do not give the patient direct feedback about his/her performances on any of the measures, *i.e.*, the times on the Timed 25-Foot Walk or 9-HPT or the patient's total correct on PASAT. If the patient asks, "How did I do?", or something to that effect, give general encouragement and/or feedback such as, "You gave great effort today", or, "I thought things ran very smoothly today." If the patient appears distressed upon the completion of a task, it may be helpful to say something similar to, "I can see that this test was very difficult for you. It can be a very challenging task and I appreciate how you gave your full effort." It is important that you make all reasonable attempts to maintain the physical and emotional comfort of the patients as they complete the measures. At the completion of the visit be sure to thank the patient for his/her participation.

2. Timed 25-foot walk

2.1 Description

The timed 25-foot walk is a quantitative measure of lower extremity function. It is the first component of the MSFC administered at each evaluation. The patient is directed to one end of a clearly marked 25-foot (7.62 m) course and is instructed to walk 25 feet (7.62 meter) as quickly as possible, but safely. The task is immediately administered again by having the patient walk back the same distance. Patients may use assistive devices when doing this task. In clinical trials, it is recommended that the study physician selects the appropriate assistive device for each patient.

2.2 Materials needed

Stopwatch, clipboard, timed 25-foot walk record form, marked 25-foot (7.62 m) distance in an unobstructed hallway, assistive device (if needed).

2.3 Time limit per trial

3 minutes (180 seconds) per trial.

2.4 Discontinuation rules

1. If the patient cannot complete Trial 2 of the timed walk after a 5-minute rest period.
2. If the patient cannot complete a trial (one way) in 3 minutes.

2.5 Administration

Trial 1

Make sure that the stopwatch is set to 0:00. For the timed 25-foot walk, the subject should be directed to one end of a clearly marked 25-foot (7.62 m) course (clearly defined on the floor or on the wall) and instructed to stand just behind the starting line. Point out where the 25-foot course ends, then instruct the patient as follows:

> **"I'd like you to walk 25 feet [or 7.62 meters] as quickly as possible, but safely. Do not slow down until after you've passed the finish line. Ready? Go."**

Begin timing when the lead foot is lifted and crosses the starting line. The examiner should walk along with the patient as he/she completes the task. Stop timing when the lead foot crosses the finish line. The examiner should then record the subject's walk time to within 0.1 second, rounding as needed. Round up to the next tenth if hundredth's place is > .05, round down if hundredth's place is < .05 (*e.g.*, 32.45" would round to 32.5" but 32.44" would round to 32.4"). Once the time is recorded, be sure to reset the stopwatch.

Trial 2

After completing the first timed walk, position the patient just behind the line where s/he is now standing, repeat the same instructions, and have the patient complete the walk again.

2.6 Assistive devices

In clinical trials and other serial studies, the goal is to use the same assistive device at each study visit. The treating neurologist should select an assistive device at the beginning of the study for each patient who needs one, keeping in mind that the patient may deteriorate modestly over the course of a trial. In general, patients should use their customary assistive device(s), NOT the least assistance possible to complete the test. For patients with significant gait impairment, the treating neurologist should have the patient use a rolling walker even if this is not the patient's customary device. In general, non-wheeled walkers should not be used. If a patient does use an assistive device, this should be noted on the Record Form.

2.7 Completing the record form

Record any circumstances that you believe may have affected the patient’s performance. These are factors that may have affected the trial but were not severe enough to necessitate repetition of the trial:

- The patient had a cold or reports not feeling well.
- The patient tripped but did not fall.

If a situation arises that necessitates the repetition of a trial, indicate the reason a trial had to be repeated on the Record Form. Examples of reasons to repeat a trial include, but are not limited to, the following:

- The patient fell during the walk.
- Examiner forgot to start or stop stopwatch.
- Examiner forgot to reset stopwatch in between trials.
- The patient stopped to talk to someone while walking, or another person/thing somehow interfered with walk.

Record only the times for the two successfully completed trials of the timed 25-foot walk. If the patient could not complete one or both of the trials of the timed 25-foot walk, record this in the appropriate section of the Record Form. For example, if the patient's disease has progressed and/or physical limitations prohibit him or her from completing the trial, you should indicate "Unable to complete trial due to physical limitations", and record any specifics that you can observe (*i.e.*, patient in a wheelchair now and unable to walk, etc.). If the patient did not complete a trial for any other reason, specify this as well (*e.g.*, patient fell and was too fatigued to complete another trial; patient refused to complete trial).

2.8 Questions and answers

Q. Does it matter what kind of shoes the patient wears?

A. As long as the style of the shoe is consistent for each patient from visit to visit, it does not matter what kind of shoes are worn. Encourage the patient to wear comfortable shoes and discourage patients from wearing, for example, high-heeled shoes one visit and running shoes the next.

Q. Is the patient allowed to pause while walking the 25-foot distance?

A. The patient should be encouraged to walk at a steady pace, one that he or she can sustain for 25 feet (7.62 m). However, pauses are allowed as long as the patient can complete the walk within the 3-minute time limit.

Q. Is the patient allowed to run?

A. No. The patient is to walk quickly but should not run.

Q. Is the patient allowed to wear an ankle-foot orthosis (APO) during the timed 25-foot walk?

A. If the patient typically wears an APO and wears it every time the timed 25-foot walk is performed, the patient may wear his/her APO. Be sure to record this on the Record Form.

Q. What should I do if the patient wants to use the wall as support while walking?

A. The patient is only allowed to use customary assistive devices while walking (*e.g.*, crutch, cane, wheeled walker) and therefore is not allowed to use a wall for continued support during the timed 25-foot walk.

Q. What should I do if the patient asks to lean on me for support while walking?

A. This also is not allowed. Only customary assistive devices should be used if the patient needs extra support while walking.

Q. How many times is the patient allowed to touch the wall or my arm momentarily for support during the walk?

A. The patient is allowed to touch the wall or your arm a maximum of two times. If he or she touches the wall/your arm more than twice, repeat the trial, emphasizing to the patient that he or she is to walk using only the support of an assistive device. If the patient does not use an assistive device, notify the study coordinator or treating physician that the patient may need to be reevaluated for use of an assistive device.

Q. What's the difference between a cane and a crutch?

A. A crutch extends under the axilla or supports the upper arm whereas the patient merely holds onto a cane.

Q. What should I do if the patient drops his or her cane (or other assistive device) while walking?

A. Restart the trial and record the reason on the source document form.

Q. Non-wheeled walkers are not allowed. What should l do if this is the assistive device the patient typically uses?

A. Supply the patient with an accepted assistive device to use for the study visits. If it is a device unfamiliar to the patient, time should be allowed to practice with the device before the timed 25-foot walk is administered.

Q. It is standard practice at my site to use a guard belt during tasks such as the timed 25- foot walk. Is that O.K. for this study?

A. If this is standard practice for your site and all patients will consistently wear the guard belt for all trials of the timed 25-foot walk for every visit, then this is acceptable. The device should neither assist nor encumber the patient.

Q. Is it O.K. if the patient carries his/her purse or coat while he/she is walking?

A. The patient should walk unencumbered. Patients should not be carrying any of their belongings during the timed 25-foot walk. Furthermore, coats, purses, etc. should not be draped over wheeled walkers or other assistive devices while the patient is completing the Timed 25-Foot Walk.

Q. What should I do if the patient falls while s/he is walking?

A. If the patient has not injured him/herself and is able to continue, start the trial over, reading the full instructions again. Emphasize that the patient should walk as quickly as possible, but safely. Record that the trial was repeated on the Record Form.

Q. The patient keeps falling during the walk. How many attempted trials are permitted before I should discontinue and indicate that the patient was unable to complete the trial?

A. Two. If the patient cannot successfully complete one trial of the timed 25-foot walk in two consecutive attempts, discontinue the trial. Be sure to indicate this on the source document form.

Q. What should I do if I forgot to reset the stopwatch between trials?

A. If the stopwatch was not reset to 0:00, the trial will have to be repeated. On the timed 25-foot walk record form, note that the trial was repeated and why.

3. 9-hole peg test (9-HPT)

3.1 Description

The 9-HPT is a quantitative measure of upper extremity (arm and hand) function. The 9-HPT is the second component of the MSFC to be administered. Both the dominant and non-dominant hands are tested twice (two consecutive trials of the dominant hand, followed immediately by two consecutive trials of the non-dominant hand). It is important that the 9-HPT be administered on a

solid table (not a rolling hospital bed-side table) and that the small rubber feet are fixed under the 9-HPT apparatus (or the apparatus be anchored by other method).

3.2 Materials needed

9-HPT apparatus, stopwatch, clipboard, 9-HPT Record Form

3.3 Time limit per trial

5 minutes (300 seconds)

3.4 Discontinuation rules

1. If the patient cannot complete one trial of the 9-HPT in 5 minutes.
2. If the patient cannot complete a trial with his or her dominant hand within 5 minutes, move on to the trials with the non-dominant hand.
3. If the patient cannot complete a trial with his or her non-dominant hand, move on to the PASAT.

3.5 Administration

Dominant hand – Trial 1

Make sure that the stopwatch is set to "0:00."

Introduce this section by saying, **"Now, we're going to be measuring your arm and hand function "**

If this is the first visit, ask, **"Are you right- or left-handed?"**

Make a note of the dominant hand for subsequent instructions. Place the 9-HPT apparatus on the table directly in front of the patient. Arrange the apparatus so that the side with the pegs is in front of the hand being tested and the side with the empty pegboard is in front of the hand not being tested.

Read the following instructions to the patient:

> **"On this test, I want you to pick up the pegs one at a time, using one hand only, and put them into the holes as quickly as you can in any order until all the holes are filled. Then, without pausing, remove the pegs one at a time and return them to the container as quickly as you can. We'll have you do this two (2) times with each hand. We'll start with your [DOMINANT] hand. You can hold the peg board steady with your [NON-DOMINANT] hand. If a peg falls onto the table, please retrieve it and continue with the task. If a peg falls on the floor, keep working on the task and I will retrieve it for you. See how fast you can put all of the pegs in and take them out again. Are you ready? Begin."**

Start timing as soon as the patient touches the first peg, and stop timing when the last peg hits the container. If a peg drops on the floor, the examiner may retrieve it and put it back in the peg box. However, if a peg drops onto the table, allow the patient to retrieve it. Record the patient's time under "Dominant hand – Trial 1".

If the subject stops after having put all the pegs into the holes, prompt the subject to remove them as well by saying, **"And now remove them all."**

If the subject begins to remove more than one peg at a time, correct him/her by saying, **"Pick up one peg at a time."**

Dominant hand – Trial 2

After the first trial with the dominant hand, say, **"Good. Now, I'd like you to do the same thing again, once again using your [DOMINANT] hand. See how fast you can put all of the pegs in and take them out again. Ready? Begin."**

Again, start timing as soon as the patient touches the first peg, and stop timing when the last peg hits the container. Record the patient's time under "Dominant hand – Trial 2".

Non-dominant hand – Trials 1 and 2

After the second trial with the dominant hand, rotate the apparatus 180 degrees such that the side with the pegs is now in front of the non-dominant hand and the empty pegboard is in front of the dominant hand.

Then say, **"OK. Now I'd like you to switch and use your [NON-DOMINANT] hand. This time, you can use your [DOMINANT] hand to stabilize the peg board. Ready? Begin."**

Administer, time, and record the two non-dominant hand trials following the procedures described above for dominant hand trials.

3.6 Completing the record form

Record any circumstances that you believe may have affected the patient's performance. These are factors that may have affected the trial, but were not severe enough to necessitate repetition of the trial. Examples include (but are not limited to) the following:

- The patient dropped a peg.
- The patient has a cold.
- The patient forgot eyeglasses and had difficulty seeing pegs.
- The patient talked during the task.

If a trial is repeated, indicate this and specify the reason it had to be repeated. Examples of reasons to repeat a trial include the following:

- The patient knocked entire apparatus on the floor.
- The examiner forgot to start or stop stopwatch.
- The examiner forgot to reset the stopwatch in between trials.

Record only the times for the two successfully completed trials for each hand on the 9-HPT. If the patient could not complete one or both of the trials for either hand of the 9-HPT, record this in the appropriate section of the Record Form.

If the patient's disease has progressed and/or physical limitations prohibit him or her from completing the trial, the examiner should mark "Unable to complete trial due to physical limitations" and then record any specifics that can be observed (*e.g.* "patient unable to use right hand, patient could not complete within time limit" etc.). If the patient did not complete a trial for any other reason, describe the specific circumstances (*e.g.* "patient refused").

3.7 Questions and answers

Q. Is the patient allowed to take a break between the Timed Walk and the 9- HPT?

A. Yes, but the patient is only allowed to rest a maximum of 5 minutes after the Timed Walk before starting the 9-HPT.

Q. How should I determine the dominant hand if the patient indicates that he or she uses both hands?

A. The dominant hand is the hand with which the patient writes (or did write) the majority of the time.

Q. What should I do if the patient talks during the 9-HPT?

A. Discourage the patient from talking as he or she completes the 9-HPT. You may quickly prompt the patient to remain quiet while the trial is in progress. Then, in between trials, emphasize to the patient that his or her concentration should be centered on the 9-HPT and that s/he should wait until the test is over to talk with you.

Q. What should I do if a patient attempts to use both hands to complete a trial of the 9- HPT?

A. Prompt the patient to use only one hand (dominant or non-dominant accordingly). If the patient continues to use both hands, start the trial over, emphasizing to the patient that s/he is to use only the one hand being tested.

Q. What should I do if the patient takes out two pegs at a time?

A. Prompt the patient with, **"Pick up one peg at time."** If the patient responds to the prompt, and subsequently takes only one peg at a time, continue with the trial. If the patient continues to take out two pegs at a time, start the trial over, emphasizing to the patient that he/she must only pick up the pegs one at a time. Record the reason for the repeated trial on the Record Form.

Q. What should I do if the patient takes more than two pegs at a time or even a whole handful of pegs at once?

A. If the patient takes more than two pegs at a time, start the trial over, emphasizing that he/she must pick up only one peg at a time. Record the reason for the repeated trial on the Record Form.

Q. What should I do if the patient knocks a peg out of a hole once it has already been placed?

A. If a peg comes out of a hole and falls onto the table, let the patient retrieve it. If the peg comes out of a hole and falls onto the floor, you should retrieve it and place it in the container.

Q. What should I do if the patient knocks the whole 9-HPT apparatus on the floor?

A. Stop the trial. Reassemble the apparatus, repeat the instructions, and start the trial over. Encourage the patient to hold the pegboard steady with the hand he/she is not using, if possible. Note the incident in the appropriate section of the Record Form.

Q. What should I do if the patient cannot complete the 9-HPT with one hand?

A. Indicate this in the appropriate section of the Record Form. Administer the 9-HPT at every visit, even if the patient was unable to complete it the visit before; the patient may be able to complete the test at subsequent visits.

Q. Is the patient allowed to rest in between trials of the 9HPT?

A. No. The patient should progress directly from trial to trial, pausing only while you read the instructions for each subsequent trial.

Q. What should I do if I forgot to reset the stopwatch in between trials?

A. If the stopwatch was not reset to 0:00 at the beginning of the trial, that trial will have to be repeated. Note on the Record Form which trial{s) was repeated and why.

Q. What should I do if I forgot to turn the 9-HPT apparatus when switching to the non- dominant hand?

A. Start the trial over and note the reason for the repeated trial on the Record Form.

Q. What should I do if I realize the 9-HPT kit is missing pegs?

A. A supply of extra pegs can be ordered through the sponsor. It is the rater's responsibility to make sure that all equipment is complete and in good working order prior to a study visit.

4. Paced auditory serial addition test (PASAT)

4.1 Description

The PASAT is a measure of cognitive function that specifically assesses auditory information processing speed and flexibility, as well as calculation ability. The PASAT is the last measure administered at each evaluation. It is presented on audio CD to control the rate of stimulus presentation. Single digits are presented every 3 seconds and the patient must add each new digit to the one immediately prior to it. The test result is the number of correct sums given (out of 60 possible). To minimize familiarity with stimulus items in clinical trials and other serial studies, two alternate forms (A and B) have been developed; the order of these should be counterbalanced across testing sessions (*e.g.*, A, B, A , B). Before administering the actual PASAT test for the first time, at least three initial complete training trials (each comprising three practice sequences) should be administered. For this training, the alternate form (A or B) should be used which will be applied in the actual test. Materials needed

CD player, audio CD with PASAT stimuli, clipboard, PASAT Record Forms

4.2 Discontinuation rules

If the patient cannot get at least two answers correct (consecutive or not) on anyone of the three practice sequences.

4.3 Administration

Verify that you have the correct Record Form and CD track (Language, Form A or B) before you start reading the instructions for the Practice Trial to the patient.

PASAT-3" Practice Trials

For Part 1 say, **"On this CD you are going to hear a series of single digit numbers that will be presented at the rate of one every 3 seconds. Listen for the first two numbers, add them up, and tell me your answer. When you hear the next number, add it to the one you heard on the CD right before it. Continue to add the next number to each preceding one. Remember, you are not being asked to give me a running total, but rather the sum of the last two numbers that were spoken on the CD."**

Then give the following example: **"For example, if the first two numbers were '5' and '7', you would say '12'. If the next number were '3', you would say '10'. Then if the next number were '2', you would say '5'."**

If the patient is having difficulty understanding these instructions, write 5, 7, 3 and 2 on a sheet of paper and repeat the instructions, demonstrating how the task is done.

Then say, **"This is a challenging task. If you lose your place, just jump right back in – listen for two numbers in a row and add them up and keep going. There are some practice items at the beginning of the CD. Let's try those first."**

Play the sample items, stopping the CD after the last practice item. Repeat the practice items, if necessary, until the subject understands the instructions (up to three times). You should administer at least three complete practice trials (each comprising three practice sequences) before administering the actual test for the first time. If the patient begins to give you a running total, stop the practice immediately and explain the task again, emphasizing that he/she is not to give you a running total. Then start the practice trial again from the beginning. If the patient begins adding each number to the number two previous to it, again stop the practice immediately, explain the correct way to do the task, and start the practice trial from the beginning. If the patient merely makes a math error, do not stop the CD; continue with the practice trial. After two consecutive 'no responses', prompt him/her to resume by saying, **"Jump back in with the next two numbers you hear."**

Administer the practice sequence a maximum of three times. Record answers in the space provided on the back of the PASAT Record Form. Between the initial training session and the first actual administration of the test, the patients should be allowed a break of approximately ½ hour.

PASAT

Once it is clear that the patient possesses sufficient understanding of the task, begin the actual test. Before starting the test, remind him/her: **"Remember, if you get lost, just jump back in because I can't stop the test once it has begun."**

Discourage talking and oral calculations during the test; only the patient's answers should be spoken out loud. The patient may need prompting to continue the test if she/he gets lost. After five consecutive 'no responses', redirect the patient quickly by saying, **"Jump back in"**, but do not stop the CD.

4.4 Completing the record form

Circle all correct answers. Write in any incorrect responses in the space provided. Write "NR" (for "no response") when no response was given. If the patient corrects him/herself after giving a response, count the amended answer as the response. The amended response is the one that will be used in determining total correct, regardless of whether it was the correct or incorrect response. Slash through the old response and write in 'SC' with a circle around it to indicate that the patient self-corrected.

Each section of the PASAT has a maximum of 60 correct answers (*i.e.* 61 digits are presented for each part). Count the total number correct (number of circled answers) for the PASAT and record on both the PASAT Record Form and the Summary Score Sheet.

Finally, record any circumstances that you believe may have affected the patient's performance. These are factors that may have affected the trial, but were not severe enough to necessitate repetition of the trial. Examples include, but are not limited to, the following:

- Subtle noises outside of the testing room.
- Patient reports frustration or mild distress.
- Patient talked during test (other than to give answers).

If a trial must be repeated, indicate this and specify the reason why it had to be repeated. Examples of reasons to repeat a trial include, but are not limited to the following:

- Test interrupted (*e.g.* someone walked into the room or other major disturbance).
- Examiner error, such as starting the wrong CD track or using the wrong form.

Record only totals for the successfully completed PASAT.

If the patient is unable to perform the PASAT (*i.e.* cannot get at least two correct on any practice and at least one correct on the test portion), the examiner should indicate "unable to complete due to cognitive limitations" and record any specific observations. If the patient did not complete a trial for any other reason, record the reasons for this as well (*e.g.* “patient refused to complete test” etc.).

4.5 Questions and answers

Q. Can I operate the CD player on batteries when administering the PASAT?

A. No. You should always run the CD player on electric power to ensure that the rate of stimulus presentation is standardized.

Q. What should I do if the patient does not respond at all on the PASAT practice?

A. If the patient has not made a response to any of the first five stimulus items, stop the practice trial and explain the task again. Remind the patient to state his/her answers aloud. Do not count the five "no responses" as one of the three practice trials. (This situation is likely to occur only on the patient's first visit, when the patient is not familiar with the task.)

Q. Am I allowed to provide the patient with helpful strategies for the PASAT?

A. No. You are allowed to give the patient only the standard instructions. Further explanations are allowed if the patient is having difficulty understanding the test, but these should be general instructions, not specific strategies to improve the patient's score.

Q. Is the patient allowed to perform the calculations out loud, for example, to say "3 + 9 is 12", etc.?

A. All calculations are to be done silently. Indicate to the patient that oral calculations could interfere with his or her performance. Discourage the patient from talking, except to provide his or her answer. Watch for this behavior during practice trials and correct it before proceeding to the actual test.

Q. Is the patient allowed to "Write" on the table with his or her finger during the PASAT?

A. No. Observe the patient during the practice trials for this behavior. Instruct the patient that all calculations are to be done in his or her head and that "writing down" the numbers is not permitted.

Q. Is the patient allowed to count on his or her fingers?

A. No. Again, if this behavior is exhibited by the patient during the practice, explain that it is not allowed and that all calculations must be done in the patient's head.

Q. I had already started the PASAT test and the patient asked to start over. Is this OK?

A. No. As the instructions indicate, once the PASAT test has begun, you cannot stop it even if the patient requests to do so. The only reasons that you would stop the PASAT test would be because of a major external disturbance, equipment failure/malfunction, or something of this nature.

Q. What should I do if the patient refuses to do the PASAT?

A. The PASAT is a primary component of the MS Functional Composite. The patient should be encouraged to do the PASAT, however, patients of the NA cohort will not be excluded from the trial if they refuse to do the PASAT. In case of patients in the UK cohort, replacement of the patient should be considered if the patient may refuse to do other test also. Please, contact the Sponsor to discuss the matter.

Appendix 12

Pharmacogenetic investigations

Content

1. Introduction 2
2. Rationale and reason for (pharmaco-)genetic evaluations 2
3. Objectives 3
4. Methodology...3
5. Ethical and data privacy considerations 4
6. Sampling protocol 6
7. Evaluation of data and statistical considerations...................................7
8. Excerpt from the SAG Group SOP for pharmacogenetic studies 8

1. Introduction

This appendix specifies the pharmacogenetic evaluations to be performed under the Berlex protocol 308272 which describes a long-term follow-up of the patients enrolled in the pivotal study for Betaseron® (interferon beta 1b) in relapsing-remitting multiple sclerosis (RRMS). For these pharmacogenetic evaluations, DNA samples will be obtained and analyzed for genes hypothesized to play a role in multiple sclerosis (MS) itself or in the effects of Betaseron® on the disease.

A separate patients' informed consent is required for the pharmacogenetic part of the study. A patient's decision regarding potential participation in the pharmacogenetic evaluations will not affect any other aspects of the conduct of study 308272.

The data collected for and by means of the pharmacogenetic evaluations will contribute to a larger database from other previous, ongoing and planned studies on treatments for MS.

2. Rationale and reason for (pharmaco-)genetic evaluations

Increasing knowledge about the human genome and rapid developments of tools for genetic analysis allow to gain a much deeper understanding of the contribution of genetic factors to MS and to the treatment response.

A number of projects on disease genetics have resulted in a list of genes that seem to contribute to either the risk of developing MS or to the severity of disease course. Further sampling of DNA in therapeutic studies in MS or studies that target the long-term effects of treatment intervention as the study 308272 will help to explore these genes and additional genes (shown or hypothesized to be possibly associated with the disease or with the response to a drug) and whether they can be of predictive value, i.e. help to determine the expected treatment effect. Also, genetic information may be obtained that helps to better understand why tolerability to Betaseron® differs to a relevant extent among patients. In the absence of reliable clinical or paraclinical tests that allow to predict outcome of treatment, there is a clear need to further explore potential genetic factors, and even if clinical or paraclinical tests of predictive value are identified, such genetic information will be of additional relevance for making rationale treatment decisions.

3. Objectives

To employ genetic techniques for disease genetics and pharmacogenetics, i.e. to better understand the genetic factors

a) underlying or contributing to the etiology and, particularly, to different courses of the disease (e.g. different degrees of clinical and MRI activity; different degree of loss of cognitive function), or

b) associated with treatment response and side effects under treatment with Betaseron®.

Genetic factors from the different groups might be of predictive value and thus become relevant for treatment decisions. They are therefore denoted together as "pharmacogenetics" in this protocol.

4. Methodology

Candidate genes or gene clusters or chromosomal loci either already known from the literature or own research, or proposed in the future, will be investigated to explore whether there is an association between genetic polymorphisms and MS occurrence or disease phenotype on one hand and differential drug response with regard to efficacy and side effects on the other hand. As currently known, the following genes and chromosomal loci have been pre-specified to be of special importance regarding a potential association with MS:

- Human Leukocyte class II antigens (HLA)
- MBP
- T-cell receptor beta
- Immunoglobulin variable chain gene loci
- Tumor necrosis factor
- Interleukin-1 receptor agonist
- Interleukin-1 beta allele 2
- Interleukin-4
- Cytotoxic T-lymphocyte-associated protein 4
- Fc-gamma-receptor-IIIB and IIA
- Intercellular adhesion molecule 1
- C-C chemokine receptor 5
- Dopamine D2 receptor
- Gamma-aminobutyric acid A3 receptor
- Spinocerebellar ataxia type 2 gene
- Apolipoprotein C-II
- Glutathione S-transferase GSTM3
- Osteopontin
- Ciliary neurotrophic factor (CNTF)
- CD45.

- Chromosomal loci 6p21, 5p14-p12, 17q22-q24 and loci on chromosome 5 and 17, 4cen, 11tel, and 2q.

The following group of genes are of interest with regard to the mechanism of action and, potentially, the efficacy of Betaseron®:

- Major components of the signaling pathways of the interferon beta type I receptor, e.g. the JAK family of tyrosine kinases and the STAT family of transcription factors.
- Further downstream, via the activation of this signaling pathway, a large number of genes is either induced or their expression down-regulated. All these genes are also of interest as their gene expression changes might be contribute to the effects of Betaseron®.

Furthermore, Professor Ebers' laboratory in Oxford has recently identified a genetic marker which appears to differentiate between patients having a malignant compared to a benign course. Further details would be available at request. These findings derived as they are from the Natural History database and other patients from London (Ontario) have not been applied yet to patients in a clinical trial. The collection of long-term outcome information on this original cohort should allow for evaluation of trial-related outcome in the context of variable compliance with a continuation of therapy in an exploratory fashion. It is planned to use 4 mL of EDTA blood for this purpose.

The following institutions will be involved:

- Blood and DNA storage	- Laboratorium für Klinische Forschung (LKF)<br>D-24223 Raisdorf, Germany
- Purification of DNA	- AGOWA Gesellschaft für molekularbiologische Technologie<br>D-12489 Berlin, Germany
- Genetic analyses	- Specialized genetic laboratories capable to perform the a.m. genetic analyses. A documentation which laboratories are involved in the genetic analyses is available on request at any time.

5. Ethical and data privacy considerations

Pharmacogenetic analyses will focus on and be restricted to MS-related and/or MS-treatment-related genes of interest. Since both, the disease and the treatment response (efficacy and safety/tolerability) of Betaseron® are far from being fully understood there may be a high number of potential genes of interest.

For all pharmacogenetic activities and evaluations, a separate patient's informed consent must be obtained (cf. Appendix 2 to this protocol). The patient may decide to participate in pharmacogenetic evaluations independent of her/his decision to participate in the clinical study. The patient can withdraw her/his DNA sample at any time independent of his/her participation in the clinical study. The signed informed consent forms are stored separately from other study-related documentation,

i.e., not in the Investigator's Trial File, and separate from the hospital records for the patient. The signed informed consent forms are not monitored by any Sponsor or sponsor-related staff.

Generally, each DNA sample will be used for a limited period of time and will be destroyed 20 years after the end of the study during which it had been collected. The patient is offered the option to have parts or all of her/ his samples completely anonymized. In this case the limitations in time and scope do not apply.

Several measures have been established to ensure the privacy of the patients and the protection of data. Among these are the following:

- Double-coding of DNA samples and storage of samples separate from any other samples pertaining to the study (once shipped to the central laboratory LKF), double coding will be applied to DNA samples before any pharmacogenetic analyses are performed.
- Therefore, all pharmacogenetic data, i.e. the genetic data resulting from the analysis of the DNA samples, will also be double coded.
- Separate laboratories will handle and store the DNA samples and perform pharmacogenetic analyses according to SOPs agreed upon with the sponsor.
- At any time, without giving any reason and independently from participation in the study, the patient can request the investigator to withdraw his or her DNA sample. There is one exception when data (not the sample) cannot be withdrawn; cf. the detailed description in the SOP attached (Section 8) and the description in the patient information (Appendix 2). Sample tracking will be provided by the laboratory storing the samples for pharmacogenetic analyses; this will allow withdrawal of samples via the investigator at any time
- The sponsor has assigned the analysis of DNA data in the context of clinical results to a 'designated biometrician' who has to sign a separate confidentiality statement. Matching (single coded) phenotypic and (double coded) genetic data will be performed in a so-called 'secure data area'. This is separate hardware in a separate room with multiple access control to the room and to the data. The 'designated biometrician' will not be involved otherwise in the analysis of the underlying study.
- Any presentation or publication (internal or external) of results will not contain individual clinical or personal patient data that might allow identification of individual patients.
- A pharmacogenetics advisory board oversees all pharmacogenetic activities in the context of clinical studies sponsored by the company by giving advice on the principles for pharmacogenetic analyses and on individual protocols.
- An SOP is in place that regulates all details of sample and data handling for DNA samples (an excerpt of the sponsor's SOP covering pharmacogenetic studies is attached; cf. Section 8).
- The patient information and informed consent for the pharmacogenetic analyses describes in detail the risks, benefits, scope of investigation and scope of potential contribution to the better understanding of MS and MS treatment (cf. Appendix 2).

6. Sampling protocol

For DNA sampling, 12 mL of EDTA blood will be collected, preferably concomitantly with blood sampling scheduled for the underlying study. However, the samples may be obtained at any time during the study.

7. Evaluation of data and statistical considerations

Details of the analysis will be specified in a separate analysis plan. All analyses are considered exploratory.

In general terms, three different categories of pharmacogenetic results may be generated:

1. Disease genetics with regard to frequencies of clinical events and key clinical milestones.
2. Genetics with regard to treatment response.
3. Genetics with regard to safety and tolerability of treatment.

The following is a preliminary list of response definitions to be used in an exploratory approach to analyze pharmacogenetic findings in the context of clinical and MRI findings. This list is not considered to represent the entire range of possible analyses and may be modified as necessary during the conduct of these exploratory analyses. All pharmacogenetic analyses will be performed in a blinded fashion with regard to the original treatment group allocation.

Separately for the NA cohort (and subsets of the cohort defined by different threshold related to length of exposure to Betaseron®) and the UK cohort:

- Proportion of patients reaching an EDSS endpoint of 3.0 in the subset of patients with an EDSS <3.0 before entering the pivotal study in 1988/89.
- Proportion of patients reaching an EDSS endpoint of 6.0.
- Proportion of patients with conversion to secondary progressive MS (SPMS).
- Proportion of patients with a higher frequency of relapses vs. patients with a lower frequency of relapses. Various thresholds of relapse frequency may be used to define different subgroups of patients.
- Proportion of patients with a higher degree of MRI activity vs. patients with a lower degree of MRI activity. Various thresholds of MRI activity may be used to define different subgroups of patients.

- Proportion of patients having terminated treatment due to a range of potentially treatment-related side effects (cf. Section 7.5.2.11 of the study protocol 308272).
- Combination of relapse status and MRI activity.
- Combination of relapse status, EDSS progression status and MRI activity.
- Other MRI parameters, e.g. number and volume of T2 lesions, number and volume of hypointense lesions, cerebral atrophy.
- Such "proportion of ..." analyses may be performed for various time-points. Furthermore, "time to ..." endpoints of the same clinical parameters may be evaluated by defining subgroups of patients with longest "time to ..." intervals and those with shortest intervals.

All analyses will be guided by the clinical outcome of the study. As a general approach, analyses may be performed, in a first step, for a subgroup of patients only representing "extremes" of the spectrum of response or disease activity; further analyses may be guided by the findings of these initial analyses. However, there remains the option that no pharmacogenetic analyses will done.

To improve the power of pharmacogenetic analyses, adjustment for and interaction with a number of parameters will be performed/analyzed, e.g. gender, number of lesions at baseline, NAb status in patients with ongoing Betaseron® treatment, continuous treatment with anti-inflammatory drugs, etc.

8. Excerpt from the SAG Group SOP for pharmacogenetic studies

Excerpt from SAG's SOP (SOP-GCT11.01)
"Pharmacogenetic Analyses: Handling and Processing of DNA Samples and Data"

This excerpt of SAG's SOP is meant to be provided to IRBs and IECs together with the submission of pharmacogenetic protocols. This excerpt is a 1:1 verbatim version of all contents of the SOP except some internal organizational aspects that do not affect the handling and processing of DNA samples and data; also, the procedures for special situations that do not apply in the current study have been deleted. The complete SOP will be provided on request. Where necessary certain SAG internal functions are explained in parenthesis. This attachment contains the following appendices:

Appendix A: Flow Chart for the processing of samples and data

Appendix B: Table listing the functions and roles of personnel involved in the pharmacogenetic analyses ("Administrative structure and responsibilities")

Appendix C: Confidentiality agreement form for personnel involved in the generation and use of the DNA sample ID and for personnel designated for the combination of the pharmacogenetic data and the clinical study database during the statistical analysis

1. Purpose

This SOP describes

- the procedures employed in the sampling and handling of samples for pharmacogenetic analyses from patients enrolled in clinical studies sponsored by the SAG Group or its subsidiaries including collaborations where no formal sponsorship is adopted, and
- the procedures for handling the data obtained by these analyses with a special focus on the measures that have been installed to ensure that the confidentiality of the data is maintained.

In the context of this SOP, pharmacogenetic analyses are defined as analyses of hereditary DNA relating to the disease as well as to the efficacy and safety of a drug in a given indication: This encompasses pharmacokinetic and pharmacodynamic aspects and aspects related to disease heterogeneity.

2. Scope

All clinical studies in which pharmacogenetic analyses are to be incorporated.

3. Procedures for sampling and pharmacogenetic analyses

Two scenarios for pharmacogenetic (PGt) analyses have been defined for which the underlying concepts are described below. A combination of both is possible.

3.1 Double coding of pharmacogenetic samples and restricted access to pharmacogenetic data

Ensuring the confidentiality of data is the major overall aim of the procedures described here. Therefore the general principle to be followed is that only a minimal number of personnel at the central sample repository (CSR) or Corporate Clinical Operations (CCO; a SAG function) will be involved in handling the key code list (see below) and any PGt data that can potentially identify an individual patient.

A separate patient's informed consent must authorize the sampling for pharmacogenetic analyses and usually covers all genes relevant for the efficacy and/or safety of a drug in the given indication and the disease itself. PGt analyses are restricted to aspects related to the treatment and the disease evaluated.

Where required, an independent person (e.g. a doctor at the trial center not involved otherwise in the conduct of the main clinical trial) should be available to be contacted by the patient to discuss her/his open questions regarding PGt analyses before signing the informed consent form.

There is no access to the informed consent for PGt analyses by the sponsor. Instead CSR has to assure that for all PGt samples a written confirmation of the investigator is obtained stating that the separate patient informed consent has been signed. This document has to be sent by the investigator to the designated person at the CSR responsible for applying the second code (see below) to the PGt samples.

Counseling of the patient may become necessary in this scenario. In case of relevant results from the PGt analyses with regard to the underlying disease, the efficacy, and/or the safety of the drug investigated, investigators are informed in a timely manner using aggregated results (no individual data). They will offer counseling to their patients and the patients will have to decide whether or not to ask for a confirmatory diagnosis specific to him or her and for his or her individual data. In case of safety findings being related to PGt findings, counseling may even become mandatory (see 3.2.1).

The PGt sample intended for PGt analyses is sent to the CSR coded with the patient ID.

For the purpose of the PGt analyses themselves, Corporate Clinical Operations – Standards and IT Systems (CCO-SI; an internal SAG function) generates a second code as an additional step to de-identify samples to be used in pharmacogenetic analyses. The key code list connecting the first ID and the second ID is used in the following contexts only:

1. for the second encryption taking place at the CSR
2. to combine clinical data and PGt data temporarily for statistical analyses
3. to "translate" a set of patient IDs selected for analyses into a list of DNA sample IDs for retrieval of subsets of samples by the CSR
4. in case of withdrawal of an informed consent for destruction of the respective sample

The second ID applied by using this second key code list is called "DNA sample ID" hereafter. Only designated personnel at the CSR and at CCO has access to the key code list.

A supplemental study protocol containing a laboratory and statistical analysis plan is generated to describe the scope of the analyses to be performed, how they are to be performed and how the PGt data and the clinical data are to be statistically evaluated and aggregated for the report. The rationale for PGt analyses is based e.g. on hypotheses based on scientific literature, results of other studies or other sources of scientific data. Additional review by the local ethics committee(s) is not considered to be necessary as the approval for the full scope of analyses is obtained with the original approval.

The clinical data and the results of the PGt analyses are stored in separate databases. Access to and the statistical analyses of both, the clinical data and PGt data in combination is restricted to specially authorized members of the CCO function not involved otherwise in the conduct or evaluation of the study. She/he releases aggregated data only i.e. she/he will only release data which will not allow the identification of individual patients except in cases described under 3.2.1. The two databases are only combined for the time the statistical analyses are performed.

A supplementary report to the main study report is prepared covering the scope and results of the PGt analyses.

The PGt sample is held by the CSR and individual data are stored in the separate database for 20 years after completion of the study defined hereafter as time of database closure. Storage for variable periods of time according to regulatory and local ethics committee conditions has to be taken into consideration.

There is also the option that relevant local ethics committees may allow not to destroy but to continue to store samples and certain classifying clinical data after complete anonymization at the end of the foreseen storage time, e.g. for reasons of maintaining invaluable data/sample collections.

Confidentiality in case of special safety issues

Safety issues might trigger PGt analyses. Should the results of these analyses reveal a sufficiently high probability of prediction of severe adverse reactions to the medication the following actions are taken:

1. Future patients receiving the same study medication have to be pre-tested
2. Patients with the same genotype have to be taken off the study medication
3. Counseling to the patient has to be given in case that other marketed drugs potentially imply similar risks

This procedure might unavoidably reveal certain PGt information of individual patients.

3.2 General responsibilities

The sponsor is responsible for determining the clinical studies in which samples for PGt analyses are to be collected and for the scenario(s) to be followed. The ownership of the samples and PGt data lies with the sponsor.

Custody of the PGt samples and data as a general rule has to be handed over to the function Corporate Pharmacogenomics (CPG) by the sponsor. If custody cannot be transferred to CPG, the reasons have to be documented in writing. CPG is responsible for handling of the samples from the time of arrival at the CSR until the time of destruction of the PGt samples and data.

CPG is responsible for obtaining approval from the Pharmacogenetic Advisory Board (PGAB, see below) regarding the general procedures for PGt analyses. CPG informs the PGAB and asks for advice regarding all studies in which a PGt question is addressed.

The CSR serves as a central storage place for PGt samples. It may physically be split up into several locations. All CSRs function as one virtual repository with identical standards and procedures. The central repository has to be used for any PGt study. The CSR is responsible for generating, implementing and monitoring detailed SOP(s) to assure adherence to the rules described in this SOP.

3.3 The Pharmacogenetic Advisory Board and other bodies involved

For further review and advice a Pharmacogenetic Advisory Board (PGAB) is established with external experts in pharmacogenetics, biomedical sciences, ethics and law and representatives from Corporate Pharmacogenomics. The external experts form the majority. The PGAB approves the general procedures as they are laid down in this SOP and other relevant documents. It is informed and asked for advice by CPG on all study protocols involving PGt sampling or analyses, and whenever non-prespecified PGt analyses are to be performed. In this review process it has a veto right which can only be overruled by a decision of the member responsible for research and development in the Board of Executive Directors, SAG. The review of the study protocol can take place in parallel to seeking approval by the local ethics committees and local authorities where appropriate.

In case of non-prespecified PGt analyses, the supplemental study protocol describing the specified analyses to be done is given to the PGAB for review via CPG.

The PGAB also has to be involved in the decision making on the potential relevance of results of PGt analyses and on the need to inform the investigators with regard to counseling of patients.

Where applicable, the PGAB has to consult with any body involved in safeguarding a study (e.g. the Steering Committee and the Advisory Board of the study).

3.4 Responsibilities regarding initiation of pharmacogenetic sampling

The study manager (SAG function in studies sponsored by SAG Group companies) has the following responsibilities with regard to initiating PGt sampling:

- The study manager informs the CSR and the CPG function about the logistics used in the trial that is planned to include PGt analyses.
- She/he has to inform the relevant CCO-SI function in a timely manner about the planned sampling and about the CSR contact person(s) to which the key code list for the DNA sample ID has to be communicated.
- She/he is responsible for the actual set up of the sampling in the centers, the logistics up to the arrival at the CSR (where CPG takes custody) and the operational aspects of organizing the PGt testing and analyses in a particular study. CPG will provide support where needed.
- She/he is responsible for obtaining approval from the relevant local ethics committee(s) and local authorities where applicable for sampling for PGt analyses in a given clinical study.
- She/he is responsible for updating and archiving the table shown in appendix A.

The investigator has the following responsibilities with regard to initiating PGt sampling:

- The investigator must obtain informed consent of the patients for participation in the PGt part of the study after approval of the pharmacogenetic testing and analyses by the relevant local ethics committee(s) and (where applicable) local authorities. She/he is responsible for archiving the informed consent form separately from the patient file.

The responsibilities for processes related to the key code list for the DNA sample ID are separated from the activities in handling and analyses of the PGt data:

- CCO-SI (SAG biometry function) nominates a person responsible for providing, holding and deleting the key code list for the DNA sample ID applied at the CSR (hereafter referred to as "key code list manager"). The key code list manager or her/his designate must not be involved otherwise in the conduct or evaluation of the study.
- A designated biometrician from CCO is responsible for handling the PGt data separately from the clinical data according to the procedures defined by this SOP. Only she/he is allowed to perform joint analyses of PGt data and clinical data using the key code list for the DNA sample ID. The biometrician or her/his designate must not be involved otherwise in the conduct or evaluation of the study.
- Delegation of the two functions mentioned above is to be kept to a minimum to limit the number of staff with access to PGt clinical data from individual patients.

3.5 Sampling

The CSR or the central laboratory for an individual study ships the vials for PGt related sampling to the investigator together with the appropriate sampling form.

The sample intended for PGt analyses together with the respective forms (see below) is sent to the CSR by the investigator. Alternatively, the PGt sample can be stored at the study center and sent to CSR at regular specified intervals.

The investigator confirms with the CSR that the separate informed consent for PGt sampling and analyses has been obtained on a form accompanying the sample. If this document is missing the CSR has to clarify if informed consent was obtained. Only after these confirmations have been obtained samples can be used for any PGt analyses.

3.6 Processing and tracking of pharmacogenetic samples

Tracking records have to be kept at two physically separated locations.

Samples received by the CSR are labeled with the DNA sample ID using the key code list generated by the key code list manager. The DNA sample ID is applied before any analysis or shipment is performed or at the time of DNA extraction, whichever occurs first.

DNA is isolated by routine methods and stored in aliquots at -20°C, -70°C or in liquid nitrogen for future analyses.

The laboratories performing analyses with the PGt samples will only know and use the DNA sample ID.

The number of aliquots of DNA for each sample and their location are to be documented by the CSR.

After sample collection, DNA extraction and encryption using the key code list for the DNA sample ID has been completed, the key code list is to be returned to the responsible key code list manager at SAG's biometry function CCO-SI.

The tracking of samples being sent to other laboratories for PGt testing has to be performed by the CSR.

All sample transfers are to be recorded by the CSR (with sample transfer date, amount of DNA, volume of sample, recipient, DNA sample ID).

All analyzing laboratories keep records of storage and usage of samples and return unused residues to the CSR if no further analyses are to be expected. Alternatively, if the (remaining) amount of DNA/volume of the sample aliquots involved is small, the lab is allowed to destroy the sample residues. This is to be documented in writing, and the CSR must be informed. The CSR must also be informed if sample aliquots have been completely used by the lab.

To ensure confidentiality, all information regarding PGt samples (including e.g. billing for PGt sample collection by the centers) has to be handled by the CSR.

The core clinician (main responsible person at SAG for a clinical study), assisted by CPG and the Auditing function has to ensure that laboratories performing the PGt analyses follow the rules specified in the analysis plan.

At any time, the sponsor and regulatory authorities have the right to inspect the CSR and to audit the processes related to this SOP.

3.7 Initiating pharmacogenetic analyses

The core clinician has to initiate PGt analyses. She/he has to generate a supplemental study protocol outlining the rationale and the details of the PGt analyses and the statistical analyses to be performed. CPG assists and may “trigger” this process.

The core clinician has to ensure that the aims and scope of PGt analyses are in agreement with the aims of PGt analyses to which the patient has given informed consent.

Details of the planned pharmacogenetic and statistical analyses are to be discussed with the involved biometricians before PGt analyses are commenced. The core clinician, the biometrician and the researchers performing the PGt tests have to agree on the PGt test to be performed, the statistical analyses to be used and the required scope of evaluation of data. These procedures are described in the supplemental study protocol which has to be submitted to the PGAB for review via CPG.

CPG provides advice with regard to selection of appropriate analyzing laboratories and technical platforms to be used.

After all these prerequisites have been fulfilled, the core clinician authorizes the CSR to ship aliquots of the samples for PGt analyses.

3.8 Evaluation of combined pharmacogenetic and clinical data and report of results

The evaluation of the PGt results in the context of clinical data is to be performed by the biometrician according to the previously agreed analysis plan (as part of the original study protocol and/or the supplemental study protocol describing non-prespecified analyses, respectively). Only she/he is allowed to perform a joint analysis of the PGt data coded with the DNA sample ID and the clinical data, by use of the key code list linking the patient ID and the DNA sample ID.

The core clinician assisted by CPG, the biometrician designated for the integrated analysis, the study biometrician and the project biometrician, if applicable, are responsible for the proper discussion and usage of the aggregated results. Only aggregated results, i.e. results that do not allow the identification of individual patients will be provided by the biometrician performing the integrated analysis of data.

No public presentation or publication of results will contain individual clinical or personal patient data that might allow the identification of the patient who donated the sample.

Should the research program generate relevant results with regard to the underlying disease, the efficacy, and/or the safety of the drug investigated, investigators are informed in a timely manner of such aggregated results (i.e. no individual data). If the patient has consented to be re-contacted, they will offer counseling to their patients. Patients will have to decide whether or not to ask for his or her individual data. The PGAB has to be consulted before such information is given to the investigators (see Section 3.3). The procedures in the special case of severe adverse reactions related to a genotype are described in Section 3.1.1.

3.9 Withdrawal of samples and data

A patient may withdraw consent for samples and data be used in PGt analyses:

The written request from the patient is stored separately from the patient file by the investigator. The investigator informs the CSR about the withdrawal of the patient informed consent on a separate sheet containing only the patient ID. The CSR is responsible for asking the key code list manager to provide her/him with the respective DNA sample ID needed to retrieve the sample. The key code list manager countersigns the respective request form. In any case the CSR receives only the minimal information necessary to retrieve the respective sample.

The CSR destroys the sample or may send it to a third place for destruction. As long as samples have not been encrypted using the DNA sample ID, destruction can only take place at the CSR. This destruction process has to be documented by the CSR. The CSR confirms destruction of samples in writing to the key code list manager The CSR keeps a record of the destruction.

The CSR further informs all laboratories which have obtained aliquots of the sample about the withdrawal of this particular sample. These labs have to confirm destruction of the sample in writing.

The key code list manager informs the biometrician about the withdrawal of the consent for data be used in PGt analyses for this individual patient.

All data generated by PGt analysis of this particular sample have to be erased. The responsibility lies with the biometrician informed by the key code list manager. Data that has already been entered into an overall evaluation of the clinical study is exempted from this deletion process (see patient informed consent provided by CPG). The biometrician keeps a record of the deletion of the data. The key code list for the DNA sample ID remains unchanged.

The investigator is notified about the destruction of the sample and deletion of the data by the key code list manager. This information is passed on to the patient by the investigator who documents the process.

3.10 Final destruction of all samples, deletion of data in the separate database and deletion of the key code list

3.10.1 Time of destruction

Samples for PGt analyses will be destroyed at one of the following time points:

1. 20 years after database closure of the study.
2. There is also the possibility that relevant local ethics committees may allow not to destroy but to continue to store samples and certain classifying data (e.g. "age group 50-60") not infringing the anonymization at the end of the foreseen storage time mentioned above, e.g. for reasons of maintaining invaluable data/sample collections. In this case the corporate head of clinical development together with CPG has to determine the procedures to be applied.
3. If the sponsor decides to prematurely terminate the PGt analyses project, destruction is ordered by the corporate head of clinical development contacting CPG in writing well before the scheduled destruction date.

The study manager informs the CSR about the planned date of database closure. The CSR keeps track of the 20-year period following database closure and informs CPG in due time about expiry of the period.

3.10.2 Process of destruction of pharmacogenetic samples and key code list

CPG orders the destruction of the pharmacogenetic samples at the CSR in writing. The CSR destroys the samples or may send them to a third place for destruction. As long as samples have not been encrypted using the DNA sample ID, destruction can only take place at the CSR.

If at the time of destruction, aliquots have been sent to an analyzing laboratory, the following applies: if neither the respective residues have been returned by this lab to the CSR nor the CSR has been informed by the lab that the aliquot had been used completely or that any residues had been destroyed, then the CSR informs all such laboratories about the need to destroy any residues of these samples. These labs have to confirm destruction of samples in writing.

This destruction process has to be documented by the CSR. The CSR confirms destruction of samples in writing to CPG. The CSR keeps a record of the destruction. CPG documents this process and keeps a record of it.

CPG informs the key code list manager about the destruction of the samples. The key code list manager deletes the key code list thereafter, documents this process, informs CPG and keeps a record of it. CPG documents this process and keeps a record of it.

The procedure will be audited by an external auditor.

3.10.3 Process of destruction of pharmacogenetic data in the separate database

CPG informs the designated biometrician about the scheduled date of destruction of the data in the separate database. The biometrician deletes the individual PGt data, documents this process and keeps a record of it. CPG documents this process and keeps a record of it.

4. Abbreviations

CCO	Corporate Clinical Operations
CCO-SI	Corporate Clinical Operations Standards and IT Systems
CPG	Corporate Pharmacogenomics
CSR	Central Sample Repository
DNA	Deoxyribonucleic acid
ID	Identifier (e.g. number coding a sample)
PGt	Pharmacogenetic
PGAB	Pharmacogenetic Advisory Board
SOP	Standard Operating Procedure

5. Appendices

Appendix A: Flow Chart for the processing of samples and data

Appendix B: Table listing the functions and roles of personnel involved in the pharmacogenetic analyses ("Administrative structure and responsibilities")

Appendix C: Confidentiality agreement form for personnel involved in the generation and use of the DNA sample ID and for personnel designated for the combination of the pharmacogenetic data and the clinical study database during the statistical analysis

Appendix A: Flow Chart for the processing of samples and data

1. Study protocol mentions the possibility to perform predefined and/ or subsequently determined pharmacogenetic analysis related to the efficacy or safety of a study drug in the given indication or the disease itself (not part of exclusion/ inclusion criteria).

2a) Pharmacogenetics Advisory Board is informed and asked for advice.

2b) Local ethics approval for protocol including the pharmacogenetic analyses. Both processes can take place in parallel.

3. Generation of the key code list for the DNA sample ID.

4. Separate patient informed consent is obtained.

5. Sample with patient identifier sent to central sample repository (CSR) for storage. Second encryption generated by CCO-SI applied at CSR before first pharmacogenetic analysis.

6. A supplemental study protocol is prepared defining rationale and details of pharmacogenetic analyses.

7. Pharmacogenetic Advisory Board is informed and reviews the supplemental study protocol.

8. Sample aliquot is sent to analytical laboratory.

9. Results of pharmacogenetic analyses (encrypted by the DNA sample ID) are incorporated into a pharmacogenetics database separated from the clinical database where information is coded with the patient ID.

10. Statistical analyses are performed in the “restricted area”. A report on results of pharmacogenetic analyses is prepared as a supplement to the study report. Results of pharmacogenetic analyses will be reported and displayed only in an aggregated form, i.e. no individual data will appear in the supplemental report.

Destruction of samples and key code list for the DNA sample ID after a defined time period is described in detail in the text.

Appendix B: Administrative structures and responsibilities

This appendix lists the personnel and organizations involved in the study, according to the roles defined by SOP "Pharmacogenetic Analyses - Handling and Processing of Samples and Data"

Study number: _________

SAG SBU personnel or corresponding function in the Region (to be filled in)

Responsibility	Name	Affiliation / Address
Corporate Head of Clinical Development/ corresponding function in the Region		
Core Clinician/ corresponding function in the Region		
Study Manager/ corresponding function in the Region		
Study Biometrician/ corresponding function in the Region		
Key code list manager (CCO-SI)		
(if appropriate:) Other Study Management Personnel		

SAG CCO-SI personnel with access to the key code list for DNA sample ID

Responsibility	Name / Specifying Information	Affiliation / Address
Key code list manager		
(if appropriate:) Any other personnel with access to the Key Code List, and her/his role (i.e. reason for access)		

SAG Corporate Pharmacogenomics personnel

Responsibility	Name	Affiliation / Address
Head of Corporate Pharmacogenomics		
Person responsible for informing the Pharmacogenetic Advisory Board and custody of the samples		

Central sample repository name and personnel

Responsibility	Name	Affiliation / Address
Main responsible person at the central sample repository		
Person with main responsibility for handling of the DNA sample IDs and key code list, respectively		
Other persons responsible for handling of samples, encryption and key code handling		

Appendix C: Confidentiality Agreement

According to the SOP, all persons having access to the key code list for the DNA sample IDs must confirm in writing that she/he will keep the confidentiality of the data, and that she/he will not undertake efforts to combine clinical and pharmacogenetic data except for procedures described in this SOP.

<table>
<tr><td colspan="2">Study No.: ______________</td></tr>
<tr><td colspan="2">I am aware of the processes involved in the set up and handling of the pharmacogenetic and clinical data and the importance of ensuring the confidentiality of these data.

I confirm that I will not use the key code list for purposes other than the statistical analysis described in the respective analysis plan reviewed by the Pharmacogenetics Advisory Board. I will maintain the confidentiality of the mentioned key code list and the individual data accessed through the use of it. I will only release aggregated results.</td></tr>
<tr><td>Name and role</td><td>Date and signature</td></tr>
<tr><td></td><td></td></tr>
<tr><td></td><td></td></tr>
<tr><td></td><td></td></tr>
<tr><td></td><td></td></tr>
<tr><td></td><td></td></tr>
</table>
