# Supplementary material for: Establishing Long-Term Efficacy in Chronic Disease: Use of Recursive Partitioning and Propensity Score Adjustment to Estimate Outcome in MS
Source: PLoS One. 2011 Nov 30;6(11):e22444. doi: 10.1371/journal.pone.0022444 (PMC3227563; doi:10.1371/journal.pone.0022444)
Supplement: Appendix S1 — (DOC) [file pone.0022444.s014.doc]

**Supplemental Material**

**Appendix S1**

As noted in the main text, when attempting to determine therapeutic impact from long-term follow-up studies, there are several potential sources of bias that require mitigation (Table S1). One of the most important of these biases is due to “informed censoring” which can markedly inflate the estimated benefit of therapy. This bias results from the fact that patients who are doing well will tend to continue whatever therapy they are taking whereas patients who are doing poorly will tend to stop or switch therapy. In order to mitigate this bias, we used the so-called “medication possession ratio” or MPR [18, 19] together with exposure weighting.

**Medication Possession Ratio (MPR) and Exposure Weighting**

As noted in the main text, the first step (Table S2; Step1, A) was to transform the variable of IFNβ-1b 250 µg treatment-exposure (in years) into the MPR. The purpose of this transformation was to compensate for the bias introduced by physicians or patients making treatment decisions based on perceived efficacy (i.e., outcome-dependent censoring of exposure) [20]. Thus, the MPR was defined individually as:

MPR = (Actual-time of IFNβ-1b exposure) / (Total-time possible for IFNβ-1b exposure)

Here the total-time possible for IFNβ-1b 250 µg treatment-exposure is taken from the time of randomization in the RCT until the time of reaching the end-point being considered. This transformed variable is referred to as the “raw” or “unweighted” MPR. These “raw” MPRs were then weighted (as discussed below) to account optimally for the possibility that a patient’s response to therapy might be different based on how long they had had their illness and how disabled they were when therapy was started. For the denominator of the “raw” MPR, time began at RCT-entry and ended when a particular negative-outcome was reached or at the time of data-censoring. Thus, exposure was only counted up until the event-of-interest or, if the person never reached this endpoint, until the LTF evaluation. The purpose of using the MPR transformation is to mitigate the bias caused by the tendency for patients who are doing well to continue on a particular therapy and for those who are doing poorly to either switch therapies or stop entirely [20]. As an example of this method, consider two patients, each beginning therapy at the same disease-stage. Suppose that therapy is ineffective and that any differences in outcome are due solely to the intrinsic difference in the rate of disease progression between the two patients. Finally, suppose that the first patient develops SPMS after two years, discontinues medication, and 1 year thereafter reaches EDSS=6, whereas the second patient develops SPMS after 10 years, discontinues medication, and 5 years thereafter reaches EDSS=6. Although these patients differ only in their intrinsic rate of disease progression, and each pursues the same treatment strategy, the second patient has five-times the treatment-exposure of the first patient, making it appear (spuriously) that therapy is advantageous. In this case, the MPR transformation mitigates this bias completely because, for both patients, the MPR is identical (100% for the SPMS outcome and 67% for the EDSS≥6 outcome).

Consider, also, a related (but more complicated) second example, in which two sets of two patients have two different intrinsic progression rates (e.g., 3 and 15 years to EDSS≥6). For the sake of this example, we will suppose that therapy is effective and that it doubles the time to reaching EDSS≥6 (or any intermediate stage). Suppose that the first set of patients (patient #1 with at rapid course and patient #2 with a slow course) take medication regularly until they reach EDSS≥6 (which, therefore, occurs after 6 and 30 years respectively), whereas the second set of patients discontinue medications after 2 years (for patient #3 with the rapid course) and after 8 years (for patient #4 with the slow course). In this circumstance, patients #3 and #4 reach the EDSS≥6 outcome after 5 and 23 years respectively. Clearly, in this situation, a relationship between the number of years on therapy and outcome will be found. Thus, the ordering for years of exposure and time-to-EDSS≥6 are identical (patient #2 > #4 > #1 > #3) and, therefore, these two variables will be highly correlated (in this example; ρ = 0.84). Nevertheless, although this analysis leads to the conclusion that there is a treatment benefit, the actual estimate of that benefit is biased because the treatment effect is still entangled with the disease course. Thus, because only patients #1 and #2 derive the full benefit of therapy, and because patient #3 has been treated for a greater proportion of her expected survival-time than has patient #4, the correct ordering for the derived treatment benefit, under the assumptions of this example, will be (patient #1 = #2 > #3 > #4). As before, the MPR transformation (100%, 100%, 40%, and 35% for patients 1-4 respectively) disentangles the pace of disease from the benefit of therapy and results in the correct ordering for the derived therapeutic benefit.

The efficiency of the MPR at mitigating this bias is amply demonstrated by comparison of an analysis using the actual (unweighted) years of 250 µg IFNβ-1b-exposure to the same analysis following MPR transformation. Using the actual treatment-exposure-years (Figure 1 – A; Main Paper), the principal variable identified by the recursive partitioning (RP) algorithm [21, 22] was treatment-exposure (p<10^-16^). However, when the exposure was transformed into an “unweighted” or “raw” MPR, the RP analysis splits only on EDSS and there is no significant residual exposure-effect (Figure 1-B ; Main Paper), indicating that the original observation was predominantly due to bias from undefined sources and that this bias has been dramatically reduced by the MPR transformation. Only after the possibility of exposure-weighting is introduced (see below), does treatment re-emerge as significant factor associated with outcome (Figures 1-B and 2; Main Paper).

Exposure-weighting was accomplished using families of Bass diffusion curves [23] and exponential functions. Bass diffusion curves are S-shaped response functions that were initially used to describe the uptake of novel technology by a population consisting of early and late adopters but have found wide applicability to many other circumstances [23]. These exponential and Bass curves were used to adjust raw-treatment-exposure (MPR) either up or down based on disease-duration and EDSS at the time of treatment-initiation (Table S2; Step 1, B). Nine curves were generated for each family although the zero curve for both families is the same straight line (Figure S2). Preliminary analyses found that linear adjustments were inferior to the curvilinear weighting-schemes. However, the actual choice of Bass and exponential functions (as opposed to other S-shaped and curvilinear functions) was arbitrary. Nevertheless, these two families of curves permitted a wide range of exposure-response behaviors to be considered by the RP algorithm and, thus, allowed considerable flexibility in selecting a weighting-scheme (Figure S2). If a person stopped therapy for ≥3 months and then restarted, new weights were calculated and applied to the second treatment phase and the weighted-MPRs from the two different phases summed to give the final weighted-MPR estimate.

Exposure-weighting curves were always selected in pairs, one for the time since first symptom and the other for EDSS prior to start of therapy (Figure S3). The final, combined treatment-exposure weight was the average of the weights taken from the selected pair. Different pairs could be selected for different outcomes although, because of computational limitations, both members of a pair had to come from the same family (i.e., either Bass or exponential). This allowed for 161 possible weighting-schemes, which were applied to each patient’s raw-MPR to calculate a set of weighted-MPRs for each weighting-scheme.

All of these weighted-MPR exposures were input to an RP algorithm to predict the time-to-negative-outcome (Table S2; Step2, A). The RP method is an automated unbiased and well characterized (reproducible) search technique, which is used to select variables and cut-points based on their relationship to outcome. [21, 24-26]. RP methods have certain advantages over more traditional regression models in that they make no distributional assumptions about the data (e.g., linearity, normality, etc), use an unbiased selection process to create categories, and handle interactions automatically. Moreover, RP methods can be applied easily to survival data and they always create discrete treatment groups, which are necessary for propensity-adjusted analyses.

**Sensitivity Analyses**

The stability of the relationship between exposure-group and negative-outcome was tested by multiple sensitivity analyses using different definitions of “hard” negative-outcome (i.e., EDSS≥6, SPMS, wheelchair use, death, either EDSS≥6 or SPMS, and “any negative-outcome”), different assumptions about the underlying data, and different modeling approaches (Table S2; Step 4, A). For example, in our principal analysis, when drug exposure start or stop dates were missing for individual patients, we used dates assuming a minimum-exposure. In our sensitivity analyses, however, we used maximum-exposure instead. Similarly, in our principal analysis, the 3-month confirmed EDSS≥6 was used. For one of our sensitivity analysis (the EDSS override condition), however, the patient with an EDSS≥6 at the LTF was considered to have reached this end-point either on the first date during the trial in which an EDSS≥6 was observed (if this happened) or on the date of the LTF examination. Moreover, propensity score adjustment was conducted with 2-5 bins. In addition, several adjustment techniques were tested, including standard linear adjustment for baseline EDSS, standard categorical adjustment for baseline EDSS, and no adjustment for the baseline characteristics.

The stability of the weighting scheme selection was investigated using a bootstrap approach, in which 2000 repetitive (and random) samples were taken from the total observations (260) and the RP analysis run on each sample (Figure S4). In this method there were 260 patients in each sample although, for any given sample, some patients’ data may have been replicated whereas other patients’ data may not have been included.

The stability of the effect-size estimate for the treatment effect was investigated performing the entire analysis on 2000 repetitive random samples of 164 patients (63.2% of the total population) and using three different Cox-modeling strategies (Figure S5). The first was the same as for our principal analysis and used a 5-bin propensity score adjustment including all baseline factors as potential predictors of treatment group membership. The second utilized a stepwise selection including all baseline factors as potential covariate predictors of outcome. The third used no adjustment and only treatment group membership (high or low) was entered into the model. The results of each of these analyses (grouped according to the weighting scheme selected for a particular random sample) were compared to the “original” estimate of the effect-size, which was based on the entire 260 patient cohort, the bTN4SN2 weighting scheme (Figure S3), and a 5-bin propensity adjustment. The predictive accuracy of each model and each weighting scheme were compared by calculating the area underneath the receiver-operator curve for each model using Harrell’s c-index [33]. The predictive power for those observations (63.2%) that were used in the development of each model (i.e., “In Bag” observations) were compared to the predictive power for those observations (36.8%) that were not included during model development (i.e., “Out of Bag” observations).

**Results of the Sensitivity Analyses**

Results from every sensitivity analyses confirmed this finding. For every negative-outcome the benefit of treatment was very similar and highly significant (Figure 3; Main Paper). Because time-to-failure data were available for only 7/35 deceased-patients (Table 1; Main Paper), mortality in the LTF is considered eslewhere.^14^ Remarkably, in each of these analyses, the same two Bass response curves (bTN4SN2) were chosen, except for the SPMS analysis where the bN3 curve was chosen for EDSS at treatment-onset instead of bN2 (Table S3 and Figure S2). Furthermore, changing the assumptions about using the EDSS override or the maximum possible exposure did not alter the result (Table S3). Even considering other disease modifying therapies in the calculation of the raw-MPRs for each patient did not alter the findings. The results were identical when including “any-DMT” exposure and no treatment effect was found when IFNβ-1b exposure was excluded.

Using the bootstrap method, the selected weighting scheme was quite stable (Figure S4). Thus, 52% of the time the exact same scheme was selected and, in over 90%, the selected weighting schemes (bTN4SN3, eTN4SN3, eTN4SN4, and bTN1SN3) were very similar (Figure S4). Also, using three different Cox-modeling strategies to estimate the effect sizes, the predictive power for those observations that were used in the development of each model were quite comparable to the predictive power for those observations that were not included during model development (Figure S5). Moreover, even when each of these different weighting schemes is forced into the analysis (Table S2; Step 2, B) and the remaining steps of the analysis carried out (Table S2), the high-exposure-group is still consistently less likely to experience “any negative-outcome” compared with the low-exposure-group (p<0.0001 for each weighting scheme).
